# Synthesis of Multisubstituted 1,2,3-Triazoles: Regioselective Formation and Reaction Mechanism

**DOI:** 10.1021/acs.joc.3c02836

**Published:** 2024-03-28

**Authors:** Tzu-Ching Chi, Po-Chun Yang, Shao-Kung Hung, Hui-Wen Wu, Hong-Chi Wang, Hsin-Kuan Liu, Li-Wen Liu, Ho-Hsuan Chou

**Affiliations:** † Department of Chemistry, National Cheng Kung University, Tainan 701, Taiwan; ‡ Core Facility Center, National Cheng Kung University, Tainan 701, Taiwan; § National Tainan First Senior High School, Tainan 701, Taiwan

## Abstract

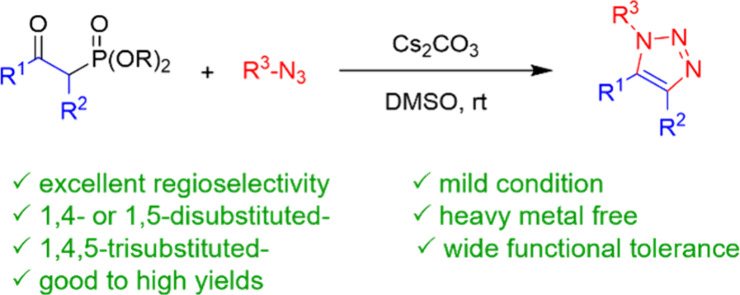

A synthetically useful approach to functionalized triazoles is described via the reaction of β-carbonyl phosphonates and azides. 1,4- and 1,5-disubstituted and 1,4,5-trisubstituted triazoles can be regio- and chemoselectively accessed under mild conditions in good to excellent yields (31 examples, up to 99%). A mechanism is proposed that rationalizes the avoidance of the 4-phosphonate byproducts, which is aligned with crystallographic and experimental evidence.

## 1 Introduction

**Scheme 1:**
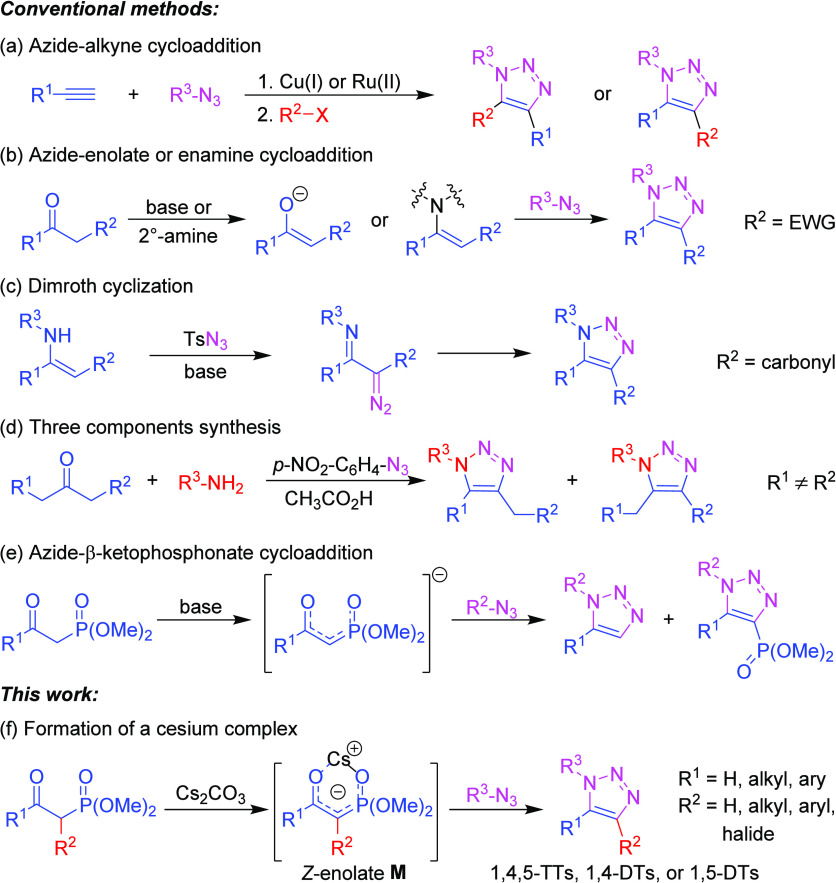
Synthesis of Multisubstituted 1,2,3-Triazoles

1,2,3-Triazoles, 5-membered heterocycles with three contiguous nitrogen atoms, have found applications in diverse areas such as organic synthesis, chemical biology, and material science.^1^ Their reactivity and promising bioactivity are highly dependent on the pattern and nature of substituents.^1a,1b,2^ Cu(I)- and Ru(II)-catalyzed 1,3-dipolar cycloadditions of azides with alkynes are conventionally used to synthesize 1,4- and 1,5-disubstituted triazoles (1,4-DTs and 1,5-DTs), respectively. To further achieve 1,4,5-trisubstituted triazoles (1,4,5-TTs), haloalkynes or aryl halides are introduced into the reaction to trap the in situ generated copper(I) triazolide intermediate for cascade coupling reactions. However, a well-designed ligand coordinating with a transition metal is paramount in minimizing the formation of undesired 1,4-DT byproducts (Scheme 1a).^3^

Highly strained cycloalkynes undergo azide–alkyne cycloadditions to furnish functionalized 1,2,3-triazoles under metal-free conditions.^4^ Further development led to the use of activated dipolarophiles such as enamines,^5^ enolates,^6^ or alkenes^7^ to accelerate the 1,3-dipolar cycloaddition with alkyl or aryl azides, as well as to synthesize a diazo imine intermediate using the Regitz diazo-transfer reagent for Dimorth cyclization. Nonetheless, the restriction of the activating groups (carbonyl or aryl moieties are required at the 4- or 5-position) limits the aforementioned protocols for generating 1,4,5-TTs (Scheme 1b,c).^5a−5d,8^ Furthermore, a lack of regioselectivity is observed with asymmetrical ketones (Scheme 1d).^5f^

Recently, 1,5-DTs were reported to be accessible via the reaction of β-ketophosphonates and azides mediated by KOH. However, to achieve good to excellent yields in the cyclization process, R^1^ was limited to isopropyl-, *t*-Bu-, *c*-hexyl-, or aryl substituents (Scheme 1e).^9^ In contrast, with R^1^ = Me, 1,5-DTs were obtained in very low yields. The innate steric substituent of R^1^ was crucial to induce a *syn*-orientation of the alkoxy anion and the phosphonate of the triazoline intermediate to facilitate phosphonate elimination in the subsequent Horner–Wittig type process; otherwise, the formation of the undesired 4-phosphonated-1,5-trisubstituted triazole dominated owing to competing water elimination.^9b^

In this study, we demonstrate that cesium carbonate serves as an effective base for promoting the reaction between β-carbonyl phosphonates and azides, yielding a variety of 1,4-/1,5-DTs and 1,4,5-TTs with good to excellent yields at room temperature (Scheme 1f). Meanwhile, X-ray crystallography and NMR spectroscopy analyses confirmed the involvement of a cesium-chelated intermediate in these [3 + 2] cycloaddition reactions, which significantly influences the chemo- and regioselectivity of the 1,2,3-triazole products.

## 2 Results and Discussion

The base required for the efficient generation of 1,4,5-TT **3aa** from α-benzyl-β-ketophosphonate **1a** and phenyl azide **2a** was systematically studied at room temperature (Table 1). Amines such as DBU, TMG, TEA, and piperidine, along with K_2_CO_3_, were ineffective in CH_3_CN (Table 1, entries 1–5). Whereas KOH in CH_3_CN was suitable for preparing 1,5-DTs,^9a^ it resulted in a very low conversion (12%; entry 6); conversely, Cs_2_CO_3_ more than doubled the yield (32%; entry 7). A change to polar aprotic solvents increased the solubility of Cs_2_CO_3_ and significantly enhanced the efficacy of the heterogeneous reactions (entries 11–13), with DMSO being more effective (95%) than DMF (73%). While Cs_2_CO_3_ was observed to be superior to other alkali carbonates (entries 12–15), providing triazole product **3aa** in good isolated yields (95% conversion), almost no product was obtained using strong bases such as NaHMDS or KHMDS in THF (entries 19 and 20).

**Table 1:**
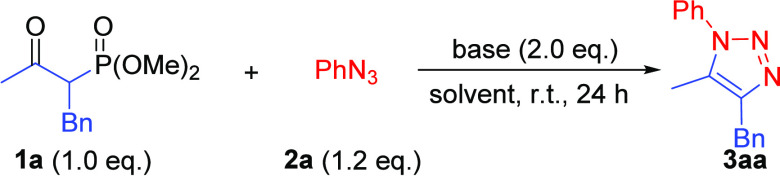
Optimization of Reaction Conditions

entry	solvent	base	concn^a^ (%)	entry	solvent	base	concn^a^ (%)
1–4^e^	MeCN	DBU, TMG, TEA, or PPR^b^	n.r.	12	DMSO	Cs_2_CO_3_	95
				13	DMSO	K_2_CO_3_	73
5	MeCN	K_2_CO_3_	n.r.	14	DMSO	Na_2_CO_3_	6
6	MeCN	KOH	12	15	DMSO	Li_2_CO_3_	<1
7	MeCN	Cs_2_CO_3_	32	16	DMSO	CsF	72
8	THF	Cs_2_CO_3_	21	17^e^	DMSO	DBU	9
9	DCM	Cs_2_CO_3_	n.r.	18	DMSO	Cs_2_CO_3_^c^	79
10	MeOH	Cs_2_CO_3_	n.r.	19^e^	THF	NaHMDS	n.r.
11	DMF	Cs_2_CO_3_	73	20^e^	THF	KHMDS	4

a The conversion was determined from the crude product through the integration ratio of ^1^H NMR.

b DBU = 1,8-diazabicyclo[5.4.0]undec-7-ene, TMG = tetramethylguanidine, TEA = triethylamine, PPR = piperidine.

c 1.2 equiv of Cs_2_CO_3_ was used.

e The reaction is homogeneous.

We next focused on the scope and limitations of the reaction under the optimized conditions (Table 2). The reactions of **1a** with aryl azides **2a**–**f** at room temperature furnished the corresponding 1,4,5-TTs **3aa**–**af** in good to excellent yields (69–96%; Table 2a). While both electron-deficient and -rich aryl azides were accommodated, the latter were less effective, but this was readily ameliorated by warming the reaction to 60 °C (cf. **3aa** and **3ad**). Neither benzyl azide **2h** nor alkyl azide **2i** underwent cyclization with phosphonate **3a**, showing no reaction at room temperature for 24 h or at 60 °C for 6 h. Additionally, extended heating led to the gradual decomposition of phosphonate **3a**.

**Table 2:**
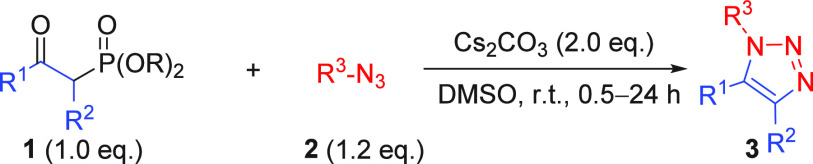

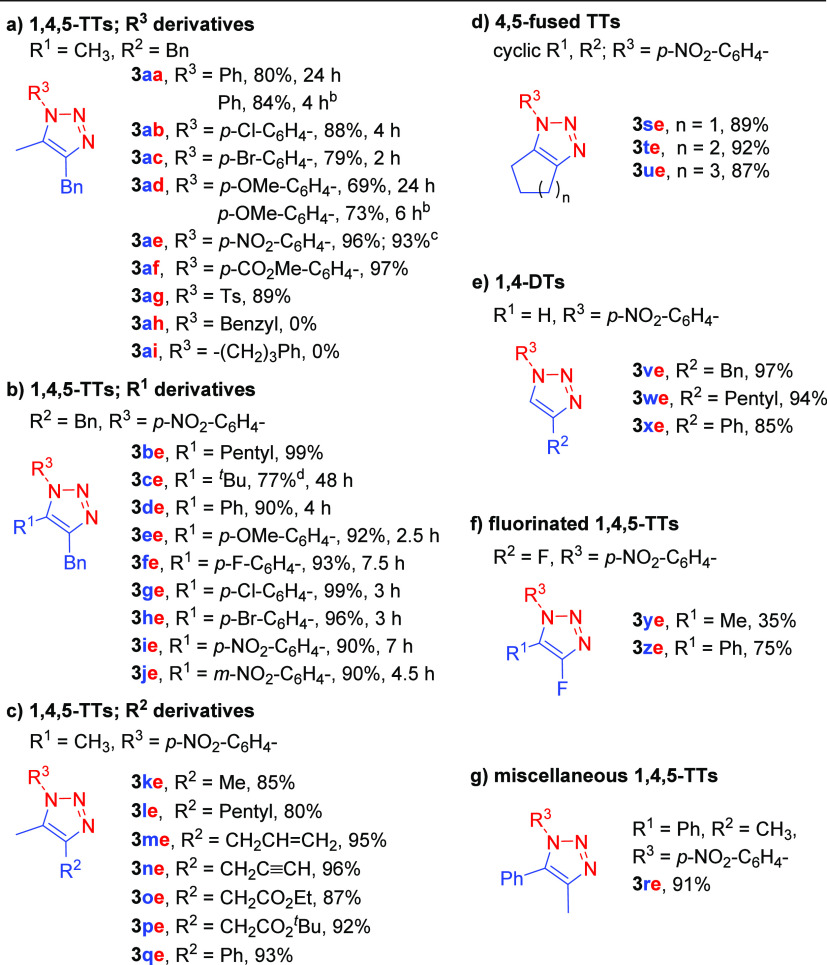
Synthesis of Diverse 1,4,5-TTs, 1,4-DTs, and 1,5-DTs^a^

a The reaction was completed at rt in 0.5 h unless the reaction time is indicated.

b The reaction was performed at 60 °C.

c The reaction was performed at 1.02 mmol scale based on **1a**.

d Starting material **1c** was recovered in 13% yield.

In contrast to previous literature precedence,^9^ the nature of R^1^ in phosphonates was no longer limited to Me; *n*-pentyl, *t*-butyl and aryl groups were tolerated and provided the desired TTs **3be**–**je** in good yields (77–99%; Table 2b). The presence of electron-donating or -withdrawing group on the benzoyl group is favored for the reaction (**3ee**–**je**) and demonstrates good reactivity toward the aryl azide **2e**. However, a bulky substituent (R^1^ = *t*-Bu, **3ce**) slowed the formation of planar *Z*-enolate, thereby decelerating the rate of the 1,3-dipolar cycloaddition process. The chemoselectivity of the substituents at the 4-position of triazole (R^2^) was systematically examined by the reaction of α-substituted-β-ketophosphonates **1k**–**q** with *p*-nitrophenyl azide **2e** (Table 2c). Terminal alkenyl, alkynyl, and ester groups were tolerated under the mild conditions used herein, furnishing the desired TTs **3me**–**pe** in good to excellent yields (87–96%). The formation of **3qe** and **3re** (Table 2g) individually shows that the orientation of R^1^ and R^2^ can be switched by the judicious choice of reaction partners. The demand for electrophilic dipoles and the preference for electron-rich dipolarophiles indicate that the 1,3-dipolar cycloaddition between azides and β-ketophosphonates is dominated by the LUMO_dipole_–HOMO_dipolarophile_ interaction.^10^

Previous reports indicated that fused bicyclic triazoles were obtained using highly strained cycloalkyne species^11a^ or extreme conditions (heat/sealed at 80 °C for an extended time).^11b−11d^ Under our developed conditions, the exposure of **2e** to cyclic β-ketophosphonates comprising a cyclopentyl, cyclohexyl, or cycloheptyl moiety formed 4,5-fused triazoles **3se**, **3te**, or **3ue**, respectively, in high yields (87–92%; Table 2d). Upon deprotonation, these cyclic phosphates consistently yield *Z*-enolates. By replacing Cs_2_CO_3_ with 5 M KOH (aq) as the base in a DMSO solution, the efficient synthesis of bicyclic triazole **3te** was achieved, yielding 87% within 30 min. 1,4-DTs **3ve**–**xe** (Table 2e) and 4-fluorinated 1,4,5-TTs **3ye** and **3ze** (Table 2f) were successfully synthesized by the reactions of **2e** with appropriately substituted 2-diethoxyphosphorylacetaldehydes **1v**–**x** and α-fluoro-β-ketophosphonates **1y**–**z**, respectively. Although fluorinated heterocycles are crucial to the pharmaceutical and agricultural industries,^12^ few reports describe the synthesis of fluorinated triazoles.^13^ Our developed synthetic method conveniently and effectively produces this important class of compounds without stringent conditions or the need for excess fluoride sources.

**Figure 1:**
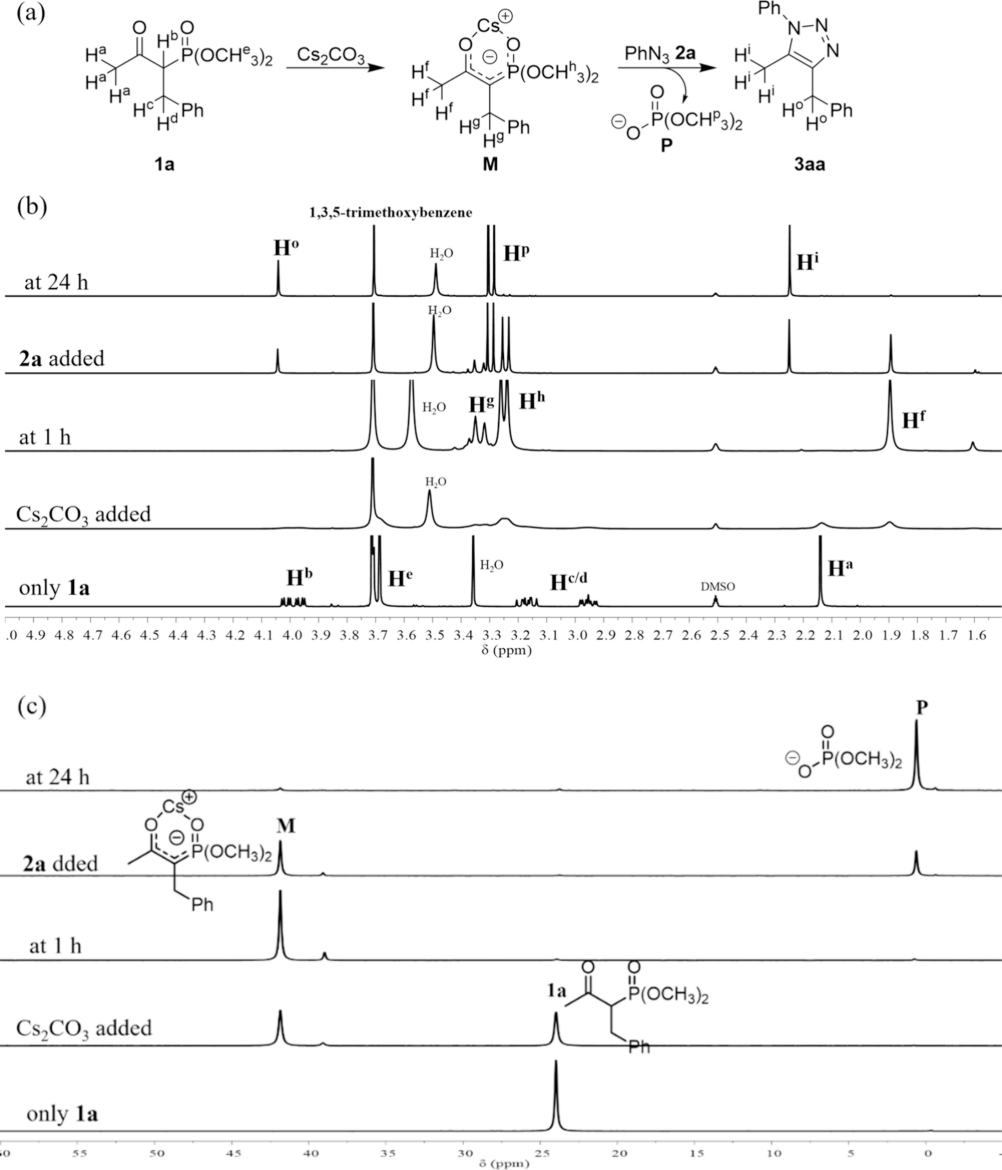
Reaction was monitored via NMR in 0.68 mL of DMSO-*d*_6_ with **1a** (0.136 mmol), **2a** (0.163 mmol), and Cs_2_CO_3_ (0.272 mmol). (a) Proposed cesium-chelated intermediate **M**. (b) ^1^H NMR and (c) ^31^P NMR spectra of triazole **3aa** formation.

**Figure 2:**
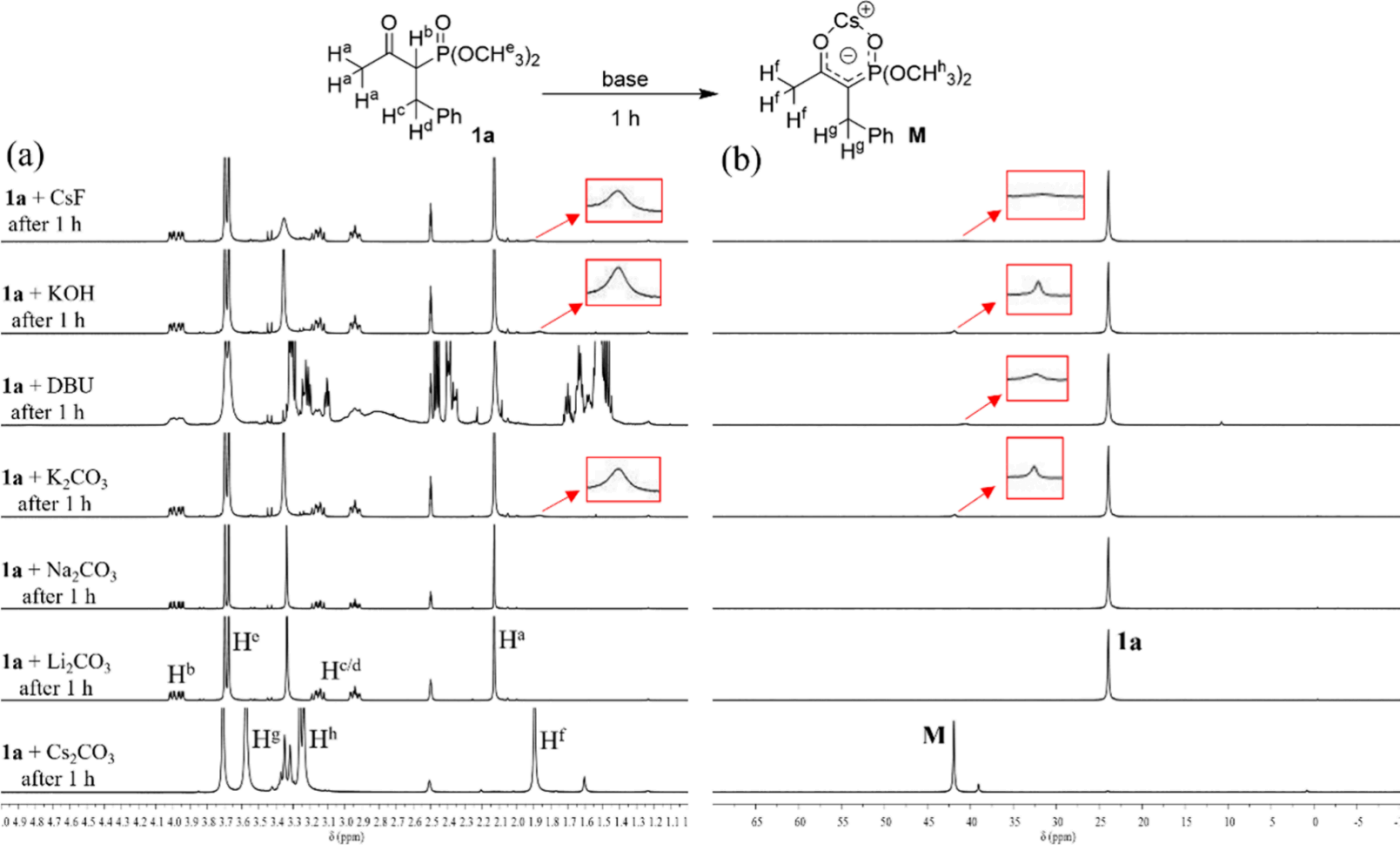
Reaction was monitored via NMR in 0.68 mL DMSO-*d*_6_ with **1a** (0.136 mmol) and base (0.251 mmol). (a) ^1^H and (b) ^31^P NMR spectra for the base treatment of β-ketophosphonate **1a** after 1 h.

To gain insight into the role of the cesium cation, the reaction of **1a** with **2a** in DMSO-*d*_6_ was continuously monitored by ^1^H NMR (500 MHz) and ^31^P NMR (202 MHz) spectroscopy (Figure 1a–c). The signal α-H^b^ (δ = 3.98 ppm) clearly identifies **1a** as an β-ketophosphonate. After being treated with Cs_2_CO_3_(s) for 1 h, the signal attributed to α-H^b^ disappeared, indicating **1a** is readily deprotonated and converted to the cesium bound carbanionic intermediate **M** (Figure 1b, at 1 h).^14a^ Upon introduction of azide **2a**, the signals of intermediate **M** steadily disappeared and were replaced by that of triazole **3aa** and phosphate **P** (at 24 h). The ^31^P NMR spectra also supported the transformation of phosphonate **1a** (δ = 24.0 ppm) to phosphate **P** (δ = 0.6 ppm) through the chelated intermediate **M** (δ = 41.8 ppm) (Figures 1c and S1 and 2 in SI).^14b−14d^ In contrast, other alkali carbonates (Li_2_CO_3_, Na_2_CO_3_, or K_2_CO_3_) or even stronger bases (KOH, DBU, NaHMDS, or KHMDS) did not generate the corresponding carbanion chelate **M** sufficiently, and thus, **3aa** was not formed effectively (Table 1 and Figure 2)

**Figure 3:**
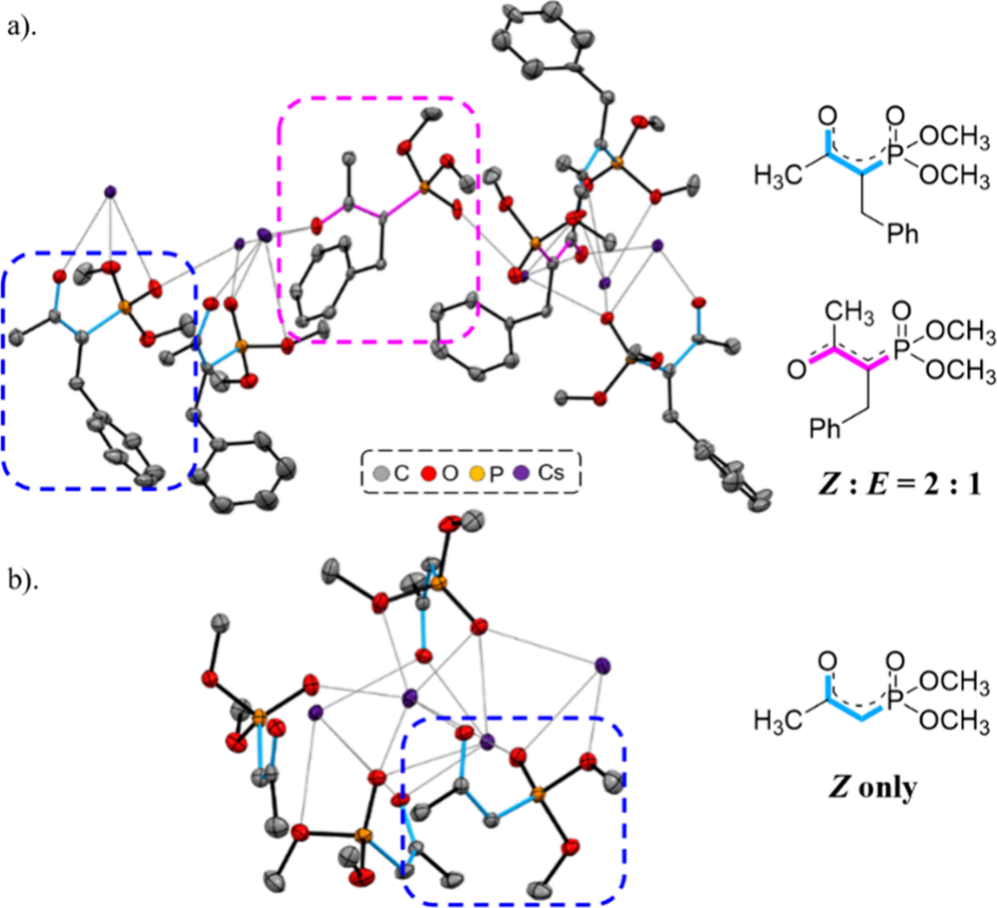
Single crystal X-ray structures of cesium enolate (a) **6** (CCDC 2156078) and (b) **7** (CCDC 2156080).

The advantage of Cs_2_CO_3_ over K_2_CO_3_ was evident when dimethyl (2-oxopropyl)phosphonate **4a** was used as the dipolarophile. Under the Cs_2_CO_3_-mediated conditions, 1,5-DTs **5** were formed exclusively in most cases with good to excellent yields (71–91%). In contrast, the chemoselectivity between **5** and undesired 4-phosphonated TT **5′** was reported low or even reversed (**5′ae**) in the presence of K_2_CO_3_ (Table 3).^9b^ The existence of the cesium-chelated intermediate was studied via the crystallization of **1a** and **4a** individually with Cs_2_CO_3_ to form **6** and **7**, respectively (as Cs atoms were severely disordered, hydrogen atoms in structure **6** could not be defined). The cesium enolate crystal structure of **6** showed a *Z*/*E* ratio of 2:1, while the Z conformation was observed exclusively for **7** (Figure 3). The individual exposures of **6** and **7** to **2a** in DMSO gave only **3aa** and **5aa**, respectively.

**Table 3:**
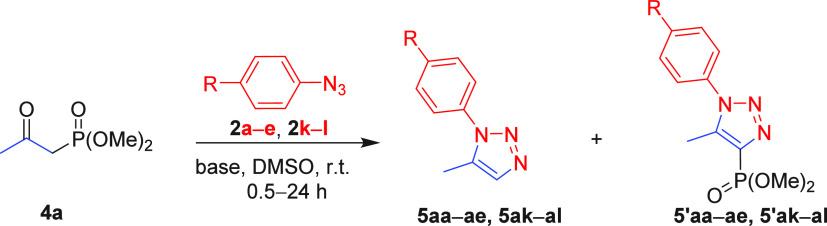

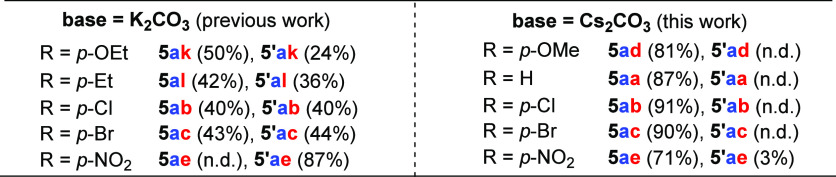
Comparison between K_2_CO_3_ and Cs_2_CO_3_

**Figure 4:**
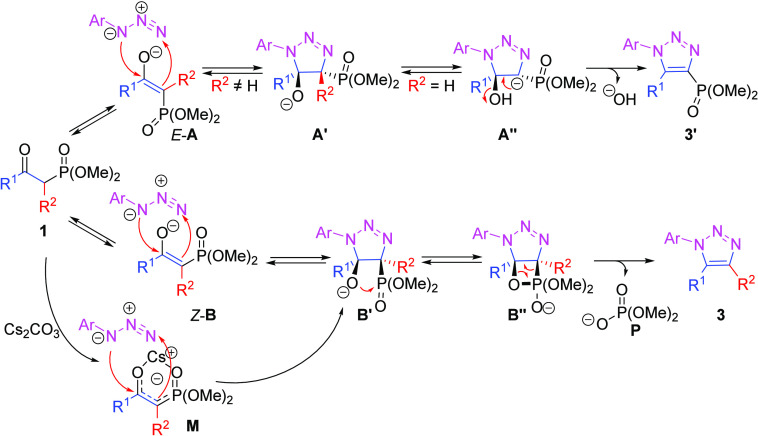
Proposed mechanism for the formation of 1,2,3-triazoles in the presence or absence of Cs_2_CO_3_.

A reaction mechanism is proposed, as depicted in Figure 4. In the absence of the chelation effect, the equilibrium between enolates *E*-**A** and Z-**B** is governed by the steric effect of R^1^ and R^2^, as well as by the interaction between the alkoxy anion and the phosphonate. Either the *E*- or *Z*-olefinic anion (*E*-**A** or *Z*-**B)**, acting as a dipolarophile, competitively reacts with an azide to form **3′** or **3**, respectively, via the triazoline intermediate **A′** or **B′**. After protonation and deprotonation, the orientation of **A′** with R^2^ = H tends to eliminate hydroxide and yield the 4-phosphonated triazole **3′**. The substituted R^2^ occupations (R^2^ ≠ H) is bias against the previous pathway and equilibrates the *Z*-**B** conformer that invariably forms **3**. However, in the presence of Cs_2_CO_3_, the enolate of β-ketophosphonate would preferentially bind in the *Z*-conformation as cesium enolate intermediate **M**, irrespective of the nature of R^2^. The resulting *syn*-orientation of the alkoxy anion and phosphonate facilitates the formation of the oxaphosphetane **B′′** and accelerates the HWE-type elimination to form **3**.

## 3 Conclusion

We found that Cs_2_CO_3_ in DMSO is a unique system to facilitate the formation of 1,2,3-triazoles from β-carbonyl phosphonates under mild conditions. The reaction is highly regioselective, with the formation of expected substituted triazolyl products in high yield. We demonstrated the cesium-chelated *Z*-enolate as an efficient dipolarophile in the [3 + 2] cyclization with an applied azide dipole. The protocol allows access to not only 1,4- or 1,5-DTs but also 1,4,5-TTs with various functional groups in good to excellent yields. This protocol successfully provided access to 1,4,5-TTs containing diverse functionalities at positions 4 or 5, which were previously not readily accessible.

## 4 Experimental Section

### General Procedure for the Synthesis of Multisubstituted 1,2,3-Triazoles

α-Substituted-β-ketophosphonate **1** (1.0 equiv, 0.3 M) and cesium carbonate (2.0 equiv) were mixed in DMSO for 10 min, and azide **2** (1.2 equiv, 0.3 M) in DMSO was injected into the resulting reaction. After the reaction was completed according to thin-layer chromatography (TLC), the solution was diluted with EtOAc (10 mL) and washed with brine (15 mL × 3) to remove DMSO. Occasionally, the product might be found in the aqueous layer. Then the aqueous layer could be extracted with additional EtOAc if needed. The organic layers were all combined, dried over anhydrous MgSO_4_, filtered, and concentrated under reduced pressure to give the crude residue. The residue was purified by flash column chromatography on silica gel to give the desired product.

## Data Availability

The data underlying this study are available in the published article and its Supporting Information.

## Supporting Information Available

Experimental procedures; FT-IR spectra, NMR spectra, mass spectra, and crystal data for all new compounds (PDF)

## References

[ref1] aKharb R.; Sharma P. C.; Yar M. S. Pharmacological Significance of Triazole Scaffold. J. Enzyme Inhib. Med. Chem. 2011, 26, 1–21. 10.3109/14756360903524304. 20583859

[ref2] Wuest F.; Tang X.; Kniess T.; Pietzsch J.; Suresh M. Synthesis and Cyclooxygenase Inhibition of Various (Aryl-1,2,3-Triazole-1-Yl)-Methanesulfonyl-phenyl Derivatives. Bioorg. Med. Chem. 2009, 17, 1146–1151. 10.1016/j.bmc.2008.12.032. 19157881

[ref3] aMeldal M.; Tornøe C. W. Cu-Catalyzed Azide–Alkyne Cycloaddition. Chem. Rev. 2008, 108, 2952–3015. 10.1021/cr0783479. 18698735

[ref4] aCodelli J. A.; Baskin J. M.; Agard N. J.; Bertozzi C. R. Second-Generation Difluorinated Cyclooctynes for Copper-Free Click Chemistry. J. Am. Chem. Soc. 2008, 130, 11486–11493. 10.1021/ja803086r. 18680289 PMC2646667

[ref5] aLi W.; Wang J. Lewis Base Catalyzed Aerobic Oxidative Intermolecular Azide–Zwitterion Cycloaddition. Angew. Chem., Int. Ed. 2014, 53, 14186–14190. 10.1002/anie.201408265. 25319520

[ref6] aShashank A. B.; Karthik S.; Madhavachary R.; Ramachary D. B. An Enolate-Mediated Organocatalytic Azide–Ketone [3 + 2]-Cycloaddition Reaction: Regio-selective High-Yielding Synthesis of Fully Decorated 1,2,3-Triazoles. Chem. – Eur. J. 2014, 20, 16877–16881. 10.1002/chem.201405501. 25367870

[ref7] aKayet A.; Pathak T. 1,5-Disubstituted 1,2,3-Triazolylation at C1, C2, C3, C4, and C6 of Pyranosides: A Metal-Free Route to Triazolylated Monosaccharides and Triazole-Linked Disaccharides. J. Org. Chem. 2013, 78, 9865–9875. 10.1021/jo401576n. 24050453

[ref8] aCheng G.; Zeng X.; Shen J.; Wang X.; Cui X. A Metal-Free Multicomponent Cascade Reaction for the Regiospecific Synthesis of 1,5-Disubstituted 1,2,3-Triazoles. Angew. Chem., Int. Ed. 2013, 52, 13265–13268. 10.1002/anie.201307499. 24227395

[ref9] aGonzález-Calderón D.; Fuentes-Benítes A.; Díaz-Torres E.; González-González C. A.; González-Romero C. Azide–Enolate 1,3-Dipolar Cycloaddition as an Efficient Approach for the Synthesis of 1,5-Disubstituted 1,2,3-Triazoles from Alkyl/Aryl Azides and β-Ketophosphonates. Eur. J. Org. Chem. 2016, 2016, 668–672. 10.1002/ejoc.201501465.

[ref10] aSustmann R.; Trill H. Substituent Effects in 1,3-Dipolar Cycloadditions of Phenyl Azide. Angew. Chem. Chem., Int. Ed. Engl. 1972, 11, 838–840. 10.1002/anie.197208382.

[ref11] aYoshida S.; Karaki F.; Uchida K.; Hosoya T. Generation of Cycloheptynes and Cyclooctynes via a Sulfoxide–Magnesium Exchange Reaction of Readily Synthesized 2-Sulfinylcycloalkenyl Triflates. Chem. Commun. 2015, 51, 8745–8748. 10.1039/C5CC01784J. 25882340

[ref12] aShin H.-N.; Seo S. H.; Choo H.; Kuem G.; Choi K. I.; Nam G. Synthesis and Antibacterial Activities of New Piperidine Substituted (5*R*)-[1,2,3]Triazolylmethyl and (5*R*)-[(4-F-[1,2,3]Triazolyl)Methyl] Oxazolidinones. Bioorg. Med. Chem. Lett. 2013, 23, 1193–1196. 10.1016/j.bmcl.2013.01.033. 23385213

[ref13] aJana S.; Adhikari S.; Cox M. R.; Roy S. Regioselective Synthesis of 4-Fluoro-1,5-Disubstituted-1,2,3-Triazoles from Synthetic Surrogates of α-Fluoroalkynes. Chem. Commun. 2020, 56, 1871–1874. 10.1039/C9CC09216A. 31950943

[ref14] aWu B.; Chen H.; Gao M.; Gong X.; Hu L. Synthesis of 1,3-Aminoalcohols and Spirocyclic Azetidines via Tandem Hydroxymethylation and Aminomethylation Reaction of β-Keto Phosphonates with *N*-Nosyl-*O*-(2-Bromoethyl)Hydroxylamine. Org. Lett. 2021, 23, 4152–4157. 10.1021/acs.orglett.1c01091. 33999643

