# Supplementary material for: Synthesis of Multisubstituted 1,2,3-Triazoles: Regioselective Formation and Reaction Mechanism
Source: J Org Chem. 2024 Mar 28;89(8):5401–8. doi: 10.1021/acs.joc.3c02836 (PMC11040735; doi:10.1021/acs.joc.3c02836)
Supplement: Supplementary file 1 — jo3c02836_si_001.pdf [file jo3c02836_si_001.pdf]

Supporting Information for

Synthesis of Multisubstituted 1,2,3-Triazoles: Regioselective Formation and Reaction Mechanism

Tzu-Ching Chi,[a] Po-Chun Yang,[a] Shao-Kung Hung,[a] Hui-Wen Wu,[a] Hong-Chi Wang,[a] Hsin-Kuan Liu,[b] Li-Wen Liu,[c] and Ho-Hsuan Chou [a,*]

[a] *Department of Chemistry, National Cheng Kung University, Tainan 701, Taiwan.*

[b] *Core Facility Center, National Cheng Kung University, Tainan 701, Taiwan.*

[c] *National Tainan First Senior High School, Tainan 701, Taiwan.*

Phone: (+886)-6-2757575-65350
Fax: (+886)-6-2740552
Email: hohsuan@ncku.edu.tw

Table of Contents

1. General information………………………………………………………... S3
2. Time course of ^{1}H- and ^{31}P-NMR for the reaction of [3+2] cycloaddition….. S4
3. Using NaHMDS or KHMDS as a base for the triazole synthesis……….…. S5
4. Experimental procedures and characterization data …….……….……….. S5

   4.1 General procedure for the synthesis of compound **4**…………………...… S5

   4.2 General procedure for the synthesis of compound **1**……….................... S11

   4.3 General procedure for the synthesis of compound **2**……….................... S26

   4.4 General procedure for the synthesis of compound **3** or **5**..……….......... S29
5. SC-XRD Structure analysis…………………………………………….. S49
6. Reference……………………………………………………………... S50
7. NMR spectra……………………………………………………………. S52
8. Crystal data and structure refinement……………………………………… S118

1 General information

Chemical reagents were obtained from chemical suppliers and used without further purification. Acetonitrile, dichloromethane, and tetrahydrofuran were distilled over calcium hydride. Hexane and ethyl acetate were purified by reduced pressure and stored with 4Å molecular sieve. Other anhydrous solvents were purchased from the suppliers. Chromatography was performed with columns of 60 μm silica gel (FUJI). Thin-layer chromatography (TLC) were performed on the plates of Silicagel 60 F_{254} (Merck). Products were visualized by using UV radiation at 254 nm or staining with aqueous solution of potassium permanganate. The phrase "concentrated under reduced pressure" refers to the removal of solvents and other volatile materials using a rotary evaporator. Residual solvents were removed from samples at high vacuum.

^{1}H NMR spectra were recorded by Brucker spectrometer 400 or 500 MHz. ^{13}C{^{1}H} NMR spectra were recorded by Brucker spectrometer 100 or 125 MHz. ^{19}F{^{1}H} NMR spectra were recorded by Brucker spectrometer 376 ,470, or 658 MHz. ^{31}P{^{1}H} NMR spectra were recorded by Brucker spectrometer 162 or 202 MHz. Standard abbreviations of spin multiplicity such as s, d, t, q, p, sext, sept, m, br, dd, td, ABq were respectively referring to singlet, doublet, triplet, quartet, pentet, sextet, septet, multiplet, broad, doublet of doublet, triplet of doublet, AB quartet. Chemical shifts are reported in ppm with a solvent resonance as an internal standard (^{1}H NMR; TMS, DMSO-d_6, $CDCl_3$, as internal standard, indicating 0, 2.50, 7.26 ppm, respectively, ^{13}C{^{1}H} NMR; $CDCl_3$, as internal standard, indicating, 77.16 ppm.) As for ^{31}P{^{1}H} NMR spectra, PPh_3 (-6.0 ppm) was used as an external reference standard and the recorded s.r. value (s.r. = 70.02) served as manual calibration to every ^{31}P{^{1}H} NMR spectra.

Infrared spectra were obtained from Perkin-Elmer Spectrum RX FTIR spectrometer. All the absorption maxima value are expressed in wavenumber (cm^{-1}). For mass spectra, Electrospray Ionization (ESI) were collected on a High Resolution Orbitrap Mass Spectrometer Tandem Liquid Chromatography and Electrophoresis Analysis System (brand: Thermo Fisher Scientific Inc.; model: Q-Extractive Plus). Electrospray Ionization (ESI) were also collected on High Performance Liquid Chromatography Tandem Mass Spectrometer (brand: Varian; Model: VARIAN 901-MS). Melting point was measured on BUCHI Melting Point B-450.

2 Time course of ^{1}H- and ^{31}P-NMR for the reaction of [3+2] cycloaddition[a]

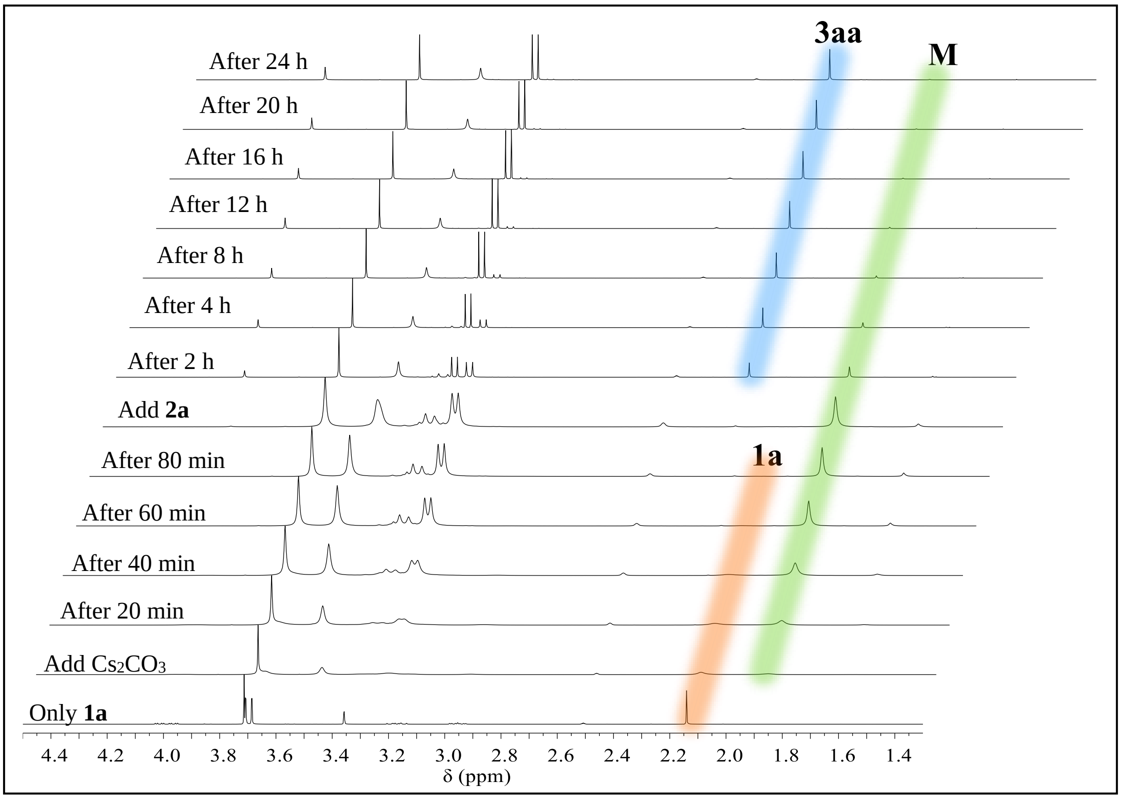

Figure S1. Time course of cesium cation chelation and [3+2] cycloaddition on ^{1}H NMR

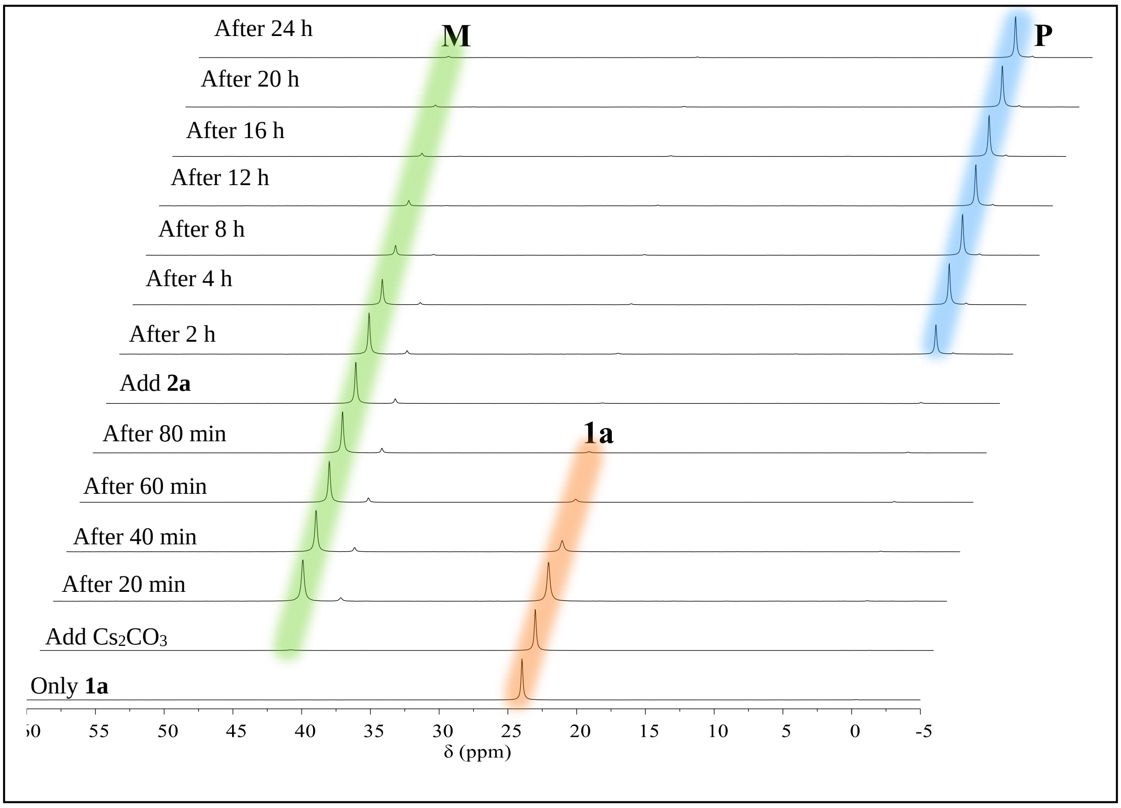

Figure S2. Time course of cesium cation chelation and [3+2] cycloaddition on ^{31}P NMR

[a]The reaction was monitored via NMR in 0.68-mL DMSO-d_6 with 1a (0.136 mmol), **2a** (0.163 mmol), and Cs_2CO_3 (0.272 mmol).

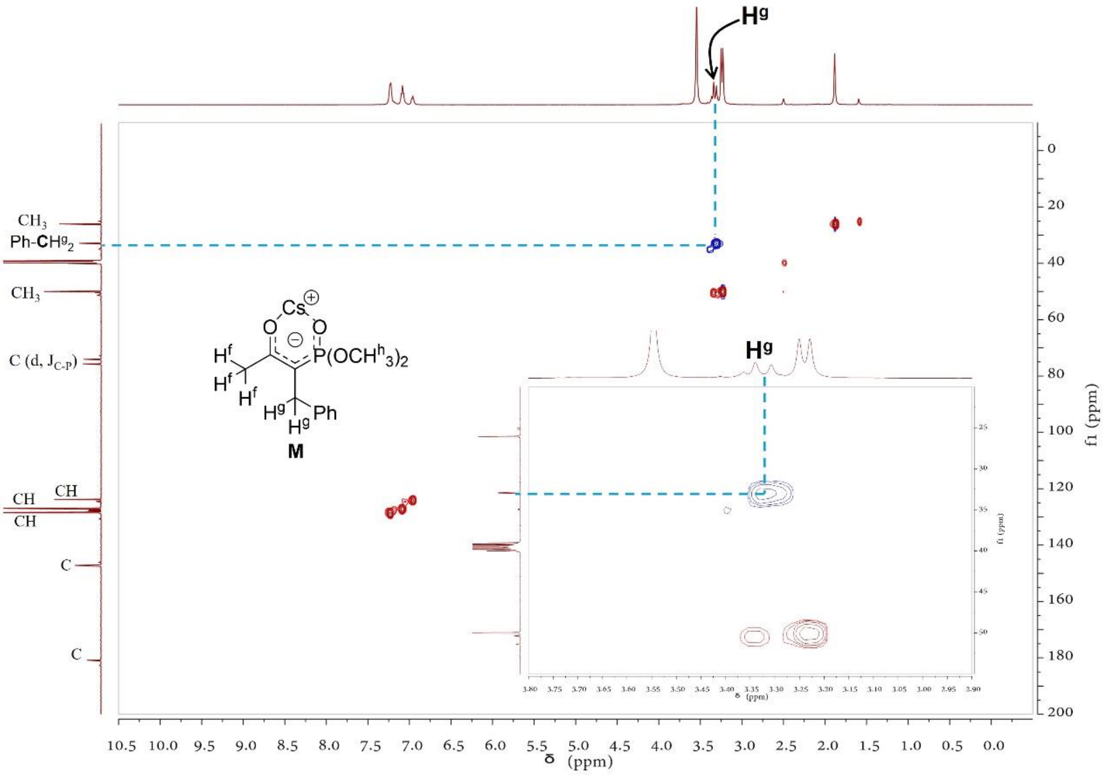

Figure S3. The HSQC spectrum of intermediate **M**.

3 Using NaHMDS or KHMDS as a base for the triazole synthesis.

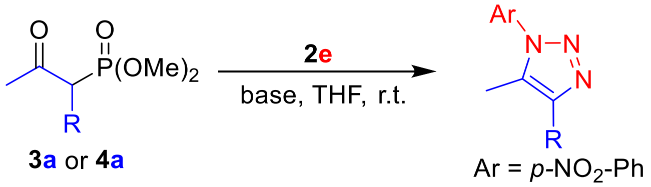

base	R= Bn **3ae**	R= H **5ae**
NaHMDS	44%	48%
KHMDS	13%	20%

Table S1. The results of NaHMDS or KHMDS as a base for the triazole synthesis.

4 Experimental procedures and characterization data

4.1 General procedure for the synthesis of compound 4

General procedure A:

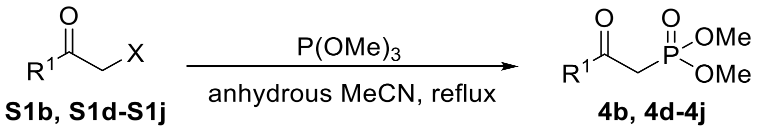

Compound **S1** (1.0 eq.) in anhydrous MeCN (0.1 M) was added by trimethyl phosphite (2.5 eq.). The resulting mixture was refluxed until the reaction was completed according to thin layer chromatography (TLC). The solution was then cooled to room temperature,

diluted with EtOAc (10 mL), and washed with brine (10 mL x 3) to remove excess trimethyl phosphite. The combined aqueous solution was extracted with EtOAc (10 mL x 3). The organic layers were all combined, dried over anhydrous $MgSO_4$, filtered, and concentrated under reduced pressure to give the crude residue. The residue was purified by flash column chromatography on silica gel to give compound **4**.

General procedure B:

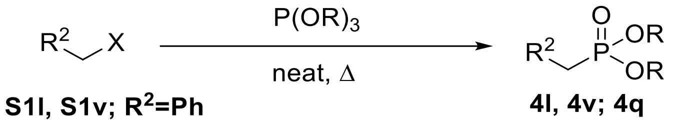

The mixture of compound **S1** (1.0 eq.) and trialkyl phosphite (2.0 eq.) was heated at an indicated temperature (120-170 °C, oil bath temperature) in a round-bottom flask with a Dean-Stark trap as a volatile by-product receiver. When the reaction was completed, the undesired by-products and excess starting material were distilled off to yield the desired phosphonate **4**, which could be used directly for the next step without further purification.

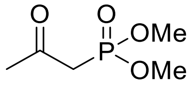

4a Compound **4a** was commercially available.

Dimethyl (2-oxoheptyl)phosphonate (4b)

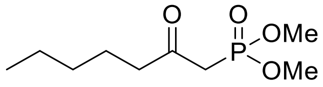

4b

According to the **General Procedure A**, 1-bromoheptan-2-one **S1b** (135.6 mg, 0.700 mmol, 1.0 eq.) and trimethyl phosphite (207 μl, 1.755 mmol, 2.5 eq.) were carried out in anhydrous MeCN (0.1 M) solution. The obtained residue was purified by flash column chromatography on silica gel, eluting with 100% EtOAc (R_f = 0.60) to give **4b** as light-yellow liquid (109.6 mg, 0.493 mmol, 70% yield).

^{1}H NMR (400 MHz, $CDCl_3$) δ = 3.77 (d, J = 11.2 Hz, 6H), 3.07 (d, J = 22.7 Hz, 2H), 2.59 (t, J = 7.4 Hz, 2H) 1.57 (quint., J = 7.4 Hz, 2H), 1.32-1.23 (m, 4H), 0.87 (t, J = 6.9 Hz, 3H). All spectroscopic data were in agreement with the literature values.[1]

Dimethyl (3,3-dimethyl-2-oxobutyl)phosphonate (4c)

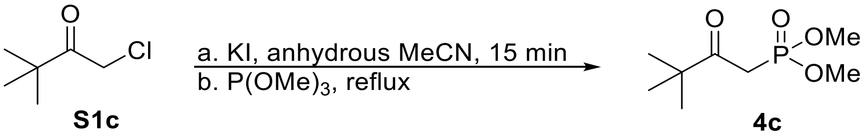

1-Chloro-3,3-dimethylbutan-2-one **S1c** (292 µl, 2.229 mmol 1.0 eq.) was dissolved in anhydrous MeCN (0.1 M), followed by adding potassium iodide (407 mg, 2.452 mmol, 1.1 eq.) slowly. After the solution was stirred for 15 min, trimethyl phosphite (657 µl, 5.572 mmol, 2.5 eq.) was injected into the solution. The mixture was refluxed until the reaction was completed according to thin layer chromatography (TLC). The solution was then cooled to room temperature, diluted with EtOAc (10 mL), extracted with brine (10 mL × 3) to remove excess $P(OMe)_3$. The organic layers were dried over $MgSO_4$, filtered, and concentrated under reduced pressure to give the crude residue. The residue was purified by flash column chromatography on silica gel, eluting with 100% EtOAc (R_f = 0.53) to give **4c** as colorless oil (175 mg, 0.841 mmol, 37% yield).
^1H NMR (400 MHz, $CDCl_3$, ppm) δ = 3.79 (d, *J* = 11.2 Hz, 6H), 3.17 (d, *J* = 21.6 Hz, 2H), 1.17 (s, 9H). All spectroscopic data were in agreement with the literature values.[2]

Dimethyl (2-oxo-2-phenylethyl) phosphonate (4d)

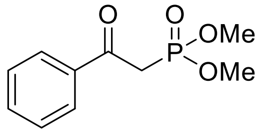

4d

According to the **General Procedure A**, 2-bromo-1-phenylethan-1-one **S1d** (150 mg, 0.754 mmol 1.0 eq.) and trimethyl phosphite (222 µl, 1.884 mmol, 2.5 eq.) were carried out in anhydrous MeCN (0.1 M) solution. The crude residue was purified by flash column chromatography on silica gel, eluting with 100% EtOAc (R_f = 0.45) to give **4d** as light-yellow oil (129.2 mg, 0.567 mmol, 75% yield).
^1H NMR (400 MHz, $CDCl_3$, ppm) δ = 8.04-7.97 (m, 2H), 7.61 (t, *J* = 7.4 Hz, 1H), 7.49 (t, *J* = 7.6 Hz, 2H), 3.79 (d, *J* = 11.2 Hz, 6H), 3.65 (d, *J* = 22.6 Hz, 2H). All spectroscopic data were in agreement with the literature values.[3]

Dimethyl (2-(4-methoxyphenyl)-2-oxoethyl)phosphonate (4e)

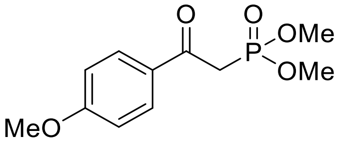

4e

According to the **General Procedure A**, 2-bromo-1-(4-methoxyphenyl)ethan-1-one **S1e** (150 mg, 0.655 mmol 1.0 eq.) and trimethyl phosphite (193 µl, 1.638 mmol, 2.5 eq.) were carried out in anhydrous MeCN (0.1 M) solution. The crude residue was purified by flash column chromatography on silica gel, eluting with 100% EtOAc (R_f = 0.33) to give **4e** as colorless oil (158.8 mg, 0.615 mmol, 94% yield).
^1H NMR (400 MHz, $CDCl_3$, ppm) δ = 8.01-7.97 (m, 2H), 6.97-6.94 (m, 2H), 3.88 (s,

3H), 3.78 (d, J = 11.2 Hz, 6H), 3.59 (d, J = 22.6 Hz, 2H). All spectroscopic data were in agreement with the literature values.[3]

Dimethyl (2-(4-fluorophenyl)-2-oxoethyl)phosphonate (4f)

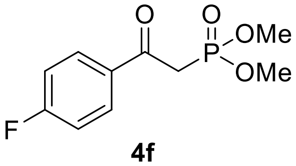

4f

According to the **General Procedure A**, 2-bromo-1-(4-fluorophenyl)ethan-1-one **S1f** (150 mg, 0.695 mmol 1.0 eq.) and trimethyl phosphite (205 µl, 1.737 mmol, 2.5 eq.) were carried out in anhydrous MeCN (0.1 M) solution. The crude residue was purified by flash column chromatography on silica gel, eluting with 100% EtOAc (R_f = 0.45) to give **4f** as yellow oil (132.8 mg, 0.540 mmol, 78% yield).

^{1}H NMR (400 MHz, $CDCl_3$, ppm) δ = 8.07-8.03 (m, 2H), 7.18-7.14 (m, 2H), 3.79 (d, J = 11.2 Hz, 6H), 3.62 (d, J = 22.7 Hz, 2H). All spectroscopic data were in agreement with the literature values.[3]

Dimethyl (2-(4-chlorophenyl)-2-oxoethyl) phosphonate (4g)

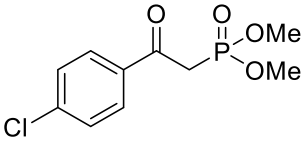

4g

According to the **General Procedure A**, 2-bromo-1-(4-chlorophenyl)ethan-1-one **S1g** (150 mg, 0.647 mmol 1.0 eq.) and trimethyl phosphite (191 µl, 1.618 mmol, 2.5 eq.) were carried out in anhydrous MeCN (0.1 M) solution. The crude residue was purified by flash column chromatography on silica gel, eluting with 100% EtOAc (R_f = 0.45) to give **4g** as yellow oil (106.2 mg, 0.405 mmol, 63% yield).

^{1}H NMR (400 MHz, $CDCl_3$, ppm) δ = 7.98-7.94 (m, 2H), 7.49-7.45 (m, 2H), 3.79 (d, J = 11.2 Hz, 6H), 3.61(d, J = 22.8 Hz, 2H). All spectroscopic data were in agreement with the literature values.[3]

Dimethyl (2-(4-bromophenyl)-2-oxoethyl)phosphonate (4h)

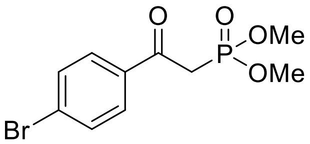

4h

According to the **General Procedure A**, 2-bromo-1-(4-bromophenyl)ethan-1-one **S1h** (150 mg, 0.544 mmol 1.0 eq.) and trimethyl phosphite (160 µl, 1.360 mmol, 2.5 eq.)

were carried out in anhydrous MeCN (0.1 M) solution. The crude residue was purified by flash column chromatography on silica gel, eluting with 100% EtOAc (R_f = 0.50) to give **4h** as yellow oil (109.4 mg, 0.358 mmol, 66% yield).

^{1}H NMR (400 MHz, $CDCl_3$, ppm) δ = 7.89-7.86 (m, 2H), 7.65-7.62 (m, 2H), 3.79 (d, *J* = 11.2 Hz, 6H), 3.61 (d, *J* = 22.8 Hz, 2H). All spectroscopic data were in agreement with the literature values.[3]

Dimethyl (2-(4-nitrophenyl)-2-oxoethyl)phosphonate (4i)

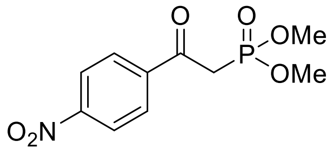

4i

According to the **General Procedure A**, 2-bromo-1-(4-nitrophenyl)ethan-1-one **S1i** (300 mg, 1.235 mmol 1.0 eq.) and trimethyl phosphite (273 μl, 3.087 mmol, 2.5 eq.) were carried out in anhydrous MeCN (0.1 M) solution. The crude residue was purified by flash column chromatography on silica gel, eluting with 100% EtOAc (R_f = 0.50) to give **4i** as yellow oil (74.9 mg, 0.274 mmol, 22% yield).

^{1}H NMR (400 MHz, $CDCl_3$, ppm) δ = 8.35-8.32 (m, 2H), 8.21-8.17 (m, 2H), 3.80 (d, *J* = 11.3 Hz, 6H), 3.68 (d, *J* = 22.9 Hz, 2H). All spectroscopic data were in agreement with the literature values.[3]

Dimethyl (2-(3-nitrophenyl)-2-oxoethyl) phosphonate (4j)

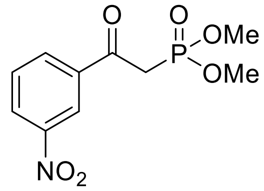

4j

According to the **General Procedure A**, 2-bromo-1-(3-nitrophenyl)ethan-1-one **S1j** (300 mg, 1.235 mmol 1.0 eq.) and trimethyl phosphite (364 μl, 3.087 mmol, 2.5 eq.) were carried out in anhydrous MeCN (0.1 M) solution. The crude residue was purified by flash column chromatography on silica gel, eluting with 100% EtOAc (R_f = 0.38) to give **4j** as yellow oil (107.5 mg, 0.394 mmol, 32% yield).

^{1}H NMR (400 MHz, $CDCl_3$, ppm) δ = 8.85 (t, *J* = 2.0 Hz, 1H), 8.46 (ddd, *J* = 8.2, 2.2, 1.0 Hz, 1H), 8.36 (dt, *J* = 7.8, 1.2 Hz, 1H), 7.72 (t, *J* = 8.0 Hz, 1H), 3.81 (d, *J* = 11.3 Hz, 6H), 3.70 (d, *J* = 22.9 Hz, 2H). All spectroscopic data were in agreement with the literature values.[4]

Dimethyl hexylphosphonate (4l)

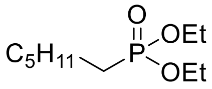

4l

According to the **General Procedure B**, 1-bromohexane **S1l** (2 ml, 14.360 mmol, 1.0 eq.) and triethylphosphite (4.96 ml, 28.720 mmol, 2.0 eq.) were carried out at 170 °C (oil bath temperature). The resulting residue was distilled to remove by-products at 120 °C under 650 mbar and yield the desired product **4l** (100% EtOAc, R_f = 0.50) as colorless liquid (3160.6 mg, 14.22 mmol, 99% yield).

^{1}H NMR (400 MHz, $CDCl_3$, ppm) δ = 4.16-4.02 (m, 4H), 1.75-1.66 (m, 2H), 1.64-1.54 (m, 2H), 1.41-1.25 (m, 6H), 1.32 (t, *J* = 7.1 Hz, 6H), 0.89 (t, *J* = 7.3 Hz, 3H). All spectroscopic data were in agreement with the literature values.[5]

Dimethyl benzylphosphonate (4q)

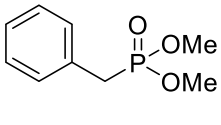

4q

According to the **General Procedure B**, benzylbromide (2 ml, 15.827 mmol, 1.0 eq.) and trimethylphosphite (3.73 ml, 31.654 mmol, 2.0 eq.) were carried out at 120 °C (oil bath temperature). The resulting residue was distilled to remove by-products at 110 °C under 650 mbar and yield the desired product **4q** (100% EtOAc, R_f = 0.45) as colorless liquid colorless liquid (3125.5 mg, 15.614 mmol, 99% yield).

^{1}H NMR (500 MHz, $CDCl_3$, ppm) δ = 7.34-7.24 (m, 5H), 3.67 (d, *J* = 10.8 Hz, 6H), 3.17 (d, *J* = 21.7 Hz, 2H). All spectroscopic data were in agreement with the literature values.[6]

Diethyl phenethylphosphonate (4v)

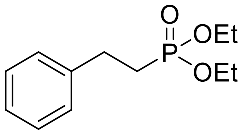

4v

According to the **General Procedure B**, (2-bromoethyl)benzene **S1v** (2 ml, 14.628 mmol, 1.0 eq.) and triethylphosphite (5.02 ml, 29.256 mmol, 2.0 eq.) were carried out at 170 °C (oil bath temperature). The resulting residue was distilled to remove by-products at 120 °C under 650 mbar and yield the desired product **4v** (40% v/v EtOAc

in hexane, $R_f = 0.45$) as colorless liquid colorless liquid (3508.3 mg, 14.482 mmol, 99% yield).

^1H NMR (400 MHz, $CDCl_3$, ppm) δ = 7.32-7.20 (m, 5H), 4.14-4.06 (m, 4H), 2.95-2.89 (m, 2H), 2.10-2.02 (m, 2H), 1.32 (t, J = 7.1 Hz, 6H). All spectroscopic data were in agreement with the literature values.[7]

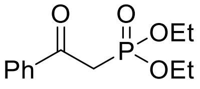

4z

Compound 4z was commercially available.

4.2 General procedure for the synthesis of compound 1

General Procedure C:

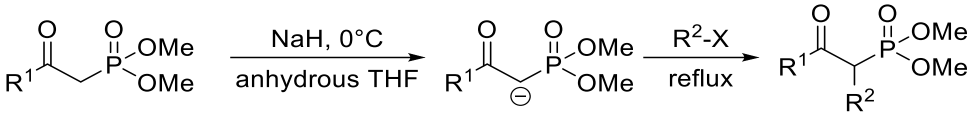

4a-4j **1a-1k, 1m-1p, 1r**

The diluted compound **4** (1.0 eq., 1.0 M in anhydrous THF) was slowly injected into the solution of sodium hydride (1.1 eq. 0.1 M) in anhydrous THF at 0 °C. The resulting mixture was stirred for another 30 min before alkylhalide (1.1 eq.) was added. The mixture was refluxed until the reaction was completed according to thin layer chromatography (TLC). The solution was cooled to room temperature, quenched by NH_4Cl (sat.), and diluted with EtOAc (10 mL). The resulting mixture was washed with brine (10 mL) and the aqueous layer was separated for further extraction with EtOAc (10 mL × 3). The organic layers were all combined, dried over anhydrous $MgSO_4$, filtered, and concentrated under reduced pressure to give the crude residue. The residue was purified by flash column chromatography on silica gel to give compound **1**.

General Procedure D:

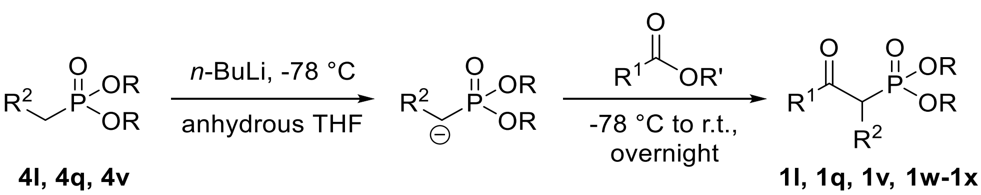

4l, 4q, 4v **1l, 1q, 1v, 1w-1x**

2.5 M *n*-BuLi in hexane (1.1 eq.) was slowly added into the solution of compound **4** (1.0 eq., 1.0 M) in anhydrous THF at -78 °C and the resulting mixture was kept stirring for another 1 hour. After acylation reagent (1.5 eq.) was added, the solution was warmed up to room temperature for reacting overnight. After the reaction was completed, the

solution was quenched by NH_4Cl (sat.) and diluted with EtOAc (10 mL). The resulting mixture was extracted with brine (10 mL x 2) and the aqueous layers were collected for further back extraction with EtOAc (10 mL × 3). The organic layers were all combined, dried over anhydrous $MgSO_4$, filtered, and concentrated under reduced pressure to give the crude residue. The residue was purified by flash column chromatography on silica gel to give compound **1**.

General Procedure E:

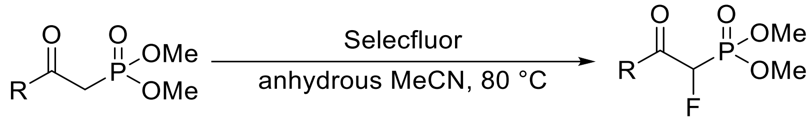

4a, 4z **1y, 1z**

Compound **4** (1.0 eq., 0.05 M) in anhydrous acetonitrile was added by Selectfluor (2.0 eq.) and continuously stirred for 10 min at room temperature. The reaction was then heated to reflux for another 24 h. After the reaction was completed, the mixture was cooled to room temperature, quenched by NH_4Cl (sat., 20 mL), and extracted with Et_2O (10 mL x 2). The organic layers were all combined, washed with brine (15 mL), dried over anhydrous $MgSO_4$, filtered, and concentrated under reduced pressure to give the crude residue which was purified by flash column chromatography on silica gel to give compound **1**.

Dimethyl (3-oxo-1-phenylbutan-2-yl)phosphonate (1a)

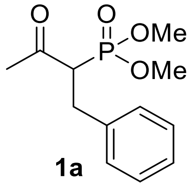

1a

According to the **General Procedure C**, compound **4a** (272.3 mg, 1.674 mmol, 1.0 eq.), 60% sodium hydride (72.1 mg, 1.803 mmol, 1.1 eq.), and benzylbromide (315.0 mg, 1.841 mmol, 1.1 eq.) were carried out in anhydrous THF (0.1 M). The residue was purified by flash column chromatography on silica gel, eluting with 100% EtOAc (R_f = 0.50) to give **1a** as colorless oil (202.9 mg, 0.7918 mmol, 47% yield).

^{1}H NMR (400 MHz, $CDCl_3$, ppm) δ = 7.29-7.14 (m, 5H), 3.80 (d, J = 11.0 Hz, 3H), 3.80 (d, J = 10.9 Hz, 3H), 3.55 (ddd, J = 23.9, 11.1, 3.4 Hz, 1H), 3.31 (ddd, J = 14.1, 11.2, 7.8 Hz, 1H), 3.10 (ddd, J = 14.1, 10.7, 3.4 Hz, 1H), 2.16 (s, 3H); ^{13}C{^{1}H} NMR (100 MHz, $CDCl_3$, ppm) δ = 203.1 (d, J_{C-P} = 4.4 Hz), 138.8 (d, J_{C-P} = 15.9 Hz), 128.7, 128.6, 126.8, 54.6 (d, J_{C-P} = 123.0 Hz), 53.6 (d, J_{C-P} = 6.7 Hz), 53.3 (d, J_{C-P} = 6.8 Hz), 32.3 (d, J_{C-P} = 4.8 Hz), 32.1; ^{31}P NMR (202 MHz, $CDCl_3$, ppm) δ = 23.6; IR (KBr, cm$^-$

[1]) 2957, 2854, 1715, 1252, 1030; HRMS (ESI$^+$) m/z calculated for $(C_{12}H_{17}O_4P+H)^+$: 257.0937, found: 257.0936.

Dimethyl (3-oxo-1-phenyloctan-2-yl)phosphonate (1b)

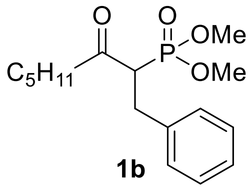

1b

According to the **General Procedure C**, compound **4b** (109.6 mg, 0.494 mmol, 1.0 eq.), 60% sodium hydride (21.7 mg, 0.543 mmol, 1.1 eq.), and benzylbromide (92.9 mg, 0.543 mmol, 1.1 eq.) were carried out in anhydrous THF (0.1 M). The residue was purified by flash column chromatography on silica gel, eluting with 80% v/v EtOAc in hexane (R_f = 0.60) to give **1b** as colorless oil (83.2 mg, 0.267 mmol, 54% yield).
^{1}H NMR (400 MHz, $CDCl_3$, ppm) δ = 7.27-7.13 (m, 5H), 3.80 (d, J = 11.0 Hz, 3H), 3.79 (d, J = 10.9 Hz, 3H), 3.53 (ddd, J = 23.4, 11.3, 3.3 Hz, 1H), 3.30 (ddd, J = 13.8, 11.4, 7.4 Hz, 1H), 3.08 (ddd, J = 13.8, 10.5, 3.2 Hz, 1H), 2.58 (dt, J = 17.5, 7.2 Hz, 1H), 2.17 (dt, J = 17.4, 7.2 Hz, 1H), 1.41-1.32 (m, 2H), 1.21-1.12 (m, 2H), 1.11-1.01 (m, 2H), 0.80 (t, J = 7.2 Hz, 3H); $^{13}C\{^1H\}$ NMR (100 MHz, $CDCl_3$, ppm) δ = 205.3 (d, J_{C-P} = 4.1 Hz), 138.8 (d, J_{C-P} = 16.1 Hz), 128.6, 126.7, 53.9 (d, J_{C-P} = 123.4 Hz), 53.5 (d, J_{C-P} = 6.4 Hz), 53.1 (d, J_{C-P} = 6.6 Hz), 45.0, 32.5 (d, J_{C-P} = 4.6 Hz), 30.8, 22.7, 22.3, 13.8; ^{31}P NMR (202 MHz, $CDCl_3$, ppm) δ = 23.8; IR (KBr, cm^{-1}) 2956, 2857, 1713, 1252, 1031; HRMS (ESI$^+$) m/z calculated for $(C_{16}H_{25}O_4P+H)^+$: 313.1563, found: 313.1559.

Dimethyl (4,4-dimethyl-3-oxo-1-phenylpentan-2-yl)phosphonate (1c)

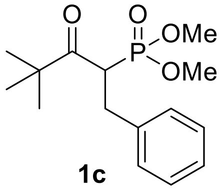

1c

According to the **General Procedure C**, compound **4c** (60.0 mg, 0.288 mmol, 1.0 eq.), 60% sodium hydride (21.2 mg, 0.318 mmol, 1.1 eq.), and benzylbromide (54.2 mg, 0.317 mmol, 1.1 eq.) were carried out in anhydrous THF (0.1 M). The residue was purified by flash column chromatography on silica gel, eluting with 100% EtOAc (R_f = 0.63) to give **1c** as colorless oil (51.2 mg, 0.172 mmol, 60% yield).
^{1}H NMR (400 MHz, $CDCl_3$, ppm) δ = 7.27-7.17 (m, 5H), 3.91 (ddd, J = 20.4, 10.7, 4.1 Hz, 1H), 3.79 (d, J = 10.9 Hz, 6H), 3.26-3.11 (m, 2H), 0.83 (s, 9H); $^{13}C\{^1H\}$ NMR (100

MHz, $CDCl_3$, ppm) δ = 211.0 (d, $J_{C\text{-}P}$ = 5.3 Hz), 138.7 (d, $J_{C\text{-}P}$ = 16.3 Hz), 129.1, 128.4, 126.7, 53.4 (d, $J_{C\text{-}P}$ = 6.6 Hz), 52.9 (d, $J_{C\text{-}P}$ = 6.9 Hz), 48.6 (d, $J_{C\text{-}P}$ = 125.8 Hz), 45.1, 34.9 (d, $J_{C\text{-}P}$ = 5.3 Hz); ^{31}P NMR (202 MHz, $CDCl_3$, ppm) δ = 22.4; IR (KBr, cm^{-1}) 2958, 1703, 1455, 1252, 1031; HRMS (ESI^+) m/z calculated for $(C_{15}H_{23}O_4P+H)^+$: 299.1407, found 299.1404.

Dimethyl (1-oxo-1,3-diphenylpropan-2-yl)phosphonate (1d)

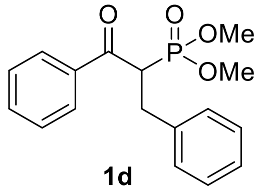

According to the **General Procedure C**, compound **4d** (200.0 mg, 0.877 mmol, 1.0 eq.), 60% sodium hydride (38.6 mg, 0.965 mmol, 1.1 eq.), and benzylbromide (165.0 mg, 0.965 mmol, 1.1 eq.) were carried out in anhydrous THF (0.1 M). The residue was purified by flash column chromatography on silica gel, eluting with 100% EtOAc (R_f = 0.65) to give **1d** as white solid (181.5 mg, 0.571 mmol, 64% yield).
Melting point = 116.1-116.6 °C; ^{1}H NMR (400 MHz, $CDCl_3$, ppm) δ = 7.80 (dd, *J* = 8.3, 1.4 Hz, 2H), 7.51 (tt, *J* = 7.4, 1.1 Hz, 1H), 7.38 (dd, *J* = 8.0, 7.7 Hz, 2H), 7.21-7.10 (m, 5H), 4.44 (ddd, *J* = 22.8, 11.0, 3.4 Hz, 1H), 3.78 (d, *J* = 10.9 Hz, 3H), 3.70 (d, *J* = 11.0 Hz, 3H), 3.54 (ddd, *J* = 13.9, 11.0, 8.0 Hz, 1H), 3.30 (ddd, *J* = 13.9, 10.6, 3.4 Hz, 1H); ^{13}C{^{1}H} NMR (100 MHz, $CDCl_3$, ppm) δ = 195.7 (d, $J_{C\text{-}P}$ = 5.2 Hz), 138.9 (d, $J_{C\text{-}P}$ = 15.7 Hz), 137.6, 133.4, 128.7, 128.6, 126.7, 53.5 (d, $J_{C\text{-}P}$ = 5.6 Hz), 53.5 (d, $J_{C\text{-}P}$ = 4.6 Hz), 49.0 (d, $J_{C\text{-}P}$ = 125.5 Hz), 33.5 (d, $J_{C\text{-}P}$ = 4.4 Hz); ^{31}P NMR (202 MHz, $CDCl_3$, ppm) δ = 23.8; IR (KBr, cm^{-1}) 2955, 1679, 1447, 1248, 1030; HRMS (ESI^+) m/z calculated for $(C_{17}H_{19}O_4P+H)^+$: 319.1094, found: 319.1088.

Dimethyl (1-(4-methoxyphenyl)-1-oxo-3-phenylpropan-2-yl)phosphonate (1e)

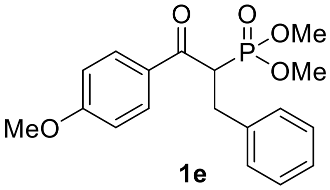

According to the **General Procedure C**, compound **4e** (74.0 mg, 0.287 mmol, 1.0 eq.), 60% sodium hydride (21.0 mg, 0.315 mmol, 1.1 eq.), and benzylbromide (58.8 mg, 0.344 mmol, 1.1 eq.) were carried out in anhydrous THF (0.1 M). The residue was purified by flash column chromatography on silica gel, eluting with 100% EtOAc (R_f = 0.63) to give **1e** as yellow oil (78.8 mg, 0.226 mmol, 79% yield).

^{1}H NMR (400 MHz, $CDCl_3$, ppm) δ = 7.84-7.80 (m, 2H), 7.20-7.09 (m, 5H), 6.87-6.84 (m, 2H), 4.38 (ddd, *J* = 22.7, 11.0, 3.4 Hz, 1H), 3.83 (s, 3H), 3.77 (d, *J* = 10.9 Hz, 3H), 3.71 (d, *J* = 10.9 Hz, 3H), 3.54 (ddd, *J* = 13.9, 11.0, 8.0 Hz, 1H), 3.28 (ddd, *J* = 14.0, 10.8, 3.3 Hz, 1H); ^{13}C{^{1}H} NMR (100 MHz, $CDCl_3$, ppm) δ = 193.7 (d, $J_{C\text{-}P}$ = 4.8 Hz), 163.8, 139.0 (d, $J_{C\text{-}P}$ = 15.9 Hz), 131.1, 130.5, 128.7, 128.5, 126.6, 113.8, 55.5, 53.5 (d, $J_{C\text{-}P}$ = 6.4 Hz), 53.4 (d, $J_{C\text{-}P}$ = 6.4 Hz), 48.4 (d, $J_{C\text{-}P}$ = 125.8 Hz), 33.4 (d, $J_{C\text{-}P}$ = 4.3 Hz); ^{31}P NMR (202 MHz, $CDCl_3$, ppm) δ = 24.3; IR (KBr, cm^{-1}) 2956, 2852, 1667, 1600, 1318, 1243, 1176, 1028; HRMS (ESI^+) m/z calculated for $(C_{18}H_{21}O_5P+H)^+$: 349.1199, found: 349.1196.

Dimethyl (1-(4-fluorophenyl)-1-oxo-3-phenylpropan-2-yl)phosphonate (1f)

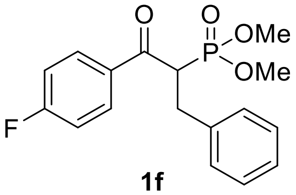

1f

According to the **General Procedure C**, compound **4f** (155.5 mg, 0.6317 mmol, 1.0 eq.), 60% sodium hydride (27.8 mg, 0.695 mmol, 1.1 eq.), and benzylbromide (118.9 mg, 0.695 mmol, 1.1 eq.) were carried out in anhydrous THF (0.1 M). The residue was purified by flash column chromatography on silica gel, eluting with 100% EtOAc (R_f = 0.73) to give **1f** as yellow oil (138.4 mg, 0.412 mmol, 65% yield).

^{1}H NMR (400 MHz, $CDCl_3$, ppm) δ = 7.86-7.81 (m, 2H), 7.21-7.10 (m, 5H), 7.07-7.02 (m, 2H), 4.37 (ddd, *J* = 22.8, 11.0, 3.4 Hz, 1H), 3.79 (d, *J* = 11.0 Hz, 3H), 3.73 (d, *J* = 10.9 Hz, 3H), 3.52 (ddd, *J* = 13.9, 11.0, 7.9 Hz, 1H), 3.29 (ddd, *J* = 13.9, 10.5, 3.3 Hz, 1H); ^{13}C{^{1}H} NMR (100 MHz, $CDCl_3$, ppm) δ = 194.0 (d, $J_{C\text{-}P}$ = 4.9 Hz), 165.9 (d, $J_{C\text{-}F}$ = 254.2 Hz), 138.7 (d, $J_{C\text{-}P}$ = 15.9 Hz), 134.0, 131.3 (d, $J_{C\text{-}F}$ = 9.4 Hz), 128.6, 126.7, 115.7 (d, $J_{C\text{-}F}$ = 21.8 Hz), 53.6 (d, $J_{C\text{-}P}$ = 6.7 Hz), 53.4 (d, $J_{C\text{-}P}$ = 6.7 Hz), 49.1 (d, $J_{C\text{-}P}$ = 125.8 Hz), 33.4 (d, $J_{C\text{-}P}$ = 4.4 Hz); ^{31}P NMR (202 MHz, $CDCl_3$, ppm) δ = 23.5; ^{19}F NMR (658 MHz, $CDCl_3$, ppm) δ = -105.5; IR (KBr, cm^{-1}) 2956, 1679, 1597, 1236, 1159, 1030; HRMS (ESI^+) m/z calculated for $(C_{17}H_{18}FO_4P+H)^+$: 337.1000, found: 337.0996.

Dimethyl (1-(4-chlorophenyl)-1-oxo-3-phenylpropan-2-yl)phosphonate (1g)

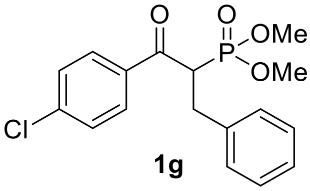

1g

According to the **General Procedure C**, compound **4g** (104.4 mg, 0.399 mmol, 1.0 eq.), 60% sodium hydride (29.3 mg, 0.439 mmol, 1.1 eq.), and benzylbromide (75.1 mg, 0.439 mmol, 1.1 eq.) were carried out in anhydrous THF (0.1 M). The residue was purified by flash column chromatography on silica gel, eluting with 80% v/v EtOAc in hexane (R_f = 0.55) to give **1g** as white solid (86.2 mg, 0.245 mmol, 61% yield).
Melting point = 99.8-100.3 °C; ^{1}H NMR (400 MHz, $CDCl_3$, ppm) δ = 7.78-7.72 (m, 2H), 7.37-7.33 (m, 2H), 7.22-7.10 (m, 5H), 4.36 (ddd, J = 22.8, 11.1, 3.3 Hz, 1H), 3.79 (d, J = 11.0 Hz, 3H), 3.72 (d, J = 11.0 Hz, 3H), 3.52 (ddd, J = 13.9, 11.1, 7.8 Hz, 1H), 3.29 (ddd, J = 13.9, 10.5, 3.3 Hz, 1H); $^{13}C\{^1H\}$ NMR (100 MHz, $CDCl_3$, ppm) δ = 194.6 (d, J_{C-P} = 4.8 Hz), 140.0, 138.7 (d, J_{C-P} = 15.9 Hz), 135.9, 130.0, 128.9, 128.7, 126.8, 53.7 (d, J_{C-P} = 7.1 Hz), 53.6 (d, J_{C-P} = 7.0 Hz), 49.2 (d, J_{C-P} = 125.7 Hz), 33.5 (d, J_{C-P} = 4.4 Hz); ^{31}P NMR (202 MHz, $CDCl_3$, ppm) δ = 23.4; IR (KBr, cm^{-1}) 2955, 1679, 1589, 1250, 1182, 1030; HRMS (ESI$^+$) m/z calculated for $(C_{17}H_{18}ClO_4P+H)^+$: 353.0704, found: 353.0703.

Dimethyl (1-(4-bromophenyl)-1-oxo-3-phenylpropan-2-yl)phosphonate (1h)

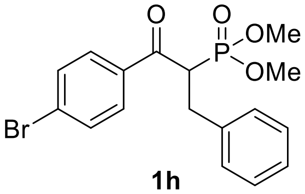

1h

According to the **General Procedure C**, compound **4h** (114.9 mg, 0.376 mmol, 1.0 eq.), 60% sodium hydride (27.5 mg, 0.413 mmol, 1.1 eq.), and benzylbromide (70.6 mg, 0.413 mmol, 1.1 eq.) were carried out in anhydrous THF (0.1 M). The residue was purified by flash column chromatography on silica gel, eluting with 80% v/v EtOAc (R_f = 0.68) in hexane to give **1h** as white solid (88.9 mg, 0.224 mmol, 60% yield).
Melting point = 95.9-96.4 °C; ^{1}H NMR (400 MHz, $CDCl_3$, ppm) δ = 7.68-7.64 (m, 2H), 7.54-7.50 (m, 2H), 7.22-7.10 (m, 5H), 4.35 (ddd, J = 22.8, 11.1, 3.2 Hz, 1H), 3.79 (d, J = 11.0 Hz, 3H), 3.72 (d, J = 11.0 Hz, 3H), 3.52 (ddd, J = 13.9, 11.1, 7.6 Hz, 1H), 3.29 (ddd, J = 13.9, 10.5, 3.3 Hz, 1H); $^{13}C\{^1H\}$ NMR (100 MHz, $CDCl_3$, ppm) δ = 194.8 (d, J = 5.1 Hz), 138.7 (d, J = 15.7 Hz), 136.3, 131.9, 130.1, 128.8, 128.7, 126.8, 53.7 (d, J_{C-P} = 7.5 Hz), 53.6 (d, J_{C-P} = 7.6 Hz), 49.2 (d, J_{C-P} = 125.6 Hz), 33.4 (d, J_{C-P} = 4.4 Hz); ^{31}P NMR (202 MHz, $CDCl_3$, ppm) δ = 23.3; IR (KBr, cm^{-1}) 2954, 2852, 1680, 1585, 1251, 1030; HRMS (ESI$^+$) m/z calculated for $(C_{17}H_{18}BrO_4P+H)^+$: 397.0199, found: 397.0200.

Dimethyl (1-(4-nitrophenyl)-1-oxo-3-phenylpropan-2-yl)phosphonate (1i)

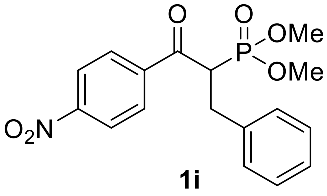

1i

According to the **General Procedure C**, compound **4i** (74.9 mg, 0.274 mmol, 1.0 eq.), 60% sodium hydride (20.2 mg, 0.301 mmol, 1.1 eq.), and benzylbromide (51.5 mg, 0.301 mmol, 1.1 eq.) were carried out in anhydrous THF (0.1 M). The residue was purified by flash column chromatography on silica gel, eluting with 80% v/v EtOAc in hexane (R_f = 0.63) to give **1i** as yellow oil (82.7 mg, 0.228 mmol, 62% yield).

^{1}H NMR (400 MHz, $CDCl_3$, ppm) δ = 8.23-8.19 (m, 2H), 7.93-7.88 (m, 2H), 7.23-7.11 (m, 5H), 4.40 (ddd, *J* = 23.0, 11.2, 3.3 Hz, 1H), 3.82 (d, *J* = 11.0 Hz, 3H), 3.75 (d, *J* = 11.0 Hz, 3H), 3.52 (ddd, *J* = 13.9, 11.2, 7.5 Hz, 1H), 3.31 (ddd, *J* = 13.8, 10.2, 3.3 Hz, 1H); ^{13}C{^{1}H} NMR (100 MHz, $CDCl_3$, ppm) δ = 194.9 (d, J_{C-P} = 5.2 Hz), 150.3, 142.1, 138.4 (d, J_{C-P} = 15.9 Hz), 129.5, 128.8, 128.6, 127.0, 123.8, 53.8 (d, J_{C-P} = 6.8 Hz), 53.6 (d, J_{C-P} = 6.6 Hz), 50.2 (d, J_{C-P} = 125.7 Hz), 33.5 (d, J_{C-P} = 4.5 Hz); ^{31}P NMR (202 MHz, $CDCl_3$, ppm) δ = 22.4; IR (KBr, cm^{-1}) 2955, 2853, 1687, 1526, 1347, 1250, 1028; HRMS (ESI$^+$) m/z calculated for $(C_{17}H_{18}NO_6P+H)^+$: 364.0944, found: 364.0942.

Dimethyl (1-(3-nitrophenyl)-1-oxo-3-phenylpropan-2-yl)phosphonate (1j)

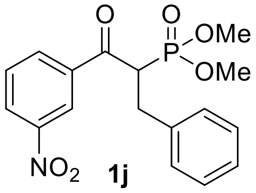

1j

According to the **General Procedure C**, compound **4j** (78.9 mg, 0.289 mmol, 1.0 eq.), 60% sodium hydride (21.2 mg, 0.318 mmol, 1.1 eq.), and benzylbromide (54.4 mg, 0.318 mmol, 1.1 eq.) were carried out in anhydrous THF (0.1 M). The residue was purified by flash column chromatography on silica gel, eluting with 80% v/v EtOAc in hexane (R_f = 0.48) to give **1j** as yellow oil (35.3 mg, 0.097 mmol, 34% yield).

^{1}H NMR (400 MHz, $CDCl_3$, ppm) δ = 8.6 (t, *J* = 2.0 Hz, 1H), 8.35 (m, 1H), 8.11 (d, *J* = 7.8 Hz, 1H), 7.59 (t, *J* = 8.0 Hz, 1H), 7.23-7.11 (m, 5H), 4.41 (ddd, *J* = 22.9, 11.2, 3.4 Hz, 1H), 3.82 (d, *J* = 11.0 Hz, 3H), 3.77 (d, *J* = 11.0 Hz, 3H), 3.53 (ddd, *J* = 13.9, 11.2, 7.5 Hz, 1H), 3.33 (ddd, *J* = 13.7, 10.1, 3.3 Hz, 1H); ^{13}C{^{1}H} NMR (100 MHz, $CDCl_3$, ppm) δ = 193.9 (d, J_{C-P} = 5.0 Hz), 148.4, 138.7, 138.4 (d, J_{C-P} = 15.9 Hz), 134.1, 129.9, 128.8, 128.7, 127.6, 127.0, 123.5, 53.9 (d, J_{C-P} = 6.5 Hz), 53.6 (d, J_{C-P} = 6.6 Hz), 49.8 (d, J_{C-P} = 125.8 Hz), 33.5 (d, J_{C-P} = 4.4 Hz); ^{31}P NMR (202 MHz, $CDCl_3$, ppm) δ

= 22.5; IR (KBr, cm^{-1}) 2957, 2854, 1688, 1514, 1351, 1250, 1031; HRMS (ESI^{+}) m/z calculated for $(C_{17}H_{18}NO_6P+H)^+$: 364.0944, found: 364.0942.

Dimethyl (3-oxobutan-2-yl)phosphonate (1k)

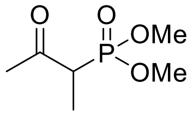

1k

According to the **General Procedure C**, compound **4a** (295.6 mg, 1.780 mmol, 1.0 eq.), 60% sodium hydride (79.5 mg, 1.988 mmol, 1.1 eq.), and methyl iodide (277.9 mg, 1.958 mmol, 1.1 eq.) were carried out in anhydrous THF (0.1 M). The residue was purified by flash column chromatography on silica gel, eluting with 100% EtOAc (R_f = 0.40) to give **1k** as colorless oil (120.0 mg, 0.6665 mmol, 38% yield).

^{1}H NMR (400 MHz, $CDCl_3$, ppm) δ = 3.79 (d, J = 10.9 Hz, 3H), 3.78 (d, J = 10.9 Hz, 3H), 3.26 (dq, J = 25.5, 7.1 Hz, 1H), 2.34 (s, 3H), 1.37 (dd, J = 18.0, 7.1 Hz, 3H). All spectroscopic data were in agreement with the literature values.[8]

Dimethyl (2-oxooctan-3-yl)phosphonate (1l)

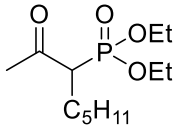

1l

According to the **General Procedure D**, compound **4l** (515.0 mg, 2.652 mmol, 1.0 eq.), 2.5 M *n*-BuLi (1.2 ml, 2.917 mmol, 1.1 eq.), and ethyl acetate (350.5 mg, 3.978 mmol, 1.5 eq.) were carried out in anhydrous THF (0.1 M). The residue was purified by flash column chromatography on silica gel, eluting with 100% EtOAc (R_f = 0.60) to give **1l** as colorless liquid (432.3 mg, 1.636 mmol, 62% yield).

^{1}H NMR (400 MHz, $CDCl_3$, ppm) δ = 4.10-3.97 (m, 4H), 3.05 (ddd, J = 24.3, 10.8, 3.6 Hz, 1H), 2.22 (s, 3H), 1.98-1.87 (m, 1H), 1.74-1.60 (m, 1H), 1.24 (t, J = 7.0 Hz, 6H), 1.21-1.17 (m, 6H), 0.79 (t, J = 6.6 Hz, 3H); $^{13}C\{^1H\}$ NMR (100 MHz, $CDCl_3$, ppm) δ = 203.8 (d, J_{C-P} = 4.4 Hz), 62.6 (d, J_{C-P} = 6.7 Hz), 62.5 (d, J_{C-P} = 6.8 Hz), 53.7 (d, J_{C-P} = 124.0 Hz), 31.4, 31.1, 28.2 (d, J_{C-P} = 14.8 Hz), 26.4 (d, J_{C-P} = 5.1 Hz), 22.3, 16.4 (d, J_{C-P} = 5.9 Hz), 16.3 (d, J_{C-P} = 5.9 Hz), 13.9. All spectroscopic data were in agreement with the literature values.[9]

Dimethyl (2-oxohex-5-en-3-yl)phosphonate (1m)

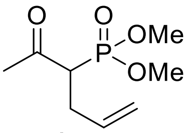

1m

According to the **General Procedure C**, compound **4a** (102.2 mg, 0.615 mmol, 1.0 eq.), 60% sodium hydride (27.0 mg, 0.677 mmol, 1.1 eq.), and 3-bromoprop-1-ene (81.8 mg, 0.677 mmol, 1.1 eq.) were carried out in anhydrous THF (0.1 M). The residue was purified by flash column chromatography on silica gel, eluting with 100% EtOAc (R_f = 0.50) to give **1m** as colorless oil (28.5 mg, 0.1380 mmol, 22% yield).

^{1}H NMR (400 MHz, $CDCl_3$, ppm) δ = 5.75-5.65 (m, 1H), 5.10-5.02 (m, 2H), 3.78 (d, *J* = 11.0 Hz, 6H), 3.27 (ddd, *J* = 24.1, 10.9, 3.8 Hz, 1H), 2.80-2.70 (m, 1H), 2.59-2.46 (m, 1H), 2.30 (s, 3H); ^{13}C{^{1}H} NMR (100 MHz, $CDCl_3$, ppm) δ = 203.0 (d, $J_{C\text{-}P}$ = 3.6 Hz), 134.4 (d, $J_{C\text{-}P}$ = 69.9 Hz), 117.4, 53.5 (d, $J_{C\text{-}P}$ = 6.7 Hz), 53.3 (d, $J_{C\text{-}P}$ = 6.8 Hz), 52.7 (d, $J_{C\text{-}P}$ = 124.4 Hz), 31.4, 30.5 (d, $J_{C\text{-}P}$ = 4.7 Hz); ^{31}P NMR (162 MHz, $CDCl_3$, ppm) δ = 24.2; IR (KBr, cm^{-1}) 2959, 1716, 1250, 1185, 1030; HRMS (ESI^+) m/z calculated for $(C_8H_{15}O_4P+H)^+$: 207.0781, found: 207.0774.

Dimethyl (2-oxohex-5-yn-3-yl)phosphonate (1n)

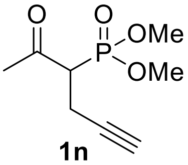

1n

According to the **General Procedure C**, compound **4a** (104.0 mg, 0.626 mmol, 1.0 eq.), 60% sodium hydride (27.5 mg, 0.6875 mmol, 1.1 eq.), and 3-bromoprop-1-yne (81.9 mg, 0.6885 mmol, 1.1 eq.) were carried out in anhydrous THF (0.1 M). The residue was purified by flash column chromatography on silica gel, eluting with 100% EtOAc (R_f = 0.45) to give **1n** as colorless oil (24.8 mg, 0.1215 mmol, 20% yield).

^{1}H NMR (400 MHz, $CDCl_3$, ppm) δ = 3.79 (d, *J* = 11.1 Hz, 3H), 3.78 (d, *J* = 11.0 Hz, 3H), 3.43 (ddd, *J* = 23.8, 10.4, 4.1 Hz, 1H), 2.93-2.83 (m, 1H), 2.67-2.58 (m, 1H), 2.39 (s, 3H), 2.00 (t, *J* = 2.6 Hz, 1H); ^{13}C{^{1}H} NMR (100 MHz, $CDCl_3$, ppm) δ = 201.6 (d, $J_{C\text{-}P}$ = 3.6 Hz), 80.8 (d, $J_{C\text{-}P}$ = 19.8 Hz), 70.0, 53.7 (d, $J_{C\text{-}P}$ = 6.6 Hz),53.4 (d, $J_{C\text{-}P}$ = 6.8 Hz), 52.0 (d, $J_{C\text{-}P}$ = 124.9 Hz), 31.4, 16.4 (d, $J_{C\text{-}P}$ = 3.7 Hz); ^{31}P NMR (162 MHz, $CDCl_3$, ppm) δ = 22.3; IR (KBr, cm^{-1}) 2960, 1718, 1361, 1250, 1184, 1031; HRMS (ESI^+) m/z calculated for $(C_8H_{13}O_4P+H)^+$: 205.0624, found: 205.0616.

Ethyl 3-(dimethoxyphosphoryl)-4-oxopentanoate (1o)

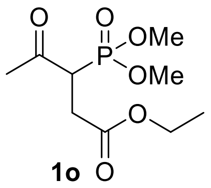

1o

According to the **General Procedure C**, compound **4a** (135.3 mg, 0.815 mmol, 1.0 eq.), 60% sodium hydride (35.8 mg, 0.897 mmol, 1.1 eq.), and ethyl 2-bromoacetate (149.7 mg, 0.897 mmol, 1.1 eq.) were carried out in anhydrous THF (0.1 M). The residue was purified by flash column chromatography on silica gel, eluting with 100% EtOAc (R_f = 0.50) to give **1o** as colorless liquid (86.6 mg, 0.3434 mmol, 42% yield). ^{1}H NMR (400 MHz, $CDCl_3$, ppm) δ = 4.12 (q, J = 7.12 Hz, 2H), 3.779 (d, J = 11.0 Hz, 3H), 3.777 (d, J = 11.0 Hz, 3H), 3.72 (ddd, J = 25.0, 11.2, 3.3 Hz, 1H), 3.14 (ddd, J = 17.9, 11.3, 7.0 Hz, 1H), 2.69 (ddd, J = 17.6, 9.5, 3.2 Hz, 1H), 2.43 (s, 3H), 1.25 (t, J = 7.2 Hz, 3H); ^{13}C{^{1}H} NMR (100 MHz, $CDCl_3$, ppm) δ = 202.1 (d, $J_{C\text{-}P}$ = 3.2 Hz), 171.3 (d, $J_{C\text{-}P}$ = 76.2 Hz), 61.2, 53.5 (d, $J_{C\text{-}P}$ = 6.7 Hz), 53.2 (d, $J_{C\text{-}P}$ = 6.7 Hz), 48.1 (d, $J_{C\text{-}P}$ = 126.7 Hz), 31.4, 31.2, 31.2; ^{31}P NMR (162 MHz, $CDCl_3$, ppm) δ = 23.3; IR (KBr, cm^{-1}) 2961, 1719, 1254, 1159, 1029; HRMS (ESI^{+}) m/z calculated for $(C_9H_{17}O_6P+H)^+$: 253.0836, found: 253.0830.

***tert*-Butyl 3-(dimethoxyphosphoryl)-4-oxopentanoate (1p)**

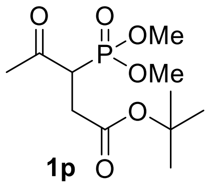

1p

According to the **General Procedure C**, compound **4a** (94.1 mg, 0.567 mmol, 1.0 eq.), 60% sodium hydride (24.9 mg, 0.624 mmol, 1.1 eq.), and *tert*-butyl 2-bromoacetate (121.7 mg, 0.624 mmol, 1.1 eq.) were carried out in anhydrous THF (0.1 M). The residue was purified by flash column chromatography on silica gel, eluting with 100% EtOAc (R_f = 0.50) to give **1p** as colorless liquid (94.3 mg, 0.3365 mmol, 59% yield). ^{1}H NMR (500 MHz, $CDCl_3$, ppm) δ = 3.77 (d, J = 11.0 Hz, 3H), 3.78 (d, J = 11.0 Hz, 3H), 3.67 (ddd, J = 25.2, 11.4, 3.2 Hz, 1H), 3.07 (ddd, J = 17.5, 11.4, 6.9 Hz, 1H), 2.63 (ddd, J = 17.5, 9.4, 3.2 Hz, 1H), 2.42 (s, 3H), 1.42 (s, 9H); ^{13}C{^{1}H} NMR (100 MHz, $CDCl_3$, ppm) δ = 202.2, 170.3 (d, $J_{C\text{-}P}$ = 19.3 Hz), 81.5, 53.5 (d, $J_{C\text{-}P}$ = 6.7 Hz), 53.2 (d, $J_{C\text{-}P}$ = 6.6 Hz), 48.1 (d, $J_{C\text{-}P}$ = 126.0 Hz), 32.3, 31.4, 28.0; ^{31}P NMR (162 MHz, $CDCl_3$, ppm) δ = 23.8; IR (KBr, cm^{-1}) 2980, 1720, 1368 ,1255, 1152, 1031; HRMS (ESI^{+}) m/z calculated for $(C_{11}H_{21}O_6P+H)^+$: 281.1149, found: 281.1149.

Dimethyl (2-oxo-1-phenylpropyl)phosphonate (1q)

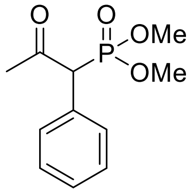

1q

According to the **General Procedure D**, compound **4q** (509.5 mg, 2.545 mmol, 1.0 eq.), *n*-BuLi (1.2 ml, 2.800 mmol, 1.1 eq.), and ethyl acetate (336.4 mg, 3.818 mmol, 1.5 eq.) were carried out in anhydrous THF (0.1 M). The residue was purified by flash column chromatography on silica gel, eluting with 80% v/v EtOAc in hexane (R_f = 0.55) to give **1q** as white solid (258.9 mg, 1.069 mmol, 42%).

Melting point = 53.9-54.4 °C; ^{1}H NMR (400 MHz, $CDCl_3$, ppm) δ = 7.46-7.15 (m, 5H), 4.42 (d, J = 23.5 Hz, 1H), 3.72 (d, J = 11.0 Hz, 3H), 3.67 (d, J = 10.9 Hz, 3H), 2.28 (s, 3H); ^{13}C{^{1}H} NMR (100 MHz, $CDCl_3$, ppm) δ = 201.1 (d, $J_{C\text{-}P}$ = 3.9 Hz), 130.8 (d, $J_{C\text{-}P}$ = 7.9 Hz), 129.7 (d, $J_{C\text{-}P}$ = 7.0 Hz), 128.9, 128.2, 59.6 (d, $J_{C\text{-}P}$ = 133.2 Hz), 53.8 (d, $J_{C\text{-}P}$ = 6.6 Hz), 53.4 (d, $J_{C\text{-}P}$ = 6.9 Hz), 30.5 (d, $J_{C\text{-}P}$ = 3.3 Hz); ^{31}P NMR (202 MHz, $CDCl_3$, ppm) δ = 21.2; IR (KBr, cm^{-1}) 2959, 1719, 1246, 1030, 830; HRMS (ESI$^+$) m/z calculated for $(C_{11}H_{15}O_4P+H)^+$: 243.0781, found: 243.0780.

Dimethyl (1-oxo-1-phenylpropan-2-yl)phosphonate (1r)

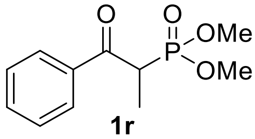

1r

According to the **General Procedure C**, compound **4d** (291.9 mg 1.280 mmol, 1.0 eq.), 60% sodium hydride (56.8 mg, 1.420 mmol, 1.1 eq.), and methyl iodide (199.9 mg, 1.408 mmol, 1.1 eq.) were carried out in anhydrous THF (0.1 M). The residue was purified by flash column chromatography on silica gel, eluting with 100% EtOAc (R_f = 0.50) to give **1r** as colorless oil (65.6 mg, 0.3379 mmol, 26% yield).

^{1}H NMR (400 MHz, $CDCl_3$, ppm) δ = 8.02-7.99 (m, 2H), 7.61-7.57 (m, 1H), 7.51-7.46 (m, 2H), 4.19 (dq, J = 22.9, 7.0 Hz, 1H), 3.77 (d, J = 10.9 Hz, 3H), 3.72 (d, J = 10.9 Hz, 3H), 1.54 (dd, J = 18.2, 7.1 Hz, 3H). All spectroscopic data were in agreement with the literature values.[10]

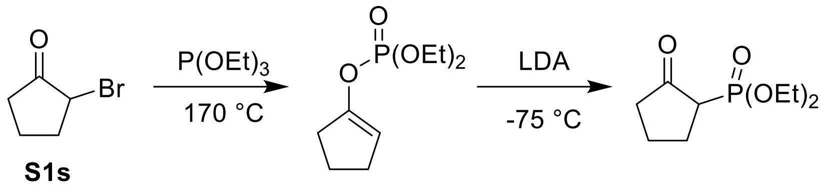

S1s **S2s** **1s**

Cyclopent-1-en-1-yl diethyl phosphate (S2s)

According to the **General Procedure B**, 2-bromocyclopentan-1-one **S1s** (794.9 mg, 4.876 mmol, 1.0 eq.) and triethyl phosphite (1620.5 mg, 9.753 mmol, 2.0 eq.) were carried out at 170 °C (oil bath temperature). The resulting residue was distilled to remove by-products at 120 °C under 650 mbar and yield the desired product **S2s** as colorless liquid (1019.6 mg, 4.630 mmol, 95% yield), which could be used directly for the next step without further purification.

^{1}H NMR (400 MHz, $CDCl_3$, ppm) δ = 5.27-5.24 (m, 1H), 4.22-4.08 (m, 4H), 2.48-2.43 (m, 2H), 2.36-2.30 (m, 2H), 1.97-1.89 (m, 2H), 1.37-1.32 (m, 6H). All spectroscopic data were in agreement with the literature values.[11]

Diethyl (2-oxocyclopentyl)phosphonate (1s)

Compound **S2s** (1019.6 mg, 4.630 mmol, 1.0 eq.) in THF (10 ml) was slowly dropped into the solution of LDA (2.3 eq.) in THF (6 ml) at -75 °C. After stirring for 1.5 h, the reaction was added by the solution of AcOH (4 eq.) in ether (1 M) and then moved to the room temperature. The reaction was filtered and concentrated under reduced pressure to give the crude residue, which was purified by flash column chromatography on silica gel, eluting with 80% v/v EA in hexane (R_f = 0.50) to yield compound **1s** as yellow oil (387.9 mg, 1.762 mmol, 38% yield).

^{1}H NMR (400 MHz, $CDCl_3$, ppm) δ = 4.25-4.09 (m, 4H), 2.78-2.68 (m, 1H), 2.42-2.09 (m, 5H), 1.95-1.86 (m, 1H), 1.39-1.26 (m, 6H). All spectroscopic data were in agreement with the literature values.[12]

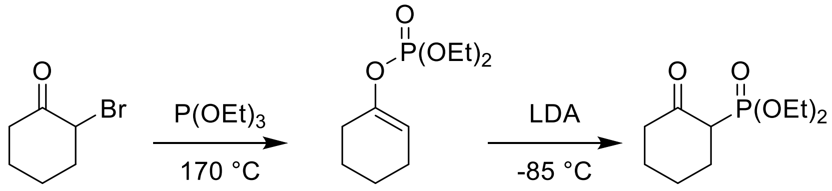

S1t **S2t** **1t**

Cyclohex-1-en-1-yl diethyl phosphate (S2t)

According to the **General Procedure B**, 2-bromocyclohexan-1-one **S1t** (1011.7 mg, 5.715 mmol, 1.0 eq.) and triethyl phosphite (2 ml, 11.429 mmol, 2.0 eq.) were carried

out at 170 °C (oil bath temperature). The resulting residue was distilled to remove by-products at 120 °C under 650 mbar and yield the desired product **S2t** as colorless liquid (1162.8 mg, 4.964 mmol, 87% yield), which could be used directly for the next step without further purification.
^{1}H NMR (400 MHz, $CDCl_3$, ppm) δ = 5.49-5.46 (m, 1H), 4.19-4.11 (m, 4H), 2.22-2.18 (m, 2H), 2.10-2.05 (m, 2H), 1.74-1.68 (m, 2H), 1.59-1.53 (m, 2H), 1.37-1.32 (m, 6H). All spectroscopic data were in agreement with the literature values.[12]

Diethyl (2-oxocyclohexyl)phosphonate (1t)

Compound **S2t** (248.0 mg, 1.059 mmol, 1.0 eq.) in THF (10 ml) was slowly dropped into the solution of LDA (2.2 eq.) in THF (6 ml) at -85 °C. After stirring for 1.5 h, the reaction was added by the solution of AcOH (4 eq.) in ether (1 M) and then moved to the room temperature. The reaction was filtered and concentrated under reduced pressure to give the crude residue, which was purified by flash column chromatography on silica gel, eluting with 60% v/v EA in hexane (R_f = 0.30) to yield compound **1s** as colorless oil (153.1 mg, 0.654 mmol, 62% yield).
^{1}H NMR (400 MHz, $CDCl_3$, ppm) δ = 10.75 (s, 0.1H, enol form), 4.19-4.00 (m, 4H), 2.96 (dt, J = 23.4, 6.0 Hz, 0.9H, ketol form), 2.68-2.60 (m, 0.9H), 2.39-1.58 (m, 7.1H), 1.39-1.26 (m, 6H). All spectroscopic data were in agreement with the literature values.[13]

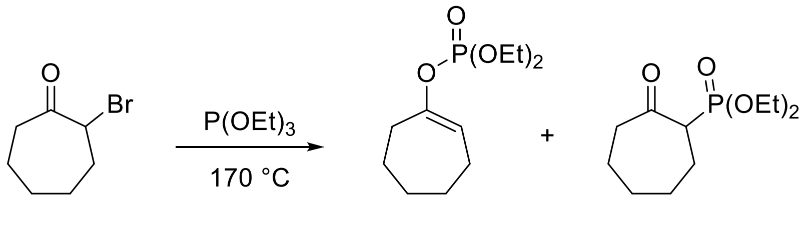

S1u **S2u** **1u**

Cyclohept-1-en-1-yl diethyl phosphate (S2u) and **diethyl (2-oxocycloheptyl)-phosphonate (1u)**

According to the **General Procedure B**, 2-bromocycloheptan-1-one **S1u** (1010.7 mg, 5.290 mmol, 1.0 eq.) and triethyl phosphite (1758.0 mg, 10.580 mmol, 2.0 eq.) were carried out at 170 °C (oil bath temperature). The resulting residue was distilled to remove by-products at 120 °C under 650 mbar. The crude residue was purified by flash column chromatography on silica gel, eluting with 100% EA (R_f = 0.80 for **S2u**, R_f = 0.50 for **1u**) to yield compound **S2u** as colorless oil (906.2 mg, 3.650 mmol, 69% yield) and compound **1u** as colorless liquid (341.5 mg, 1.375 mmol, 26% yield).

cyclohept-1-en-1-yl diethyl phosphate (S2u): ^{1}H NMR (400 MHz, $CDCl_3$, ppm) δ =

5.64-5.61 (m, 1H), 4.18-4.11 (m, 4H), 2.43-2.39 (m, 2H), 2.10-2.04 (m, 2H), 1.73-1.53 (m, 6H), 1.37-1.33 (m, 6H). All spectroscopic data were in agreement with the literature values.[14]

diethyl (2-oxocycloheptyl)phosphonate (1u) : ^{1}H NMR (400 MHz, $CDCl_3$, ppm) δ = 10.94 (s, 0.03 H, enol form), 4.17-4.09 (m, 4H), 3.03 (ddd, J = 25.2, 12.1, 5.0 Hz, 0.97H ketol form), 2.84-2.77 (m, 1H), 2.49-2.45 (m, 1H), 2.26-2.21 (m, 1H), 2.10-1.86 (m, 4H), 1.45-1.38 (m, 2H), 1.36-1.30 (m, 6H), 1.26-1.14 (m, 1H). All spectroscopic data were in agreement with the literature values.[15]

Diethyl (1-oxo-3-phenylpropan-2-yl)phosphonate (1v)

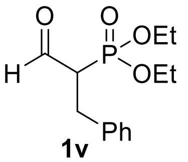

1v

According to the **General Procedure D**, compound **4v** (197.1 mg, 0.814 mmol, 1.0 eq.), *n*-BuLi (0.36 ml, 0.895 mmol, 1.1 eq.), and ethyl formate (90.5 mg, 1.221 mmol, 1.5 eq.) were carried out in anhydrous THF (0.1 M). The residue was purified by flash column chromatography on silica gel, eluting with 100% EtOAc (R_f = 0.50) to give **1v** as colorless liquid (171.2 mg, 0.634 mmol, 78% yield).
^{1}H NMR (400 MHz, $CDCl_3$, ppm) δ = 9.68 (d, J = 1.9 Hz, 1H), 7.29-7.18 (m, 5H), 4.19-4.11 (m, 4H), 3.43-3.32 (m, 2H), 3.13 (ddd, $^{2}J_{H-H}$ = 12.8 Hz, $^{3}J_{P-H}$ = 12.8 Hz, $^{3}J_{H-H}$ = 2.3 Hz, 1H), 1.34 (t, J = 7.1 Hz, 3H), 1.33 (t, J = 7.0 Hz, 3H). All spectroscopic data were in agreement with the literature values.[16]

Diethyl (1-oxoheptan-2-yl)phosphonate (1w)

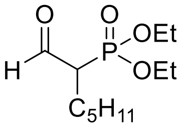

1w

According to the **General Procedure D**, **4l** (217.7 mg, 0.824 mmol, 1.0 eq.), *n*-BuLi (0.36 ml, 0.895 mmol, 1.1 eq.), and ethyl formate (91.6 mg, 1.237 mmol, 1.5 eq.) were carried out in anhydrous THF (0.1 M). The residue was purified by flash column chromatography on silica gel, eluting with 100% EtOAc (R_f = 0.70) to give **1w** as colorless liquid (165.9 mg, 0.663 mmol, 80% yield).
^{1}H NMR (400 MHz, $CDCl_3$, ppm) δ = 9.63 (d, J = 3.0 Hz, 1H), 4.19-4.11 (m, 4H), 3.00-2.90 (m, 1H), 1.90-1.60 (m, 8H), 1.34 (t, J = 7.1 Hz, 3H), 1.33 (t, J = 7.0 Hz, 3H), 0.88

(t, $J = 6.7$ Hz, 3H). All spectroscopic data were in agreement with the literature values.[17]

Dimethyl (2-oxo-1-phenylethyl)phosphonate (1x)

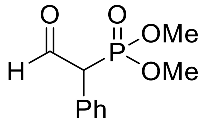

1x

According to the **General Procedure D**, **4q** (76.4 mg, 0.382 mmol 1.0 eq.), *n*-BuLi (0.17 ml, 0.425 mmol, 1.1 eq.), and methyl formate (34.7 mg, 0.573 mmol, 1.5 eq.) were carried out in anhydrous THF (0.1 M). The residue was purified by flash column chromatography on silica gel, eluting with 100% EtOAc (R_f = 0.50) to give **1x** as colorless liquid (57.5 mg, 0.252 mmol, 66% yield).

^{1}H NMR (400 MHz, $CDCl_3$, ppm) δ = 9.80 (t, $J = 3.0$ Hz, 1H), 7.52-7.20 (m, 5H), 4.25 (dd, $J = 26.0, 2.7$ Hz, 1H), 3.79 (d, $J = 11.1$ Hz, 3H), 3.62 (d, $J = 11.0$ Hz, 3H). All spectroscopic data were in agreement with the literature values.[18]

Dimethyl (1-fluoro-2-oxopropyl)phosphonate (1y)

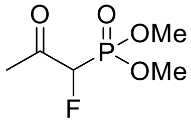

1y

According to the **General Procedure E**, compound **4a** (166.9 mg, 1.005 mmol, 1.0 eq.) and Selectfluor (711.6 mg, 2.009 mmol, 2.0 eq.) were carried out in anhydrous acetonitrile (0.05 M). The residue was purified by flash column chromatography on silica gel, eluting with 100% EtOAc (R_f= 0.50) give **1y** as colorless oil (24.2 mg, 0.1314 mmol, 13% yield).

^{1}H NMR (500 MHz, $CDCl_3$, ppm) δ = 5.22 (dd, $J = 48.0, 14.4$ Hz, 1H), 3.88 (d, $J =$ 11.0 Hz, 3H), 3.88 (d, $J = 11.0$ Hz, 3H), 3.67 (dd, $J = 4.55, 0.7$ Hz, 3H). All spectroscopic data were in agreement with the literature values.[19]

Diethyl (1-fluoro-2-oxo-2-phenylethyl)phosphonate (1z)

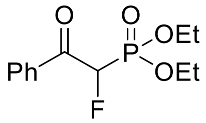

1z

According to the **General Procedure E**, diethyl (2-oxo-2-phenylethyl)phosphonate **4z** (252.8 mg, 0.987 mmol, 1.0 eq.) and Selectfluor (699.0 mg, 1.973 mmol, 2.0 eq.) were carried out in anhydrous acetonitrile (0.05 M). The residue was purified by flash column

chromatography on silica gel, eluting with 100% EtOAc (R_f = 0.50) give **1z** as yellow oil (56.1 mg, 0.205 mmol, 21% yield).
^{1}H NMR (400 MHz, $CDCl_3$, ppm) δ = 8.05-8.02 (m, 2H), 7.64-7.60 (m, 1H), 7.51-7.47 (m, 2H), 5.97 (dd, *J* = 47.4, 13.4 Hz, 1H), 4.29-4.11 (m, 4H), 1.35 (t, *J* = 7.1 Hz, 3H), 1.27 (t, *J* = 7.1 Hz, 3H). All spectroscopic data were in agreement with the literature values.[20]

4.3 General procedure for the synthesis of compound 2

General Procedure F:

$$ArNH_2 \xrightarrow[\text{2. } NaN_3]{\text{1. } NaNO_2,\ 6N\ HCl_{(aq)}} \underset{\textbf{2a-2f}}{ArN_3}$$

Aniline (1.0 eq.) in 6 N HCl (5 ml) was added by 2 ml $NaNO_2$ (1.5 eq.) aqueous solution at 0 °C. After stirring for 0.5 h, sodium azide (NaN_3) (1.5 eq.) dissolved in water (2 ml) was added dropwise into the solution at 0 °C and the reaction mixture was moved to the room temperature. After the reaction was completed according to thin layer chromatography (TLC), the solution was directly extracted with DCM. The organic layers were all combined, washed with brine, dried over anhydrous $MgSO_4$, filtered, and concentrated under reduced pressure to give the crude residue, which could be used directly for the next step without further purification.

General Procedure G:

$$RX + NaN_3 \xrightarrow[80\ °C]{DMSO} \underset{\textbf{2h}}{RN_3} + NaX$$

The solution of alkyl halide or sulfonyl halide (1 eq.) in DMSO (0.5 M) was added by NaN_3 (1.1 eq.) and stirred at 80 °C (oil bath temperature) for overnight. The reaction was cooled to room temperature before it was quenched with water (5mL) and then extracted with EA (3 x 10 ml). The organic layers were all combined, washed with brine, dried over anhydrous $MgSO_4$, filtered, and concentrated under reduced pressure to give the crude residue, which could be used directly for the next step without further purification. Compound **2h** was prepared according to this identical synthetic procedure.[21]

Azidobenzene (2a)

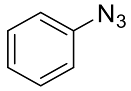

2a

According to the **General Procedure F**, aniline (209.0 mg, 2.244 mmol, 1.0 eq.), $NaNO_2$ (232.2 mg, 3.366 mmol, 1.5 eq.), and NaN_3 (218.8 mg, 3.366 mmol, 1.5 eq.) were carried out in the reaction. Compound **2a** was finally obtained as yellow liquid (140.7 mg, 1.153 mmol, 51% yield).

^{1}H NMR (400 MHz, $CDCl_3$, ppm) δ = 7.37-7.34 (m, 2H), 7.16-7.14 (m, 1H), 7.05-7.02 (m, 2H). All spectroscopic data were in agreement with the literature values.[22]

1-Azido-4-chlorobenzene (2b)

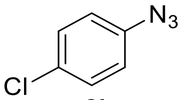

2b

According to the **General Procedure F**, 4-chloroaniline (332.0 mg, 2.603 mmol, 1.0 eq.), $NaNO_2$ (269.4 mg, 3.905 mmol, 1.5 eq.), and NaN_3 (253.8 mg, 3.905 mmol, 1.5 eq.) were carried out in the reaction. Compound **2b** was finally obtained as yellow liquid (390.5 mg, 2.543 mmol, 98% yield).

^{1}H NMR (400 MHz, $CDCl_3$, ppm) δ = 7.33-7.30 (m, 2H), 6.98-6.94 (m, 2H). All spectroscopic data were in agreement with the literature values.[23]

1-Azido-4-bromobenzene (2c)

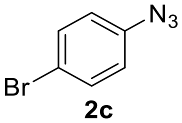

2c

According to the **General Procedure F**, 4-bromoaniline (202.4 mg, 1.177 mmol, 1.0 eq.), $NaNO_2$ (121.8 mg, 1.766 mmol, 1.5 eq.), NaN_3 (114.8 mg, 1.766 mmol, 1.5 eq.) were carried out in the reaction. Compound **2c** was finally obtained as yellow liquid (220.7 mg, 1.115 mmol, 95% yield).

^{1}H NMR (400 MHz, $CDCl_3$, ppm) δ = 7.48-7.44 (m, 2H), 6.92-6.89 (m, 2H). All spectroscopic data were in agreement with the literature values.[23]

1-Azido-4-methoxybenzene (2d)

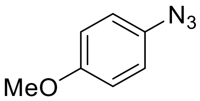

2d

According to the **General Procedure F**, 4-methoxyaniline (326.7 mg, 2.653 mmol, 1.0

eq.), $NaNO_2$ (274.5 mg, 3.980 mmol, 1.5 eq.), NaN_3 (258.7 mg, 3.980 mmol, 1.5 eq.) were carried out in the reaction. Compound **2d** was finally obtained as black liquid (352.4 mg, 2.363 mmol, 89% yield).
^{1}H NMR (400 MHz, $CDCl_3$, ppm) δ = 6.97-6.94 (m, 2H), 6.91-6.88 (m, 2H), 3.80 (s, 3H). All spectroscopic data were in agreement with the literature values.[23]

1-Azido-4-nitrobenzene (2e)

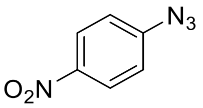

2e

According to the **General Procedure F**, 4-nitroaniline (101.2 mg, 0.7327 mmol, 1.0 eq.), $NaNO_2$ (75.8 mg, 1.099 mmol, 1.5 eq.), NaN_3 (71.4 mg, 1.099 mmol, 1.5 eq.) were carried out in the reaction. Compound **2e** was finally obtained as orange solid (118.9 mg, 0.7245 mmol, 99% yield).
^{1}H NMR (400 MHz, $CDCl_3$, ppm) δ = 8.27-8.23 (m, 2H), 7.16-7.12 (m, 2H). All spectroscopic data were in agreement with the literature values.[23]

Methyl 4-azidobenzoate (2f)

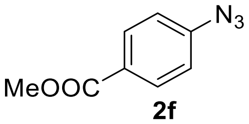

2f

According to the **General Procedure F**, methyl 4-aminobenzoate (203.7 mg, 1.323 mmol, 1.0 eq.), $NaNO_2$ (136.9 mg, 1.985 mmol, 1.5 eq.), NaN_3 (129.0 mg, 1.985 mmol, 1.5 eq.) were carried out in the reaction. Compound **2f** was finally obtained as orange solid (225.3 mg, 1.272 mmol, 96% yield).
^{1}H NMR (400 MHz, $CDCl_3$, ppm) δ = 8.05-8.02 (m, 2H), 7.08-7.06 (m, 2H), 3.91 (s, 3H). All spectroscopic data were in agreement with the literature values.[24]

Tosyl azide (2g)

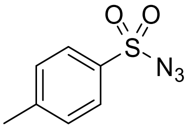

2g

NaN_3 (115.1 mg, 1.770 mmol, 1.1 eq.) in the mixture of acetone (2.2 ml) and water (1.3 ml) was added by tosyl chloride (306.8 mg, 1.609 mmol, 1.0 eq.) in acetone (2.2 ml). The reaction was stirred for 2 hours and then extracted with DCM (3 x 10 ml). The organic layers were all combined, washed with brine, dried over anhydrous $MgSO_4$,

filtered, and concentrated under reduced pressure to give tosyl azide **2g** as slight yellow liquid (307.2 mg, 1.607 mmol, 99% yield), which could be used directly for the next step without further purification.

^1H NMR (400 MHz, $CDCl_3$, ppm) δ = 7.86-7.83 (m, 2H), 7.42-7.39 (m, 2H), 2.49 (s, 3H). All spectroscopic data were in agreement with the literature values.[25]

Benzyl azide (2h)

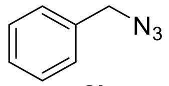

2h

According to the **General Procedure G**, (2-bromoethyl)benzene (309.4 mg, 1.809 mmol, 1.0 eq.), NaN_3 (129.4 mg, 1.990 mmol, 1.1 eq.) were carried out in the reaction. Compound **2h** was finally obtained as clear liquid (215.7 mg, 1.620 mmol, 90% yield).

^1H NMR (400 MHz, $CDCl_3$, ppm) δ = 7.42-7.30 (m, 5H), 4.35 (s, 2H). All spectroscopic data were in agreement with the literature values.[22]

4.4 General procedure for the synthesis of compound 3 or 5

General Procedure H:

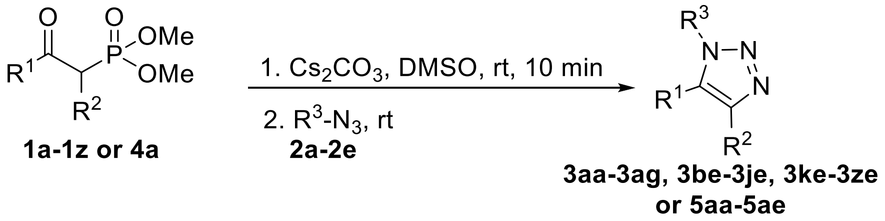

Compound **1** (1.0 eq.) and cesium carbonate (2.0 eq.) were mixed in DMSO (0.3 M) for 10 min, and then compound **2** (1.2 eq.) in DMSO (0.3 M) was injected into the resulting reaction. After the reaction was completed according to thin layer chromatography (TLC), the solution was diluted with EtOAc (10 mL) and was washed with brine (15 ml × 3) to remove DMSO. Occasionally, the product might be found in aqueous layer. Then the aqueous layer could be extracted with additional EtOAc if needed. The organic layers were all combined, dried over anhydrous $MgSO_4$, filtered, and concentrated under reduced pressure to give the crude residue. The residue was purified by flash column chromatography on silica gel to give the desired product.

4-Benzyl-5-methyl-1-phenyl-1H-1,2,3-triazole (3aa)

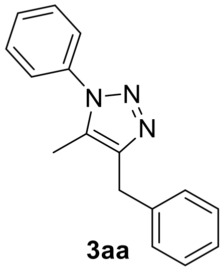

3aa

According to the **General Procedure H**, compound **1a** (62.1 mg, 0.242 mmol, 1.0 eq.), cesium carbonate (159.2 mg, 0.489 mmol, 2.0 eq.), and compound **2a** (34.7 mg, 0.291 mmol, 1.2 eq.) were carried out in the reaction. The residue was purified by flash column chromatography on silica gel, eluting with 40% v/v EtOAc in hexane (R_f = 0.60) to give **3aa** as brown liquid (48.6 mg, 0.195 mmol, 80% yield).

^{1}H NMR (400 MHz, $CDCl_3$, ppm) δ = 7.55-7.43 (m, 5H), 7.34-7.28 (m, 4H), 7.24-7.20 (m, 1H), 4.12 (s, 2H), 2.18 (s, 3H); ^{13}C{^{1}H} NMR (100 MHz, $CDCl_3$, ppm) δ = 144.1, 139.1, 136.7, 130.3, 129.5, 129.3, 128.6, 126.4, 125.0, 31.8, 9.0; ^{13}C{^{1}H} NMR (125 MHz, $CDCl_3$, ppm) δ = 144.0, 138.9, 136.6, 130.1, 129.3, 129.1, 128.5, 126.3, 124.8, 31.6, 8.81; ^{13}C{^{1}H} NMR (125 MHz, DMSO-d_6, ppm) δ = 143.5, 139.5, 136.4, 130.5, 129.7, 129.3, 128.51, 128.49, 126.2, 124.8, 30.6, 8.4; IR (KBr, cm^{-1}) 3029, 1599, 1505, 765; HRMS (ESI^+) m/z calculated for $(C_{16}H_{15}N_3+H)^+$: 250.1339, found 250.1338.

4-Benzyl-1-(4-chlorophenyl)-5-methyl-1H-1,2,3-triazole (3ab)

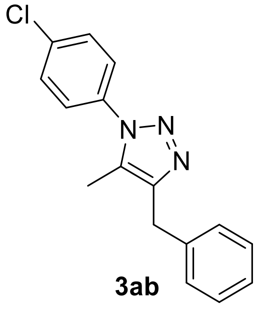

3ab

According to the **General Procedure H**, compound **1a** (28.6 mg, 0.112 mmol, 1.0 eq.), cesium carbonate (73.0 mg, 0.224 mmol, 2.0 eq.), compound **2b** (21.0 mg, 0.137 mmol, 1.2 eq.) were carried out in the reaction. The residue was purified by flash column chromatography on silica gel, eluting with 40% v/v EtOAc in hexane (R_f = 0.80) to give **3ab** as brown liquid (28 mg, 0.099 mmol, 88% yield).

^{1}H NMR (400 MHz, $CDCl_3$, ppm) δ = 7.50 (d, J = 8.5 Hz, 2H), 7.40 (d, J = 8.5 Hz, 2H), 7.31-7.21 (m, 5H), 4.10 (s, 2H), 2.17 (s, 3H); ^{13}C{^{1}H} NMR (100 MHz, $CDCl_3$, ppm) δ = 144.4, 138.9, 135.4, 135.2, 130.3, 129.8, 128.7, 128.7, 126.5, 126.2, 31.8, 9.0; IR (KBr, cm^{-1}) 2925, 1501, 1090, 834, 728; HRMS (ESI^+) m/z calculated for $(C_{16}H_{14}ClN_3+H)^+$: 284.0949, found 284.0947.

4-Benzyl-1-(4-bromophenyl)-5-methyl-1H-1,2,3-triazole (3ac)

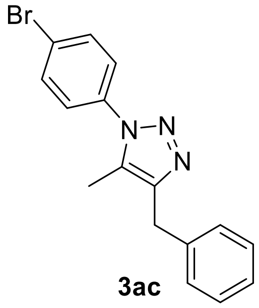

3ac

According to the **General Procedure H**, compound **1a** (56.2 mg, 0.219 mmol, 1.0 eq.), cesium carbonate (145.8 mg, 0.447 mmol, 2.0 eq.), compound **2c** (52.1 mg, 0.263 mmol, 1.2 eq.) were carried out in the reaction. The residue was purified by flash column chromatography on silica gel, eluting with 40% v/v EtOAc in hexane (R_f = 0.75) to give **3ac** as brown solid (57.0 mg, 0.174 mmol, 79%).

Melting point = 85.1-86.1 °C; ^{1}H NMR (400 MHz, $CDCl_3$, ppm) δ = 7.66 (d, J = 8.8 Hz, 2H), 7.34 (d, J = 8.7 Hz, 2H), 7.31-7.21 (m, 5H), 4.11 (s, 2H), 2.18 (s, 3H); $^{13}C\{^1H\}$ NMR (100 MHz, $CDCl_3$, ppm) δ = 144.4, 138.9, 135.7, 132.8, 130.2, 128.7, 128.6, 126.5, 126.4, 123.3, 31.8, 9.0; IR (KBr, cm^{-1}) 2925, 1496, 1070, 834, 726; HRMS (ESI^+) m/z calculated for $(C_{16}H_{14}BrN_3+H)^+$: 328.0444, found 328.0443.

4-Benzyl-1-(4-methoxyphenyl)-5-methyl-1H-1,2,3-triazole (3ad)

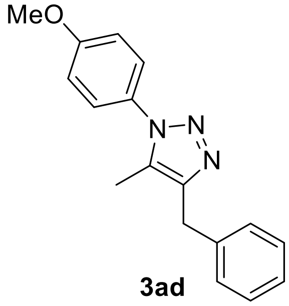

3ad

According to the **General Procedure H**, compound **1a** (40.8 mg, 0.159 mmol, 1.0 eq.), cesium carbonate (103.7 mg, 0.318 mmol, 2.0 eq.), compound **2d** (28.6 mg, 0.192 mmol, 1.2 eq.) were carried out in the reaction. The residue was purified by flash column chromatography on silica gel, eluting with 40% v/v EtOAc in hexane (R_f = 0.55) to give **3ad** as white oil (30.7 mg, 0.110 mmol, 69%).

^{1}H NMR (400 MHz, $CDCl_3$, ppm) δ = 7.34 (d, J = 8.9 Hz, 2H), 7.30-7.27 (m, 4H), 7.24-7.19 (m, 1H), 7.01 (d, J = 8.9 Hz, 2H), 4.10 (s, 2H), 3.86 (s, 3H), 2.13 (s, 3H); $^{13}C\{^1H\}$ NMR (100 MHz, $CDCl_3$, ppm) δ = 160.3, 143.9, 139.2, 130.4, 129.8, 128.70, 128.67, 126.54, 126.46, 114.7, 55.7, 31.9, 8.9; IR (KBr, cm^{-1}) 2926, 1519, 1253, 1035, 836, 731; HRMS (ESI^+) m/z calculated for $(C_{17}H_{17}N_3O+H)^+$: 280.1444, found 280.1442.

4-Benzyl-5-methyl-1-(4-nitrophenyl)-1H-1,2,3-triazole (3ae)

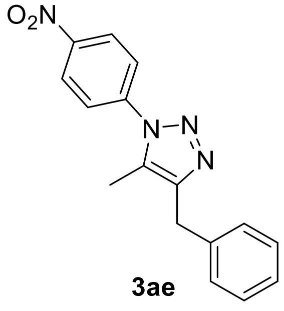

3ae

According to the **General Procedure H**, compound **1a** (43.5 mg, 0.170 mmol, 1.0 eq.), cesium carbonate (110.5 mg, 0.339 mmol, 2.0 eq.), compound **2e** (34.1 mg, 0.208 mmol, 1.2 eq.) were carried out in the reaction. The residue was purified by flash column chromatography on silica gel, eluting with 40% v/v EtOAc in hexane (R_f= 0.50) to give **3ae** as brown solid (48.1 mg, 0.163 mmol, 96% yield).

A larger scale was carried out with compound **1a** (262 mg, 1.022 mmol, 1.0 eq.), cesium carbonate (667 mg, 2.047 mmol, 2.0 eq.), and compound **2e** (201 mg, 1.225 mmol, 1.2 eq.) to afford compound **3ae** (280 mg, 0.951 mmol, 93% yield) according to the General Procedure H.

Melting point = 105.2-105.8 °C; ^{1}H NMR (400 MHz, $CDCl_3$, ppm) δ = 8.42 (d, J = 9.1 Hz, 2H), 7.72 (d, J = 9.1 Hz, 2H), 7.34-7.28 (m, 4H), 7.25-7.22 (m, 1H), 4.13 (s, 2H), 2.28 (s, 3H); ^{13}C{^{1}H} NMR (100 MHz, $CDCl_3$, ppm) δ = 147.6, 145.2, 141.5, 138.6, 130.2, 128.7, 128.6, 126.6, 125.1, 125.0, 31.6, 9.4; IR (KBr, cm^{-1}) 2925, 1598, 1522, 1345, 1089, 856, 727; HRMS (ESI$^+$) m/z calculated for $(C_{16}H_{14}N_4O_2+H)^+$: 295.1190, found 295.1188.

Methyl 4-(4-benzyl-5-methyl-1H-1,2,3-triazol-1-yl)benzoate (3af)

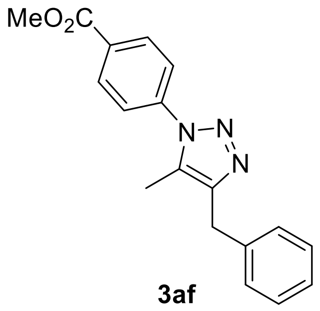

3af

According to the **General Procedure H**, compound **1a** (39.0 mg, 0.152 mmol, 1.0 eq.), cesium carbonate (102.2 mg, 0.314 mmol, 2.0 eq.), compound **2f** (32.3 mg, 0.182 mmol, 1.2 eq.) were carried out in the reaction. The residue was purified by flash column chromatography on silica gel, eluting with 40% v/v EtOAc in hexane (R_f= 0.55) to give **3af** as brown solid (45.2 mg, 0.147 mmol, 97% yield).

Melting point = 83.2-84.2 °C; ^{1}H NMR (400 MHz, $CDCl_3$, ppm) δ = 8.21 (d, J = 8.5

Hz, 2H), 7.57 (d, J =8.6 Hz, 2H), 7.33-7.29 (m, 4H), 7.24 (m, 1H), 4.12 (s, 2H), 3.96 (s, 3H), 2.23 (s, 3H); $^{13}C\{^1H\}$ NMR (100 MHz, $CDCl_3$, ppm) δ = 166.0, 144.7, 140.2, 138.8, 131.0, 130.7, 130.2, 128.7, 128.6, 126.5, 124.5, 52.6, 31.7, 9.2; IR (KBr, cm^{-1}) 2952, 1723, 1608, 1436, 1281, 1111; HRMS (ESI^+) m/z calculated for $(C_{18}H_{17}N_3O_2+H)^+$: 308.1394, found 308.1371.

4-Benzyl-5-methyl-1-tosyl-1H-1,2,3-triazole (3ag)

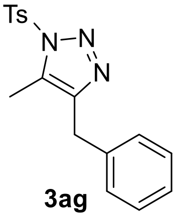

3ag

According to the **General Procedure H**, compound **1a** (34.6 mg, 0.135 mmol, 1.0 eq.), cesium carbonate (88.7 mg, 0.272 mmol, 2.0 eq.), compound **2g** (32.7 mg, 0.166 mmol, 1.2 eq.) were carried out in the reaction. The residue was purified by flash column chromatography on silica gel, eluting with 20% v/v EtOAc in hexane (R_f = 0.4) to give **3ag** as white solid (39.2 mg, 0.120 mmol, 89% yield).

Melting point = 79.9-81.2 °C; ^1H NMR (400 MHz, $CDCl_3$, ppm) δ = 7.94 (d, J = 8.4 Hz, 2H), 7.33 (d, J = 8.0 Hz, 2H), 7.28-7.20 (m, 3H), 7.09-7.07 (m, 2H), 4.01 (s, 2H), 2.43 (s, 3H), 2.08 (s, 3H); $^{13}C\{^1H\}$ NMR (100 MHz, $CDCl_3$, ppm) δ = 150.8, 148.6, 146.3, 136.5, 133.4, 130.1, 128.8, 128.6, 128.5, 126.9, 31.4, 21.9, 10.6; IR (KBr, cm^{-1}) 2927, 1596, 1480, 1393, 1196, 1090, 950; HRMS (ESI^+) m/z calculated for $(C_{17}H_{17}N_3O_2S+H)^+$: 328.1114, found 328.1109.

4-Benzyl-1-(4-nitrophenyl)-5-pentyl-1H-1,2,3-triazole (3be)

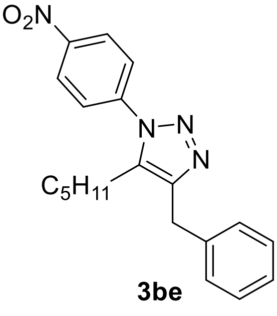

3be

According to the **General Procedure H**, compound **1b** (32.6 mg, 0.104 mmol, 1.0 eq.), cesium carbonate (68.0 mg, 0.209 mmol, 2.0 eq.), compound **2e** (20.6 mg, 0.125 mmol, 1.2 eq.) were carried out in the reaction. The residue was purified by flash column chromatography on silica gel, eluting with 20% v/v EtOAc in hexane (R_f = 0.68) to give **3be** as light-yellow oil (36.9 mg, 0.104 mmol, 99% yield).

^1H NMR (400 MHz, $CDCl_3$, ppm) δ = 8.42 (d, J = 9.2 Hz, 2H), 7.68 (d, J = 9.0 Hz,

2H), 7.34-7.29 (m, 4H), 7.25-7.21 (m, 1H), 4.13 (s, 2H), 2.64-2.60 (m, 2H), 1.30-1.07 (m, 6H), 0.76 (t, *J* = 6.8 Hz, 3H); $^{13}C\{^1H\}$ NMR (100 MHz, $CDCl_3$, ppm) δ = 147.9, 144.9, 141.9, 138.8, 134.9, 128.7, 128.6, 126.6, 125.7, 125.1, 31.7, 31.3, 28.3, 22.9, 22.1, 13.8; IR (KBr, cm^{-1}) 2928, 1598, 1526, 1346, 855, 697; HRMS (ESI^+) m/z calculated for $(C_{20}H_{22}N_4O_2+H)^+$: 351.1815, found 351.1812.

4-Benzyl-5-(tert-butyl)-1-(4-nitrophenyl)-1H-1,2,3-triazole (3ce)

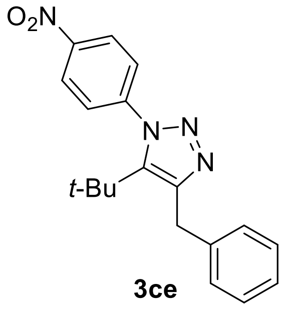

3ce

According to the **General Procedure H**, compound **1c** (46.1 mg, 0.155 mmol, 1.0 eq.), cesium carbonate (100.8 mg, 0.309 mmol, 2.0 eq.), compound **2e** (30.5 mg, 0.186 mmol, 1.2 eq.) were carried out in the reaction. The residue was purified by flash column chromatography on silica gel, eluting with 30% v/v EtOAc in hexane (R_f = 0.50) to give **3ce** as yellow solid (40.0 mg, 0.119 mmol, 77% yield).

Melting point = 150.5-150.9 °C; ^1H NMR (400 MHz, $CDCl_3$, ppm) δ = 8.39 (d, *J* = 8.9 Hz, 2H), 7.61 (d, *J* = 8.8 Hz, 2H), 7.33-7.29 (m, 2H), 7.23-7.20 (m, 3H), 4.33 (s, 2H), 1.16 (s, 9H); $^{13}C\{^1H\}$ NMR (100 MHz, $CDCl_3$, ppm) δ = 148.6, 144.7, 142.8, 142.7, 139.7, 129.4, 128.6, 128.4, 126.4, 124.4, 33.6, 32.0, 31.1; IR (KBr, cm^{-1}) 2970, 1598, 1531, 1496, 1349; HRMS (ESI^+) m/z calculated for $(C_{19}H_{20}N_4O_2 +H)^+$: 337.1659, found 337.1652.

4-Benzyl-1-(4-nitrophenyl)-5-phenyl-1H-1,2,3-triazole (3de)

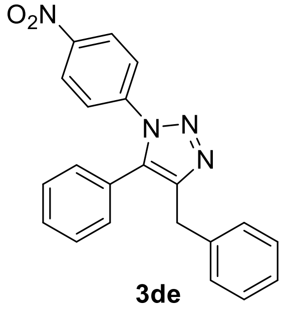

3de

According to the **General Procedure H**, compound **1d** (47.3 mg, 0.149 mmol, 1.0 eq.), cesium carbonate (98.7 mg, 0.303 mmol, 2.0 eq.), compound **2e** (29.5 mg, 0.180 mmol, 1.2 eq.) were carried out in the reaction. The residue was purified by flash column chromatography on silica gel, eluting with 20% v/v EtOAc in hexane (R_f = 0.48) to give

3de as light-yellow oil (47.5 mg, 0.133 mmol, 90% yield).

^{1}H NMR (400 MHz, $CDCl_3$, ppm) δ = 8.23 (d, *J* = 9.1 Hz, 2H), 7.50 (d, *J* = 9.1 Hz, 2H), 7.48-7.38 (m, 3H), 7.30-7.27 (m, 1H),7.26-7.19 (m, 4H), 7.12-7.10 (m, 2H), 4.10 (s, 2H); $^{13}C\{^1H\}$ NMR (100 MHz, $CDCl_3$, ppm) δ = 147.2, 145.8, 141.5, 138.9, 134.6, 129.9, 129.7, 129.4, 128.6, 126.7, 126.6, 124.8, 31.2; IR (KBr, cm^{-1}) 3087, 2924, 1598, 1525, 1347; HRMS (ESI^+) m/z calculated for $(C_{21}H_{16}N_4O_2 +H)^+$: 357.1346, found 357.1342. Note: Due to the overlap of signals, the observed peaks have been reported accordingly.

4-Benzyl-5-(4-methoxyphenyl)-1-(4-nitrophenyl)-1H-1,2,3-triazole (3ee)

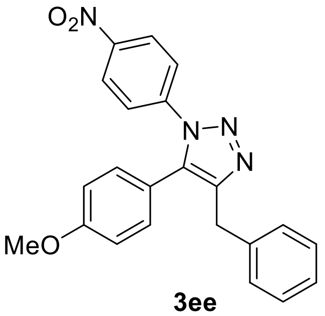

3ee

According to the **General Procedure H**, compound **1e** (34.3 mg, 0.098 mmol, 1.0 eq.), cesium carbonate (64.2 mg, 0.196 mmol, 2.0 eq.), compound **2e** (19.4 mg, 0.118 mmol, 1.2 eq.) were carried out in the reaction. The residue was purified by flash column chromatography on silica gel, eluting with 30% v/v EtOAc in hexane (R_f = 0.48) to give **3ee** as light-yellow oil (38.8 mg, 0.090 mmol, 92% yield).

^{1}H NMR (400 MHz, $CDCl_3$, ppm) δ = 8.23 (d, *J* = 9.0 Hz, 2H), 7.52 (d, *J* = 9.0 Hz, 2H), 7.30-7.28 (m, 2H), 7.25-7.19 (m, 3H), 7.00 (d, *J* = 8.8 Hz, 2H), 6.93 (d, *J* = 8.7 Hz, 2H), 4.09 (s, 2H), 3.85 (s, 3H); $^{13}C\{^1H\}$ NMR (100 MHz, $CDCl_3$, ppm) δ = 160.7, 147.2, 145.6, 141.7, 139.1, 134.6, 131.1, 128.7, 126.5, 124.8, 118.5, 114.9, 55.5, 31.3; IR (KBr, cm^{-1}) 2933, 1597, 1504, 1347, 1253, 1178; HRMS (ESI^+) m/z calculated for $(C_{22}H_{18}N_4O_3+H)^+$: 387.1452, found 387.1446. Note: Due to the overlap of signals, the observed peaks have been reported accordingly.

4-Benzyl-5-(4-fluorophenyl)-1-(4-nitrophenyl)-1H-1,2,3-triazole (3fe)

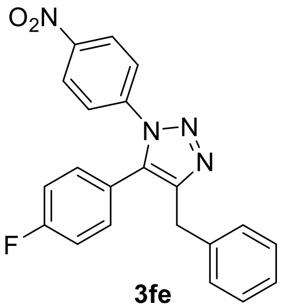

3fe

According to the **General Procedure H**, compound **1f** (46.5 mg, 0.138 mmol, 1.0 eq.), cesium carbonate (90.2 mg, 0.277 mmol, 2.0 eq.), compound **2e** (27.3 mg, 0.166 mmol, 1.2 eq.) were carried out in the reaction. The residue was purified by flash column chromatography on silica gel, eluting with 20% v/v EtOAc in hexane (R_f = 0.48) to give **3fe** as light-yellow oil (48.1 mg, 0.129 mmol, 93% yield).
^{1}H NMR (400 MHz, $CDCl_3$, ppm) δ = 8.25 (d, *J* = 9.1 Hz, 2H), 7.50 (d, *J* = 9.1 Hz, 2H), 7.30-7.19 (m, 5H), 7.13-7.05 (m, 4H), 4.09 (s, 2H); ^{13}C{^{1}H} NMR (100 MHz, $CDCl_3$, ppm) δ = 163.4 (d, $J_{C\text{-}F}$ = 250.1 Hz), 147.3, 145.9, 141.3, 138.7, 133.7, 131.7 (d, $J_{C\text{-}F}$ = 8.4 Hz), 128.7, 128.6, 126.7, 124.9, 124.8, 122.7 (d, $J_{C\text{-}F}$ = 3.2 Hz), 116.7 (d, $J_{C\text{-}F}$ = 21.9 Hz), 31.3; ^{19}F NMR (376 MHz, $CDCl_3$) δ = -109.7; IR (KBr, cm^{-1}) 2925, 1597, 1504, 1347, 1231; HRMS (ESI$^+$) m/z calculated for $(C_{21}H_{15}FN_4O_2+H)^+$: 375.1252, found 375.1242.

4-Benzyl-5-(4-chlorophenyl)-1-(4-nitrophenyl)-1H-1,2,3-triazole (3ge)

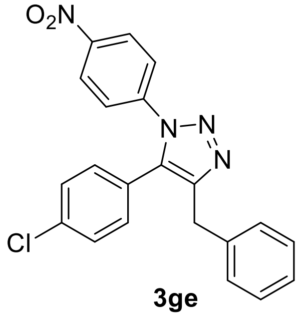

3ge

According to the **General Procedure H**, compound **1g** (44.1 mg, 0.125 mmol, 1.0 eq.), cesium carbonate (81.6 mg, 0.245 mmol, 2.0 eq.), compound **2e** (24.6 mg, 0.147 mmol, 1.2 eq.) were carried out in the reaction. The residue was purified by flash column chromatography on silica gel, eluting with 30% v/v EtOAc in hexane (R_f = 0.60) to give **3ge** as light-yellow solid (48.3 mg, 0.124 mmol, 99% yield).
Melting point = 118.4-119.4 °C; ^{1}H NMR (400 MHz, $CDCl_3$, ppm) δ = 8.26 (d, *J* = 9.3 Hz, 2H), 7.50 (d, *J* = 9.3 Hz, 2H), 7.38 (d, *J* = 8.8 Hz, 2H), 7.30-7.27 (m, 2H), 7.23-7.20 (m, 3H), 7.03 (d, *J* = 8.8 Hz, 2H), 4.09 (s, 2H); ^{13}C{^{1}H} NMR (100 MHz, $CDCl_3$, ppm) δ = 147.4, 146.0, 141.2, 138.7, 136.3, 133.6, 131.0, 129.7, 128.7, 128.6, 126.7, 125.1, 125.0, 124.9. 31.3; IR (KBr, cm^{-1}) 2923, 1598, 1520, 1346, 1093; HRMS (ESI$^+$) m/z calculated for $(C_{21}H_{15}ClN_4O_2+H)^+$: 391.0956, found 391.0954.

4-Benzyl-5-(4-bromophenyl)-1-(4-nitrophenyl)-1H-1,2,3-triazole (3he)

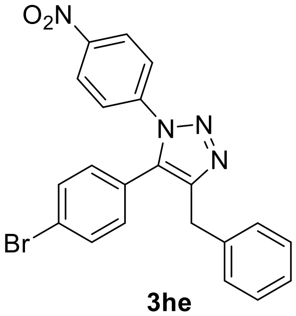

3he

According to the **General Procedure H**, compound **1h** (42.9 mg, 0.108 mmol, 1.0 eq.), cesium carbonate (71.0 mg, 0.216 mmol, 2.0 eq.), compound **2e** (21.6 mg, 0.130 mmol, 1.2 eq.) were carried out in the reaction. The residue was purified by flash column chromatography on silica gel, eluting with 30% v/v EtOAc in hexane (R_f = 0.70) to give **3he** as light-yellow solid (45.8 mg, 0.104 mmol, 96% yield).

Melting point = 144.3-145.1 °C; ^{1}H NMR (400 MHz, $CDCl_3$, ppm) δ = 8.26 (d, J = 9.2 Hz, 2H), 7.54 (d, J = 8.7 Hz, 2H), 7.50 (d, J = 9.3 Hz, 2H), 7.31-7.28 (m, 2H), 7.23-7.20 (m, 3H), 6.96 (d, J = 8.6 Hz, 2H), 4.09 (s, 2H); $^{13}C\{^1H\}$ NMR (100 MHz, $CDCl_3$, ppm) δ = 147.4, 146.0, 141.2, 138.7, 133.6, 132.7, 131.2, 128.8, 128.6, 126.7, 125.6, 125.0, 124.9, 124.5, 31.3; IR (KBr, cm^{-1}) 2924, 1598, 1526, 1346, 1072, 855; HRMS (ESI$^+$) m/z calculated for $(C_{21}H_{15}BrN_4O_2+H)^+$: 435.0451, found 435.0449.

4-Benzyl-1,5-bis(4-nitrophenyl)-1H-1,2,3-triazole (3ie)

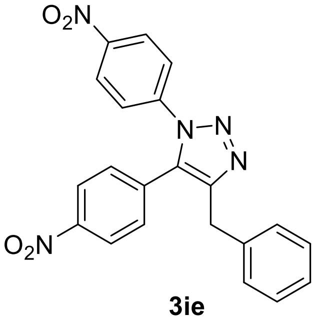

3ie

According to the **General Procedure H**, compound **1i** (35.6 mg, 0.098 mmol, 1.0 eq.), cesium carbonate (63.9 mg, 0.196 mmol, 2.0 eq.), compound **2e** (19.3 mg, 0.118 mmol, 1.2 eq.) were carried out in the reaction. The residue was purified by flash column chromatography on silica gel, eluting with 40% v/v EtOAc in hexane (R_f = 0.58) to give **3ie** as yellow solid (35.3 mg, 0.088 mmol, 90% yield).

Melting point = 137.2-137.8 °C; ^{1}H NMR (400 MHz, $CDCl_3$, ppm) δ = 8.28 (d, J = 9.1 Hz, 2H), 8.24 (d, J = 8.9 Hz, 2H), 7.49 (d, J = 9.1 Hz, 2H), 7.31-7.29 (m, 2H), 7.27 (d, J = 8.9 Hz, 2H), 7.25-7.20 (m, 3H), 4.14 (s, 2H); $^{13}C\{^1H\}$ NMR (100 MHz, $CDCl_3$, ppm) δ = 148.4, 147.7, 146.6, 140.8, 138.2, 133.3, 132.6, 130.7, 128.9, 128.6, 126.9, 125.2, 125.1, 124.4, 31.4; IR (KBr, cm^{-1}) 2956, 1600, 1520, 1349, 1110, 856; HRMS

(ESI$^+$) m/z calculated for $(C_{21}H_{15}N_5O_4+H)^+$: 402.1197, found 402.1195.

4-Benzyl-5-(3-nitrophenyl)-1-(4-nitrophenyl)-1H-1,2,3-triazole (3je)

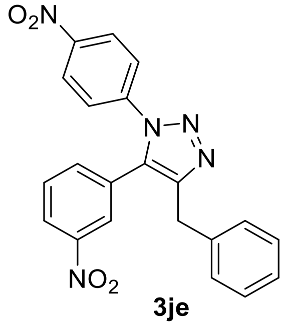

3je

According to the **General Procedure H**, compound **1j** (47.5 mg, 0.1310 mmol, 1.0 eq.), cesium carbonate (85.2 mg, 0.261 mmol, 2.0 eq.), compound **2e** (25.7 mg, 0.118 mmol, 1.2 eq.) were carried out in the reaction. The residue was purified by flash column chromatography on silica gel, eluting with 40% v/v EtOAc in hexane (R_f = 0.60) to give **3je** as yellow solid (47.2 mg, 0.118 mmol, 90% yield).

Melting point = 166.9-167.9 °C; ^{1}H NMR (400 MHz, $CDCl_3$, ppm) δ = 8.30-8.25 (m, 3H), 7.99 (t, J = 1.8 Hz, 1H), 7.57 (t, J = 8.3 Hz, 1H), 7.52-7.48 (m, 2H), 7.34 (ddd, J = 7.7, 1.6, 1.1 Hz, 1H), 7.30-7.19 (m, 5H), 4.15 (s, 2H); $^{13}C\{^1H\}$ NMR (100 MHz, $CDCl_3$, ppm) δ = 148.5, 147.6, 146.6, 140.8, 138.0, 135.4, 132.4, 130.5, 128.8, 128.7, 128.5, 126.9, 125.1, 124.6, 124.5, 31.5; $^{13}C\{^1H\}$ NMR (125 MHz, $CDCl_3$, ppm) δ = 148.5, 147.6, 146.7, 140.8, 138.0, 135.3, 132.2, 130.3, 128.8, 128.6, 128.5, 126.9, 125.1, 125.0, 124.6, 124.5, 31.5;IR (KBr, cm^{-1}) 2933, 1526, 1348, 855, 737; HRMS (ESI$^+$) m/z calculated for $(C_{21}H_{15}N_5O_4+H)^+$: 402.1197, found 402.1192.

4,5-Dimethyl-1-(4-nitrophenyl)-1H-1,2,3-triazole (3ke)

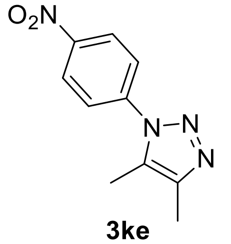

3ke

According to the **General Procedure H**, compound **1k** (21.9 mg, 0.1213 mmol, 1.0 eq.), cesium carbonate (77.8 mg, 0.2388 mmol, 2.0 eq.), compound **2e** (23.6 mg, 0.1438 mmol, 1.2 eq.) were carried out in the reaction. The residue was purified by flash column chromatography on silica gel, eluting with 40% v/v EtOAc in hexane (R_f = 0.25) to give **3ke** as yellow solid (22.6 mg, 0.104 mmol, 85% yield).

Melting point = 208.6-210.0 °C; ^{1}H NMR (400 MHz, $CDCl_3$, ppm) δ = 8.43 (d, J = 9.0 Hz, 2H), 7.73 (d, J = 9.0 Hz, 2H), 2.38 (s, 3H), 2.37 (s, 3H); $^{13}C\{^1H\}$ NMR (100 MHz,

$CDCl_3$, ppm) δ = 147.7, 142.3, 141.8, 129.7, 125.2, 124.9, 10.4, 9.3; IR (KBr, cm^{-1}) 1522, 1341, 1108, 856, 751; HRMS (ESI^+) m/z calculated for $(C_{10}H_{10}N_4O_2 + H)^+$: 219.0877, found 219.0872.

5-Methyl-1-(4-nitrophenyl)-4-pentyl-1H-1,2,3-triazole (3le)

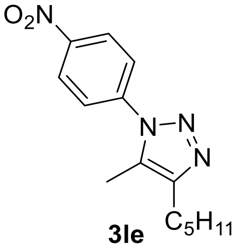

3le

According to the **General Procedure H**, compound **1l** (49.4 mg, 0.187 mmol, 1.0 eq.), cesium carbonate (124.8 mg, 0.383 mmol, 2.0 eq.), compound **2e** (37.1 mg, 0.224 mmol, 1.2 eq.) were carried out in the reaction. The residue was purified by flash column chromatography on silica gel, eluting with 20% v/v EtOAc in hexane (R_f = 0.40) to give **3le** as slight yellow solid (41.0 mg, 0.150 mmol, 80% yield).

Melting point = 97.4-98.2 °C; ^1H NMR (400 MHz, $CDCl_3$, ppm) δ = 8.42 (d, J = 9.1Hz, 2H), 7.76 (d, J = 9.2 Hz, 2H), 2.70 (t, J = 7.6 Hz, 2H), 2.36 (s, 3H), 1.78-1.71 (m, 2H), 1.40-1.37 (m, 4H), 0.94-0.90 (m, 3H); $^{13}C\{^1H\}$ NMR (100 MHz, $CDCl_3$, ppm) δ = 147.5, 146.7, 141.8, 129.4, 125.1, 124.9, 31.6, 29.1, 25.1, 22.5, 14.1, 9.3; IR (KBr, cm^{-1}) 2934, 1527, 1349, 1265, 857, 737; HRMS (ESI^+) m/z calculated for $(C_{14}H_{18}N_4O_2 + H)^+$: 275.1503, found 275.1500.

4-Allyl-5-methyl-1-(4-nitrophenyl)-1H-1,2,3-triazole (3me)

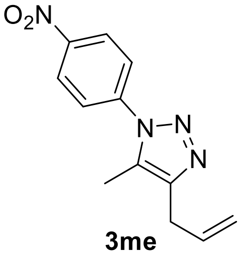

3me

According to the **General Procedure H**, compound **1m** (26.9 mg, 0.128 mmol, 1.0 eq.), cesium carbonate (83.4 mg, 0.256 mmol, 2.0 eq.), compound **2e** (25.2 mg, 0.1535 mmol, 1.2 eq.) were carried out in the reaction. The residue was purified by flash column chromatography on silica gel, eluting with 40% v/v EtOAc in hexane (R_f = 0.60) to give **3me** as yellow solid (29.8 mg, 0.122 mmol, 95% yield).

Melting point =141.0-142.0 °C; ^1H NMR (400 MHz, $CDCl_3$, ppm) δ = 8.40 (d, J = 9.0 Hz, 2H), 7.72 (d, J = 9.0 Hz, 2H), 5.98 (ddt, J = 16.8, 10.3, 6.4 Hz, 1H), 5.15-5.10 (m, 2H), 3.51 (dt, J = 6.4, 1.6 Hz, 2H), 2.35 (s, 3H); $^{13}C\{^1H\}$ NMR (100 MHz, $CDCl_3$, ppm)

δ = 147.7, 144.0, 141.6, 134.6, 130.2, 125.1, 125.1, 116.6, 29.9, 9.3; IR (KBr, cm^{-1}) 3096, 1524, 1349, 1268, 1115, 860, 752; HRMS (ESI^+) m/z calculated for ($C_{12}H_{12}N_4O_2$ +H)$^+$: 245.1033, found 245.1029.

5-Methyl-1-(4-nitrophenyl)-4-(prop-2-yn-1-yl)-1H-1,2,3-triazole (3ne)

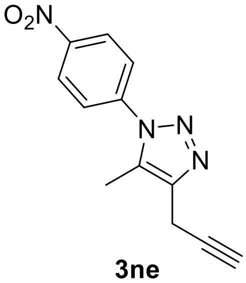

3ne

According to the **General Procedure H**, compound **1n** (24.8 mg, 0.122 mmol, 1.0 eq.), cesium carbonate (80.5 mg, 0.247 mmol, 2.0 eq.), compound **2e** (23.9 mg, 0.146 mmol, 1.2 eq.) were carried out in the reaction. The residue was purified by flash column chromatography on silica gel, eluting with 50% v/v EtOAc in hexane (R_f = 0.60) to give **3ne** brown solid (28.3 mg, 0.117 mmol, 96% yield).

Melting point = 128.5-129.5 °C; ^{1}H NMR (400 MHz, $CDCl_3$, ppm) δ = 8.44 (d, *J* = 9.0 Hz, 2H), 7.73 (d, *J* = 9.0 Hz, 2H), 3.77 (d, *J* = 2.8 Hz, 2H), 2.47 (s, 3H), 2.16 (t, *J* = 2.8 Hz, 1H); $^{13}C\{^1H\}$ NMR (100 MHz, $CDCl_3$, ppm) δ = 147.8, 141.3, 140.7, 130.7, 125.3, 125.1, 79.4, 70.3, 15.9, 9.3; IR (KBr, cm^{-1}) 3287, 3094, 1526, 1506, 1350, 1120, 839, 691; HRMS (ESI^+) m/z calculated for ($C_{12}H_{10}N_4O_2$ +H)$^+$: 243.0877, found 243.0869.

Ethyl 2-(5-methyl-1-(4-nitrophenyl)-1H-1,2,3-triazol-4-yl)acetate (3oe)

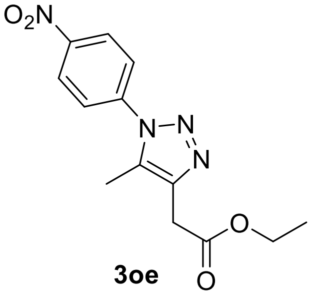

3oe

According to the **General Procedure H**, compound **1o** (26.0 mg, 0.103 mmol, 1.0 eq.), cesium carbonate (66.8 mg, 0.205 mmol, 2.0 eq.), compound **2e** (20.4 mg, 0.124 mmol, 1.2 eq.) were carried out in the reaction. The residue was purified by flash column chromatography on silica gel, eluting with 30% v/v EtOAc in hexane (R_f = 0.30) to give **3oe** as yellow solid (25.9 mg, 0.089 mmol, 87% yield).

Melting point = 78.6-79.6 °C; ^{1}H NMR (400 MHz, $CDCl_3$, ppm) δ = 8.40 (d, *J* = 9.0 Hz, 2H), 7.73 (d, *J* = 9.0 Hz, 2H), 4.18 (q, *J* = 7.1 Hz, 2H), 3.80 (s, 2H), 2.39 (s, 3H),

1.27 (t, J = 7.1 Hz, 3H); $^{13}C\{^1H\}$ NMR (100 MHz, $CDCl_3$, ppm) δ = 169.9, 147.8, 141.4, 139.6, 131.8, 125.2, 125.2, 61.5, 31.6, 14.3, 9.5; IR (KBr, cm^{-1}) 2929, 1754, 1599, 1526, 1506.1, 1346, 1254, 1199, 1029, 856; HRMS (ESI^+) m/z calculated for $(C_{13}H_{14}N_4O_4 + H)^+$: 291.1088, found 291.1083.

***tert*-Butyl 2-(5-methyl-1-(4-nitrophenyl)-1H-1,2,3-triazol-4-yl)acetate (3pe)**

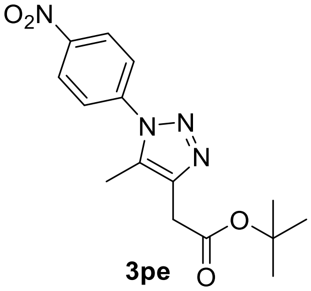

3pe

According to the **General Procedure H**, compound **1p** (42.8 mg, 0.153 mmol, 1.0 eq.), cesium carbonate (99.2 mg, 0.305 mmol, 2.0 eq.), compound **2e** (31.8 mg, 0.194 mmol, 1.2 eq.) were carried out in the reaction. The residue was purified by flash column chromatography on silica gel, eluting with 40% v/v EtOAc in hexane (R_f = 0.60) to give **3pe** as yellow solid (42.8 mg, 0.141 mmol, 92% yield).

Melting point = 123.9-124.9 °C; ^1H NMR (400 MHz, $CDCl_3$, ppm) δ = 8.43 (d, J = 8.8 Hz, 2H), 7.74 (d, J = 8.8 Hz, 2H), 3.75 (s, 2H), 2.40 (s, 3H), 1.49 (s, 9H); $^{13}C\{^1H\}$ NMR (100 MHz, $CDCl_3$, ppm) δ = 169.2, 147.8, 141.5, 140.1, 131.7, 125.2, 125.1, 81.8, 32.7, 28.2, 9.5; IR (KBr, cm^{-1}) 3097, 2983, 1733, 1596, 1522, 1346, 1255, 1226, 1155, 864; HRMS (ESI^+) m/z calculated for $(C_{15}H_{18}N_4O_4 + H)^+$: 319.1401, found 319.1376.

5-Methyl-1-(4-nitrophenyl)-4-phenyl-1H-1,2,3-triazole (3qe)

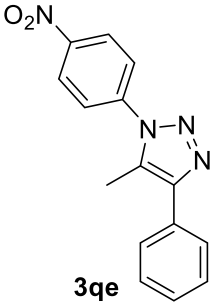

3qe

According to the **General Procedure H**, compound **1q** (30.6 mg, 0.126 mmol, 1.0 eq.), cesium carbonate (82.3 mg, 0.253 mmol, 2.0 eq.), compound **2e** (24.9 mg, 0.152 mmol, 1.2 eq.) were carried out in the reaction. The residue was purified by flash column chromatography on silica gel, eluting with 20% v/v EtOAc in hexane (R_f = 0.35) to give **3qe** as yellow solid (33.0 mg, 0.118 mmol, 93% yield).

Melting point = 142.3-143.0 °C; ^1H NMR (400 MHz, $CDCl_3$, ppm) δ = 8.47 (d, J = 9.1

Hz, 2H), 7.81-7.75 (m, 4H), 7.53-7.48 (m, 2H), 7.44-7.40 (m, 1H), 2.58 (s, 3H). All spectroscopic data were in agreement with the literature values.[26]

4-Methyl-1-(4-nitrophenyl)-5-phenyl-1H-1,2,3-triazole (3re)

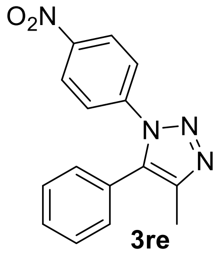

3re

According to the **General Procedure H**, compound **1r** (30.2 mg, 0.1247 mmol, 1.0 eq.), cesium carbonate (83.3 mg, 0.2557 mmol, 2.0 eq.), compound **2e** (26.2 mg, 0.1596 mmol, 1.2 eq.) were carried out in the reaction. The residue was purified by flash column chromatography on silica gel, eluting with 40% v/v EtOAc in hexane (R_f = 0.60) to give **3re** as yellow solid (31.9 mg, 0.1138 mmol, 91% yield).

Melting point = 141.0-142.0 °C; ^{1}H NMR (400 MHz, $CDCl_3$, ppm) δ = 8.24 (d, J = 8.8 Hz, 2H), 7.51 (d, J = 8.8 Hz, 2H), 7.47-7.41 (m, 3H), 7.20-7.17 (m, 2H), 2.43 (s, 3H); $^{13}C\{^1H\}$ NMR (100 MHz, $CDCl_3$, ppm) δ = 147.3, 142.9, 141.7, 134.2, 129.7, 129.5, 129.4, 127.0, 124.8, 10.8; IR (KBr, cm^{-1}) 2926, 1598, 1523, 1502, 1350, 1116, 854, 749; HRMS (ESI^+) m/z calculated for $(C_{15}H_{12}N_4O_2 + H)^+$: 281.1033, found 281.1026. Note: Due to the overlap of signals, the observed peaks have been reported accordingly.

1-(4-Nitrophenyl)-1,4,5,6-tetrahydrocyclopenta[d][1,2,3]triazole (3se)

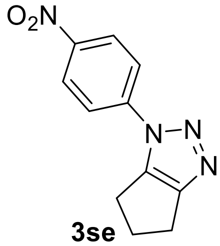

3se

According to the **General Procedure H**, compound **1s** (110.2 mg, 0.500 mmol, 1.0 eq.), cesium carbonate (326.1 mg, 1.001 mmol, 2.0 eq.), compound **2e** (98.6 mg, 0.601 mmol, 1.2 eq.) were carried out in the reaction. The residue was purified by flash column chromatography on silica gel, eluting with 40% v/v EtOAc in hexane (R_f = 0.45) to give **3se** as yellow solid (103.0 mg, 0.447 mmol, 89% yield).

Melting point = 160.5-161.5 °C; ^{1}H NMR (400 MHz, $CDCl_3$, ppm) δ = 8.39 (d, J = 9.2 Hz, 2H), 7.93 (d, J = 9.2 Hz, 2H), 3.11-3.06 (m, 2H), 2.92-2.87 (m, 2H), 2.84-2.76 (m, 2H); $^{13}C\{^1H\}$ NMR (125 MHz, $CDCl_3$, ppm) δ = 158.0, 146.7, 141.9, 141.1, 125.6,

119.7, 30.4, 24.3, 22.4; IR (KBr, cm^{-1}) 2914, 1598, 1506, 1336, 1220, 1038, 854, 748; HRMS (ESI^+) m/z calculated for $(C_{11}H_{10}N_4O_2 + H)^+$: 231.0877, found 231.0872.

1-(4-Nitrophenyl)-4,5,6,7-tetrahydro-1H-benzo[d][1,2,3]triazole (3te)

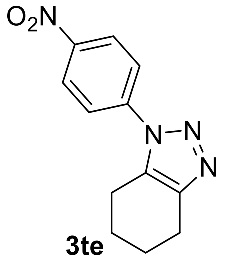

3te

According to the **General Procedure H**, compound **1t** (48.6 mg, 0.200 mmol, 1.0 eq.), cesium carbonate (130.3 mg, 0.400 mmol, 2.0 eq.), compound **2e** (39.4 mg, 0.240 mmol, 1.2 eq.) were carried out in the reaction. The residue was purified by flash column chromatography on silica gel, eluting with 40% v/v EtOAc in hexane (R_f = 0.45) to give **3te** as yellow solid (44.7 mg, 0.183 mmol, 92% yield).

Melting point = 221.5-222.5 °C; ^{1}H NMR (400 MHz, $CDCl_3$, ppm) δ = 8.40 (d, J = 9.2 Hz, 2H), 7.83 (d, J = 9.1 Hz, 2H), 2.87-2.83 (m, 4H), 1.93-1.90 (m, 4H); $^{13}C\{^1H\}$ NMR (125 MHz, $CDCl_3$, ppm) δ = 147.1, 145.2, 141.8, 132.3, 125.3, 122.8, 22.8, 22.5, 22.3, 22.0; IR (KBr, cm^{-1}) 2944, 1596, 1522, 1344, 1119, 854, 750; HRMS (ESI^+) m/z calculated for $(C_{12}H_{12}N_4O_2 + H)^+$: 245.1033, found 245.1028.

1-(4-Nitrophenyl)-1,4,5,6,7,8-hexahydrocyclohepta[d][1,2,3]triazole (3ue)

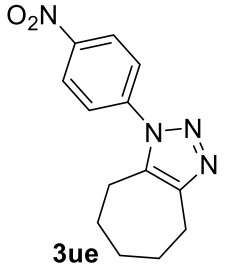

3ue

According to the **General Procedure H**, compound **1u** (86.4 mg, 0.348 mmol, 1.0 eq.), cesium carbonate (226.8 mg, 0.696 mmol, 2.0 eq.), compound **2e** (68.5 mg, 0.418 mmol, 1.2 eq.) were carried out in the reaction. The residue was purified by flash column chromatography on silica gel, eluting with 40% v/v EtOAc in hexane (R_f = 0.45) to give **3ue** as yellow solid (78.2 mg, 0.303 mmol, 87% yield).

Melting point = 165.3-166.1 °C; ^{1}H NMR (400 MHz, $CDCl_3$, ppm) δ = 8.42 (d, J = 9.0 Hz, 2H), 7.66 (d, J = 9.3 Hz, 2H), 3.00-2.98 (m, 2H), 2.82-2.79 (m, 2H), 1.93-1.88 (m,

2H), 1.82-1.72 (m, 4H); $^{13}C\{^1H\}$ NMR (125 MHz, $CDCl_3$, ppm) δ = 148.3, 147.6, 141.4, 135.7, 125.7, 124.9, 30.7, 27.1, 27.0, 26.8, 24.9; IR (KBr, cm^{-1}) 2926, 1596, 1520, 1350, 1252.6, 1108, 864; HRMS (ESI^+) m/z calculated for $(C_{13}H_{14}N_4O_2 + H)^+$: 259.1190, found 259.1183.

4-Benzyl-1-(4-nitrophenyl)-1H-1,2,3-triazole (3ve)

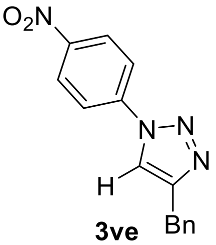

3ve

According to the **General Procedure H**, compound **1v** (46.9 mg, 0.174 mmol, 1.0 eq.), cesium carbonate (113.4 mg, 0.348 mmol, 2.0 eq.), compound **2e** (34.2 mg, 0.209 mmol, 1.2 eq.) were carried out in the reaction. The residue was purified by flash column chromatography on silica gel, eluting with 40% v/v EtOAc in hexane (R_f = 0.75) to give **3ve** as slight yellow solid (47.3 mg, 0.169 mmol, 97% yield).

^1H NMR (400 MHz, $CDCl_3$, ppm) δ = 8.38 (d, J = 9.2 Hz, 2H), 7.92 (d, J = 9.2 Hz, 2H), 7.68 (s, 1H), 7.37-7.25 (m, 5H), 4.19 (s, 2H). All spectroscopic data were in agreement with the literature values.[27]

1-(4-Nitrophenyl)-4-pentyl-1H-1,2,3-triazole (3we)

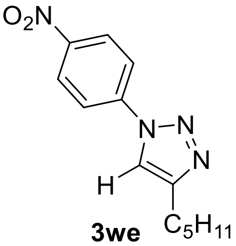

3we

According to the **General Procedure H**, compound **1w** (45.9 mg, 0.183 mmol, 1.0 eq.), cesium carbonate (119.3 mg, 0.366 mmol, 2.0 eq.), compound **2e** (36.0 mg, 0.220 mmol, 1.2 eq.) were carried out in the reaction. The residue was purified by flash column chromatography on silica gel, eluting with 30% v/v EtOAc in hexane (R_f = 0.75) to give **3we** as slight yellow solid (45.1 mg, 0.173 mmol, 94% yield).

^1H NMR (400 MHz, $CDCl_3$, ppm) δ = 8.41 (d, J = 9.1 Hz, 2H), 7.96 (d, J = 9.1 Hz, 2H), 7.82 (s, 1H), 2.82 (t, J = 7.7 Hz, 2H), 1.79-1.72 (m, 2H), 1.41-1.38 (m, 4H), 0.92 (t, J = 7.1 Hz, 3H). All spectroscopic data were in agreement with the literature values.[27]

1-(4-Nitrophenyl)-4-phenyl-*1H*-1,2,3-triazole (3xe)

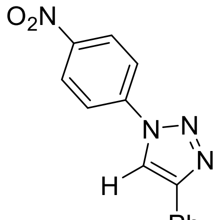

3xe

According to the **General Procedure H**, compound **1x** (19.9 mg, 0.0873 mmol, 1.0 eq.), cesium carbonate (56.9 mg, 0.175 mmol, 2.0 eq.), compound **2e** (17.2 mg, 0.105 mmol, 1.2 eq.) were carried out in the reaction. The residue was purified by flash column chromatography on silica gel, eluting with 30% v/v EtOAc in hexane (R_f = 0.75) to give **3xe** as yellow solid (19.7 mg, 0.0740 mmol, 85%).

^{1}H NMR (400 MHz, DMSO-d_6, ppm) δ = 9.53 (s, 1H), 8.51 (d, *J* = 9.1 Hz, 2H), 8.28 (d, *J* = 9.2 Hz, 2H), 7.97 (d, *J* = 8.0 Hz, 2H), 7.56-7.48 (m, 2H), 7.44-7.38 (m, 1H). All spectroscopic data were in agreement with the literature values.[27a]

we also synthesized triazole isomer **3xe'** from phosphonate isomer **1x'** for comparison, revealing distinct chemical shifts on the triazole ring due to different Ph groups: 9.53 ppm for **3xe** and 8.19 ppm for **3xe'**.

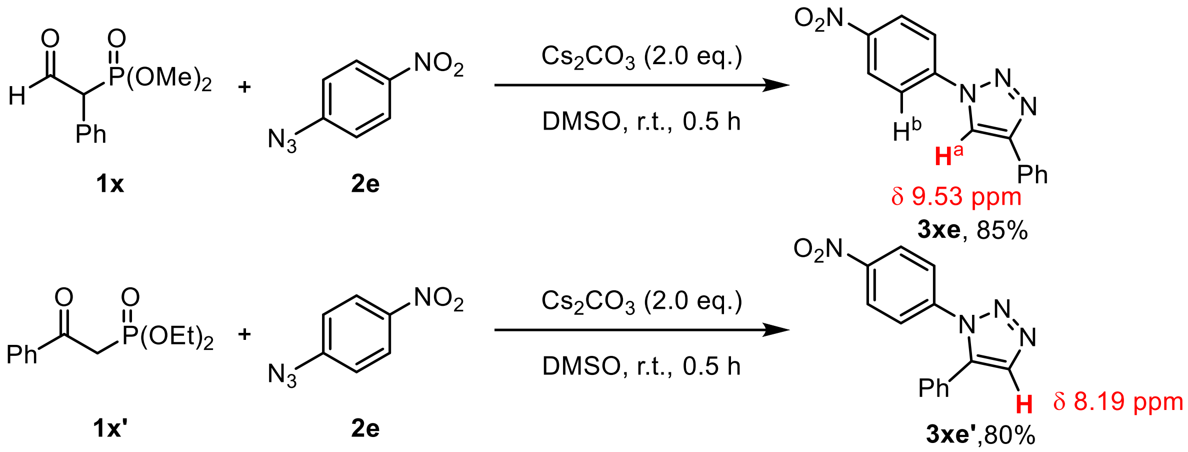

1-(4-Nitrophenyl)-5-phenyl-*1H*-1,2,3-triazole 3xe'

^{1}H NMR (400 MHz, DMSO-d_6, ppm) δ = 8.37 (d, *J* = 9.0 Hz, 2H), 8.19 (s, 1H), 7.70 (d, *J* = 9.2 Hz, 2H), 7.45-7.42 (m, 3H), 7.35-7.32 (m, 2H). ^{1}H NMR (400 MHz, $CDCl_3$, ppm) δ = 8.30 (d, *J* = 9.0 Hz, 2H), 7.89 (s, 1H), 7.59 (d, *J* = 9.0 Hz, 2H), 7.48-7.40 (m, 3H), 7.26-7.23 (m, 2H). All spectroscopic data were in agreement with the literature values[27b].

4-Fluoro-5-methyl-1-(4-nitrophenyl)-1H-1,2,3-triazole (3ye)

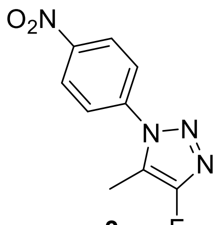

3ye

According to the **General Procedure H**, compound **1y** (15.9 mg, 0.086 mmol, 1.0 eq.), cesium carbonate (58.8 mg, 0.181 mmol, 2.0 eq.), compound **2e** (17.8 mg, 0.109 mmol, 1.2 eq.) were carried out in the reaction. The residue was purified by flash column chromatography on silica gel, eluting with 40% v/v EtOAc in hexane (R_f= 0.75) to give **3ye** as yellow solid (6.1 mg, 0.0275 mmol, 32% yield).

Melting point = 142.3-143.0 °C; ^{1}H NMR (500 MHz, $CDCl_3$, ppm) δ = 8.46 (d, J = 9.2 Hz, 2H), 7.76 (d, J = 9.2 Hz, 2H), 2.42 (d, J_{H-F} = 1.2 Hz, 3H); $^{13}C\{^1H\}$ NMR (100 MHz, $CDCl_3$, ppm) δ = 160.1 (d, J_{C-F} = 243.4 Hz), 148.2, 141.3, 125.4, 124.8, 115.8 (d, J_{C-F} = 32.3 Hz), 8.1; ^{19}F NMR (470 MHz, $CDCl_3$, ppm) δ = -147.3; IR (KBr, cm^{-1}) 2919, 1608, 1529, 1350, 1266, 1126, 862, 750; HRMS (ESI$^+$) m/z calculated for $(C_9H_7FN_4O_2+H)^+$: 223.0626, found 223.0609.

4-Fluoro-1-(4-nitrophenyl)-5-phenyl-1H-1,2,3-triazole (3ze)

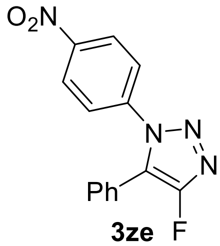

3ze

According to the **General Procedure H**, compound **1z** (52.4 mg, 0.191 mmol, 1.0 eq.), cesium carbonate (124.5 mg, 0.382 mmol, 2.0 eq.), compound **2e** (37.6 mg, 0.229 mmol, 1.2 eq.) were carried out in the reaction. The residue was purified by flash column chromatography on silica gel, eluting with 20% v/v EtOAc in hexane (R_f= 0.50) to give **3ze** as yellow solid (36.0 mg, 0.127 mmol, 75%).

Melting point = 150.0-151.0 °C; ^{1}H NMR (400 MHz, $CDCl_3$, ppm) δ = 8.32 (d, J = 9.2 Hz, 2H), 7.60 (d, J = 9.3 Hz, 2H), 7.50-7.42 (m, 3H), 7.28 -7.24 (m, 2H); $^{13}C\{^1H\}$ NMR (125 MHz, $CDCl_3$, ppm) δ = 158.9 (d, J_{C-F} = 247.7 Hz), 148.0, 141.3, 130.3, 129.5, 128.9, 125.3, 125.1, 123.7 (d, J_{C-F} = 4.0 Hz), 120.1 (d, J_{C-F} = 29.0 Hz); ^{19}F NMR (658

MHz, $CDCl_3$, ppm) δ = -144.0; IR (KBr, cm^{-1}) 3124, 1596, 1522, 1351, 1232, 1124, 990, 857, 753; HRMS (ESI^+) m/z calculated for $(C_{14}H_9FN_4O_2 + H)^+$: 285.0782, found 285.0779.

5-Methyl-1-phenyl-1H-1,2,3-triazole (5aa)

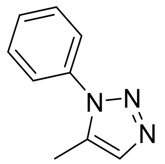

5aa

According to the **General Procedure H**, compound **4a** (44.5 mg, 0.268 mmol, 1.0 eq.), cesium carbonate (174.6 mg, 0.536 mmol, 2.0 eq.), compound **2a** (38.3 mg, 0.322 mmol, 1.2 eq.) were carried out in the reaction for 2 h. The residue was purified by flash column chromatography on silica gel, eluting with 100% EtOAc in hexane (R_f = 0.80) to give **5aa** as yellow liquid (37.3 mg, 0.234 mmol, 87% yield).
^{1}H NMR (400 MHz, $CDCl_3$, ppm) δ = 7.58-7.46 (m, 6H), 2.35 (s, 3H). All spectroscopic data were in agreement with the literature values.[28]

1-(4-Chlorophenyl)-5-methyl-1H-1,2,3-triazole (5ab)

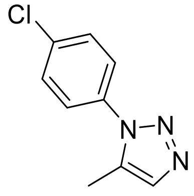

5ab

According to the **General Procedure H**, compound **4a** (64.4 mg, 0.388 mmol, 1.0 eq.), cesium carbonate (252.8 mg, 0.776 mmol, 2.0 eq.), compound **2b** (71.5 mg, 0.466 mmol, 1.2 eq.) were carried out in the reaction for 0.5 h. The residue was purified by flash column chromatography on silica gel, eluting with 40% v/v EtOAc in hexane (R_f = 0.45) to give **5ab** as white solid (68.3 mg, 0.353 mmol, 91% yield).
^{1}H NMR (400 MHz, $CDCl_3$, ppm) δ = 7.58 (d, J = 0.8 Hz, 1H), 7.55-7.51 (m, 2H), 7.45-7.41 (m, 2H), 2.35 (d, J = 0.8 Hz, 3H). All spectroscopic data were in agreement with the literature values.[29]

1-(4-Bromophenyl)-5-methyl-1H-1,2,3-triazole (5ac)

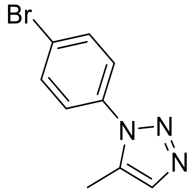

5ac

According to the **General Procedure H**, compound **4a** (67.7 mg, 0.408 mmol, 1.0 eq.), cesium carbonate (265.9 mg, 0.816 mmol, 2.0 eq.), compound **2c** (97.0 mg, 0.490 mmol, 1.2 eq.) were carried out in the reaction for 2 h. The residue was purified by flash column chromatography on silica gel, eluting with 40% v/v EtOAc in hexane (R_f = 0.45) to give **5ac** as white solid (87.7 mg, 0.368 mmol, 90% yield).

^{1}H NMR (400 MHz, $CDCl_3$, ppm) δ = 7.70-7.66 (m, 2H), 7.58 (brs, 1H), 7.39-7.35 (m, 2H), 2.35 (d, J = 0.8 Hz, 3H). All spectroscopic data were in agreement with the literature values.[30]

1-(4-Methoxyphenyl)-5-methyl-1H-1,2,3-triazole (5ad)

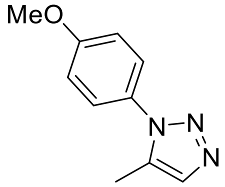

5ad

According to the **General Procedure H**, compound **4a** (53.0 mg, 0.319 mmol, 1.0 eq.), cesium carbonate (207.9 mg, 0.638 mmol, 2.0 eq.), compound **2d** (57.1 mg, 0.383 mmol, 1.2 eq.) were carried out in the reaction for 24 h. The residue was purified by flash column chromatography on silica gel, eluting with 30% v/v EtOAc in hexane to give **5ad** (R_f = 0.15) as black oil (49.0 mg, 0.259 mmol, 81% yield).

^{1}H NMR (400 MHz, $CDCl_3$, ppm) δ = 7.56 (brs, 1H), 7.40-7.34 (m, 2H), 7.05-7.03 (m, 2H), 3.88 (s, 3H), 2.31 (d, J = 0.8 Hz, 3H). All spectroscopic data were in agreement with the literature values.[29]

5-Methyl-1-(4-nitrophenyl)-1H-1,2,3-triazole (5ae) and dimethyl (5-methyl-1-(4-nitrophenyl)-1H-1,2,3-triazol-4-yl)phosphonate (5ae')

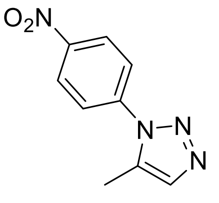

5ae

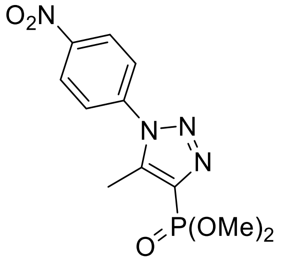

5ae'

According to the **General Procedure H**, compound **4a** (80.3 mg, 0.483 mmol, 1.0 eq.), cesium carbonate (314.9 mg, 0.966 mmol, 2.0 eq.), compound **2e** (95.0 mg, 0.579 mmol, 1.2 eq.) were carried out in the reaction for 2 h. The residue was purified by flash column chromatography on silica gel, eluting with 80% v/v EtOAc in hexane to give either **5ae** (R_f = 0.70; 70.0 mg, 0.343 mmol, 71% yield) or **5ae'** (R_f = 0.40; 4.5 mg, 0.014 mmol, 3% yield) as yellow solid.

Compound **5ae:** ^{1}H NMR (400 MHz, $CDCl_3$, ppm) δ = 8.46-8.43 (m, 2H), 7.77-7.74 (m, 2H), 7.64 (brs, 1H), 2.46 (d, *J* = 0.8 Hz, 3H). All spectroscopic data were in agreement with the literature values.[29]

Compound **5ae':** ^{1}H NMR (400 MHz, DMSO-d_6, ppm) δ = 8.50-8.46 (m, 2H), 8.03-7.99 (m, 2H), 3.77 (d, *J* = 11.4 Hz, 6H), 2.55 (d, *J* = 1.5 Hz, 3H). All spectroscopic data were in agreement with the literature values.[31]

5 SC-XRD Structure analysis

All manipulations were performed under a nitrogen atmosphere with a Vigor glove box or Schlenk techniques.

A. Preparation of crystal structure of cesium enolate 6

A 50-mL Schlenk flask equipped with a Teflon-coated magnetic stir bar was charged with 0.3385 g (1.321 mmol) **1a**, 0.6456 g (1.982 mmol, 1.5 equiv.) Cs_2CO_3, and DMF (10 mL) under nitrogen atmosphere. The reaction mixture was allowed to stir overnight at room temperature, resulting in yellow solution accompanied with white solid. Collecting the upper layer through filtration under nitrogen and all volatile materials were removed under reduced pressure. The residue was dissolved in THF (5 mL) and layered with hexane (5 mL), from which colorless crystals of **6** suitable for single-crystal X-ray diffraction were grown from that solution in 1 day at room temperature.

B. Preparation of crystal structure of cesium enolate 7

A 50-mL Schlenk flask equipped with a Teflon-coated magnetic stir bar was charged with 0.2102 g (1.2656 mmol) **4a**, 0.6185 g (1.898 mmol, 1.5 equiv.) Cs_2CO_3, and DMF (8.5 mL) under nitrogen atmosphere. The reaction mixture was allowed to stir overnight at room temperature, resulting in yellow solution accompanied with white solid.

Collecting the upper layer through filtration and all volatile materials were removed under reduced pressure. The residue was dissolved in DCM (10 mL) and layered with Et2O (10 mL), from which colorless crystals of **7** suitable for single-crystal X-ray diffraction were grown from that solution in 1 day at room temperature.

C. SC-XRD Structural Analysis

The suitable crystals were analyzed using a Bruker D8 Venture PHOTON III single-crystal diffractometer with an *I*µS 3.0 microfocus X-ray source Mo Kα radiation (λ = 0.71073 Å) at National Cheng Kung University's Core Facility Center. Data collection and integration were performed using Bruker *APEX3* software, with absorption corrections made through the program *SADABS*. The structures were solved using the *SHELXT* program and subsequently refined using the program *SHELXL*.

6 Reference

1. M. Shankar, H. R. Mohan, U. V. Prasad, M. H. Krishna, P. M. Rao, T. Lakshmikumar and G. V. Subbaraju, *Asian J. Chem.*, **2013**, *25*, 913-920.
2. B. J. Lundy, S. Jansone-Popova and J. A. May, *Org. Lett.*, **2011**, *13*, 4958-4961.
3. G.-Y. Zhang, C.-K. Li, D.-P. Li, R.-S. Zeng, A. Shoberu and J.-P. Zou, *Tetrahedron*, **2016**, *72*, 2972-2978.
4. F. M. Moghaddam, M. Daneshfar, R. Azaryan and J.-L. Pirat, *Catal. Commun.*, **2020**, *141*, 106015-106021.
5. R. Bodalski, T. J. Michalski and J. Monkiewicz, *Phosphorus, Sulfur Relat. Elem.*, **1980**, *9*, 121-122.
6. M. Sacristán, J. C. Ronda, M. Galià and V. Cádiz, *J. Appl. Polym. Sci.*, **2011**, *122*, 1649-1658.
7. S. M. A. Kedrowski and D. A. Dougherty, *Org. Lett.*, **2010**, *12*, 3990-3993.
8. X. Tao, W. Li, X. Ma, X. Li, W. Fan, L. Zhu, X. Xie and Z. Zhang, *J. Org. Chem.*, **2012**, *77*, 8401-8409.
9. B. Schilling, W. D. Woggon, A. Chougnet, T. Granier, G. Frater and A. Hanhart, WO Pat., 116339, 2008.
10. M. Murai, M. Nakamura and K. Takai, *Org. Lett.*, **2014**, *16*, 5784-5787.
11. X.-H. Hu, X.-F. Yang and T.-P. Loh, *Angew. Chem. Int. Ed.*, **2015**, *54*, 15535-15539.
12. T. Calogeropoulou, G. B. Hammond and D. F. Wiemer, *J. Org. Chem.*, **1987**, *52*, 4185-4190.
13. L. Li, W. Huang, L. Chen, J. Dong, X. Ma and Y. Peng, *Angew. Chem. Int. Ed.*, **2017**, *56*, 10539-10544.
14. E. J. Emmett, B. R. Hayter and M. C. Willis, *Angew. Chem. Int. Ed.*, **2014**, *53*, 10204-10208.

15. K. Lee and D. F. Wiemer, *J. Org. Chem.*, **1991**, *56*, 5556-5560.
16. J. M. Gil, J. H. Hah, K. Y. Park and D. Y. Oh, *Synth. Commun.*, **2000**, *30*, 789-794.
17. Y. Zanella, S. Berté-Verrando, R. Dizière and P. Savignac, *J. Chem. Soc., Perkin Trans. 1*, **1995**, *22*, 2835-2838.
18. M. Yamashita, H. Nomoto and H. Imoto, *Synthesis*, **1987**, *8*, 716-718.
19. R. D. Chambers and J. Hutchinson, *J. Fluor. Chem.*, **1998**, *92*, 45-52.
20. K. V. Tarasenko, V. D. Romanenko and A. E. Sorochinsky, *J. Fluor. Chem.*, **2018**, *211*, 124-128.
21. M. Li, N. Zheng, J. Li, Y. Zheng and W. Song, *Green Chem.*, 2020, **22**, 2394-2398.
22. F. Alonso, Y. Moglie, G. Radivoy and M. Yus, *Eur. J. Org. Chem.*, **2010**, *2010*, 1875-1884.
23. M. Kitamura, M. Yano, N. Tashiro, S. Miyagawa, M. Sando and T. Okauchi, *Eur. J. Org. Chem.*, **2011**, *2011*, 458-462.
24. H. Yang, Y. Li, M. Jiang, J. Wang and H. Fu, *Chem. Eur. J.*, **2011**, *17*, 5652-5660.
25. J. Waser, B. Gaspar, H. Nambu and E. M. Carreira, *J. Am. Chem. Soc.*, **2006**, *128*, 11693-11712.
26. A. B. Shashank, S. Karthik, R. Madhavachary and D. B. Ramachary, *Chemistry*, **2014**, *20*, 16877-16881.
27. a) D. B. Ramachary, A. B. Shashank and S. Karthik, *Angew. Chem. Int. Ed.*, **2014**, *53*, 10420-10424. b) X. Zhang, K. P. Rakesh and H,-L Qin, *Chem. Commun.* **2019**, *55,* 2845-2848.
28. P. R. Clark, G. D. Williams, J. F. Hayes and N. C. O. Tomkinson, *Angew. Chem. Int. Ed.*, **2020**, *59*, 6740-6744.
29. A. H. Banday and V. J. Hruby, *Synlett*, **2014**, *25*, 1859-1862.
30. H.-S. Dong, H.-R. Dong and T.-Q. Zhang, *J. Chem. Crystallogr.*, **2009**, *39*, 32-35.
31. N. T. Pokhodylo, O. Y. Shyyka, E. A. Goreshnik and M. D. Obushak, *ChemistrySelect*, **2020**, *5*, 260-264

7 NMR spectra

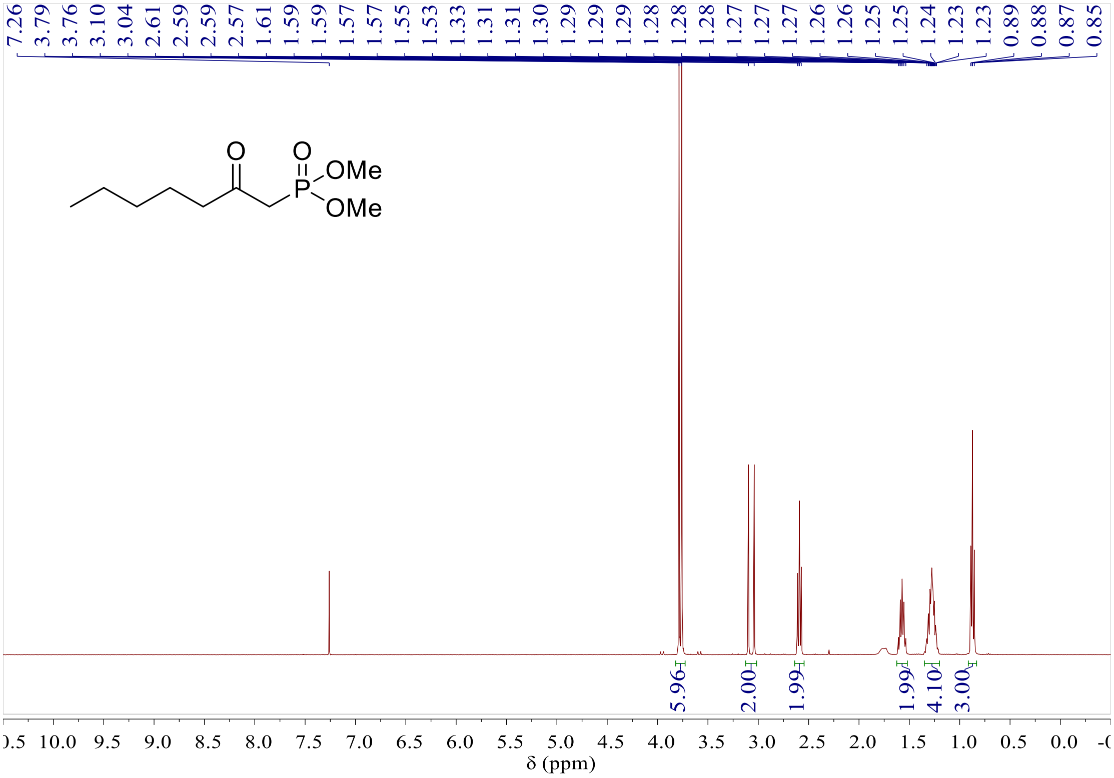

^{1}H NMR spectrum of **4b** in $CDCl_3$ (400 MHz)

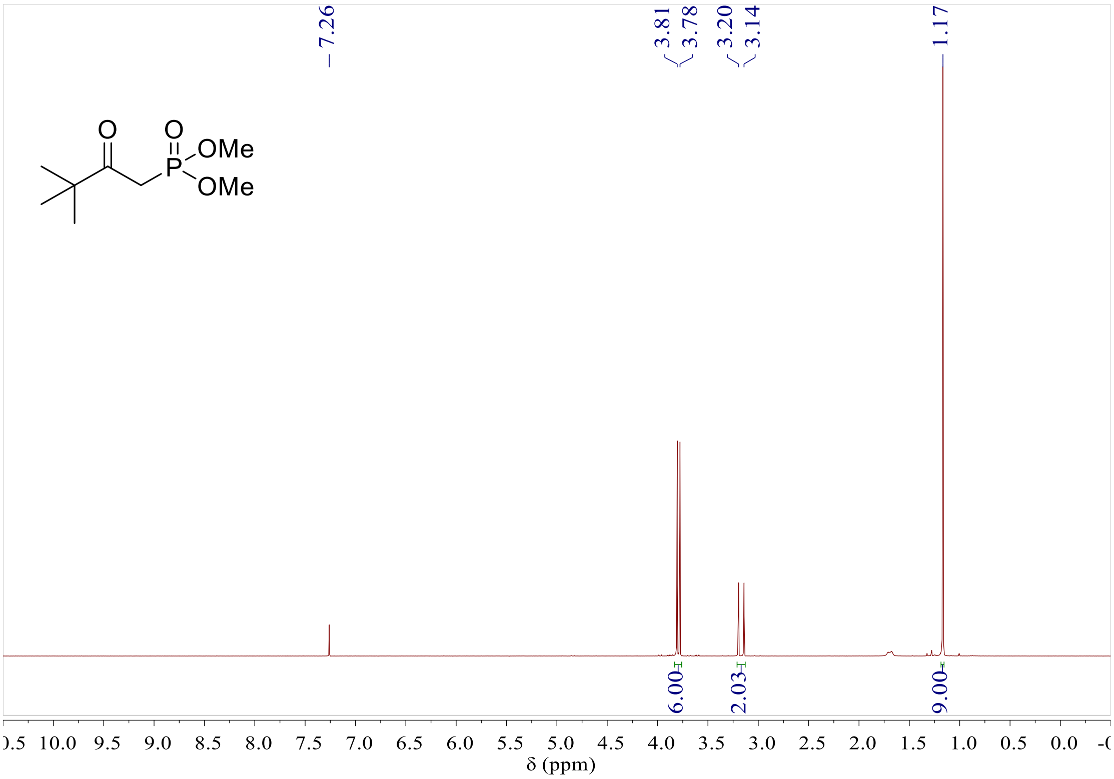

^{1}H NMR spectrum of **4c** in $CDCl_3$ (400 MHz)

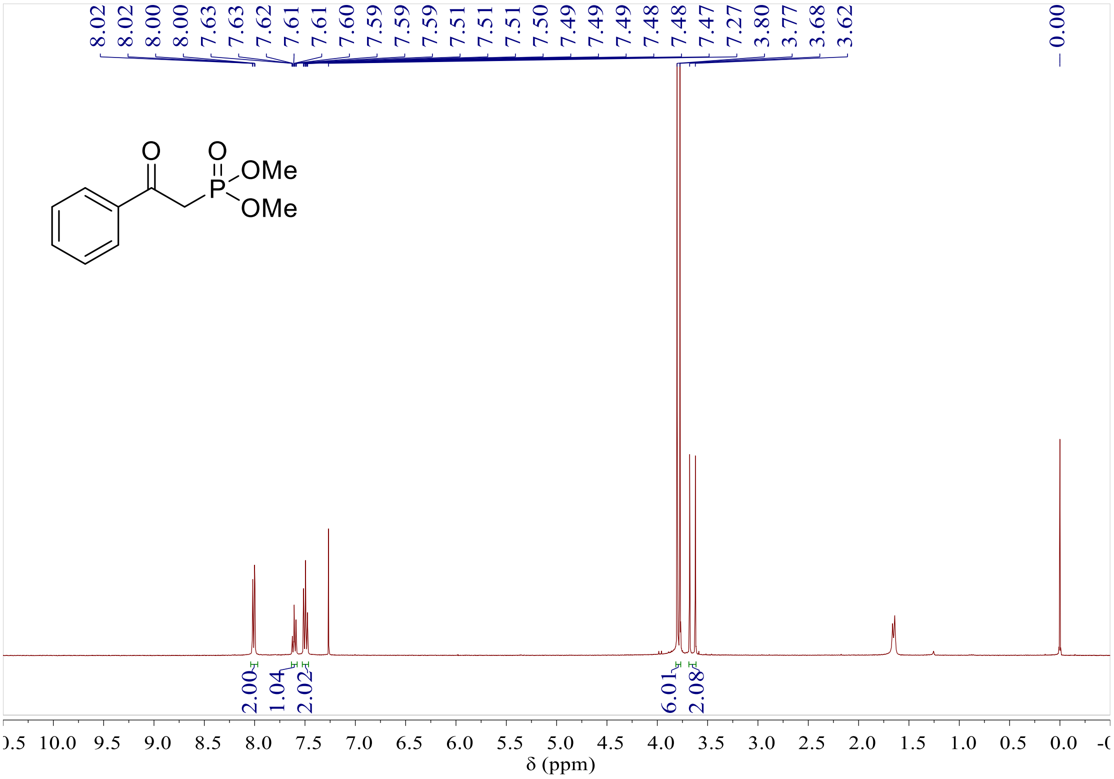

^{1}H NMR spectrum of **4d** in $CDCl_3$ (400 MHz)

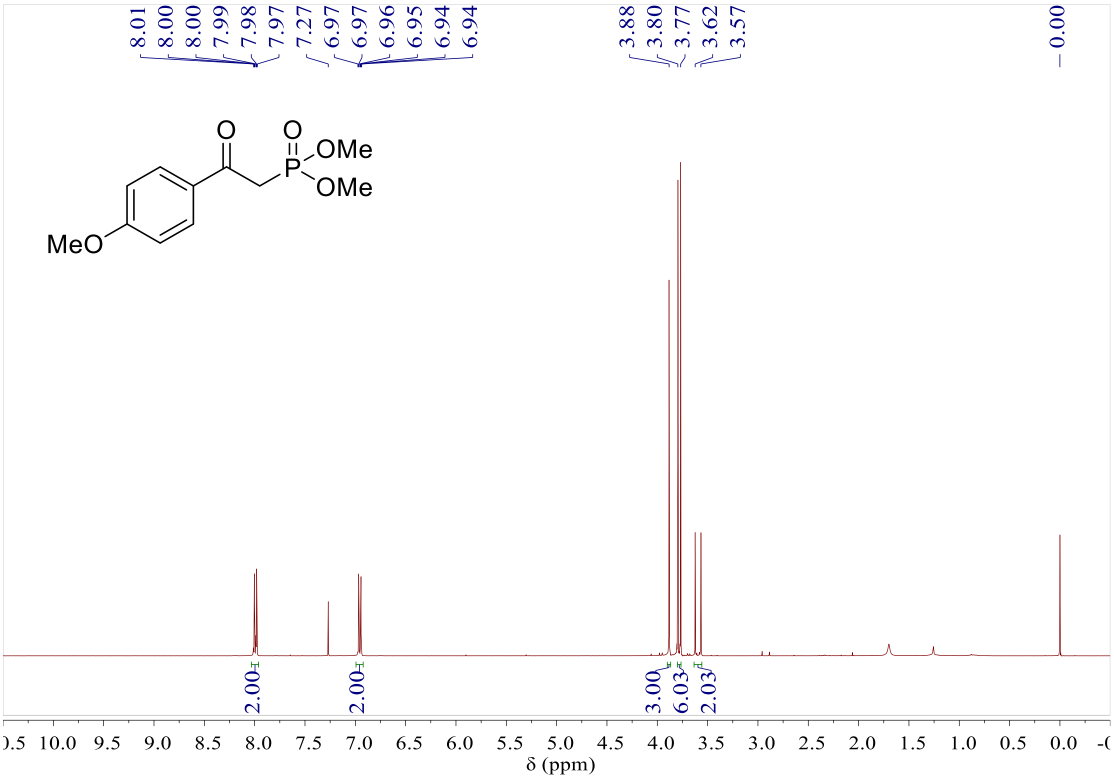

^{1}H NMR spectrum of **4e** in $CDCl_3$ (400 MHz)

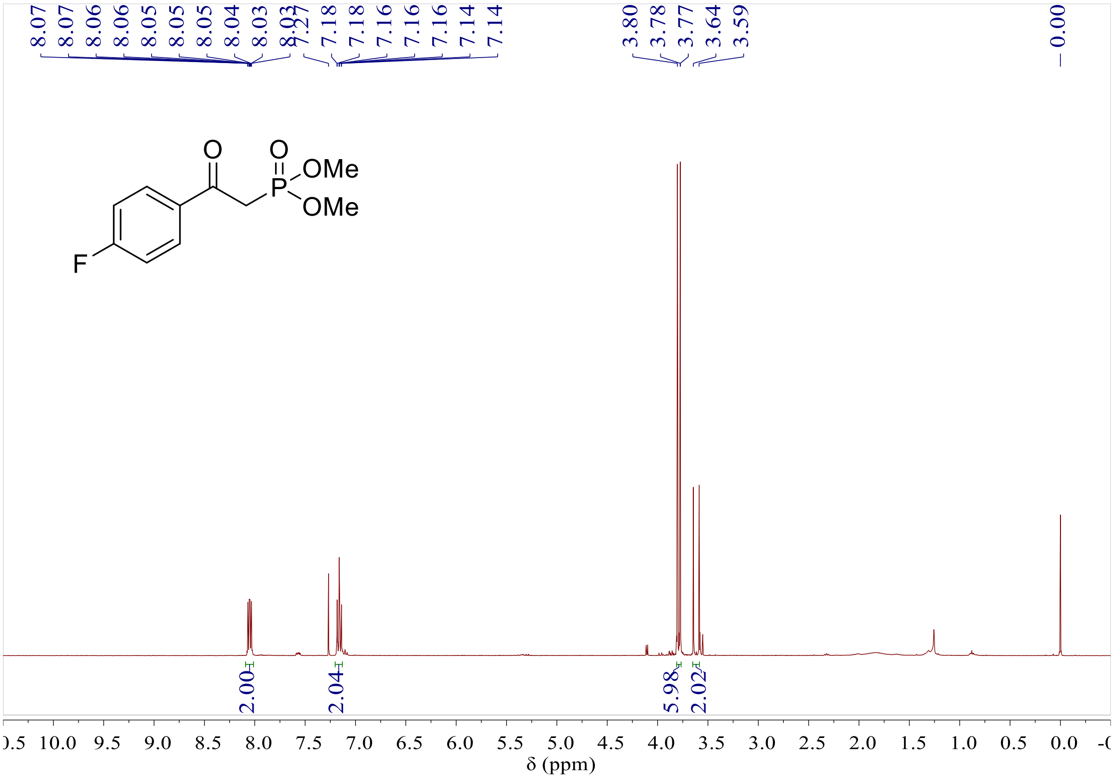

^{1}H NMR spectrum of **4f** in $CDCl_3$ (400 MHz)

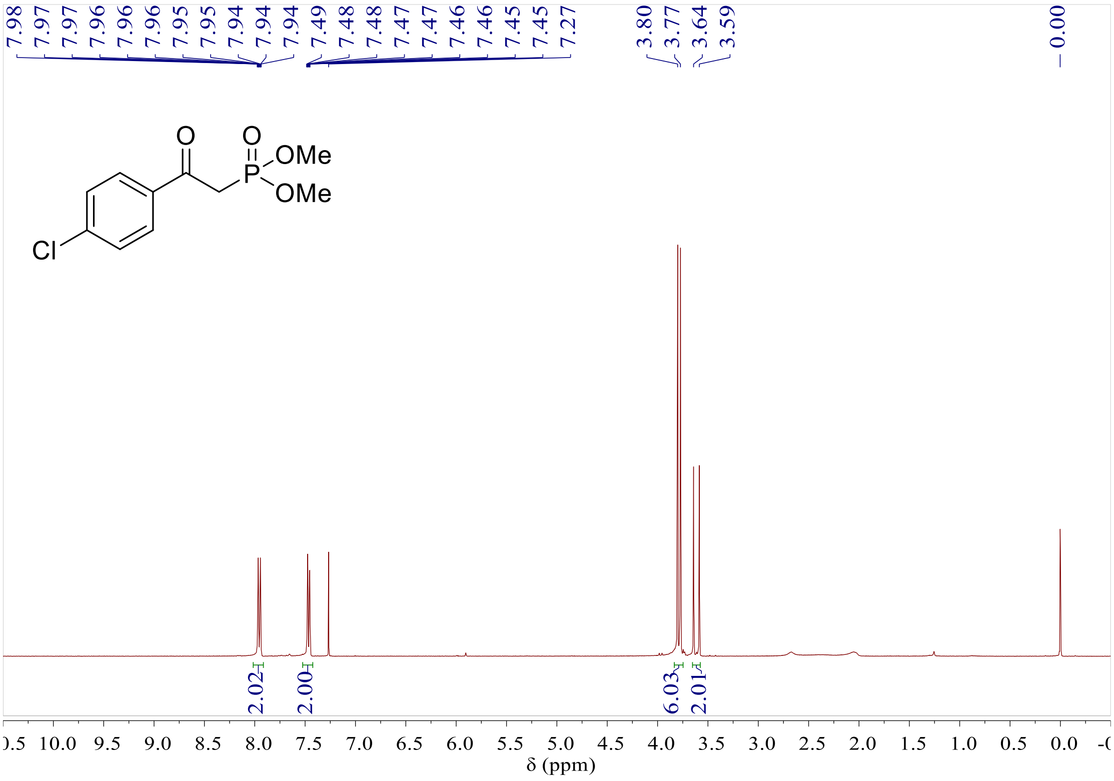

^{1}H NMR spectrum of **4g** in $CDCl_3$ (400 MHz)

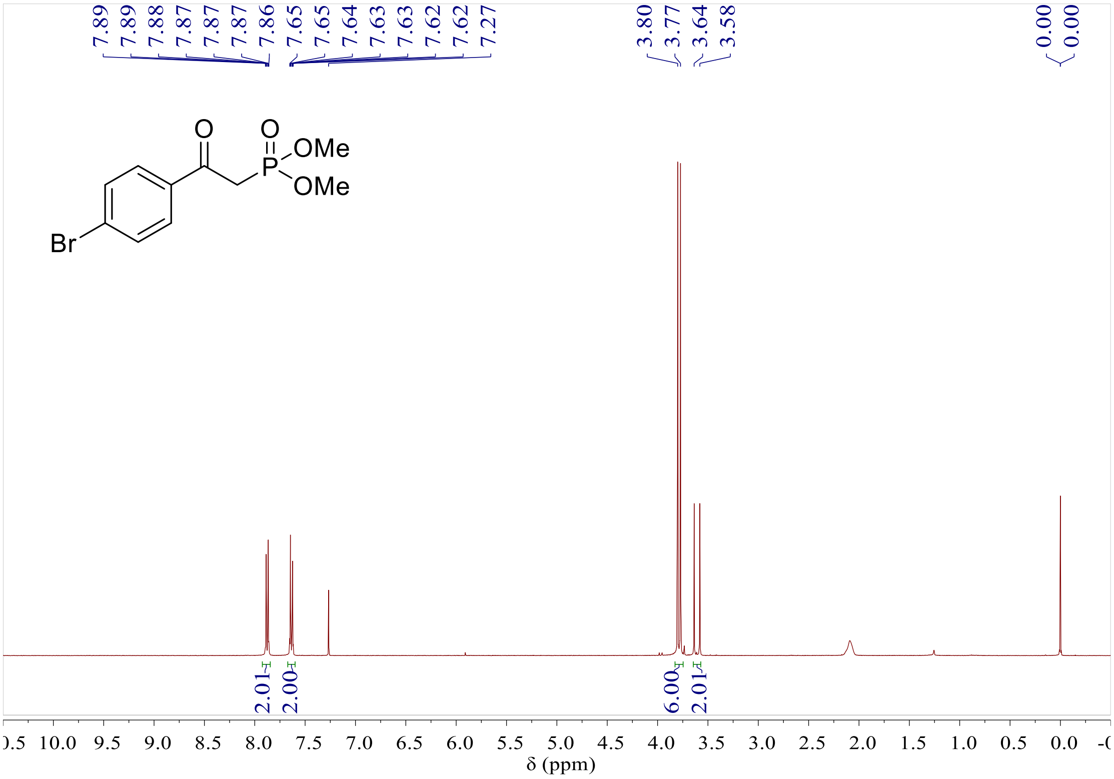

^{1}H NMR spectrum of **4h** in $CDCl_3$ (400 MHz)

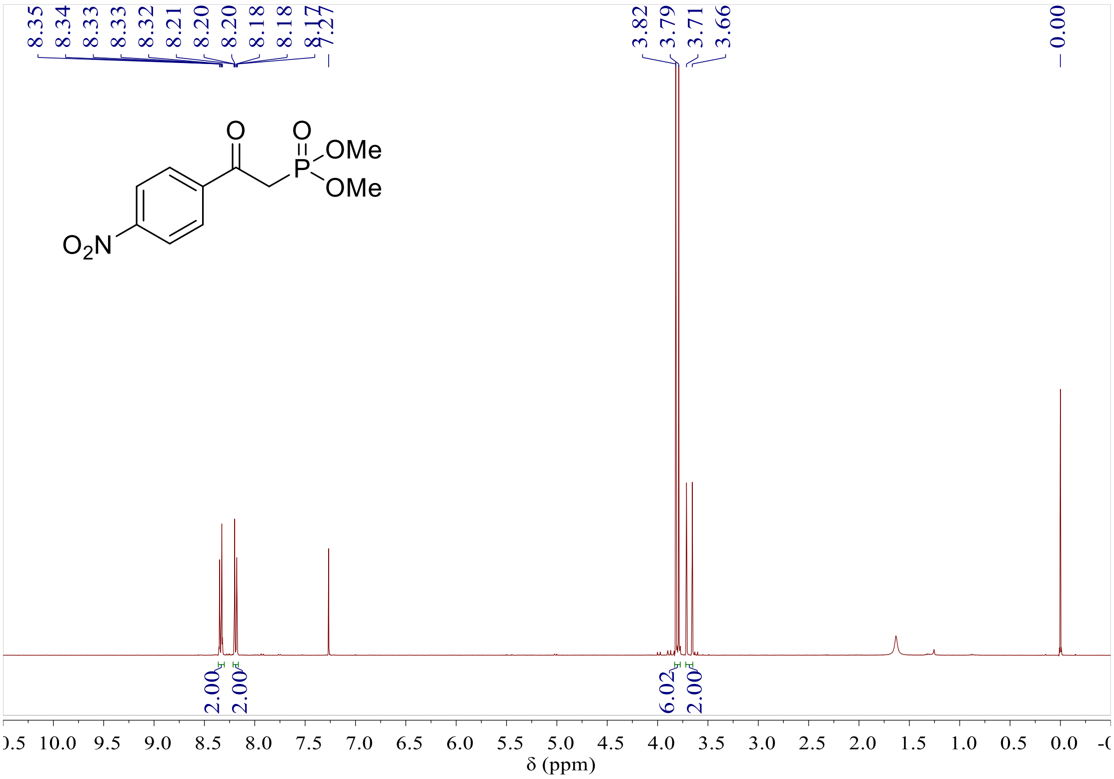

^{1}H NMR spectrum of **4i** in $CDCl_3$ (400 MHz)

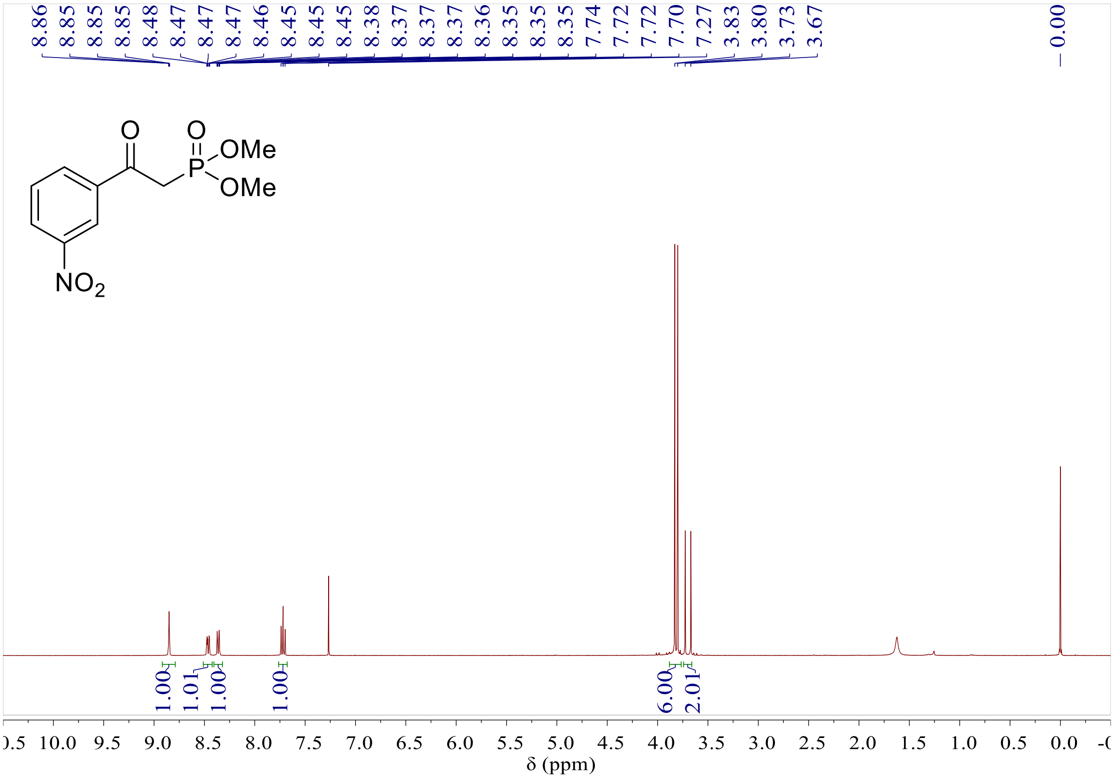

^{1}H NMR spectrum of **4j** in $CDCl_3$ (400 MHz)

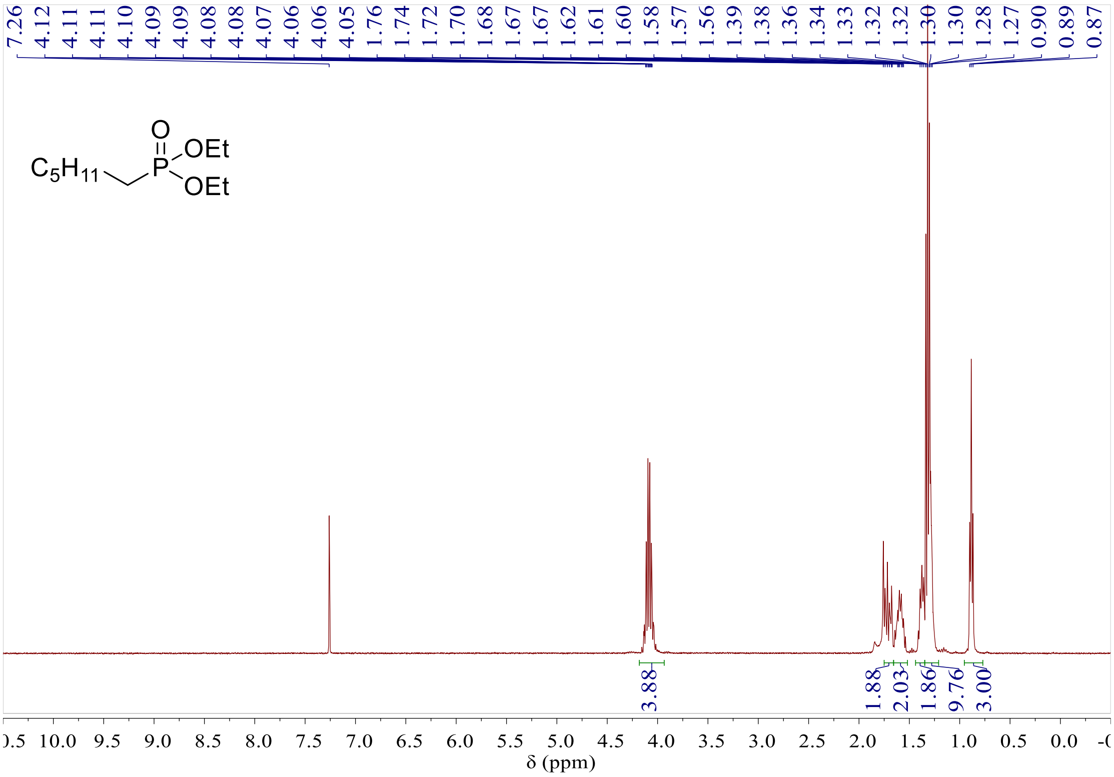

^{1}H NMR spectrum of **4l** in $CDCl_3$ (400 MHz)

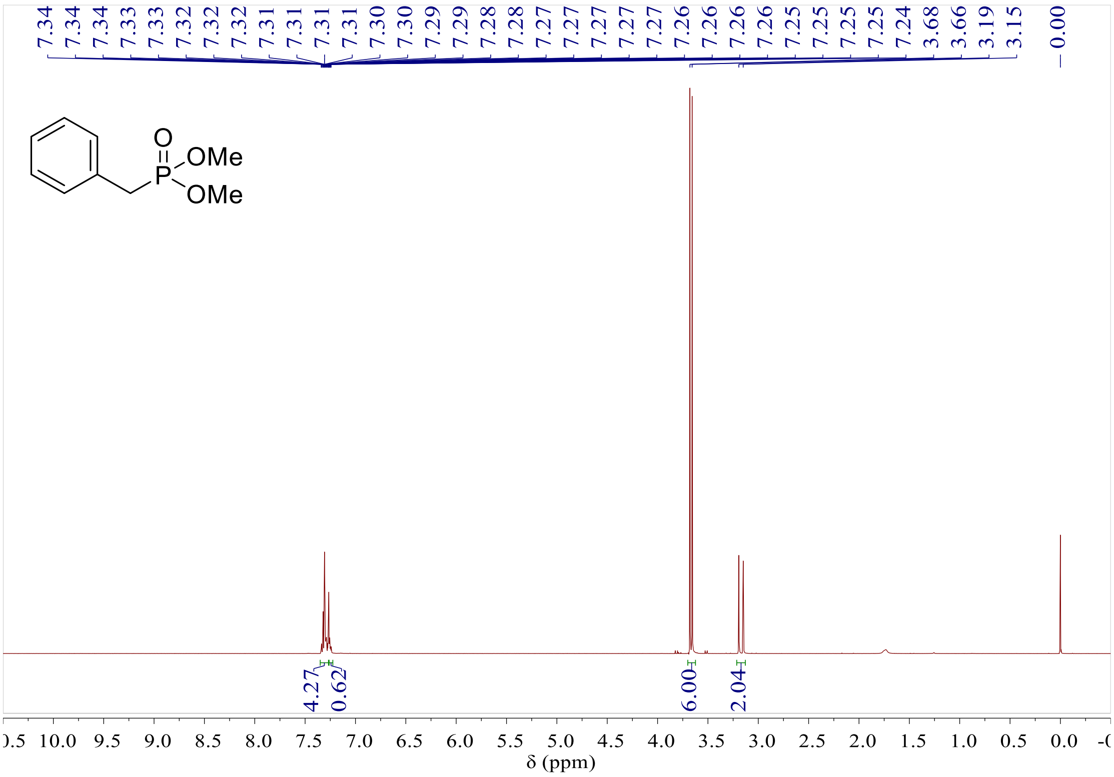

^{1}H NMR spectrum of **4q** in $CDCl_3$ (500 MHz)

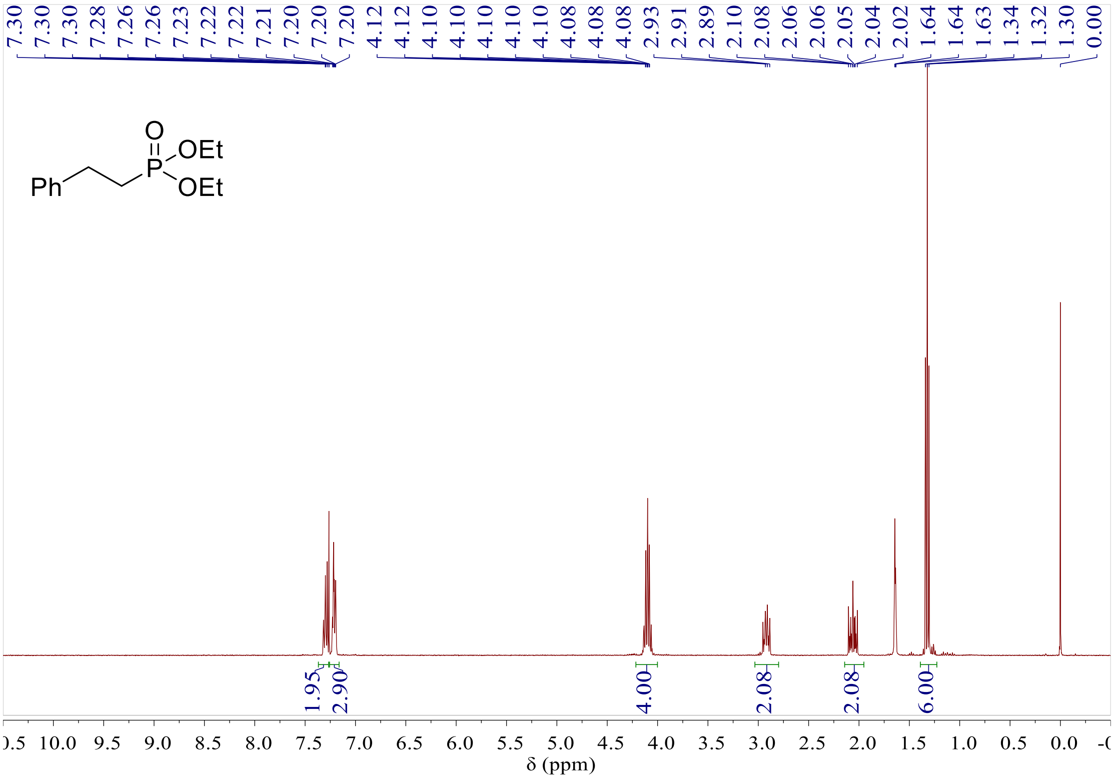

^{1}H NMR spectrum of **4v** in $CDCl_3$ (400 MHz)

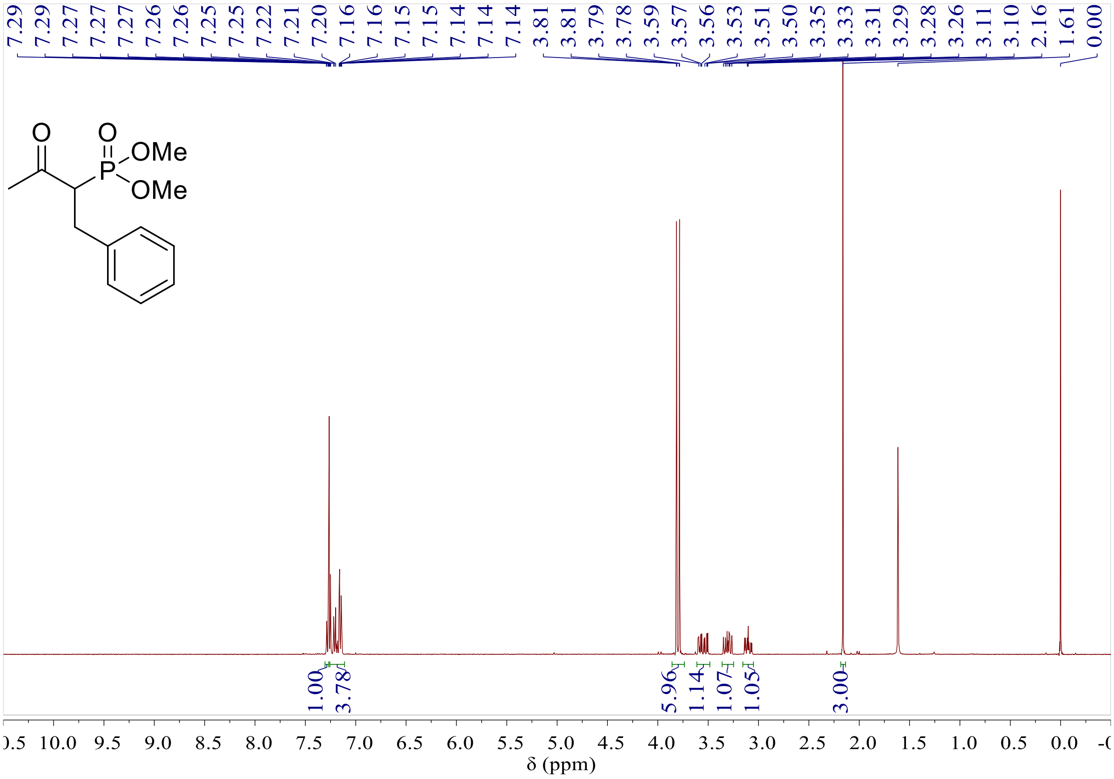

^{1}H NMR spectrum of **1a** in $CDCl_3$ (400 MHz)

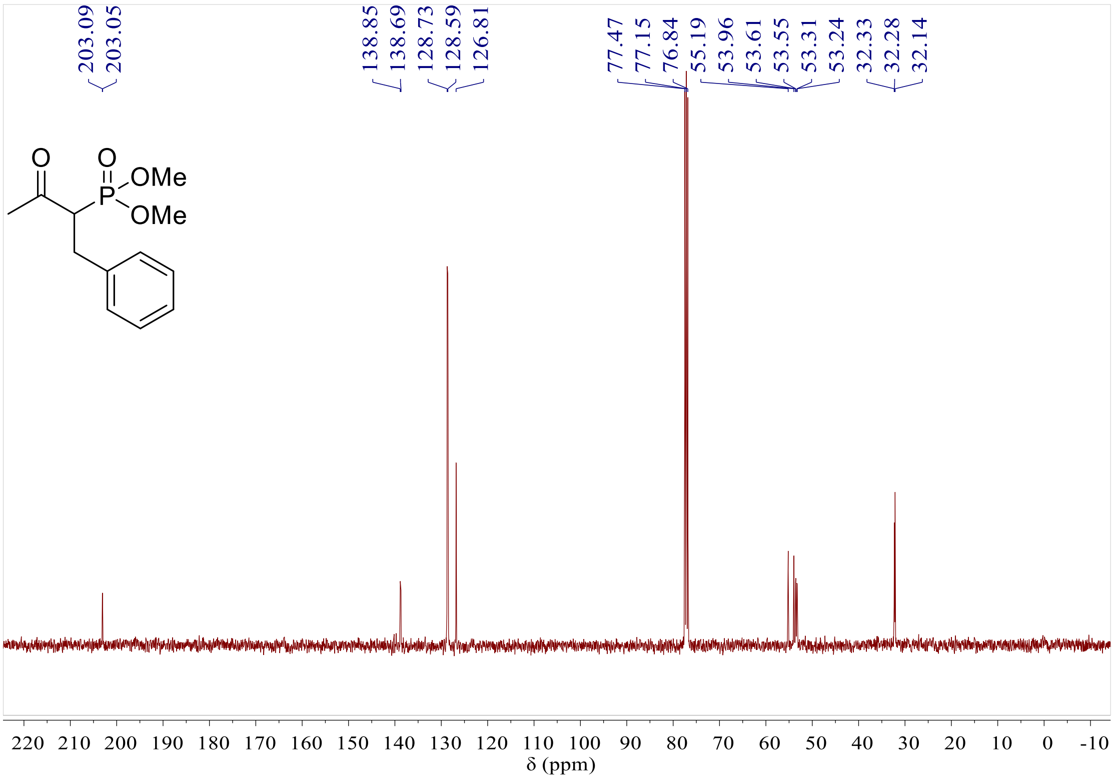

$^{13}C\{^{1}H\}$ NMR spectrum of **1a** in $CDCl_3$ (100 MHz)

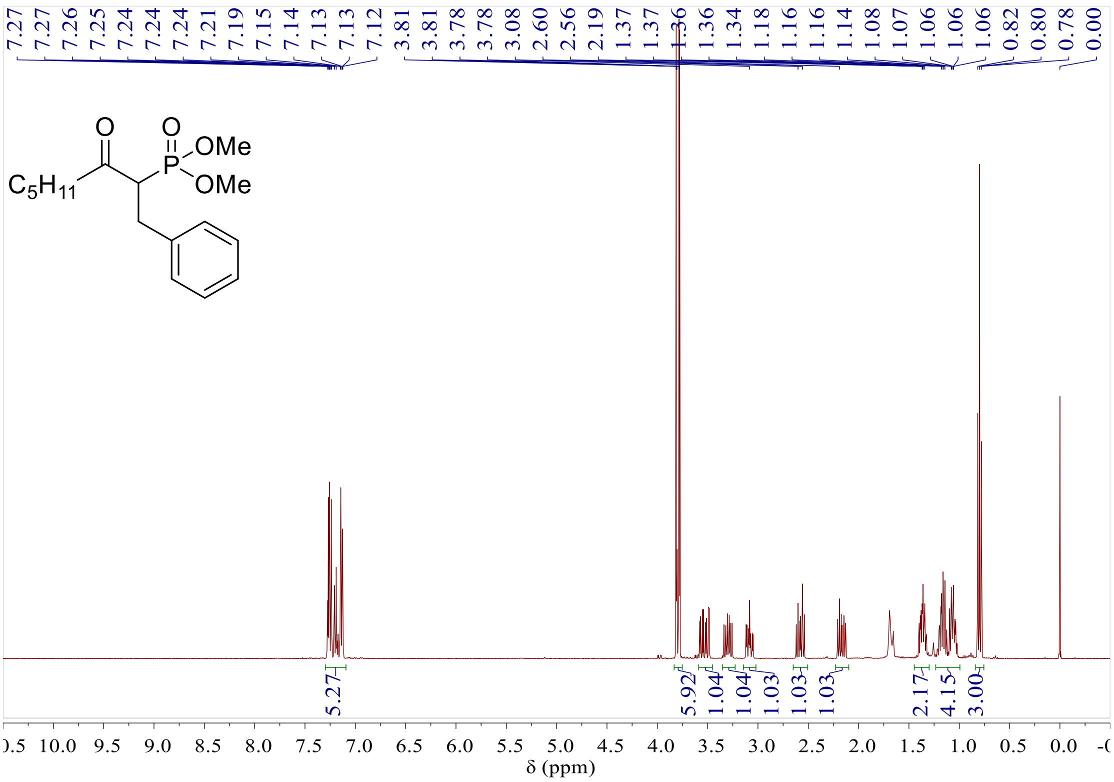

^{1}H NMR spectrum of **1b** in $CDCl_3$ (400 MHz)

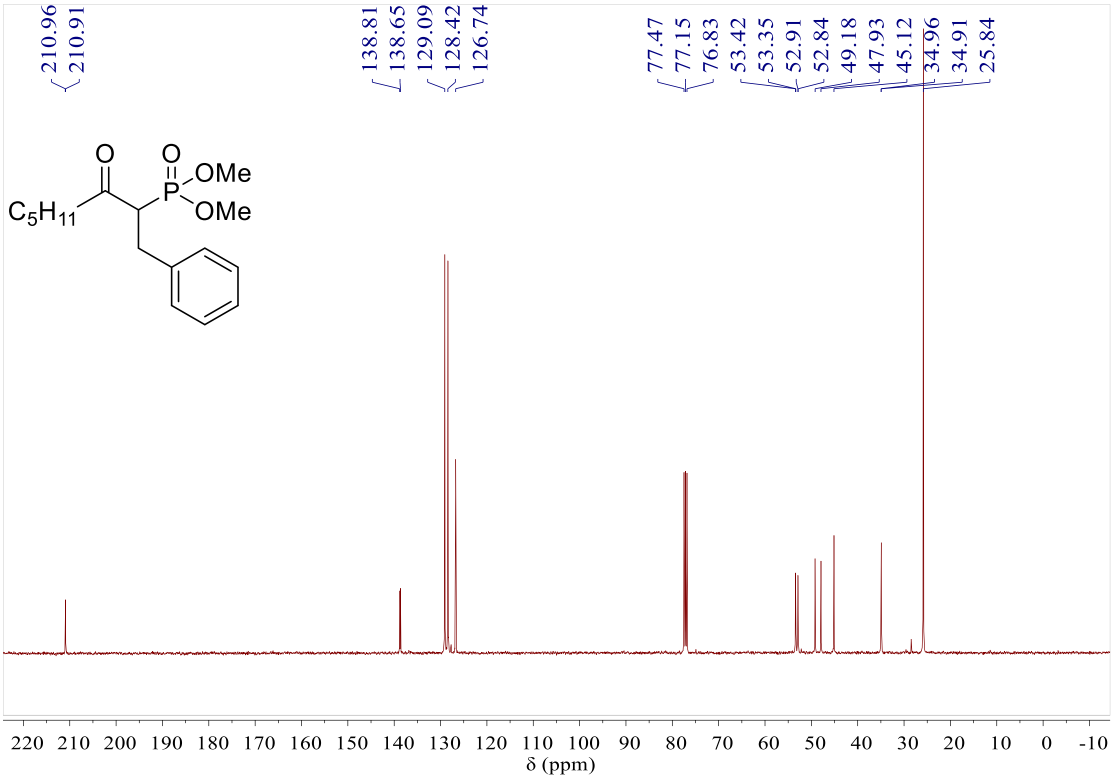

^{13}C{^{1}H} NMR spectrum of **1b** in $CDCl_3$ (100 MHz)

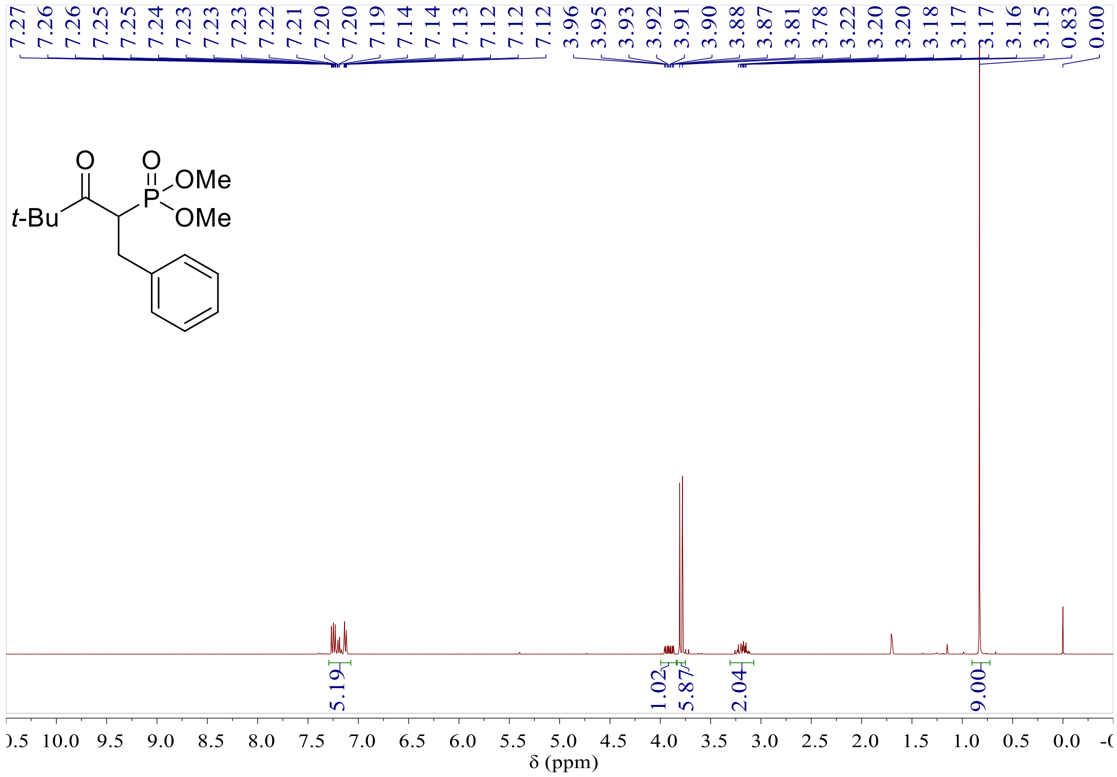

^{1}H NMR spectrum of **1c** in CDCl$_3$ (400 MHz)

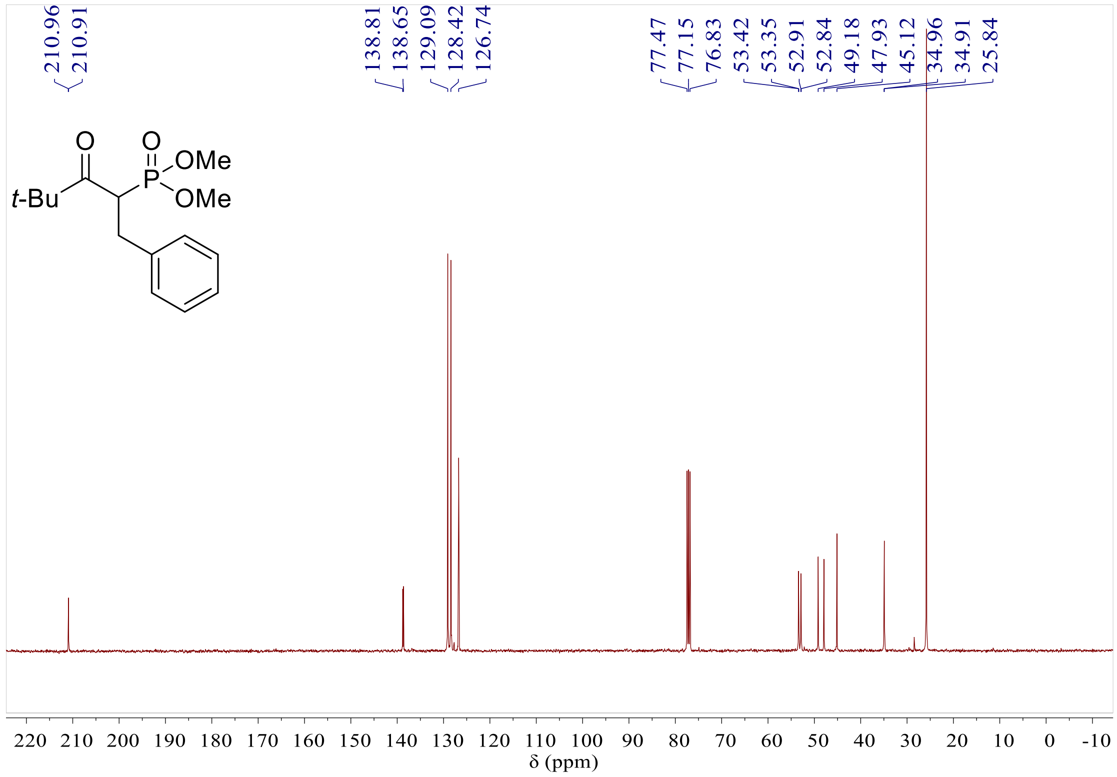

^{13}C{^{1}H} NMR spectrum of **1c** in CDCl$_3$ (100 MHz)

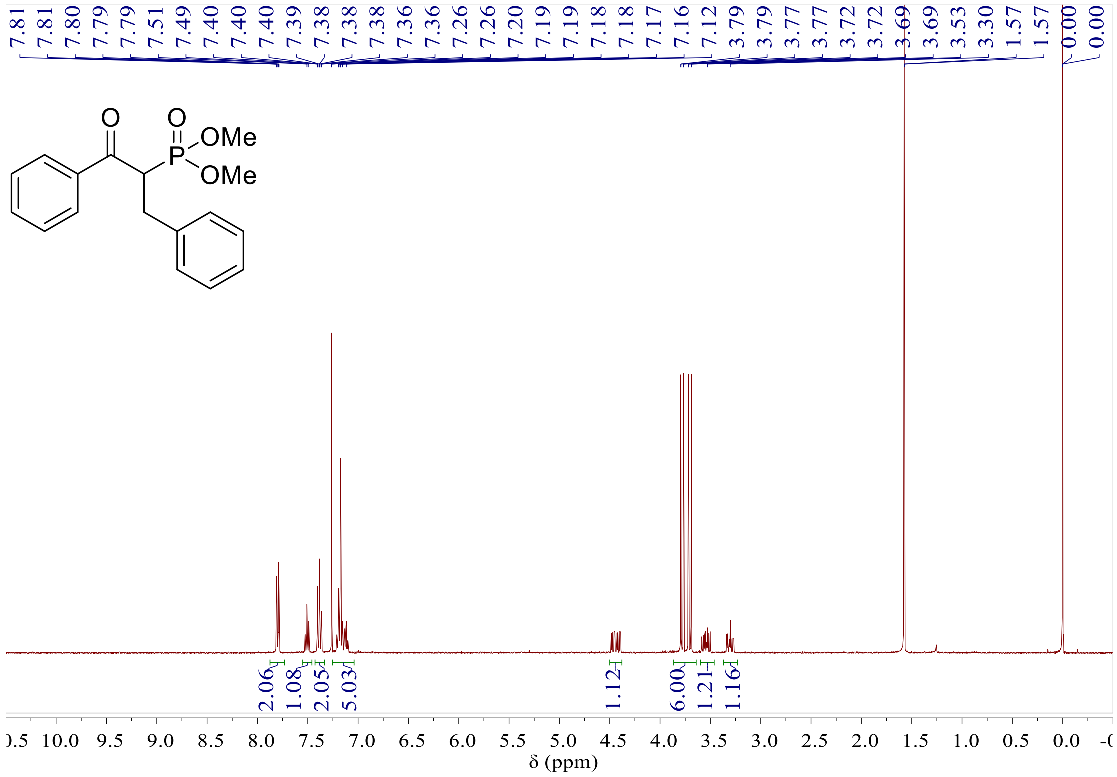

^{1}H NMR spectrum of **1d** in $CDCl_3$ (400 MHz)

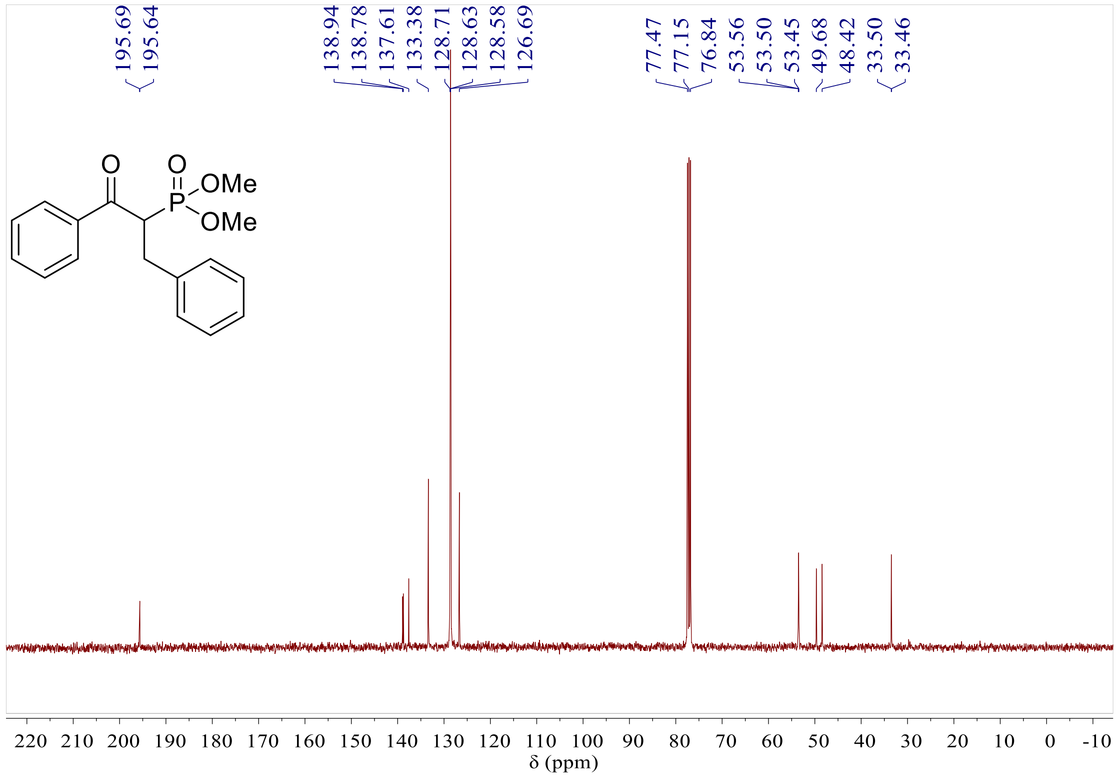

^{13}C{^{1}H} NMR spectrum of **1d** in $CDCl_3$ (100 MHz)

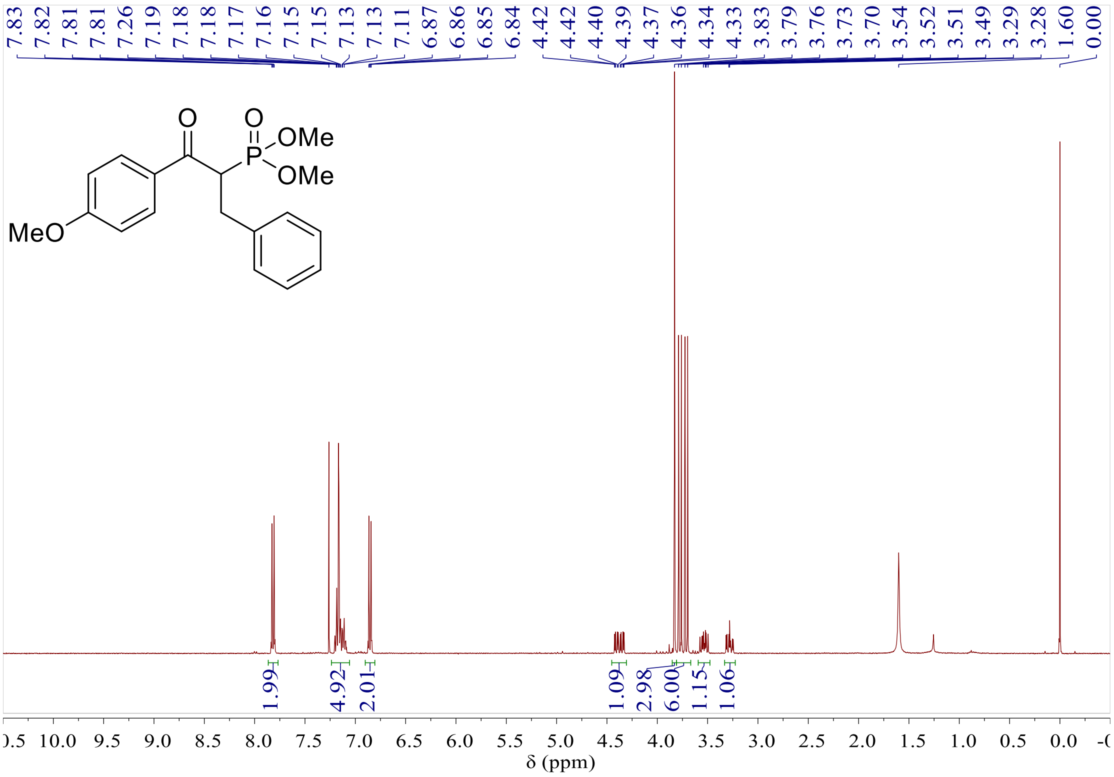

^{1}H NMR spectrum of **1e** in $CDCl_3$ (400 MHz)

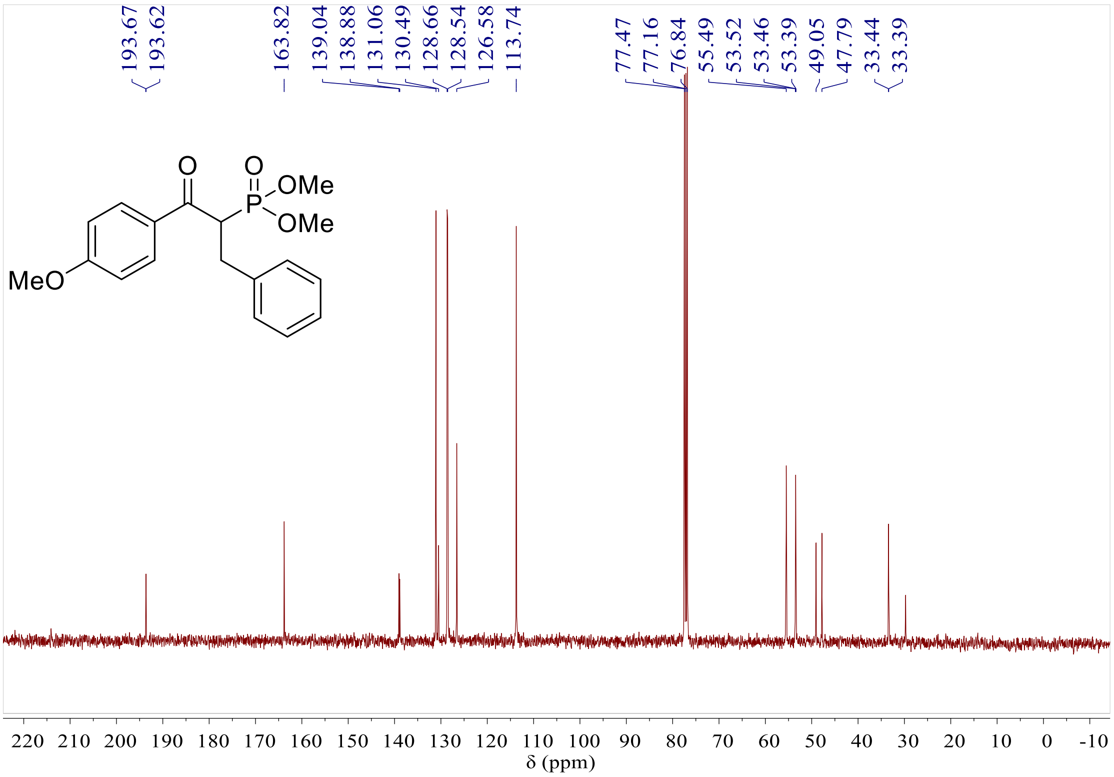

^{13}C{^{1}H} NMR spectrum of **1e** in $CDCl_3$ (100 MHz)

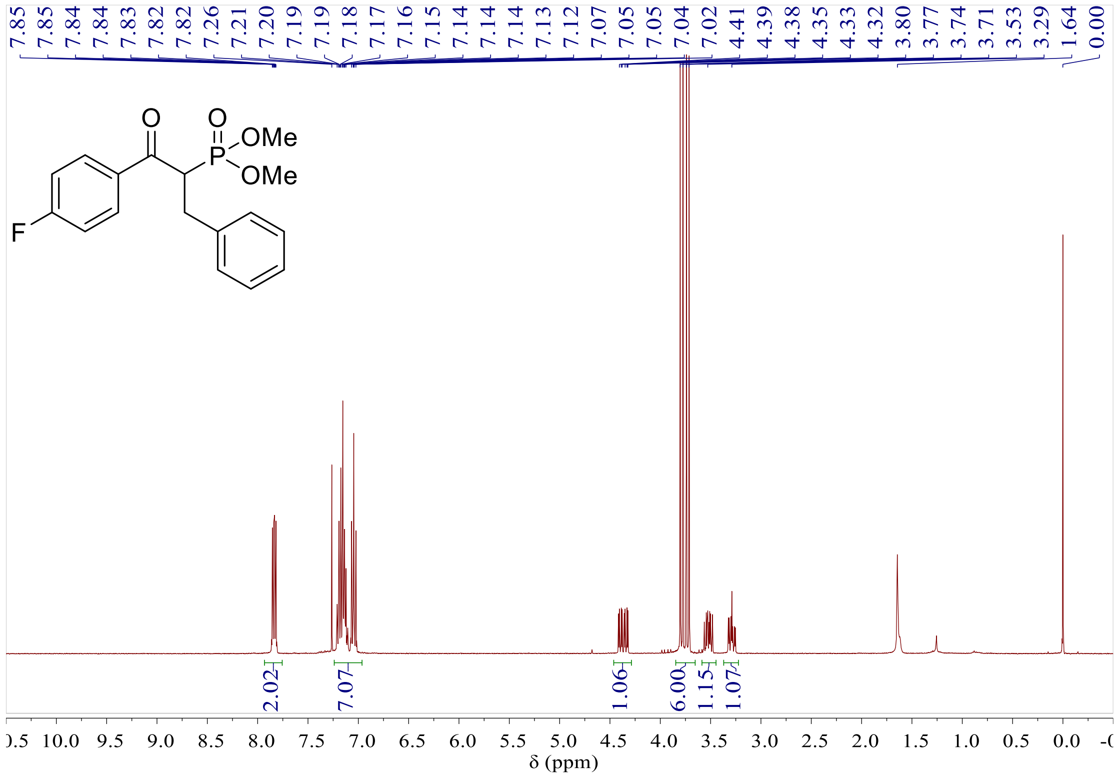

^{1}H NMR spectrum of **1f** in $CDCl_3$ (400 MHz)

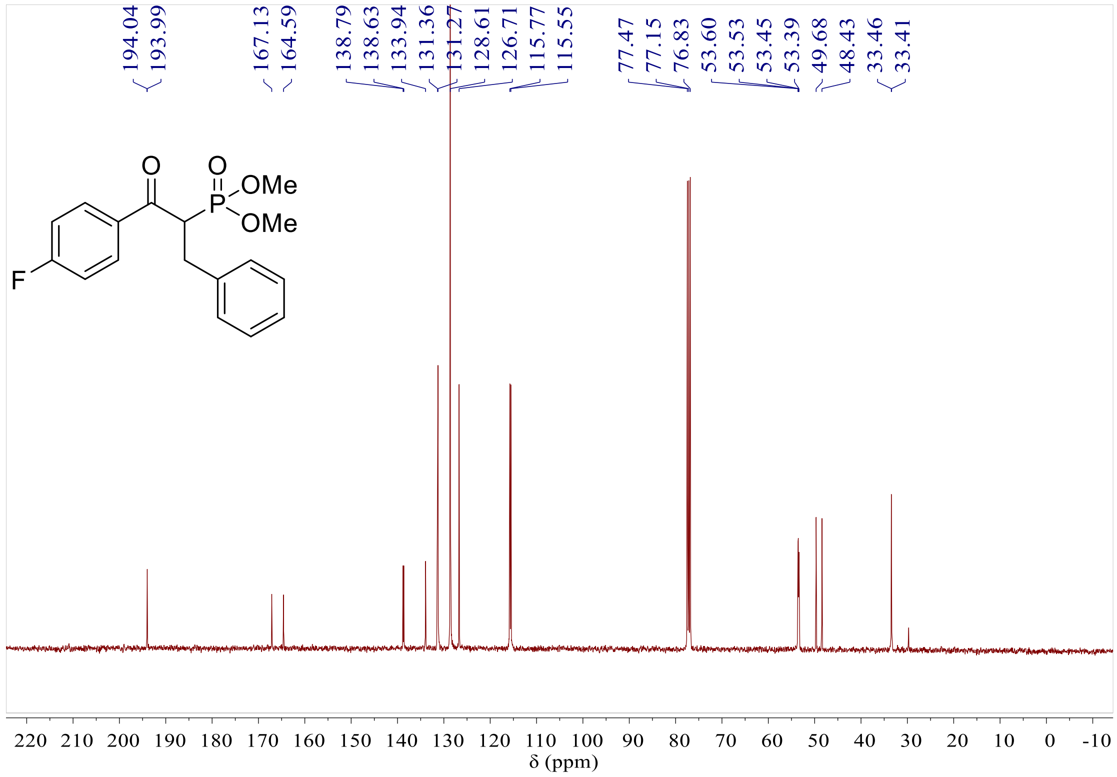

$^{13}C\{^1H\}$ NMR spectrum of **1f** in $CDCl_3$ (100 MHz)

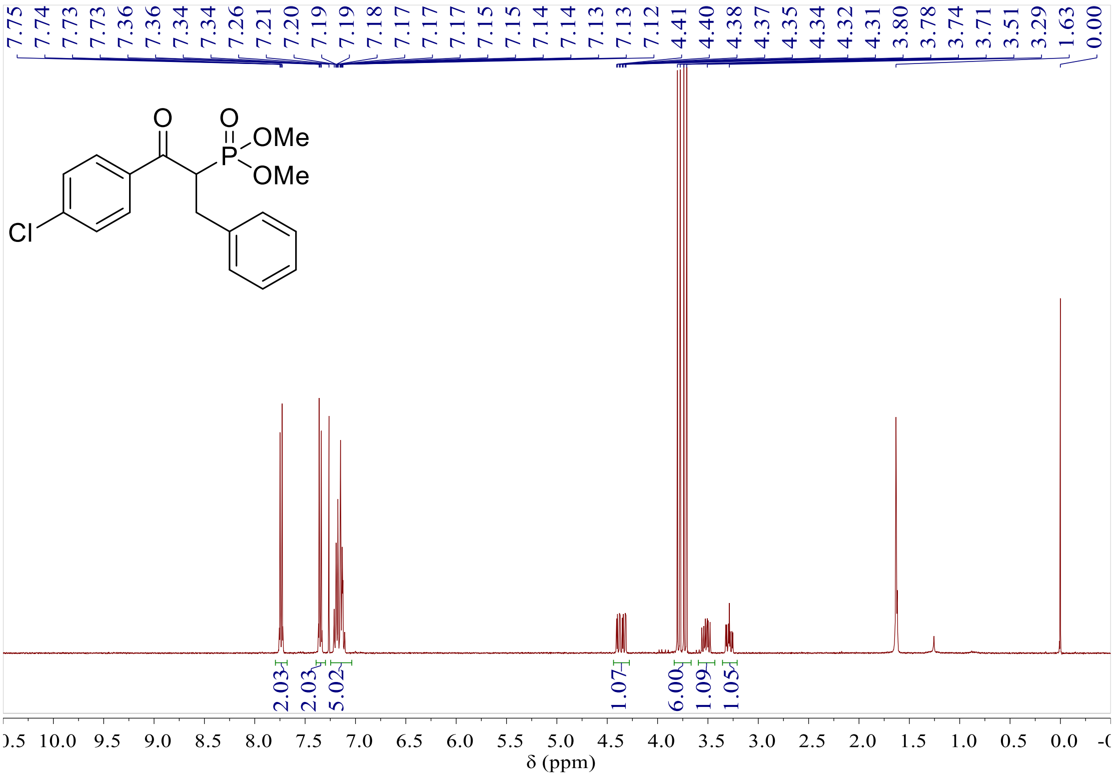

^{1}H NMR spectrum of **1g** in $CDCl_3$ (400 MHz)

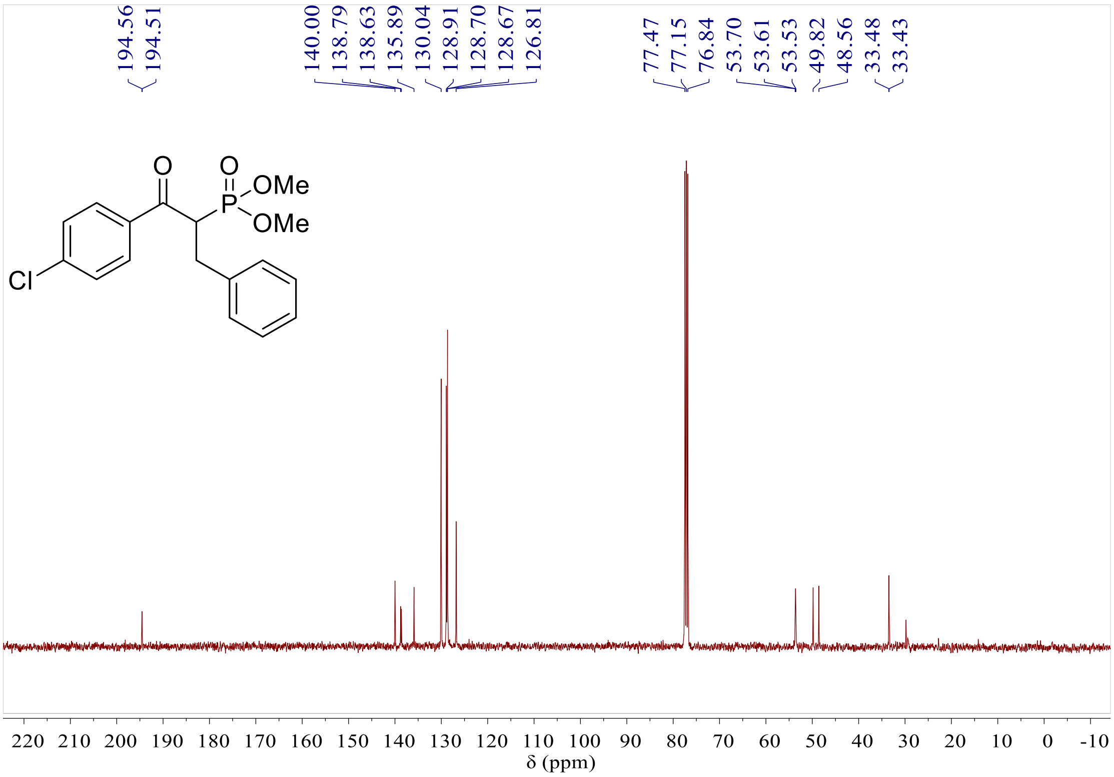

^{13}C{^{1}H} NMR spectrum of **1g** in $CDCl_3$ (100 MHz)

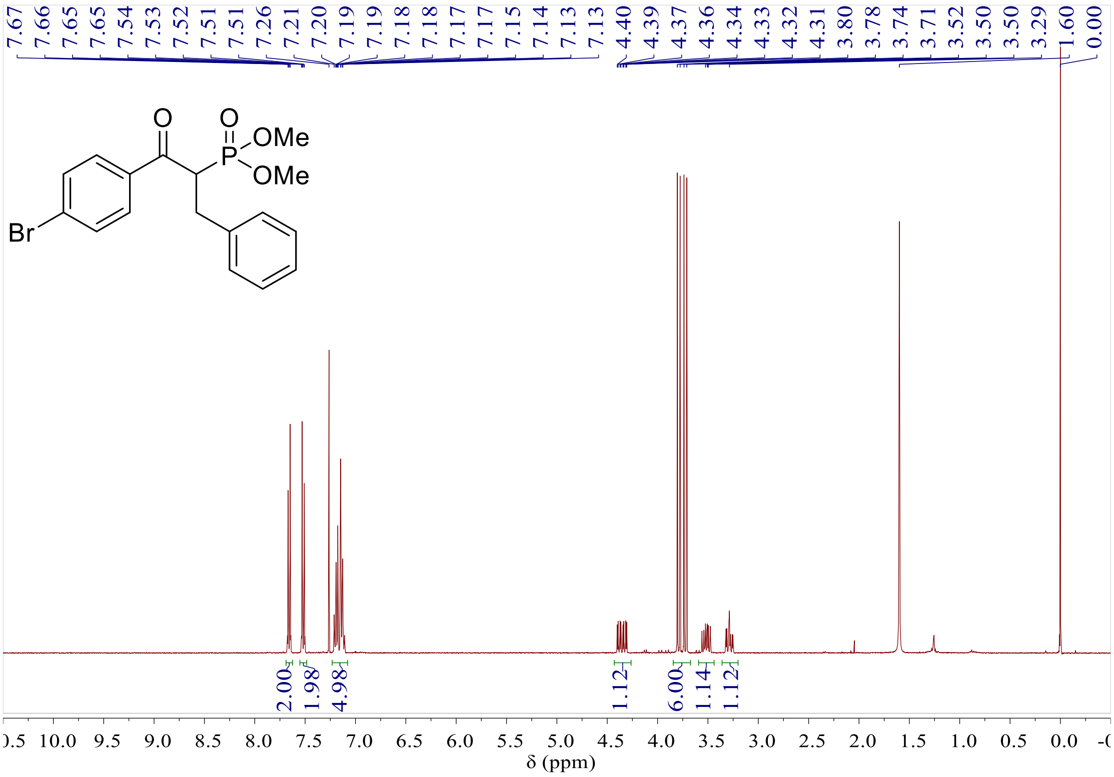

^{1}H NMR spectrum of **1h** in $CDCl_3$ (400 MHz)

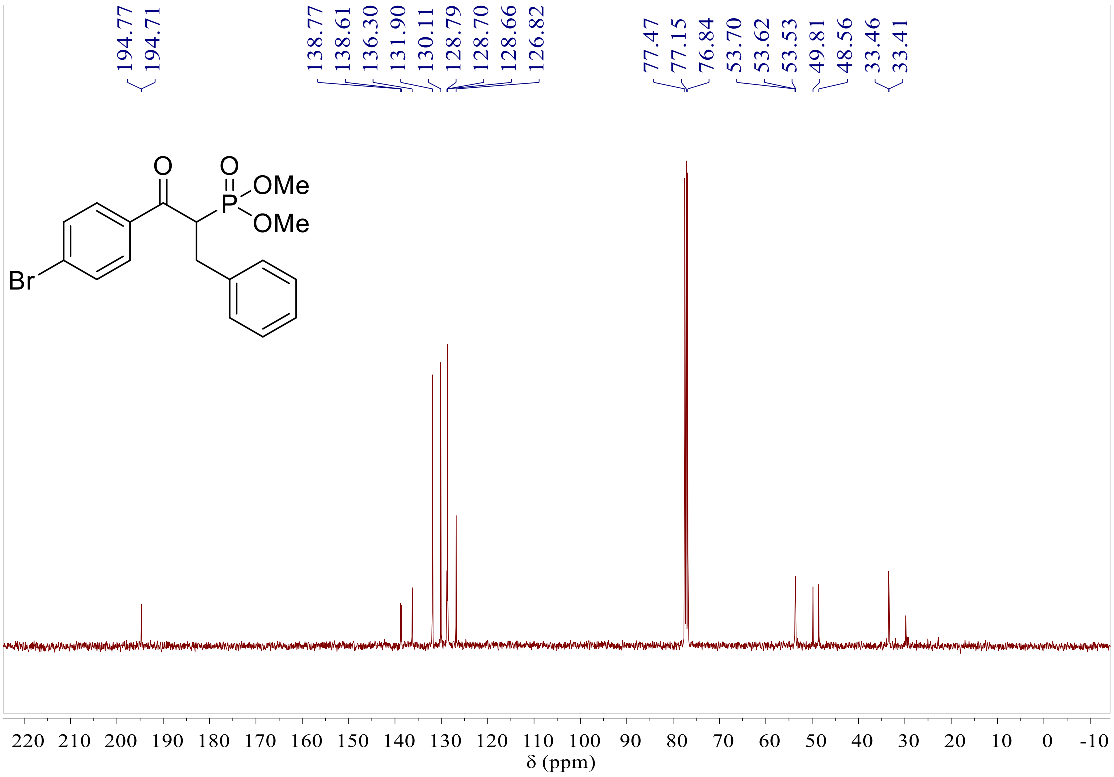

^{13}C{^{1}H} NMR spectrum of **1h** in $CDCl_3$ (100 MHz)

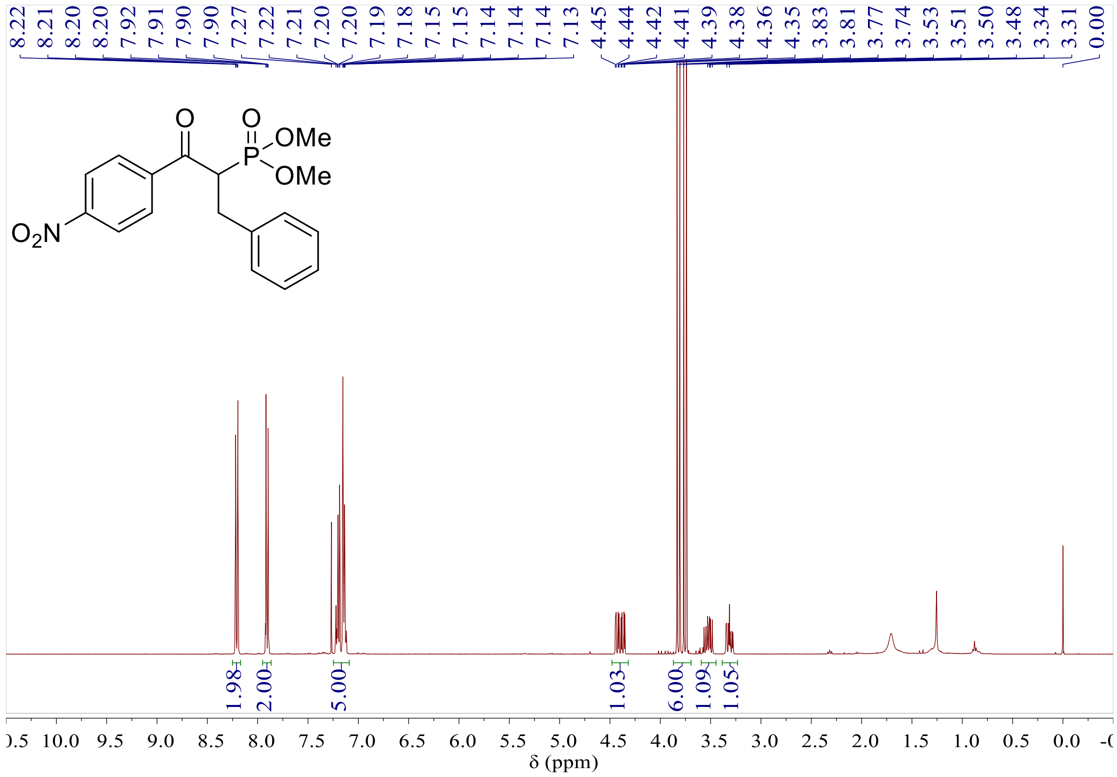

^{1}H NMR spectrum of **1i** in $CDCl_3$ (400 MHz)

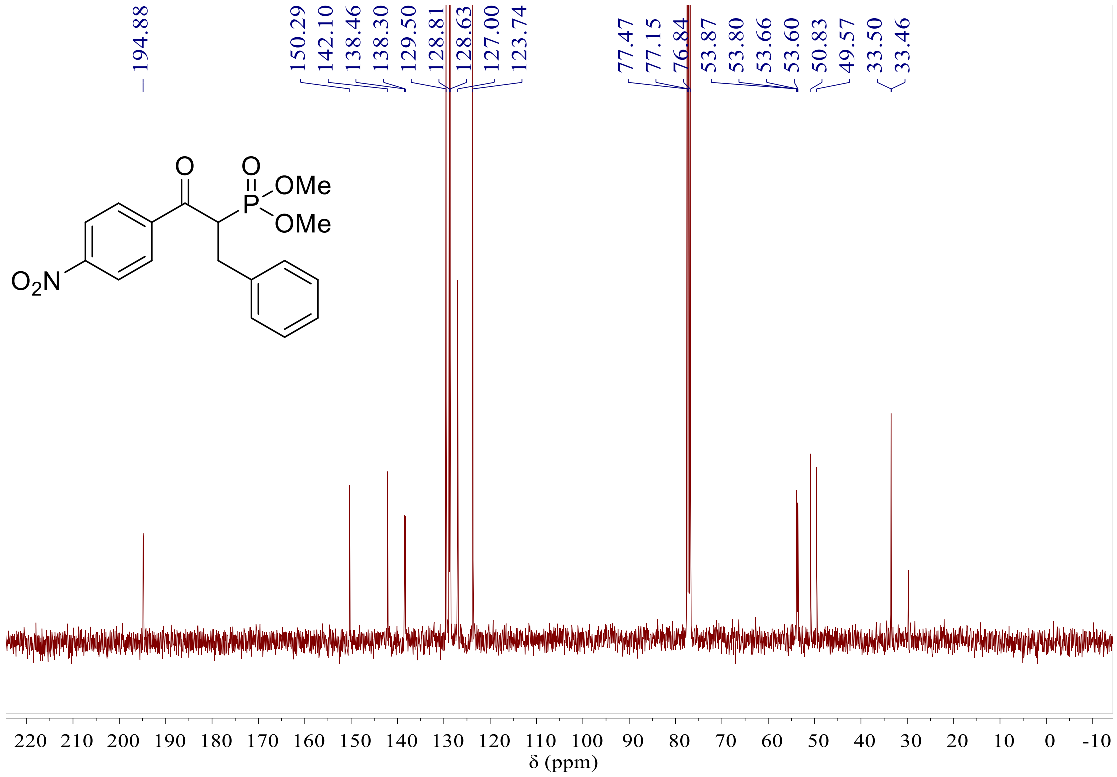

^{13}C{^{1}H} NMR spectrum of **1i** in $CDCl_3$ (100 MHz)

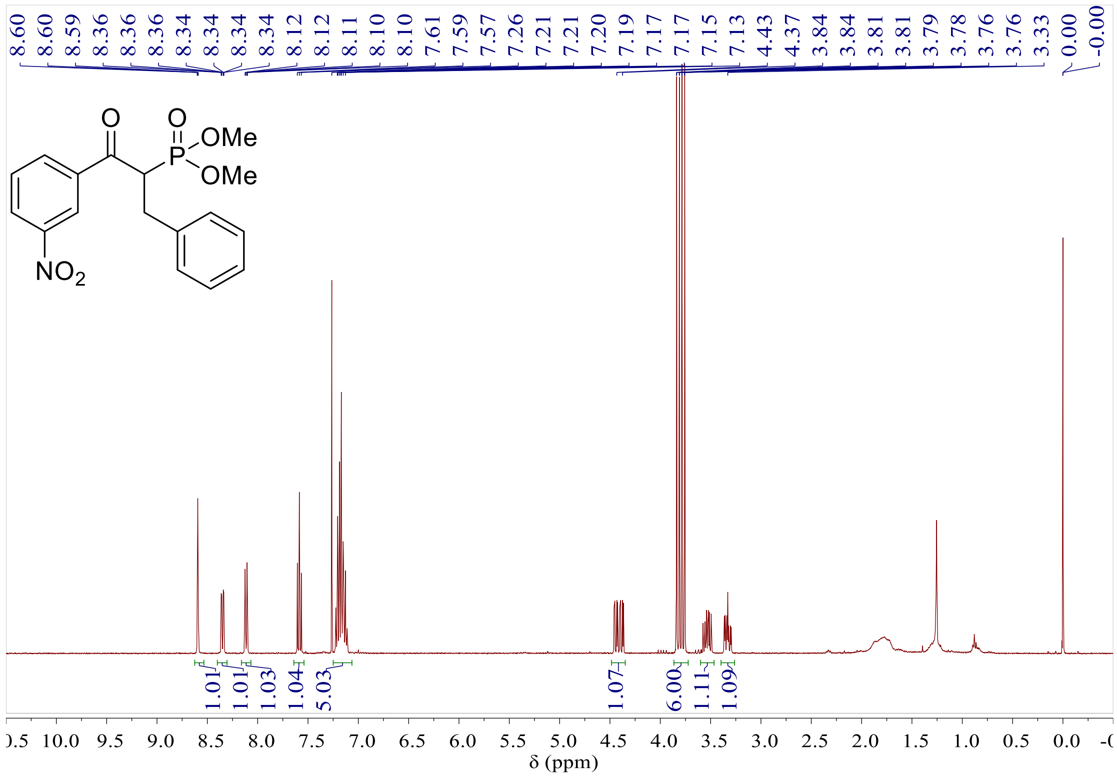

^{1}H NMR spectrum of **1j** in $CDCl_3$ (400 MHz)

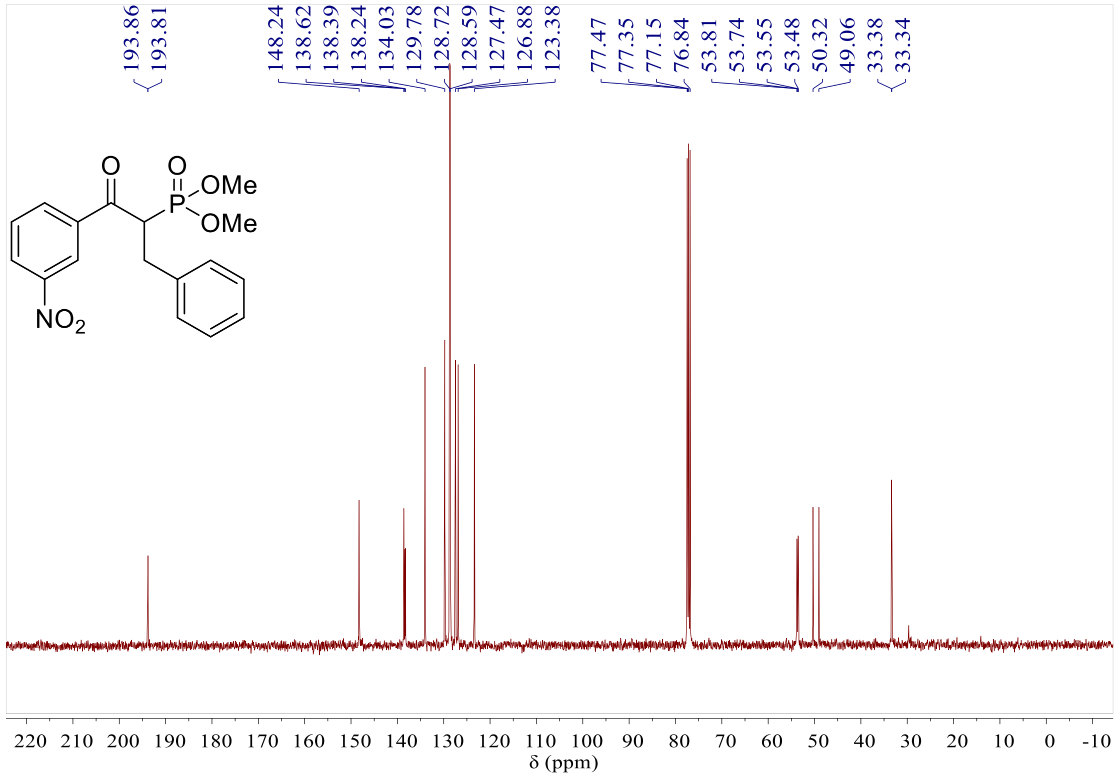

^{13}C{^{1}H} NMR spectrum of **1j** in $CDCl_3$ (100 MHz)

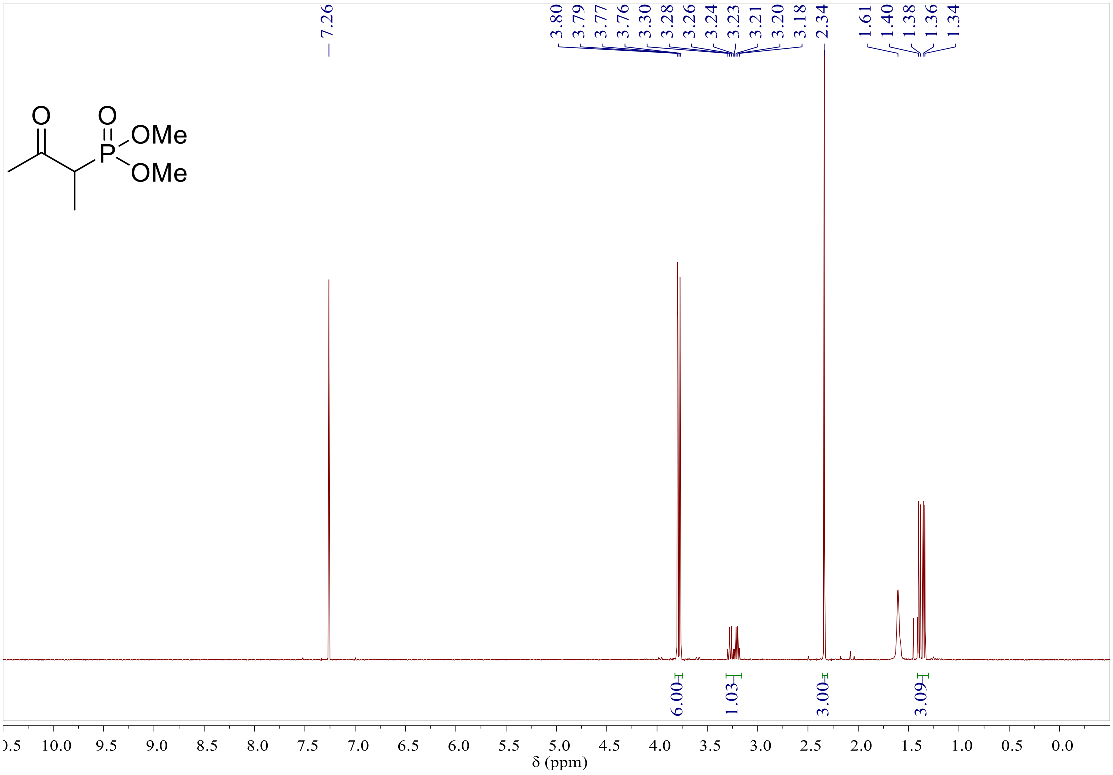

^{1}H NMR spectrum of **1k** in $CDCl_3$ (400 MHz)

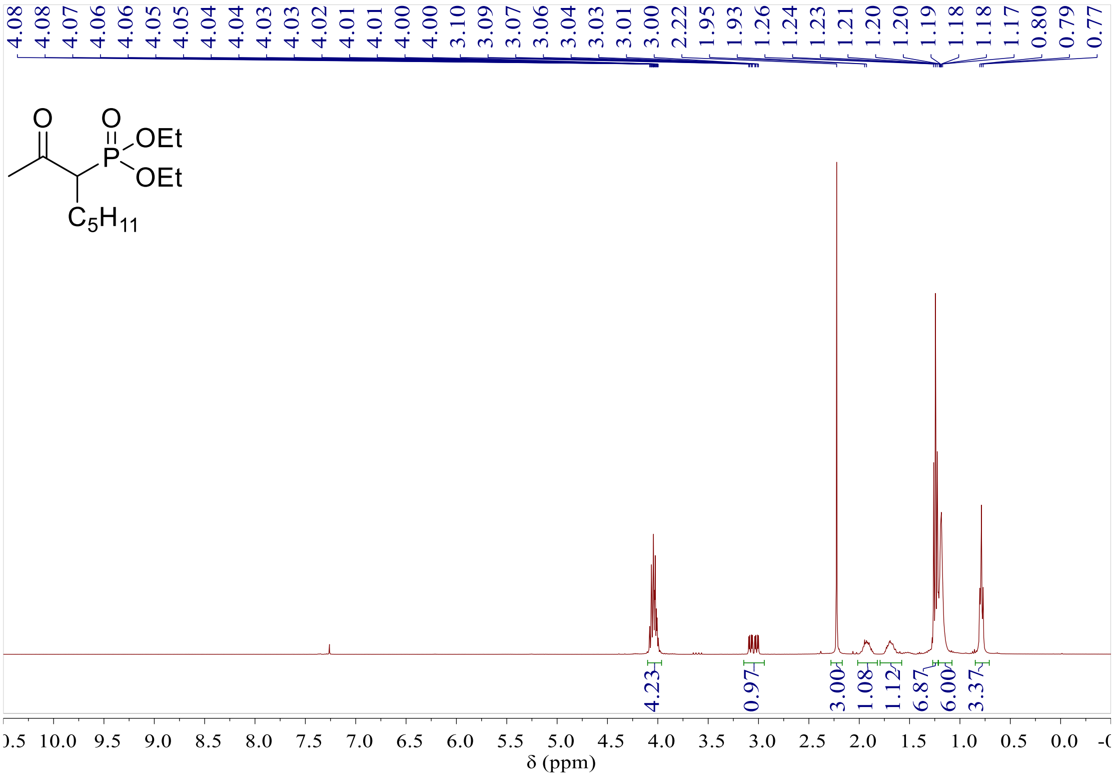

^{1}H NMR spectrum of **1l** in $CDCl_3$ (400 MHz)

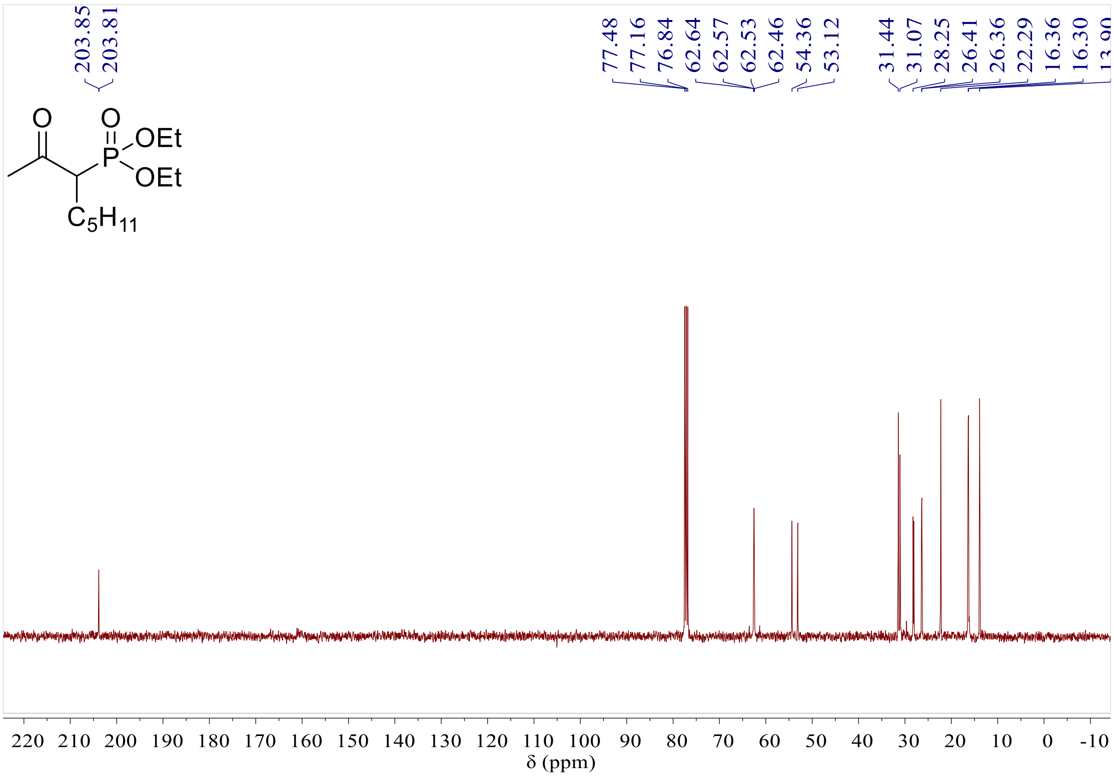

$^{13}C\{^{1}H\}$ NMR spectrum of **1l** in $CDCl_3$ (100 MHz)

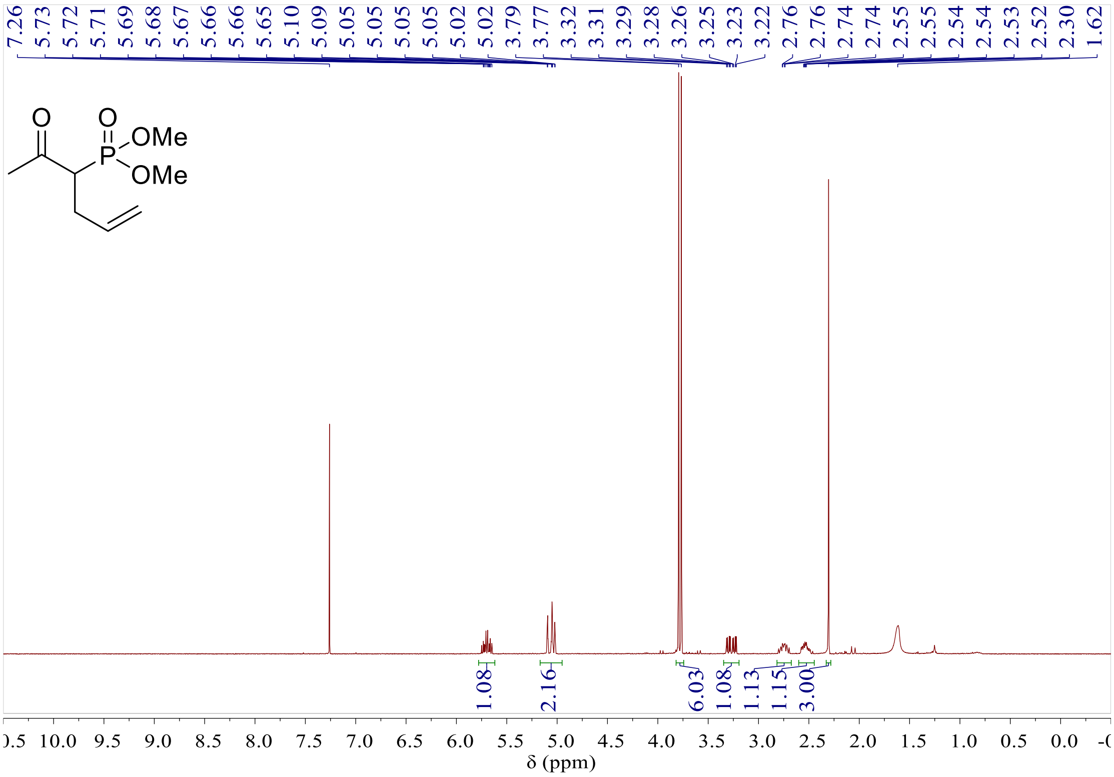

^{1}H NMR spectrum of **1m** in $CDCl_3$ (400 MHz)

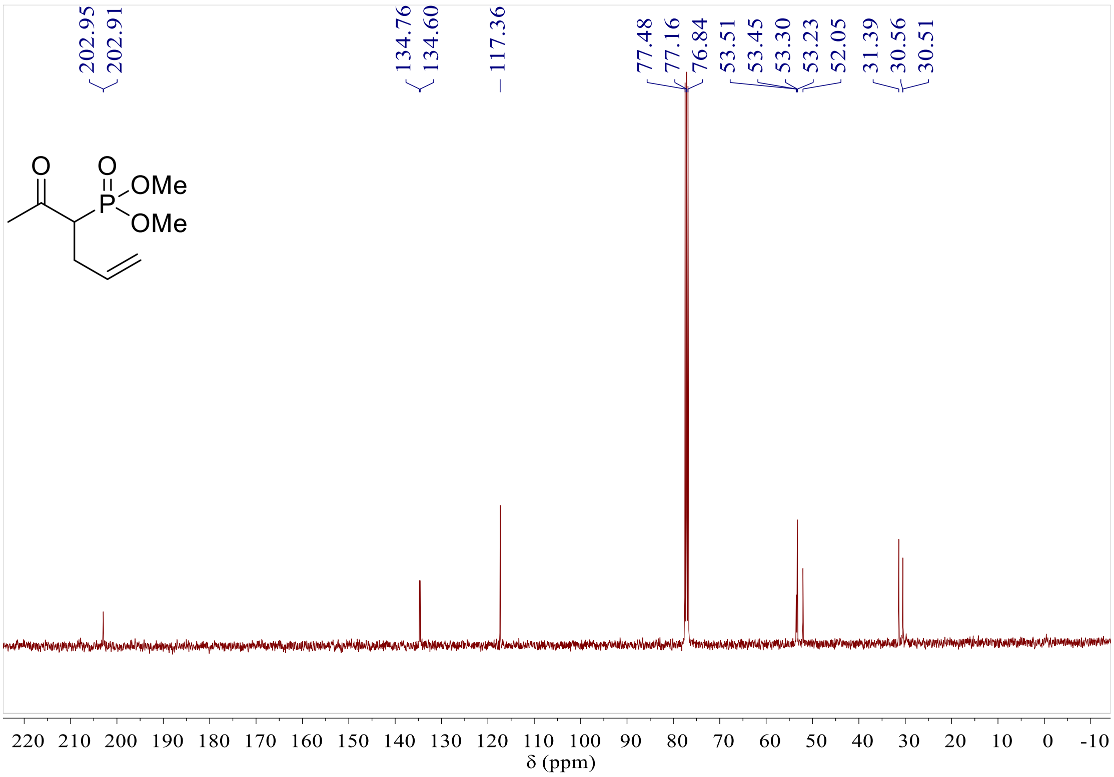

$^{13}C\{^1H\}$ NMR spectrum of **1m** in $CDCl_3$ (100 MHz)

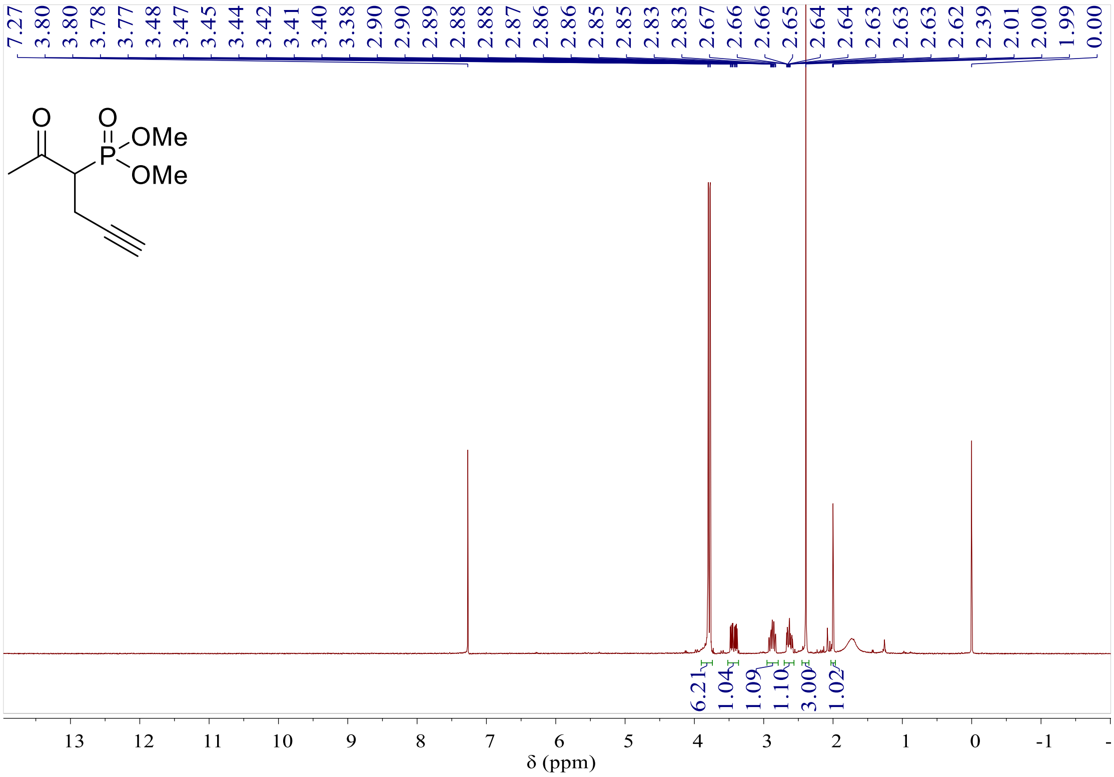

^1H NMR spectrum of **1n** in $CDCl_3$ (400 MHz)

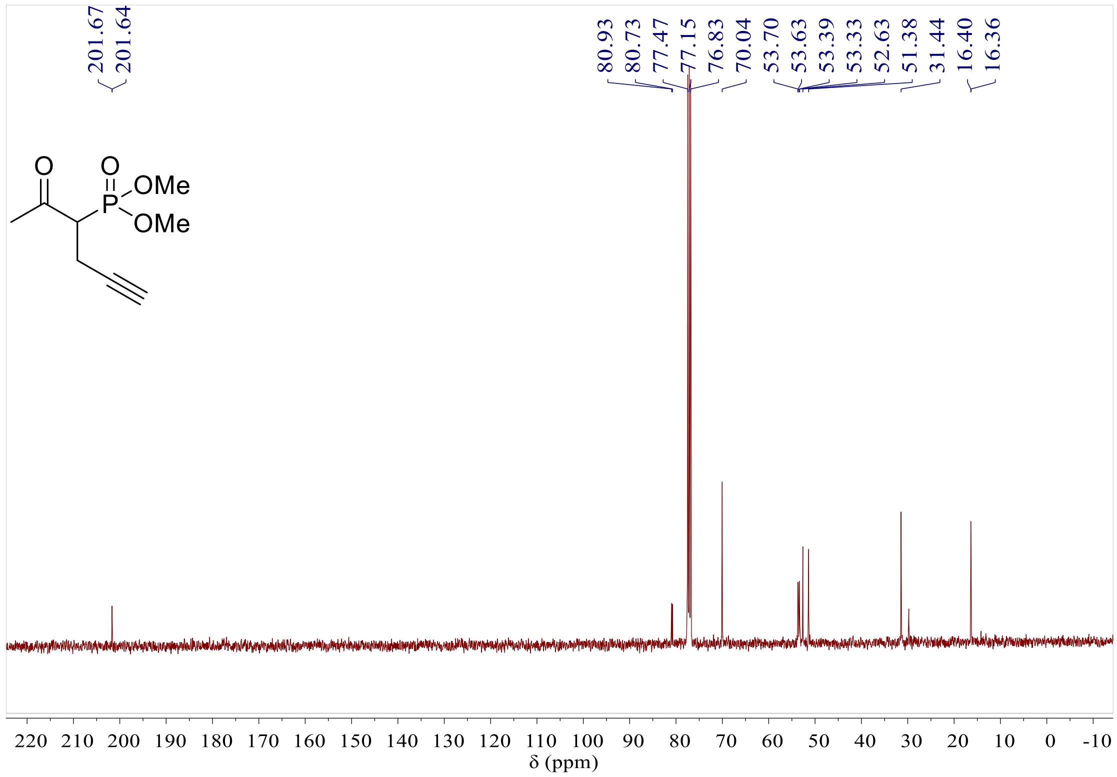

$^{13}C\{^{1}H\}$ NMR spectrum of **1n** in $CDCl_3$ (100 MHz)

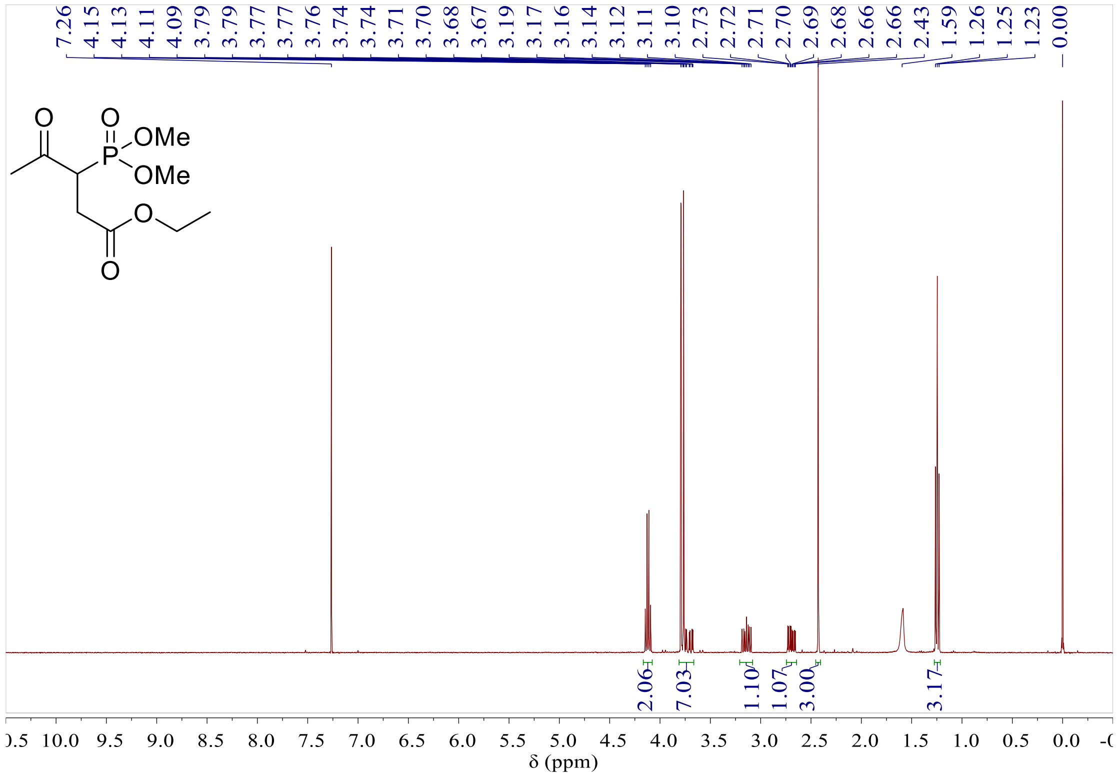

^{1}H NMR spectrum of **1o** in $CDCl_3$ (400 MHz)

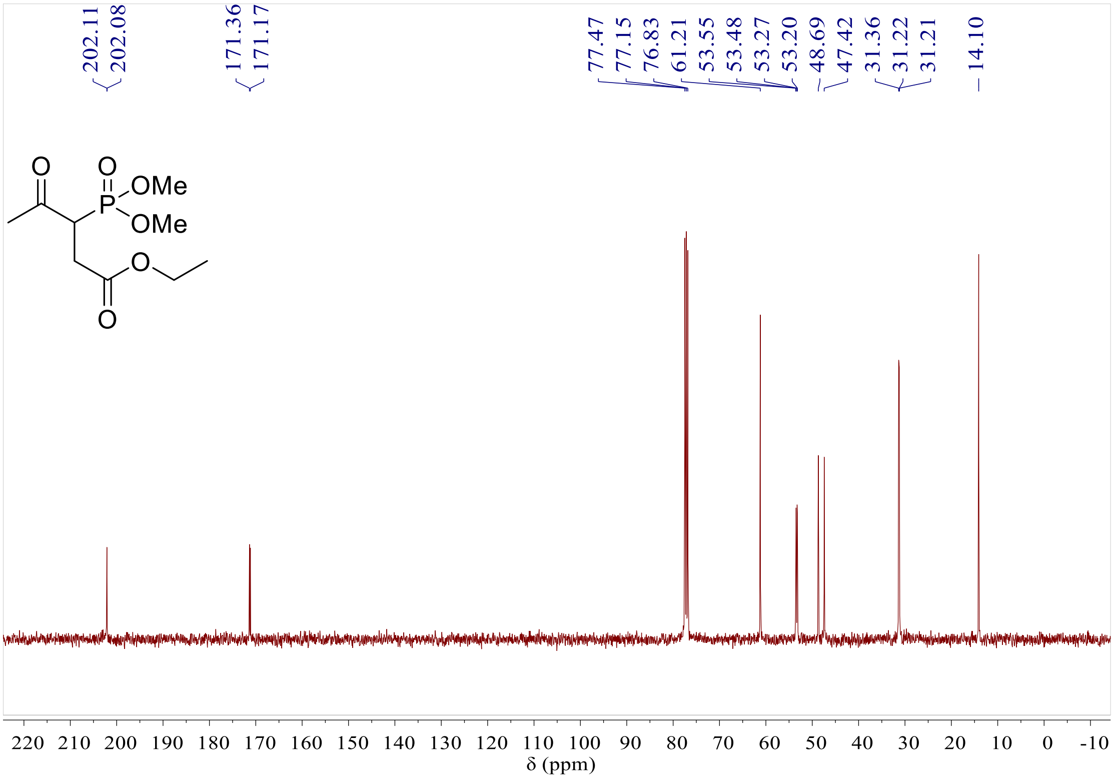

$^{13}C\{^{1}H\}$ NMR spectrum of **1o** in $CDCl_3$ (100 MHz)

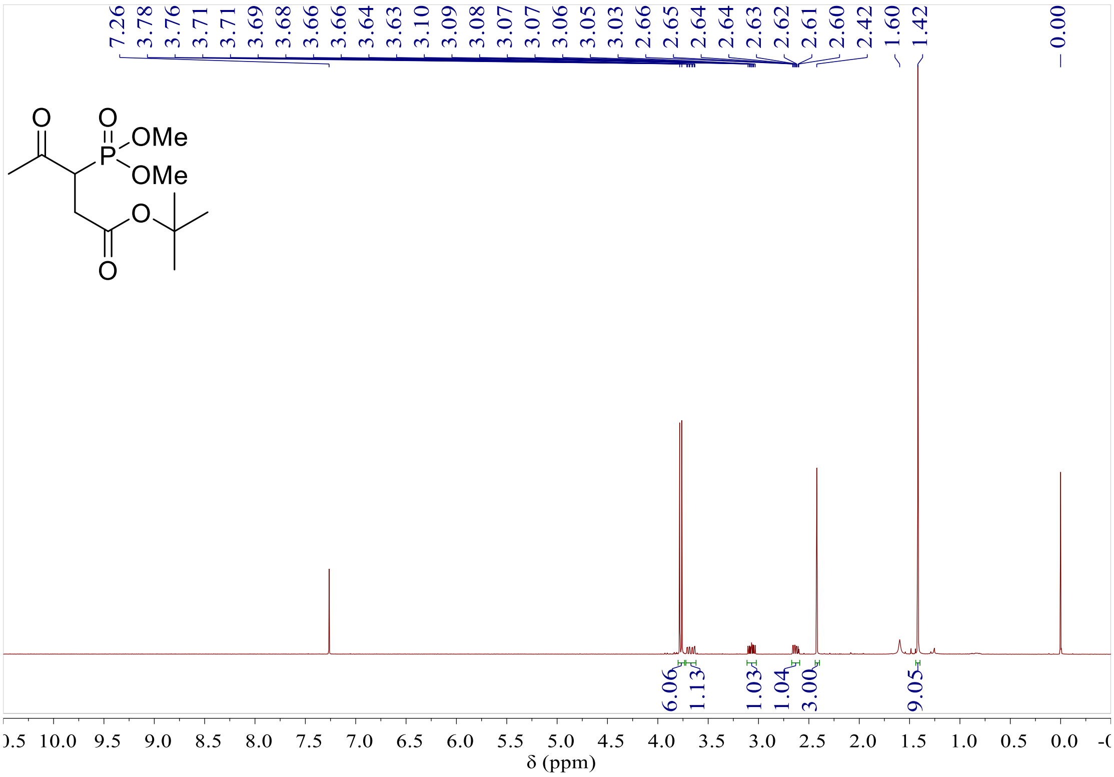

^{1}H NMR spectrum of **1p** in $CDCl_3$ (500 MHz)

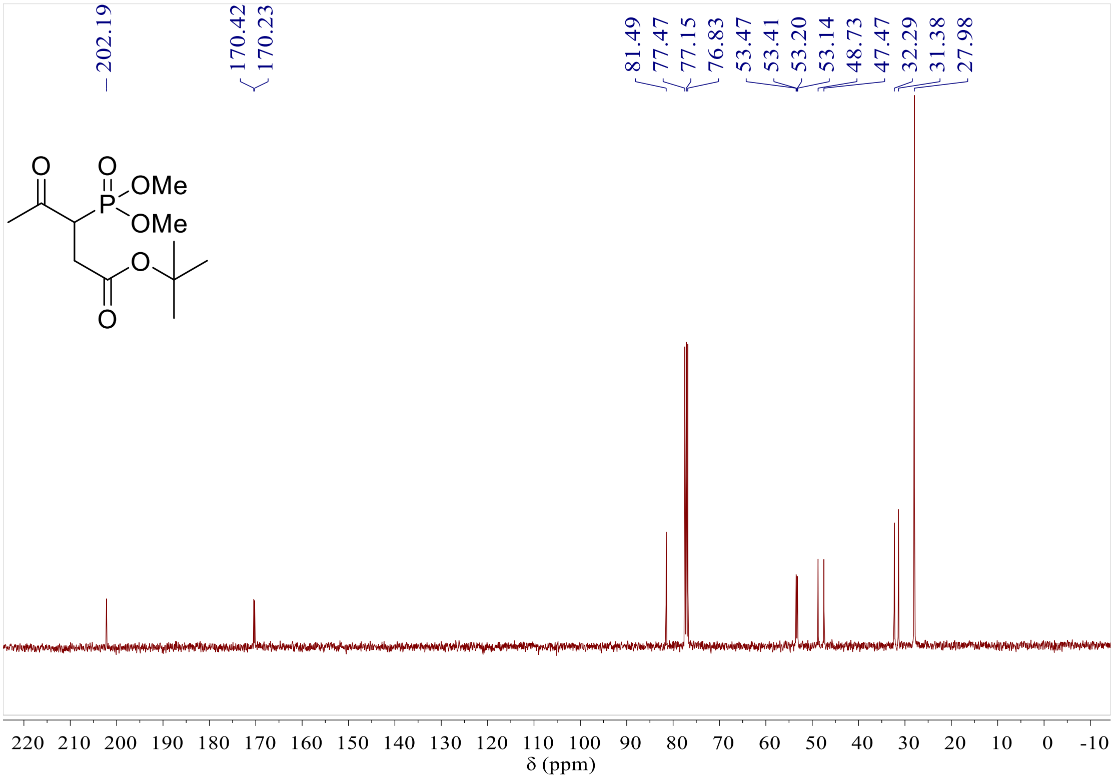

$^{13}C\{^{1}H\}$ NMR spectrum of **1p** in $CDCl_3$ (100 MHz)

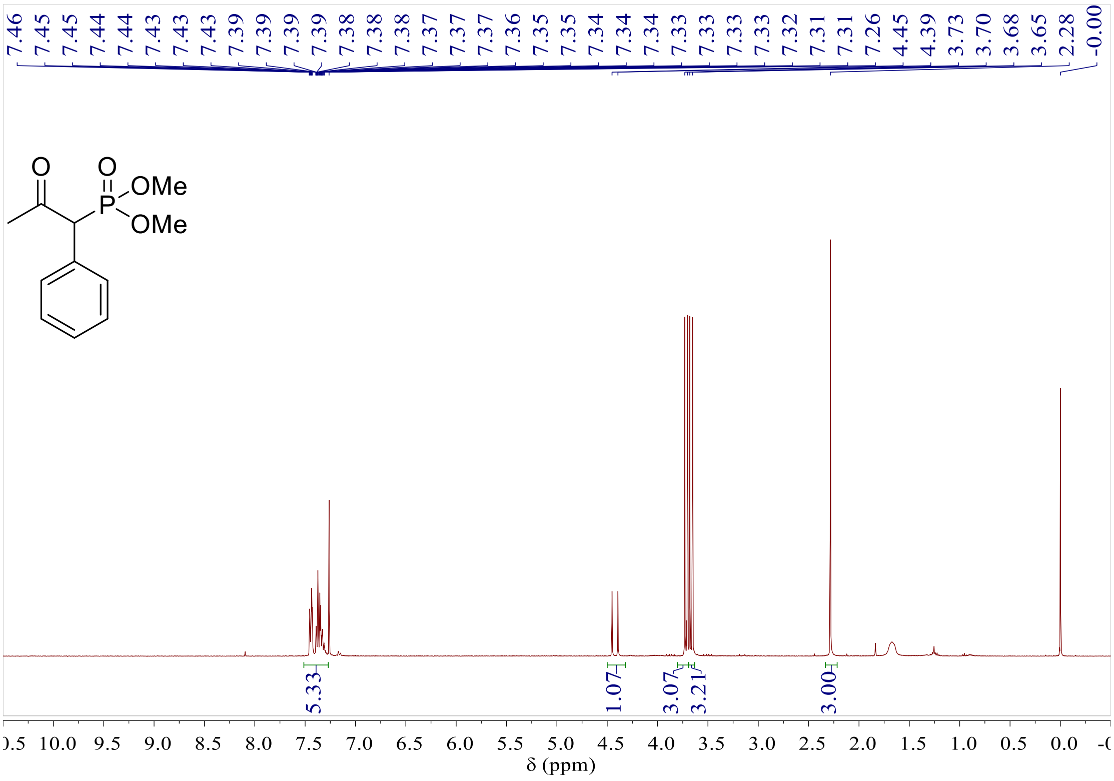

^{1}H NMR spectrum of **1q** in $CDCl_3$ (400 MHz)

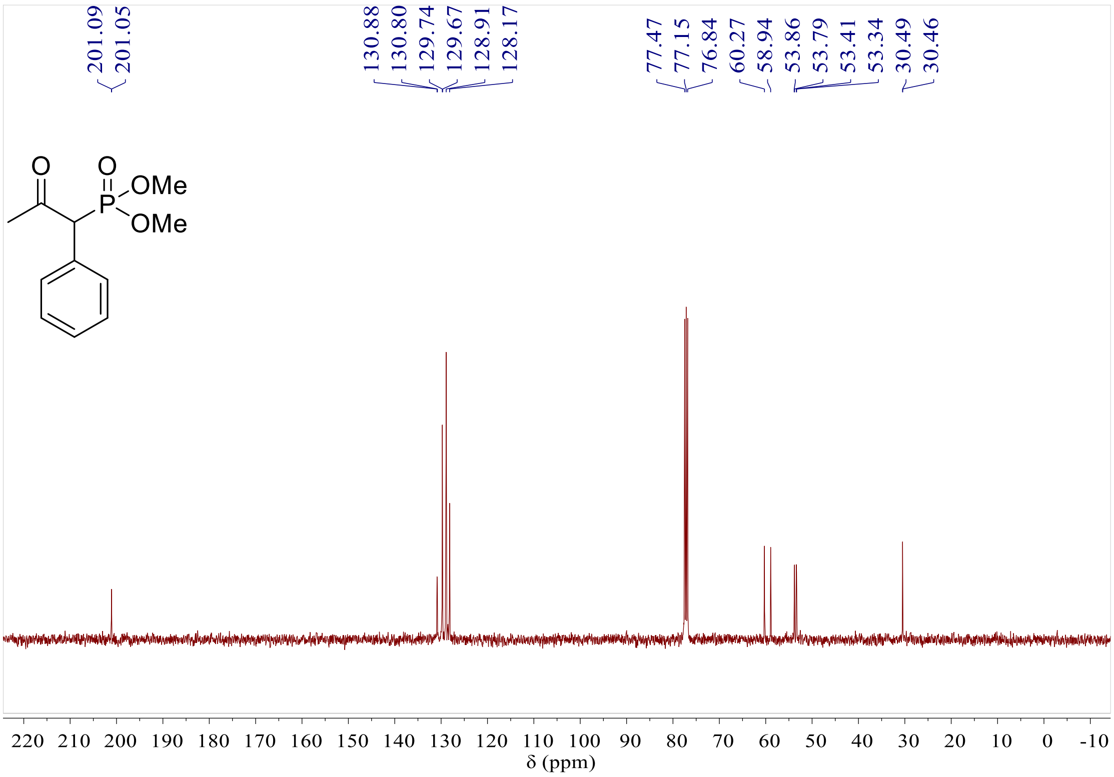

$^{13}C\{^{1}H\}$ NMR spectrum of **1q** in $CDCl_3$ (100 MHz)

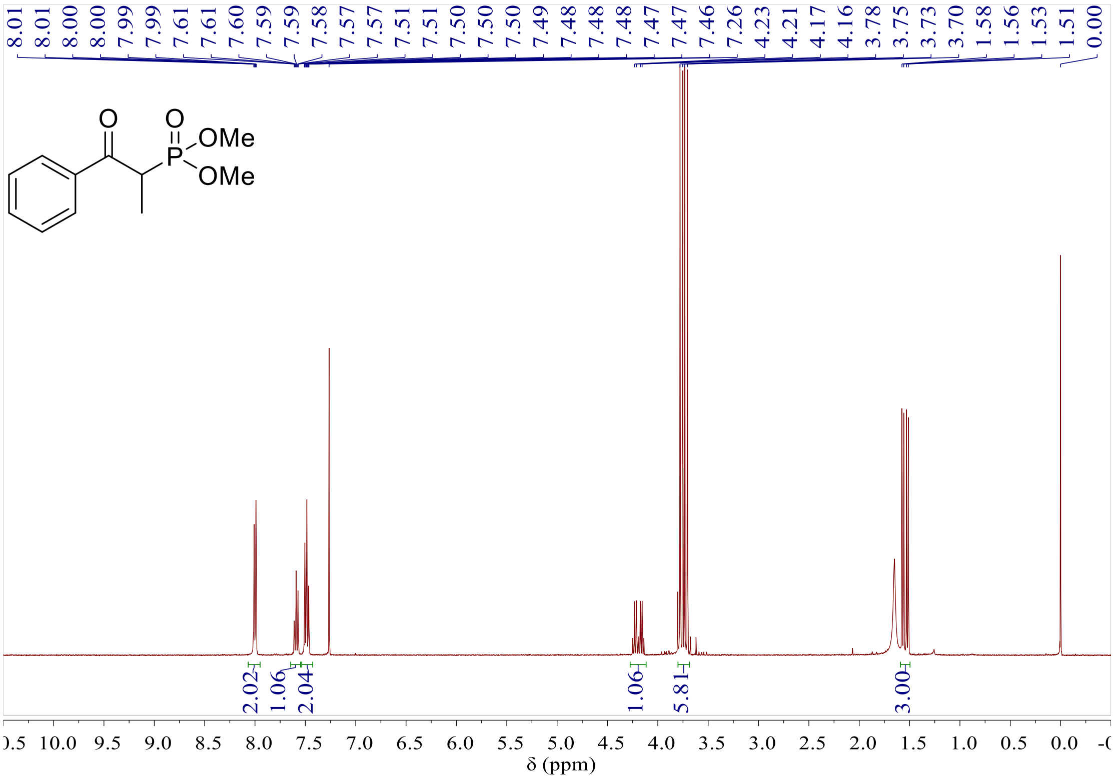

^{1}H NMR spectrum of **1r** in $CDCl_3$ (400 MHz)

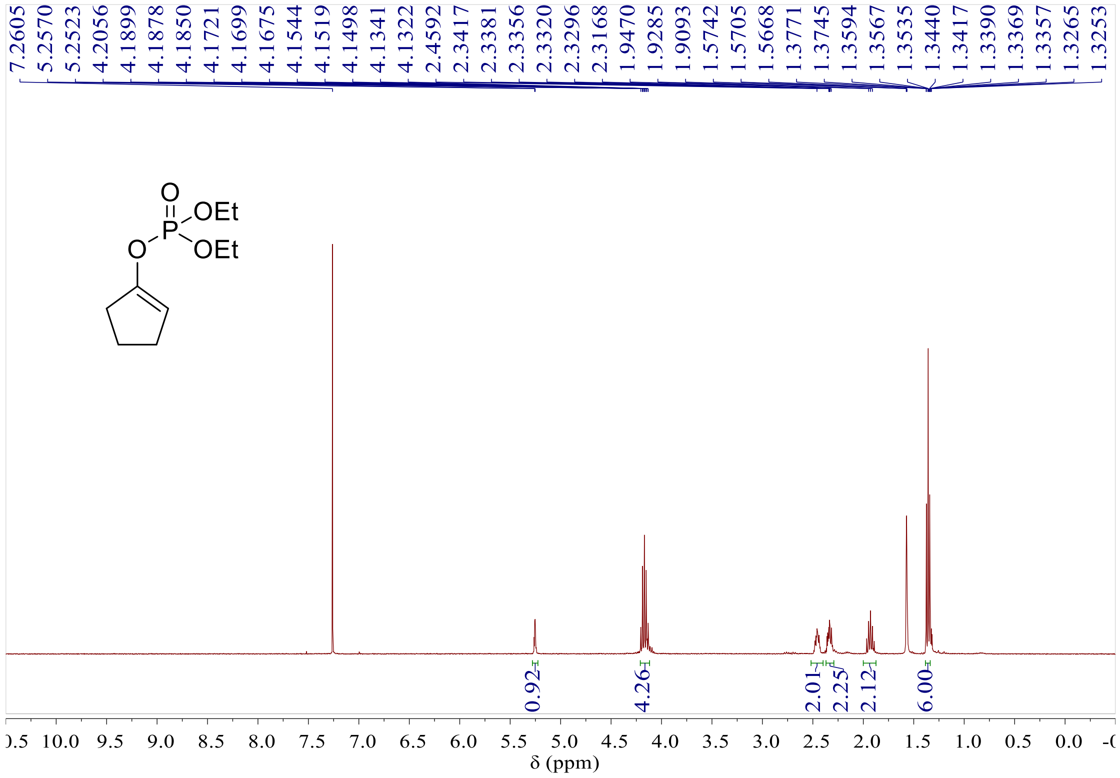

^{1}H NMR spectrum of **S2s** in $CDCl_3$ (400 MHz)

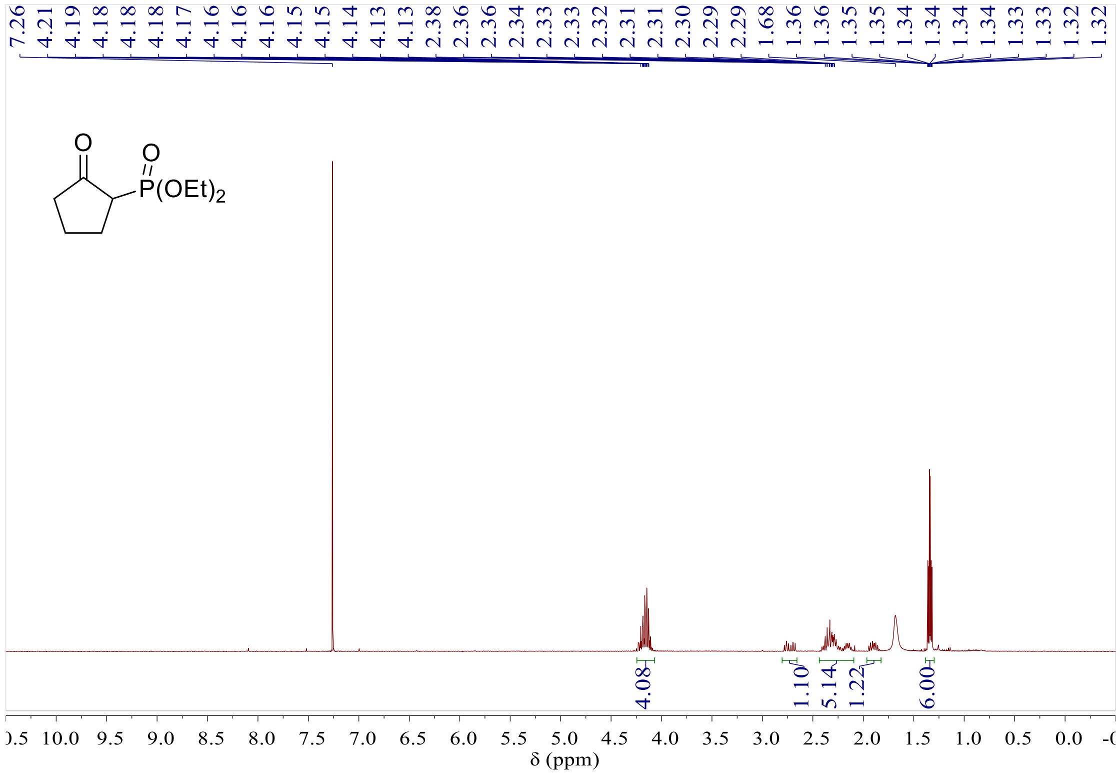

^{1}H NMR spectrum of **1s** in $CDCl_3$ (400 MHz)

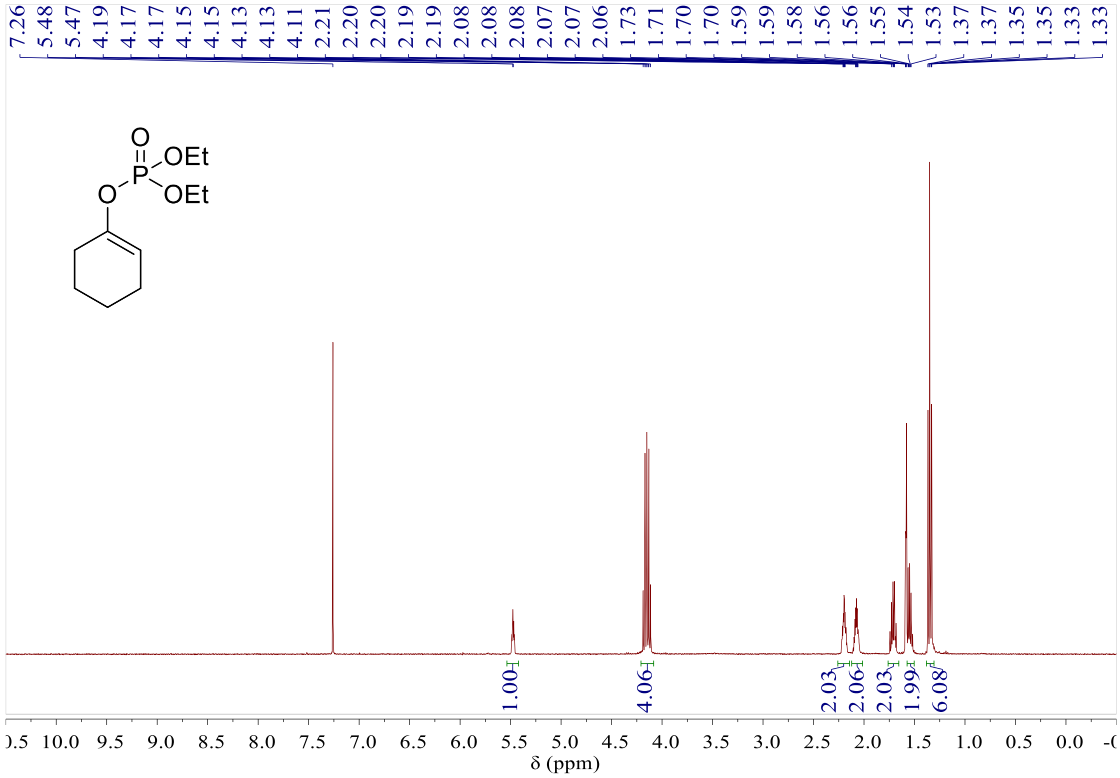

^{1}H NMR spectrum of **S2t** in $CDCl_3$ (400 MHz)

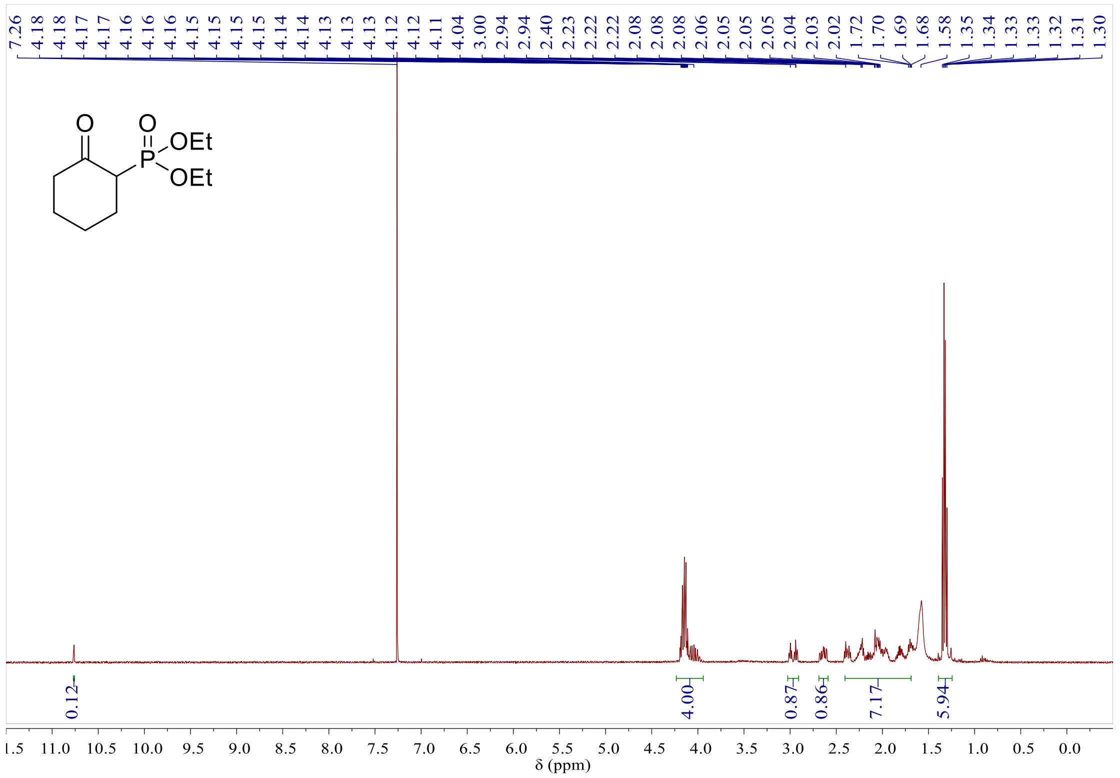

^{1}H NMR spectrum of **1t** in $CDCl_3$ (400 MHz)

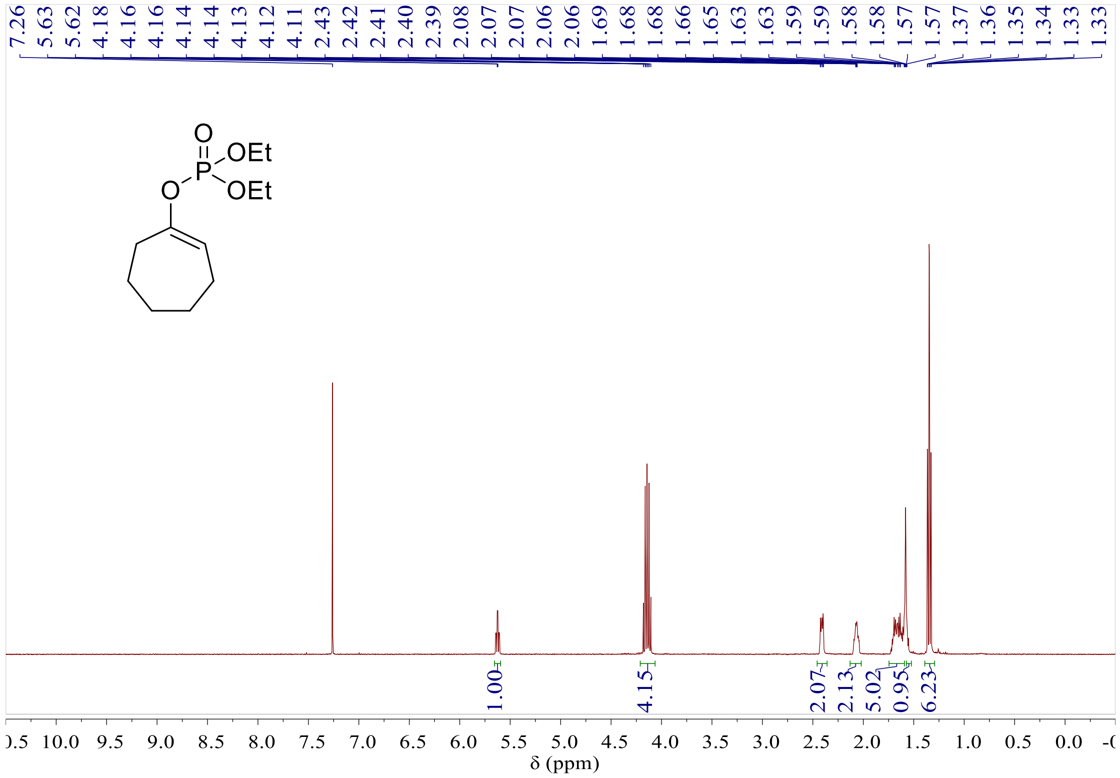

^{1}H NMR spectrum of **S2u** in $CDCl_3$ (400 MHz)

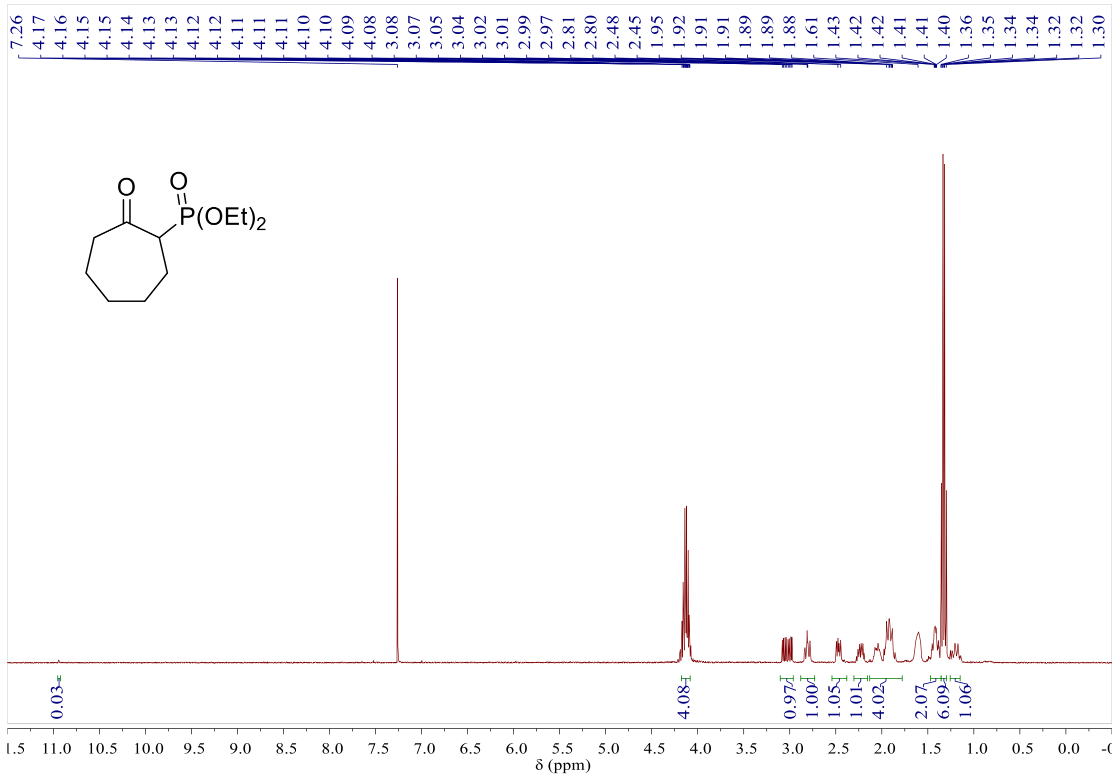

^{1}H NMR spectrum of **1u** in $CDCl_3$ (400 MHz)

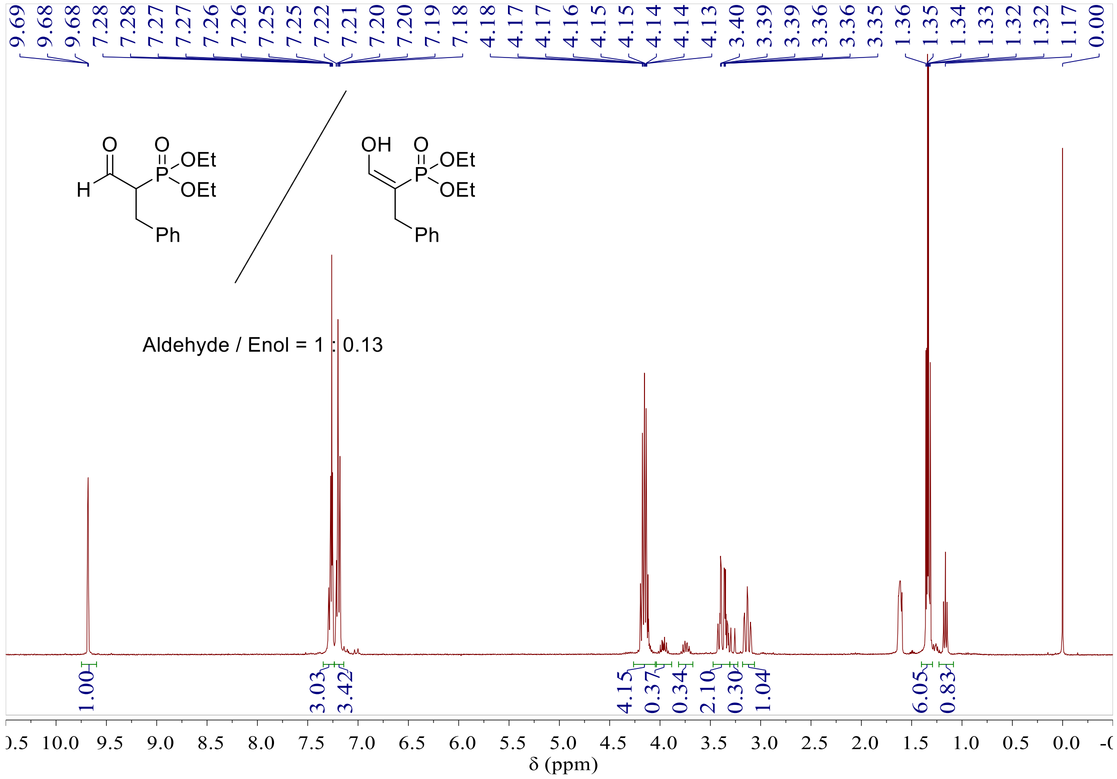

^{1}H NMR spectrum of **1v** in $CDCl_3$ (400 MHz)

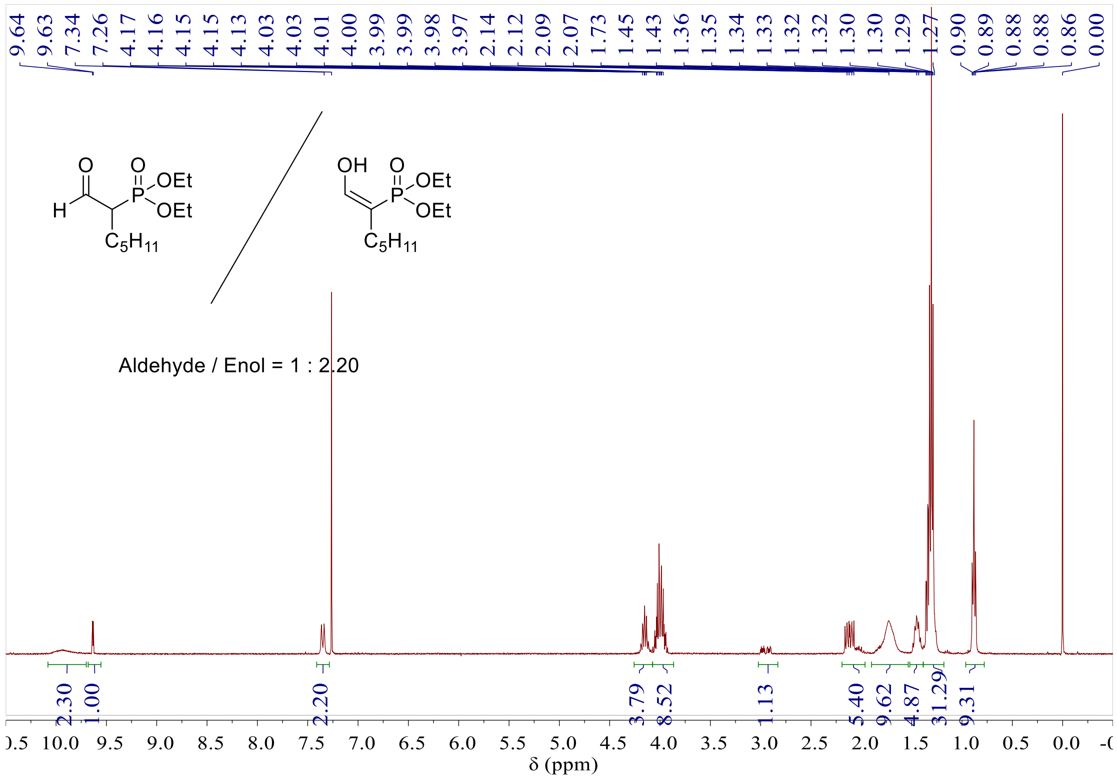

^{1}H NMR spectrum of **1w** in $CDCl_3$ (400 MHz)

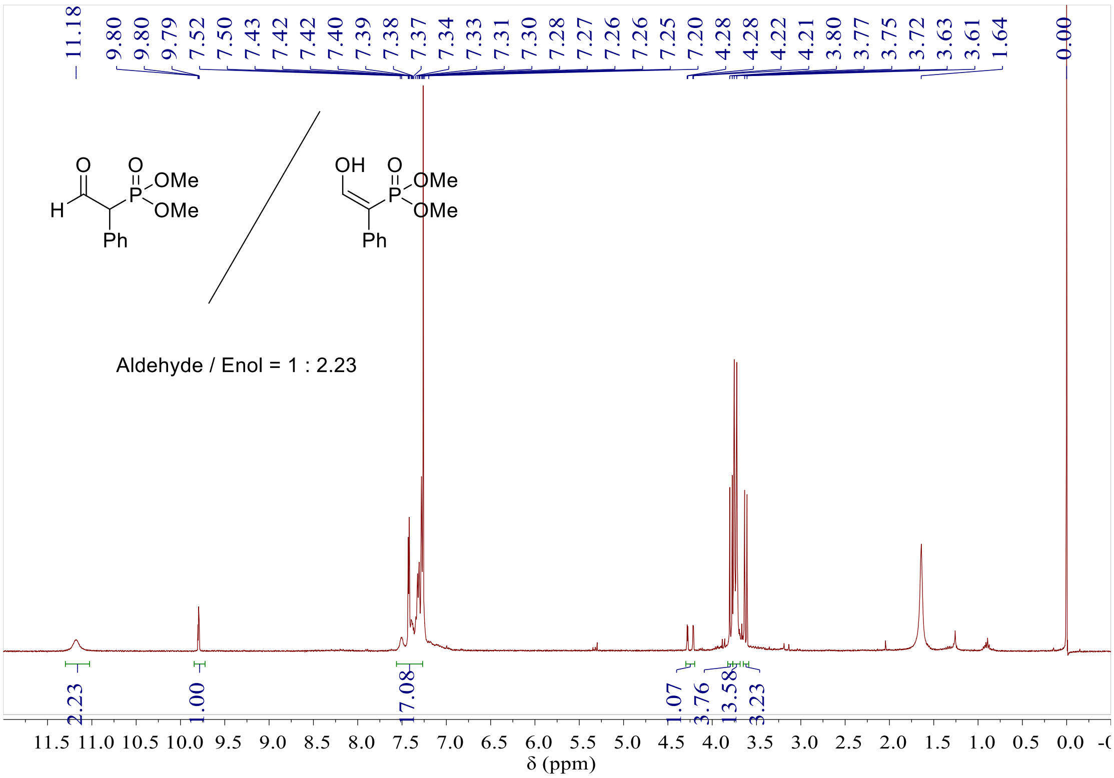

^{1}H NMR spectrum of **1x** in $CDCl_3$ (400 MHz)

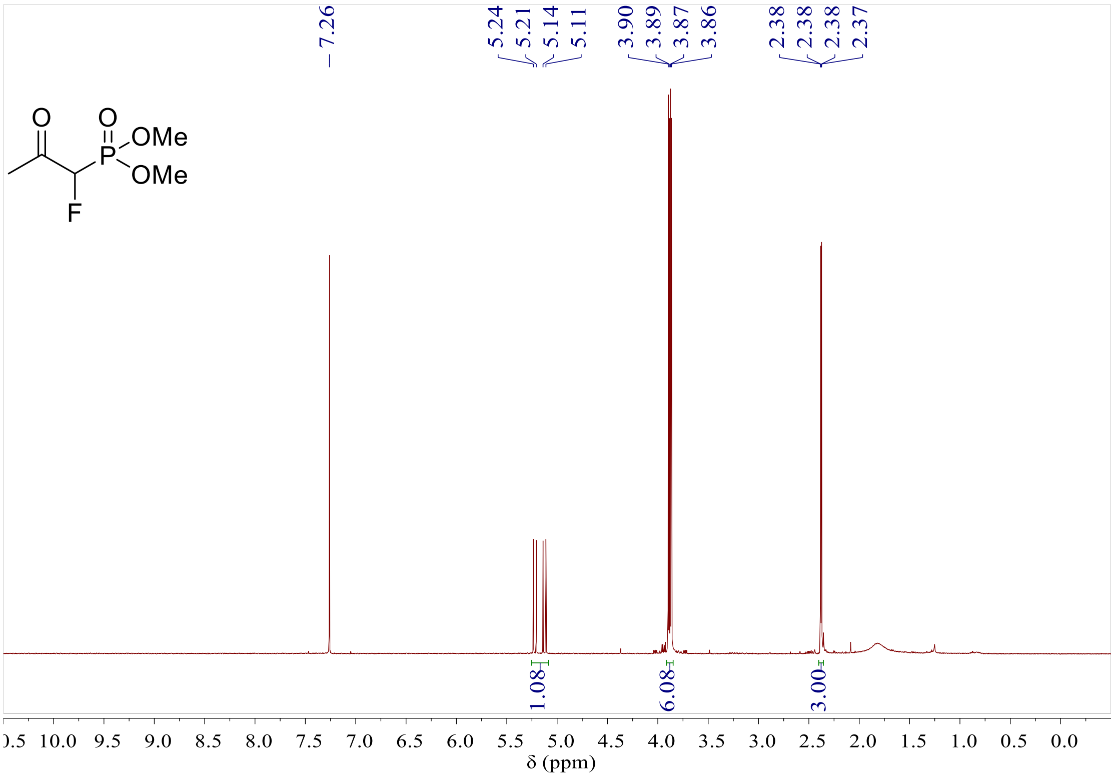

^{1}H NMR spectrum of **1y** in $CDCl_3$ (500 MHz)

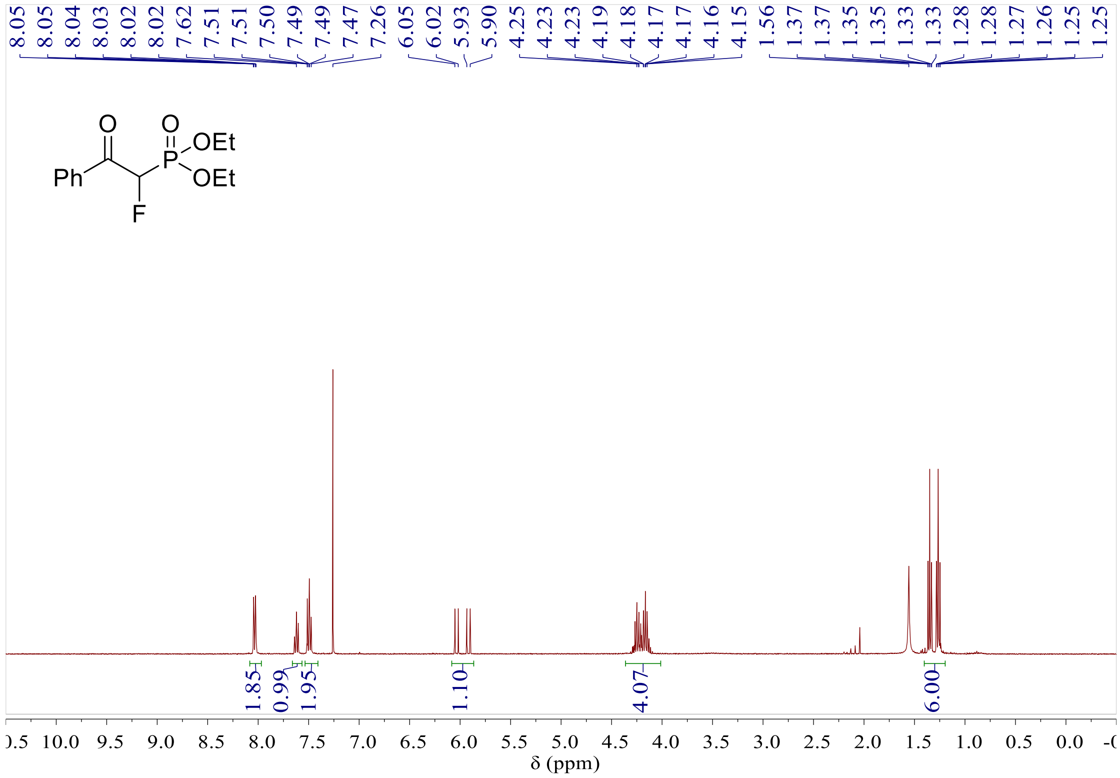

^{1}H NMR spectrum of **1z** in $CDCl_3$ (400 MHz)

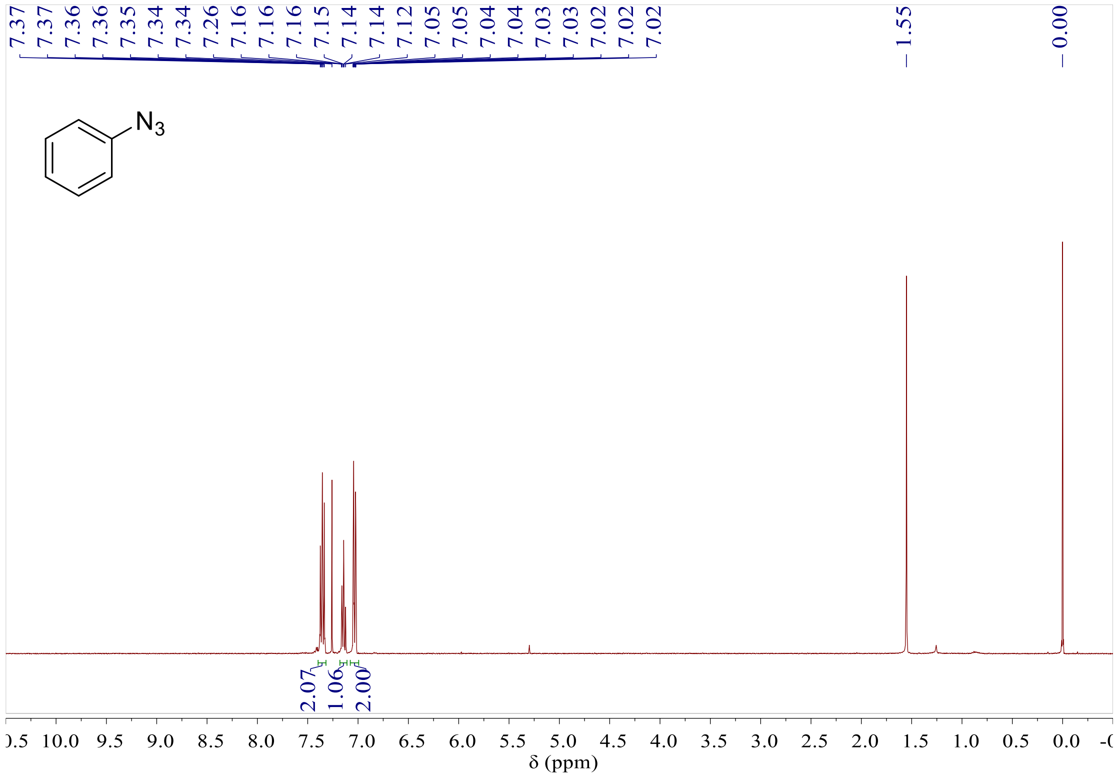

^{1}H NMR spectrum of **2a** in $CDCl_3$ (400 MHz)

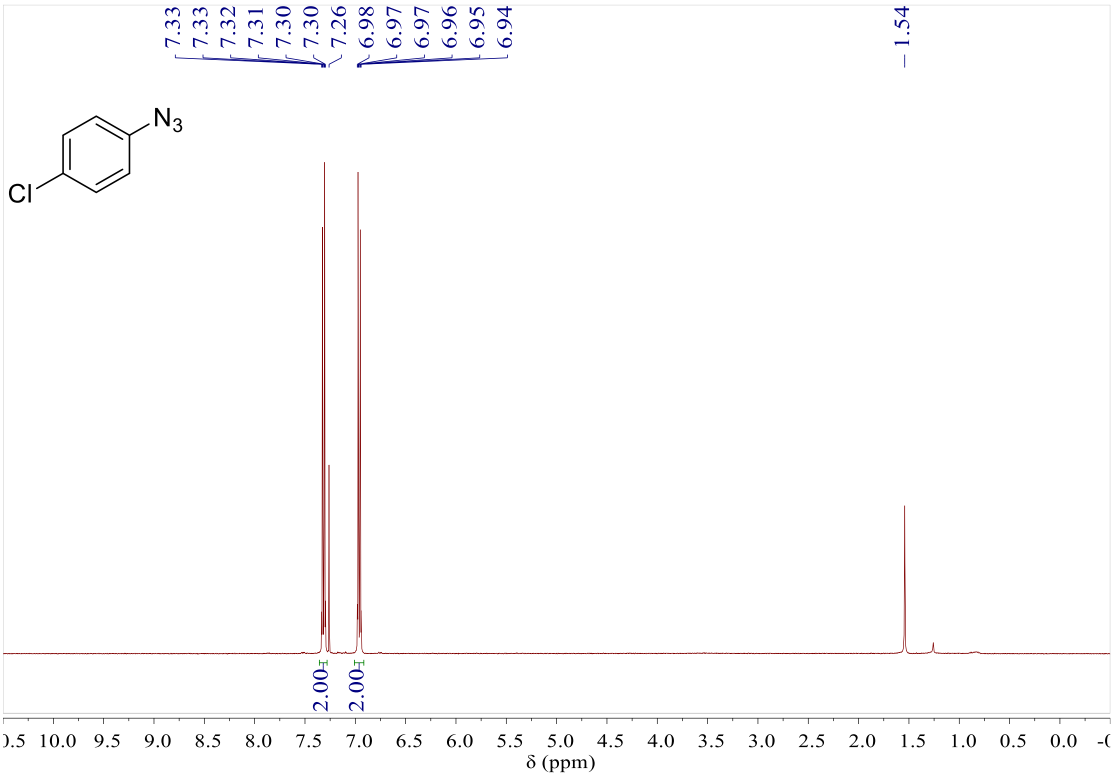

^{1}H NMR spectrum of **2b** in $CDCl_3$ (400 MHz)

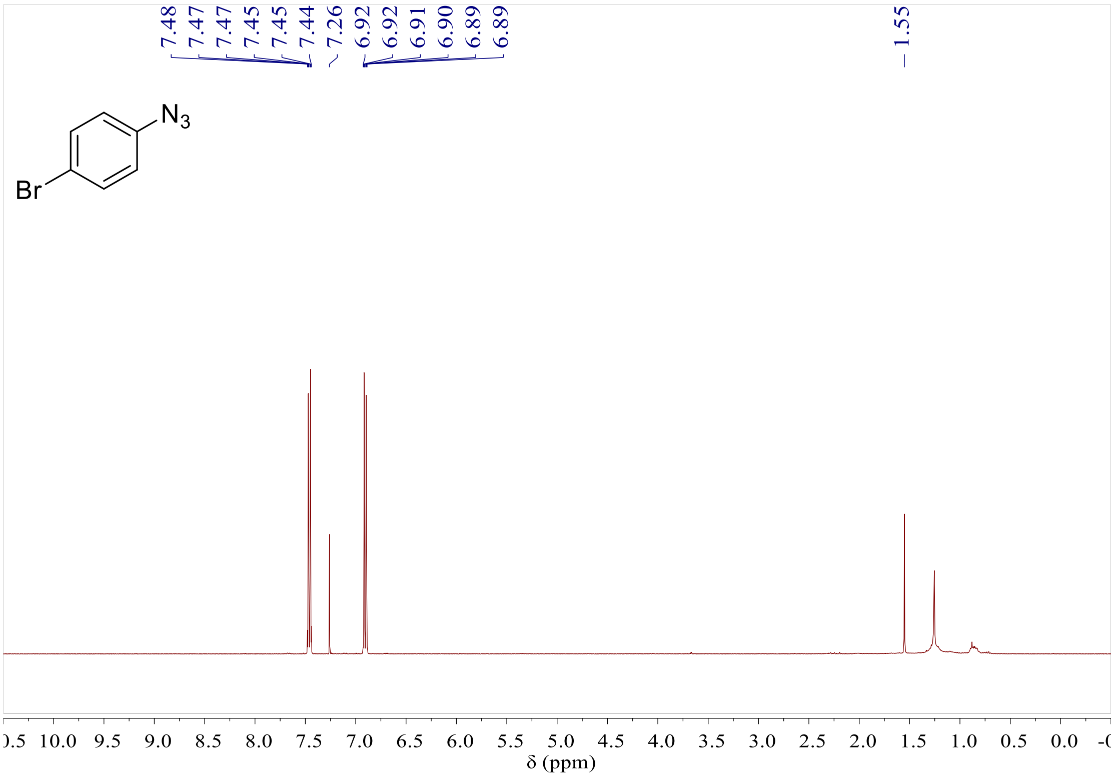

^{1}H NMR spectrum of **2c** in $CDCl_3$ (400 MHz)

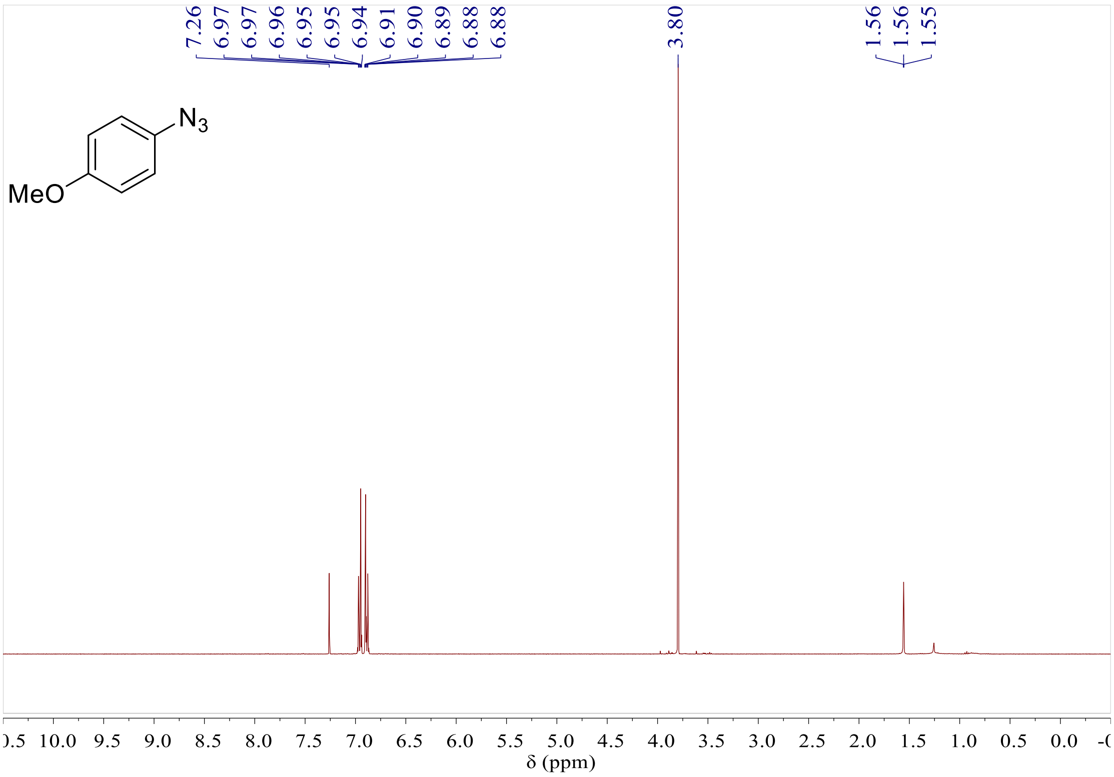

^{1}H NMR spectrum of **2d** in $CDCl_3$ (400 MHz)

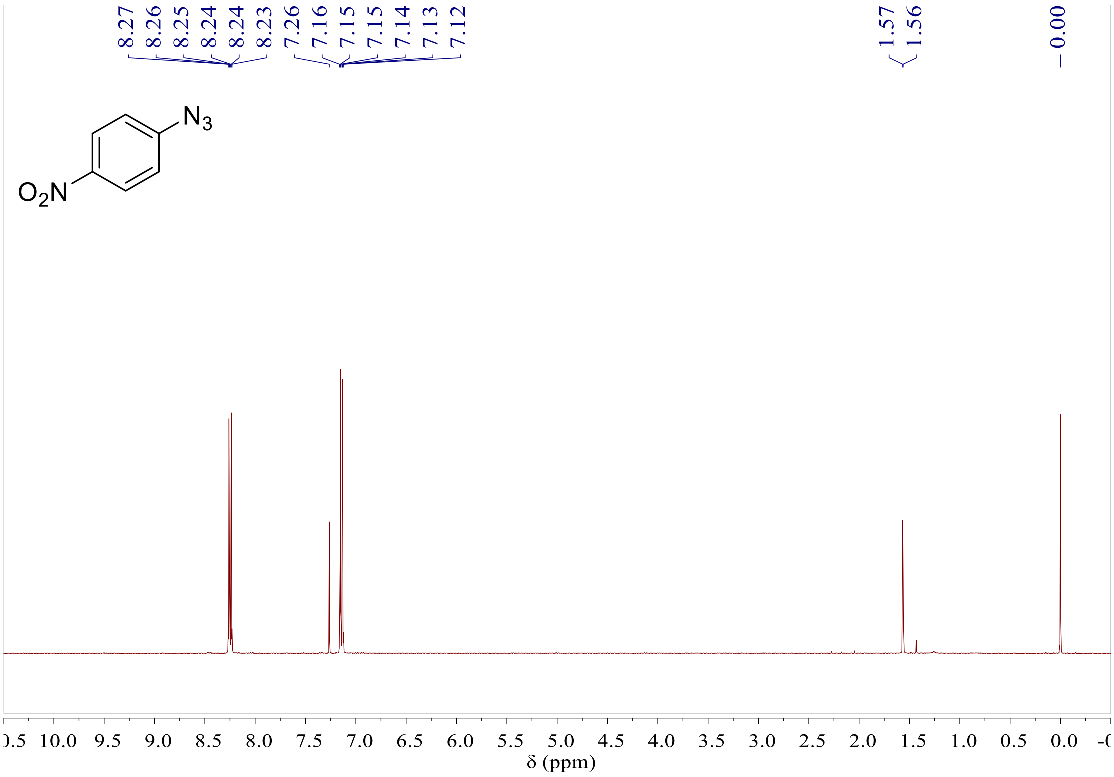

^{1}H NMR spectrum of **2e** in $CDCl_3$ (400 MHz)

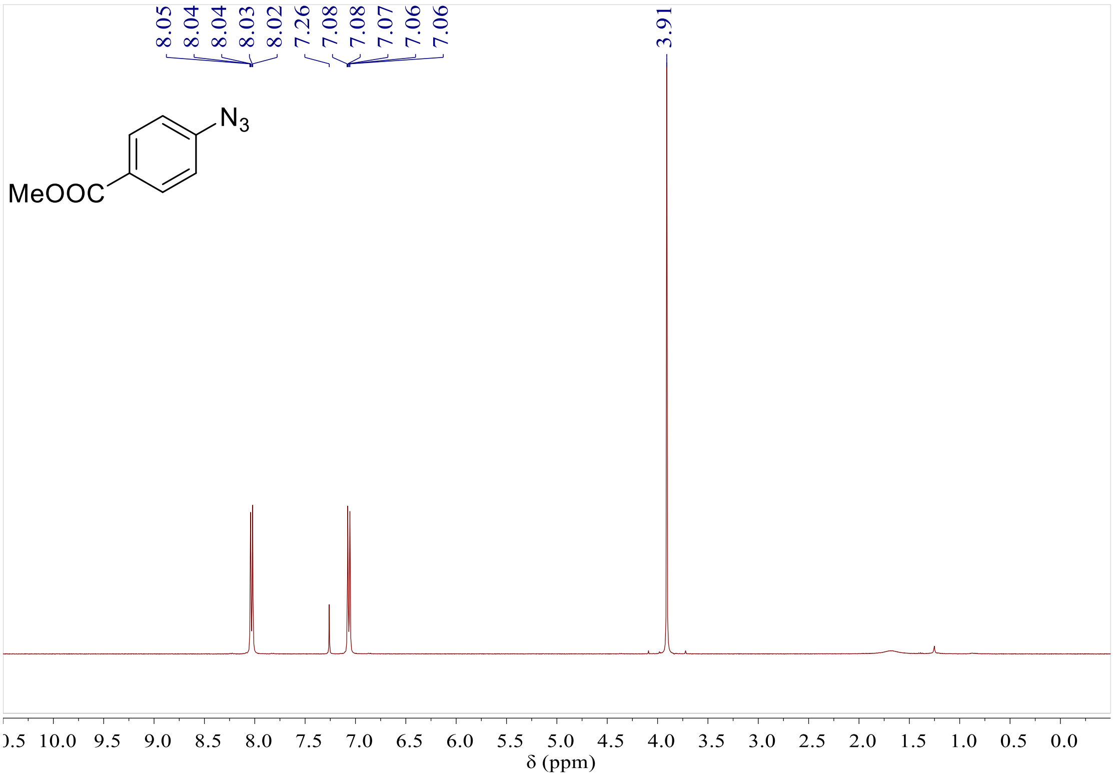

^{1}H NMR spectrum of **2f** in $CDCl_3$ (400 MHz)

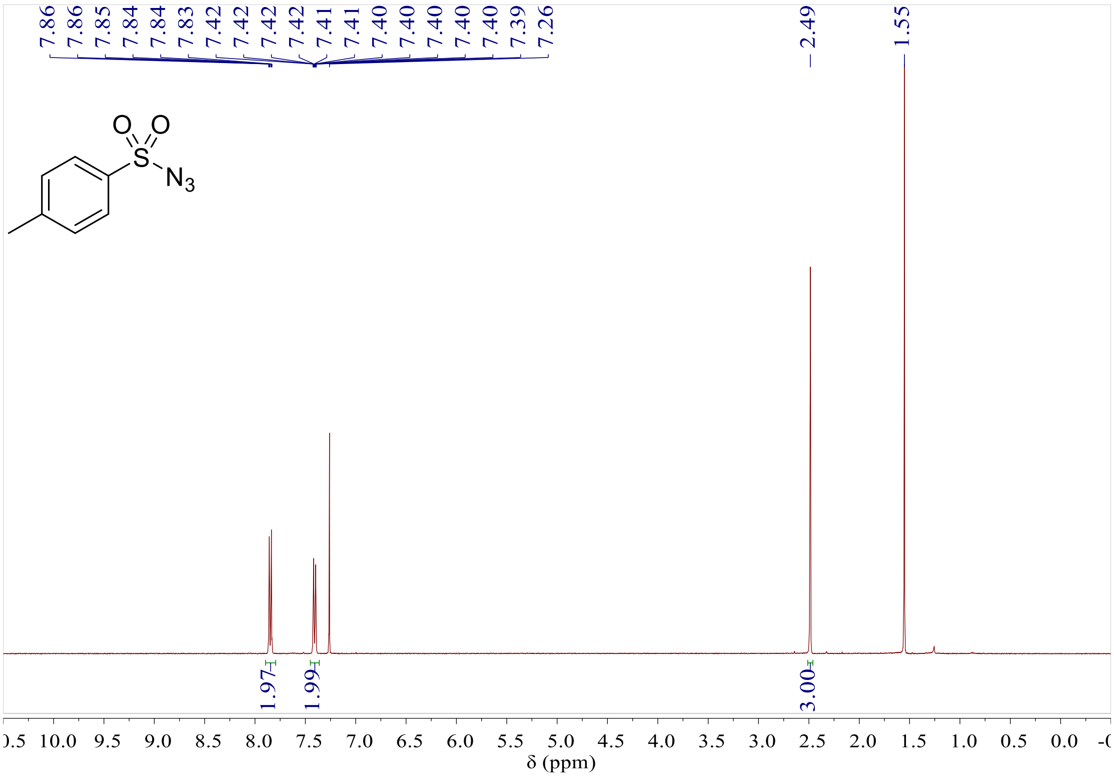

^{1}H NMR spectrum of **2g** in $CDCl_3$ (400 MHz)

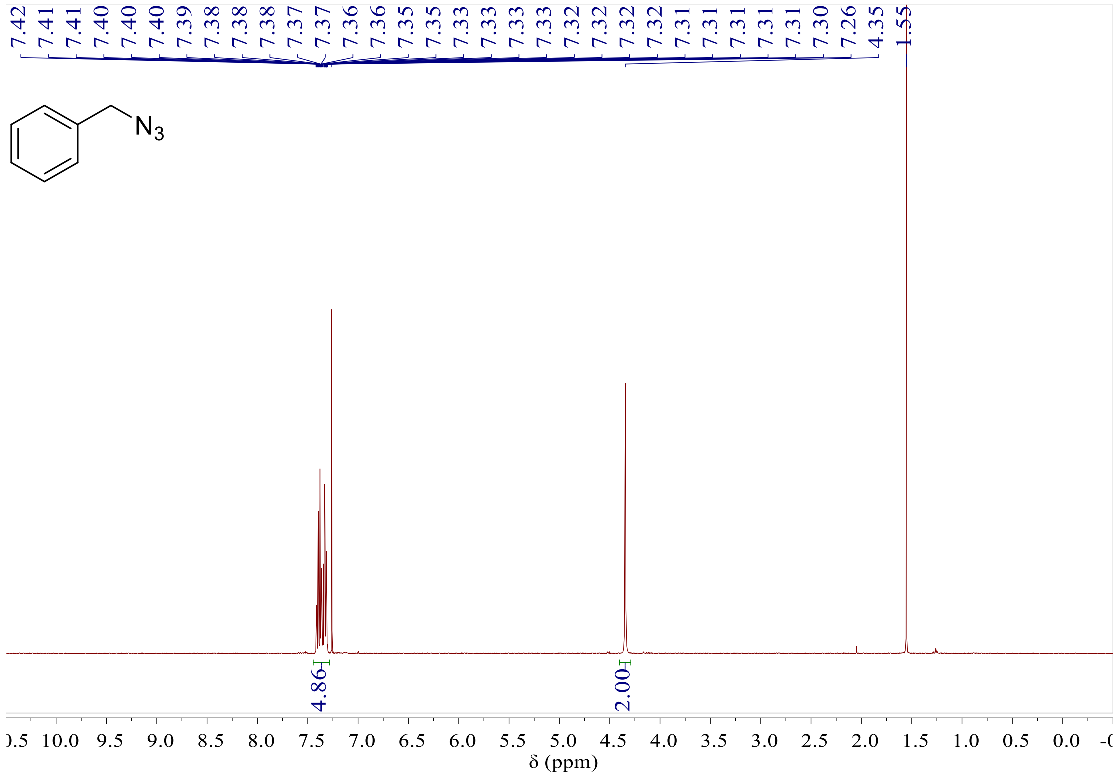

^{1}H NMR spectrum of **2h** in $CDCl_3$ (400 MHz)

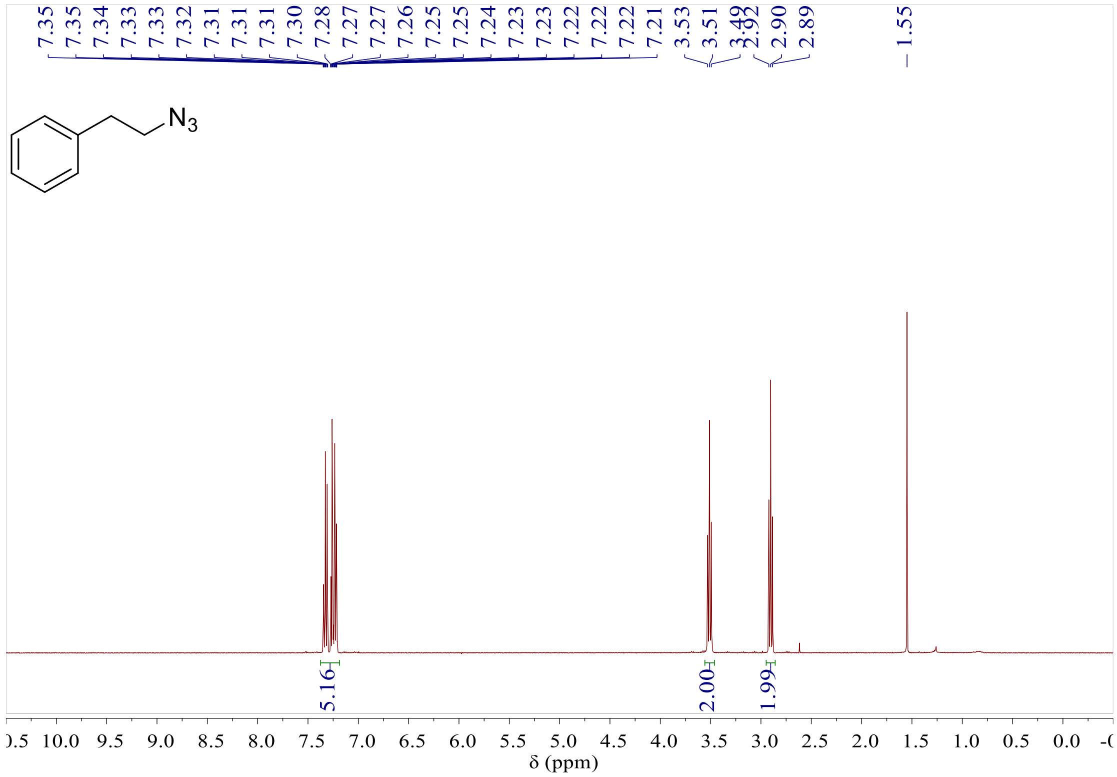

^{1}H NMR spectrum of **2i** in $CDCl_3$ (400 MHz)

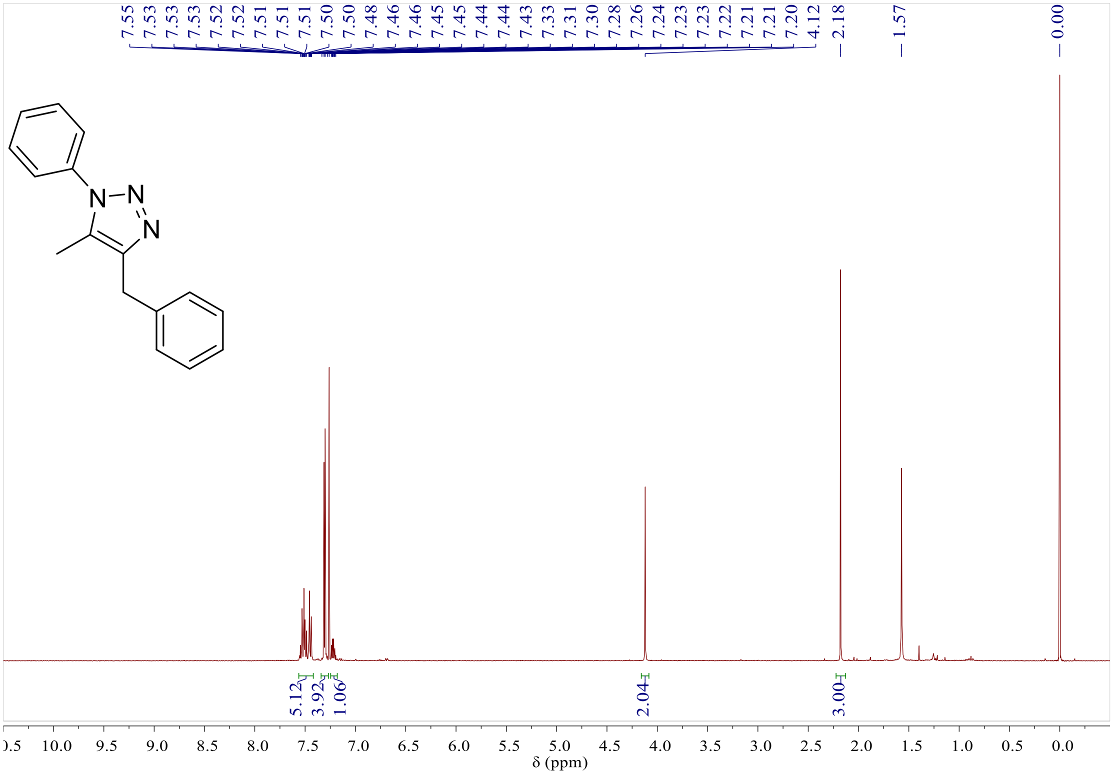

^{1}H NMR spectrum of **3aa** in $CDCl_3$ (400 MHz)

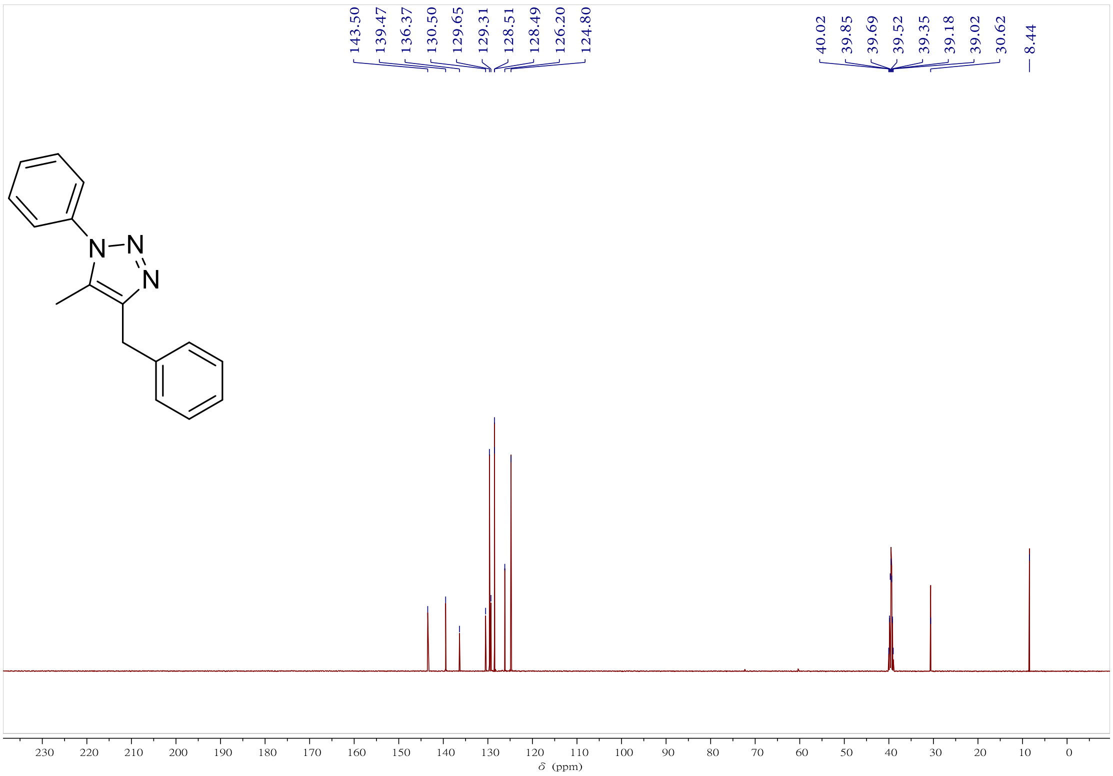

^{13}C{^{1}H} NMR spectrum of **3aa** in DMSO-d_6 (125 MHz)

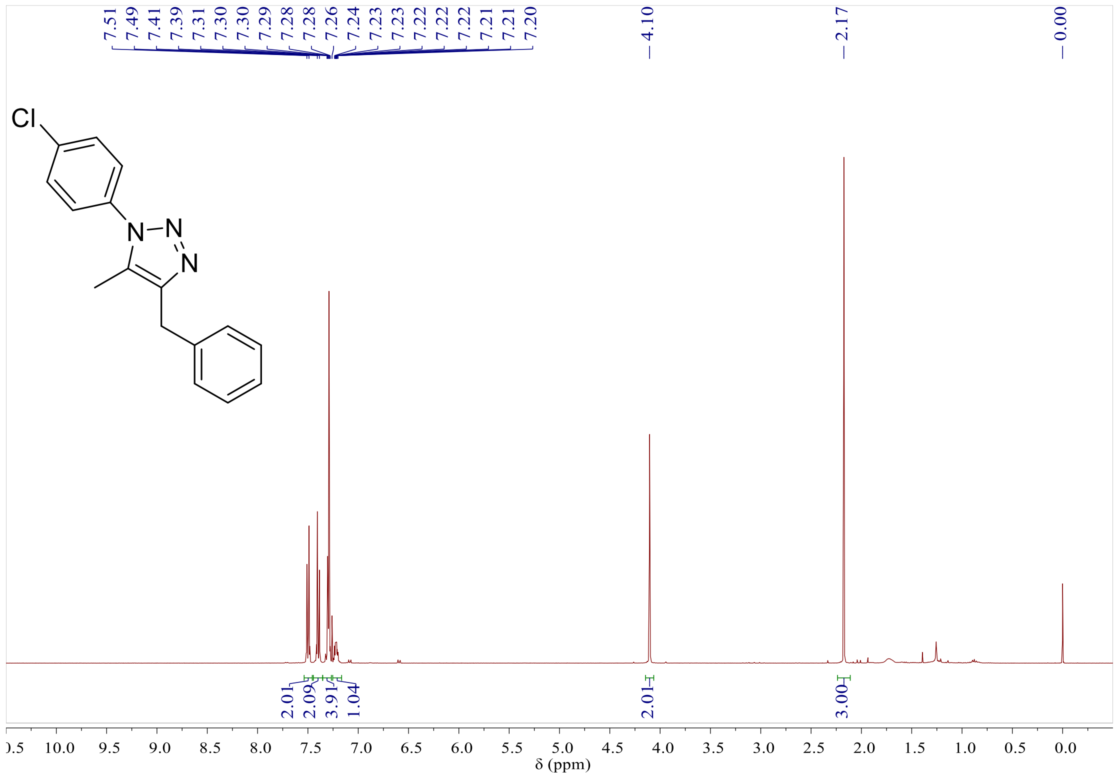

^{1}H NMR spectrum of **3ab** in $CDCl_3$ (400 MHz)

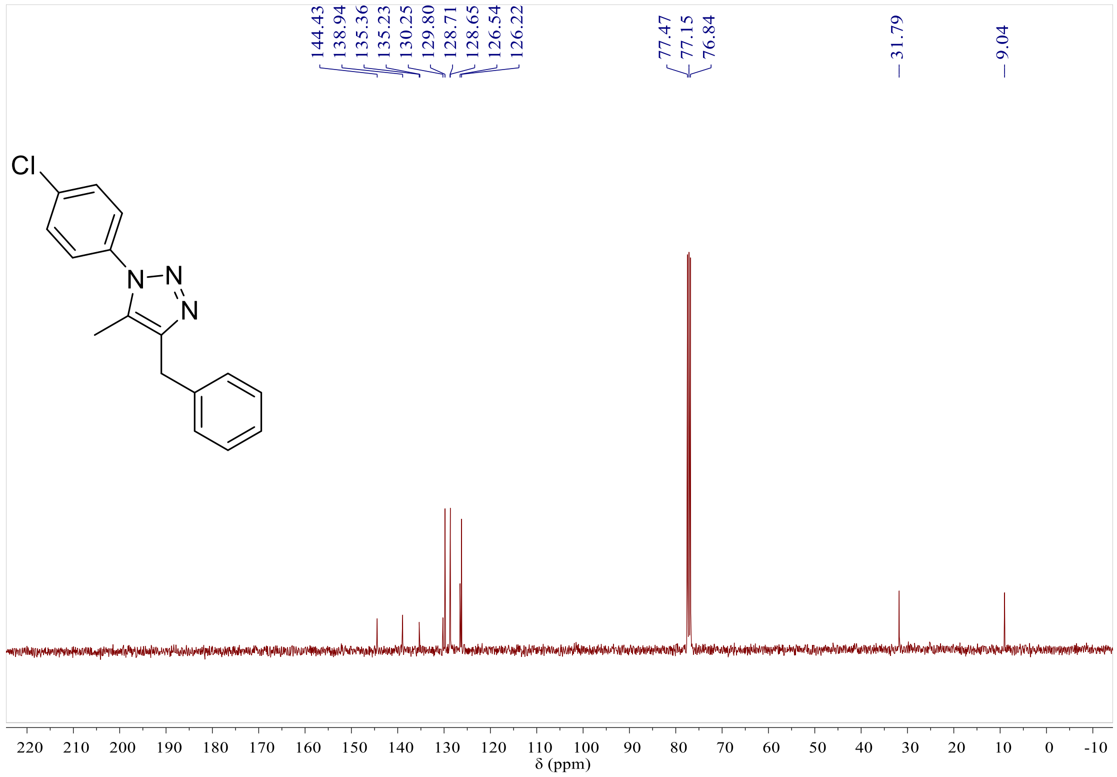

^{13}C{^{1}H} NMR spectrum of **3ab** in $CDCl_3$ (100 MHz)

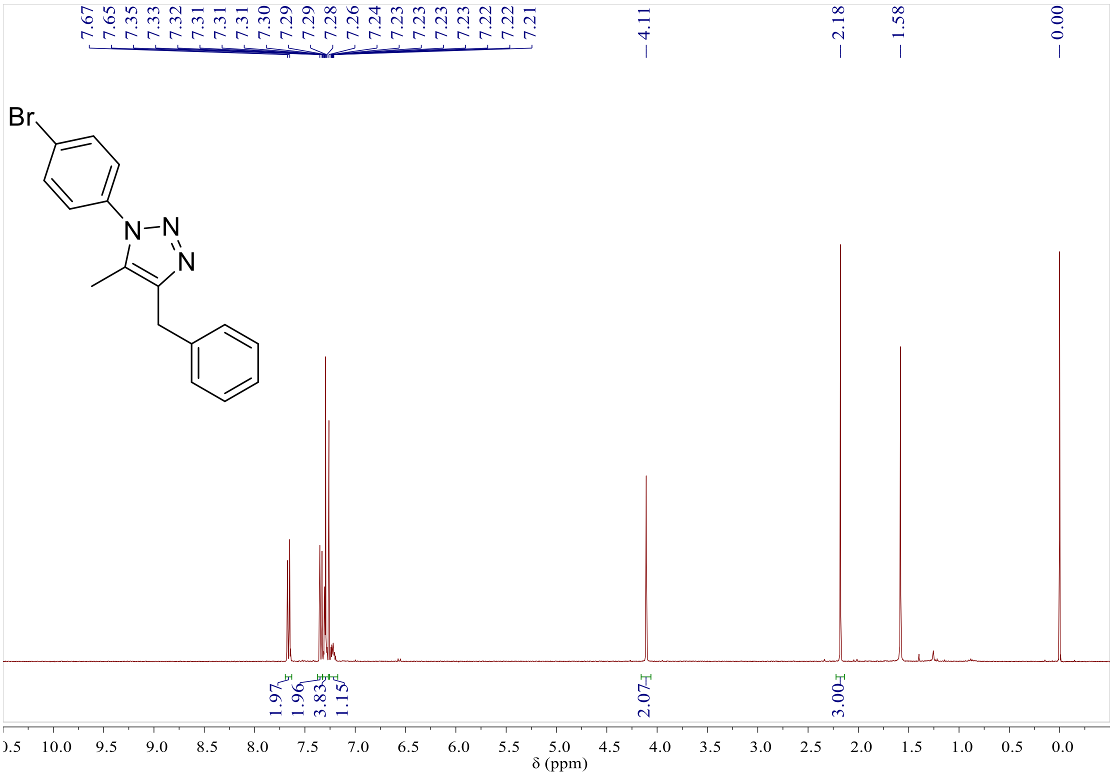

^{1}H NMR spectrum of **3ac** in $CDCl_3$ (400 MHz)

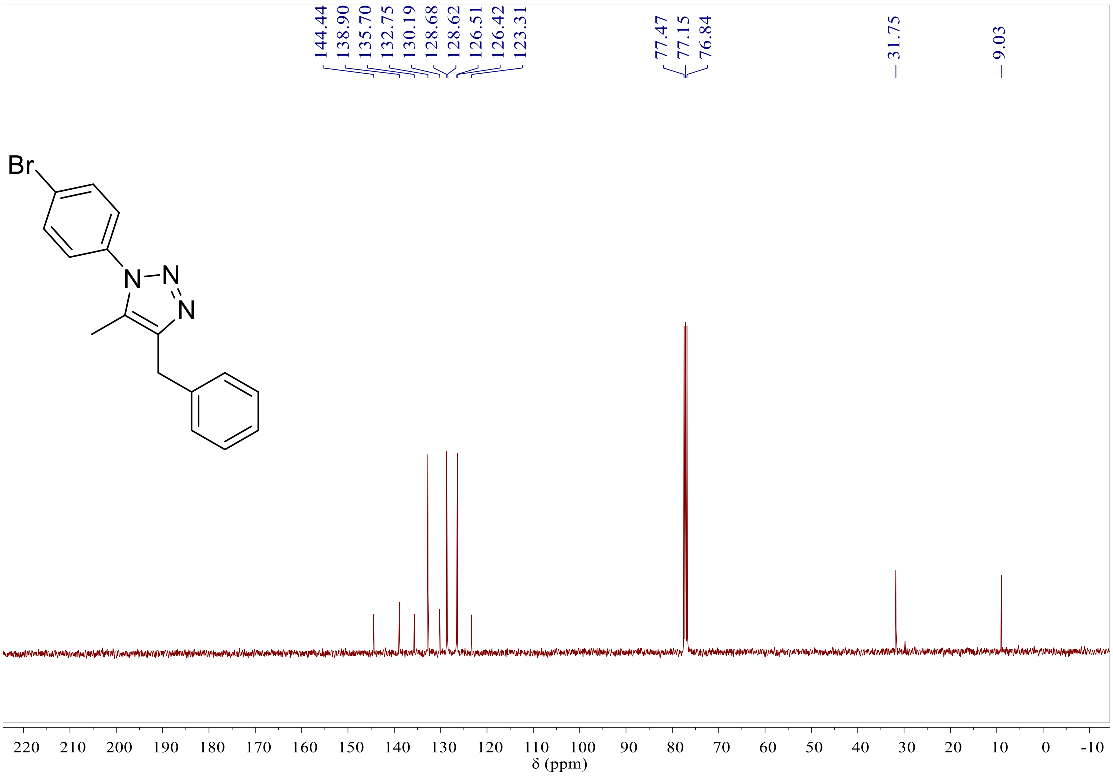

^{13}C{^{1}H} NMR spectrum of **3ac** in $CDCl_3$ (100 MHz)

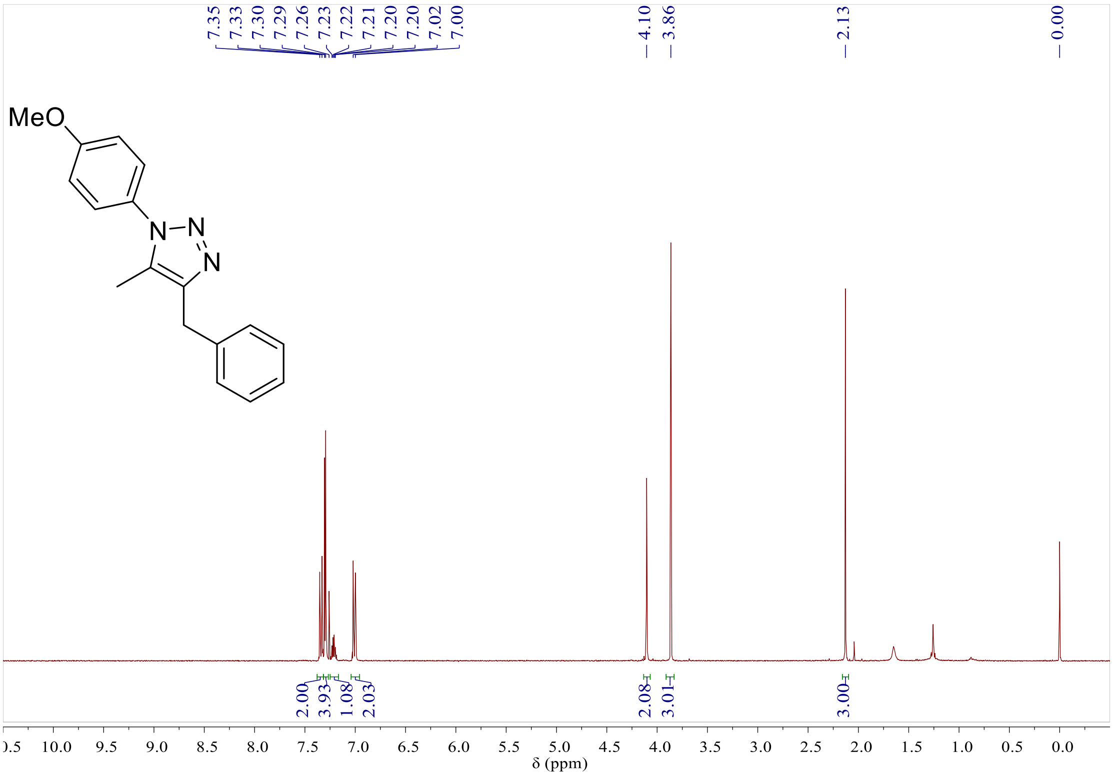

^{1}H NMR spectrum of **3ad** in $CDCl_3$ (400 MHz)

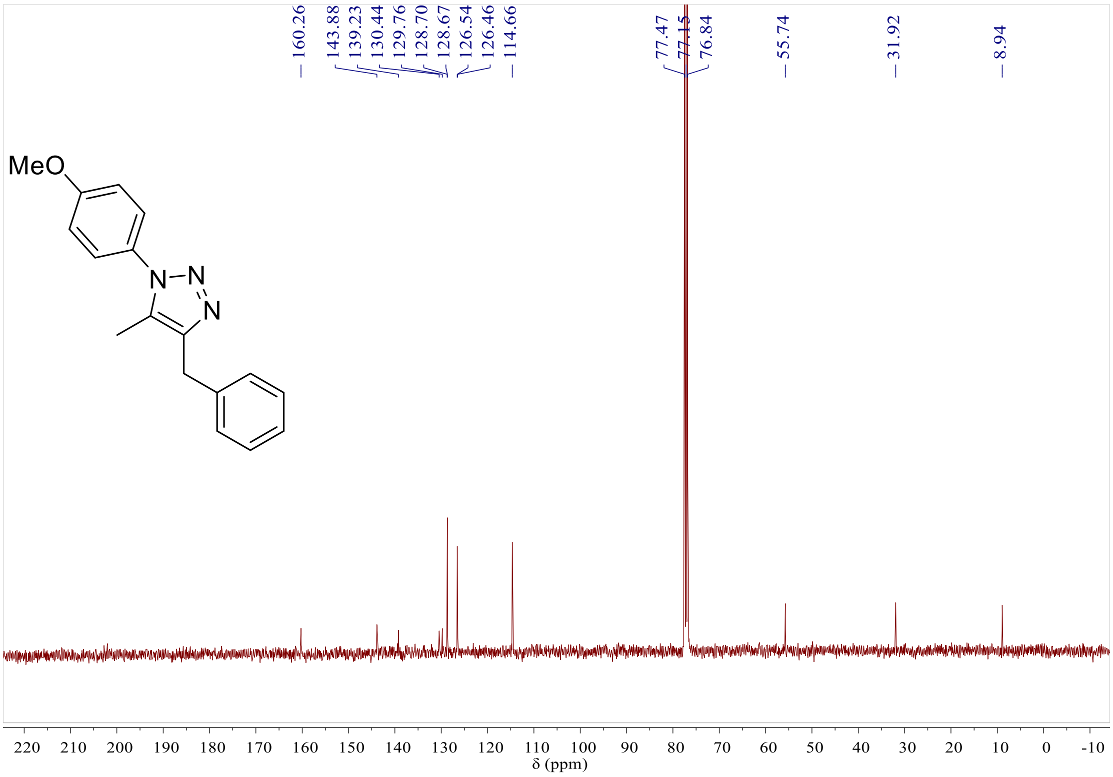

^{13}C{^{1}H} NMR spectrum of **3ad** in $CDCl_3$ (100 MHz)

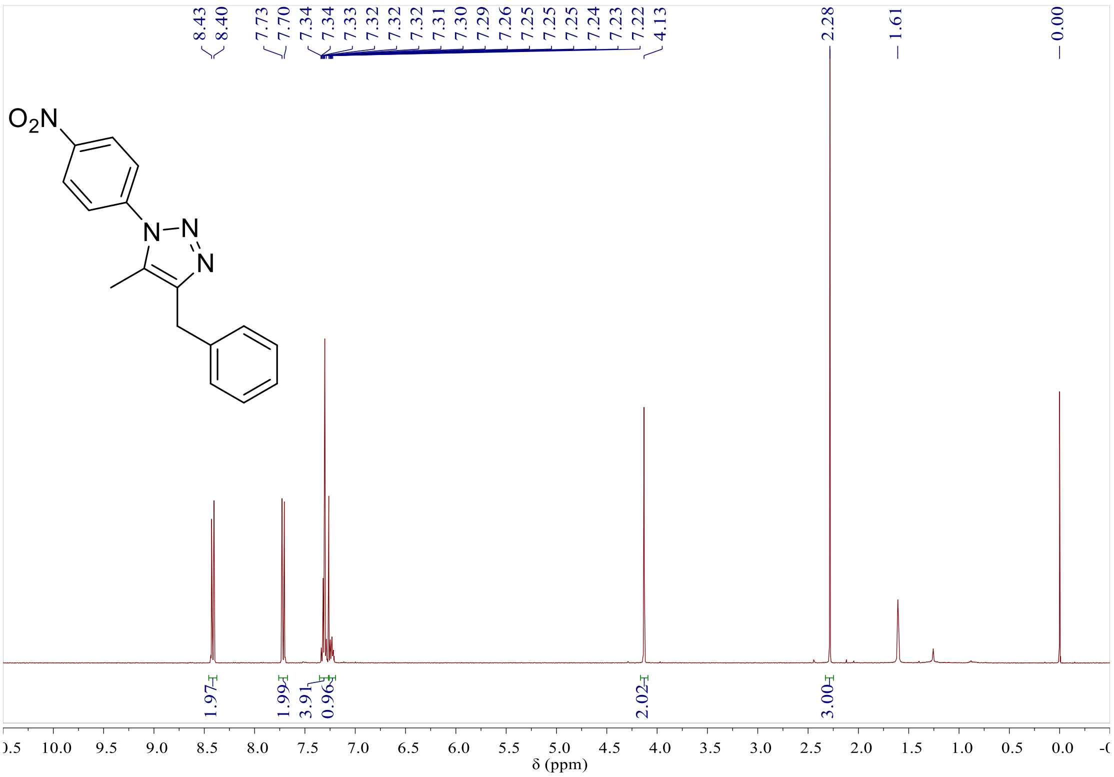

^{1}H NMR spectrum of **3ae** in $CDCl_3$ (400 MHz)

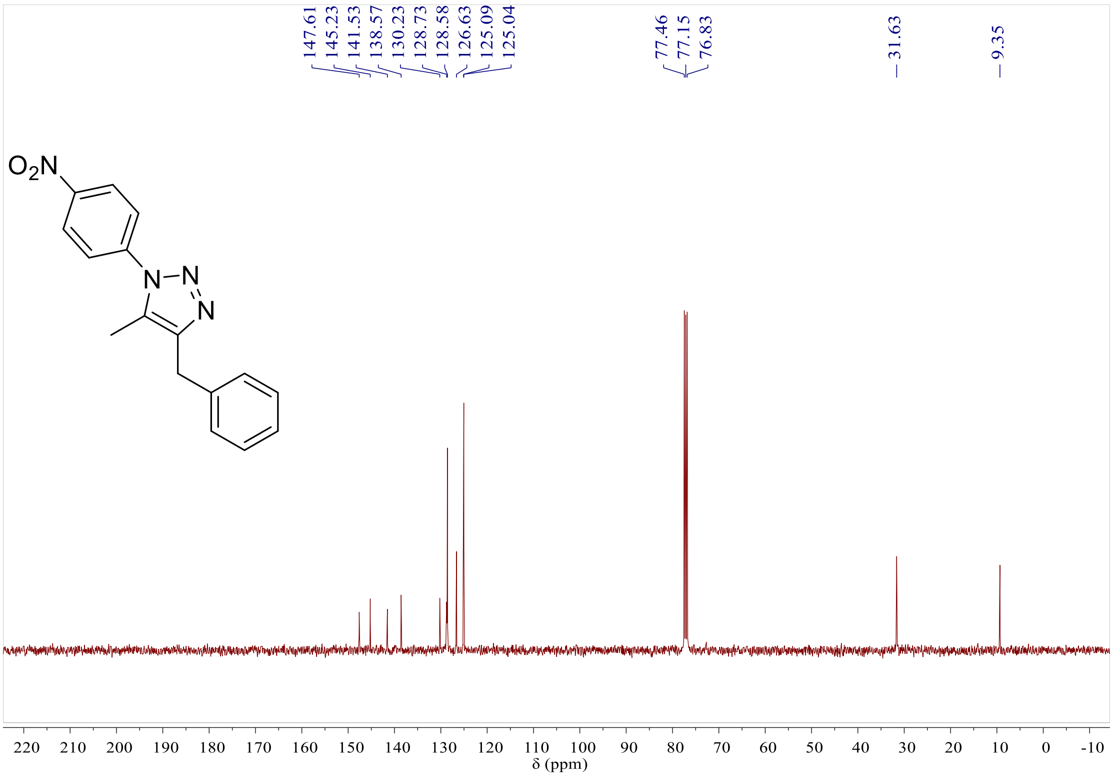

$^{13}C\{^1H\}$ NMR spectrum of **3ae** in $CDCl_3$ (100 MHz)

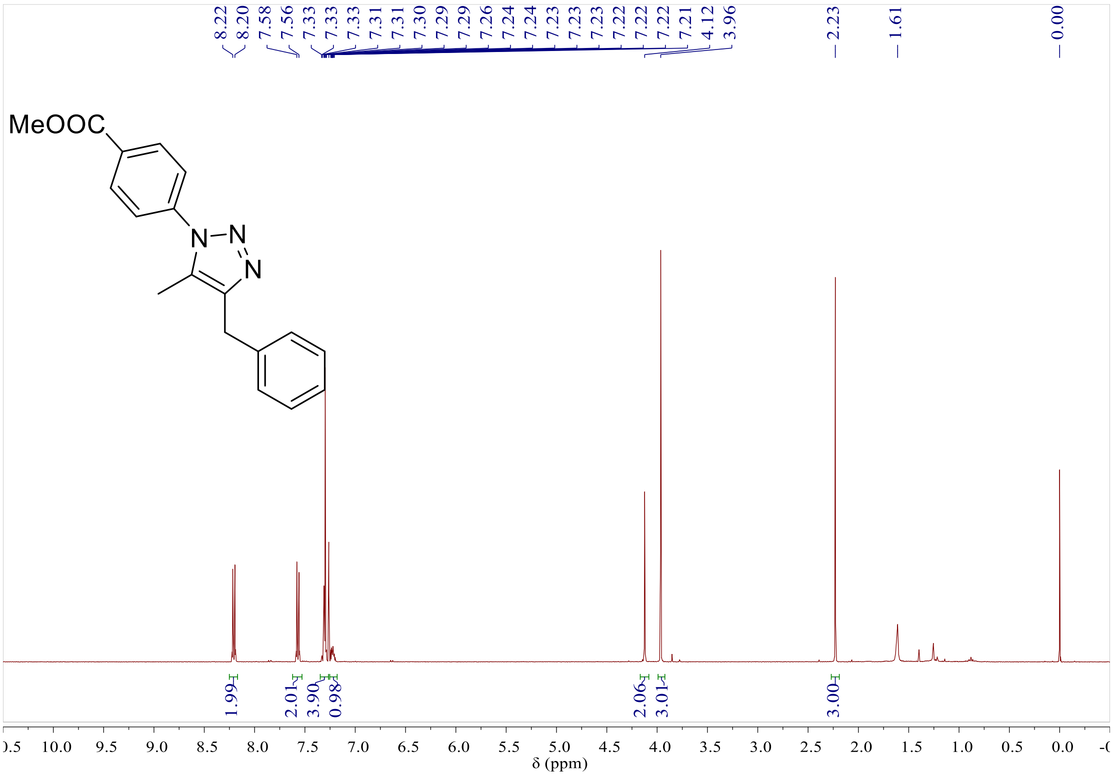

^{1}H NMR spectrum of **3af** in $CDCl_3$ (400 MHz)

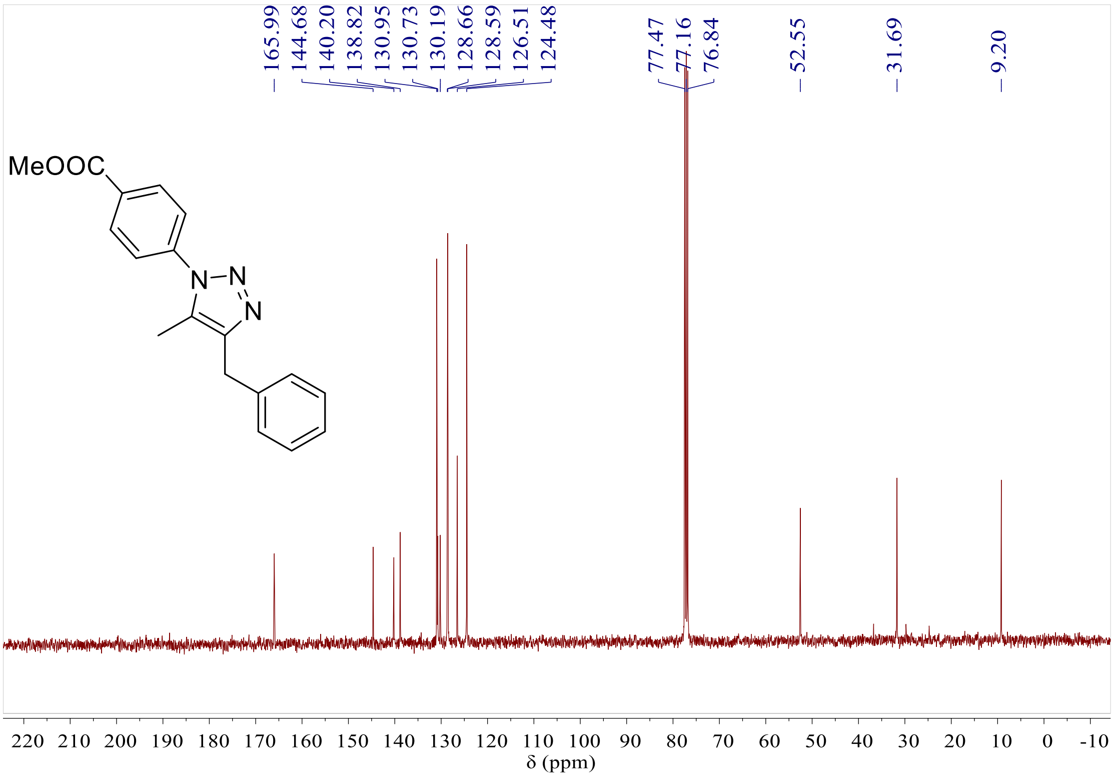

$^{13}C\{^1H\}$ NMR spectrum of **3af** in $CDCl_3$ (100 MHz)

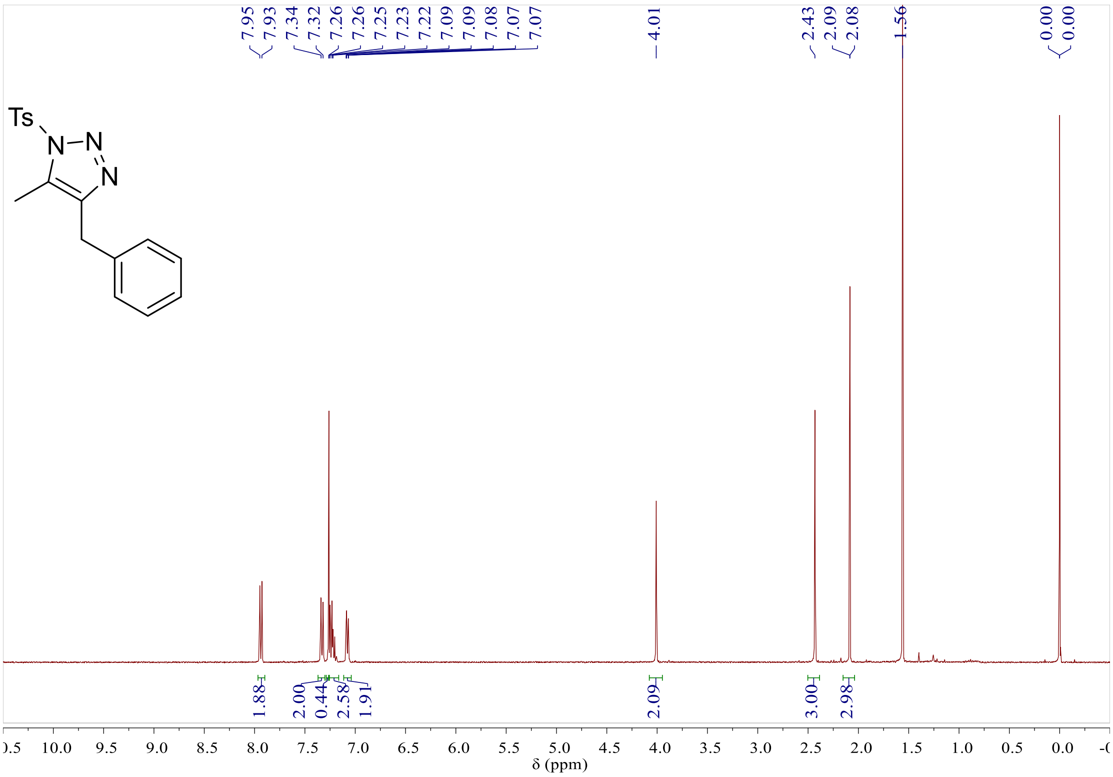

^{1}H NMR spectrum of **3ag** in $CDCl_3$ (400 MHz)

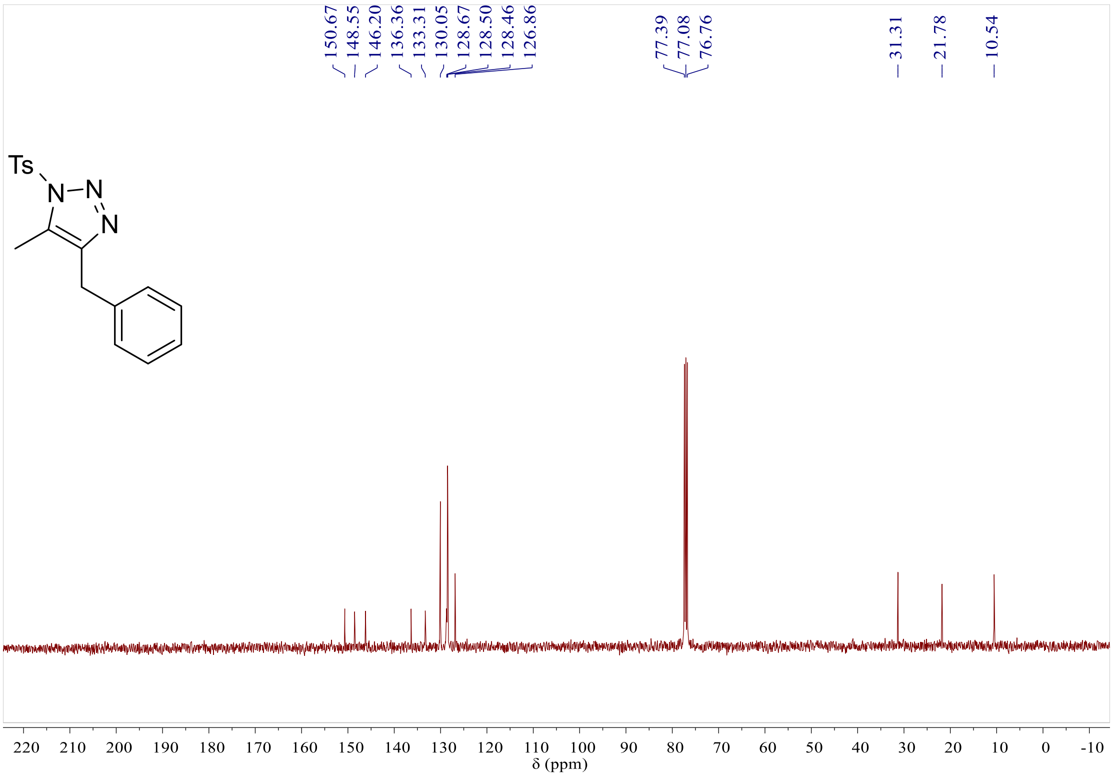

$^{13}C\{^1H\}$ NMR spectrum of **3ag** in $CDCl_3$ (100 MHz)

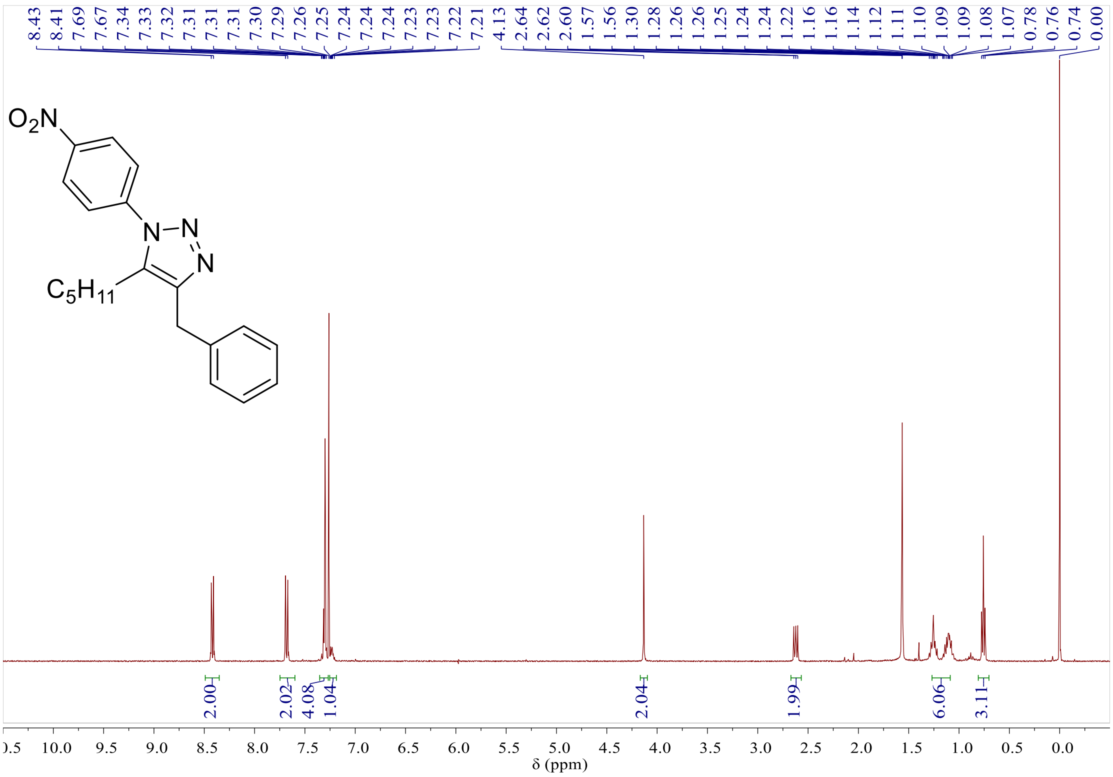

^{1}H NMR spectrum of **3be** in $CDCl_3$ (400 MHz)

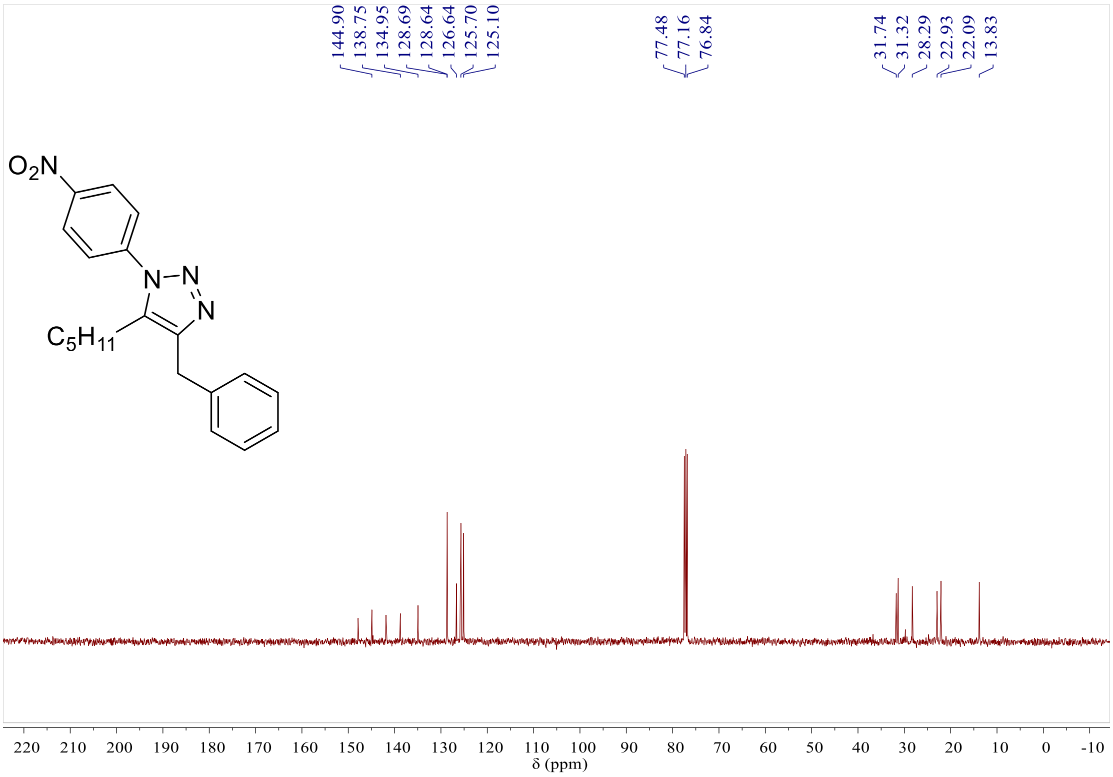

^{13}C{^{1}H} NMR spectrum of **3be** in $CDCl_3$ (100 MHz)

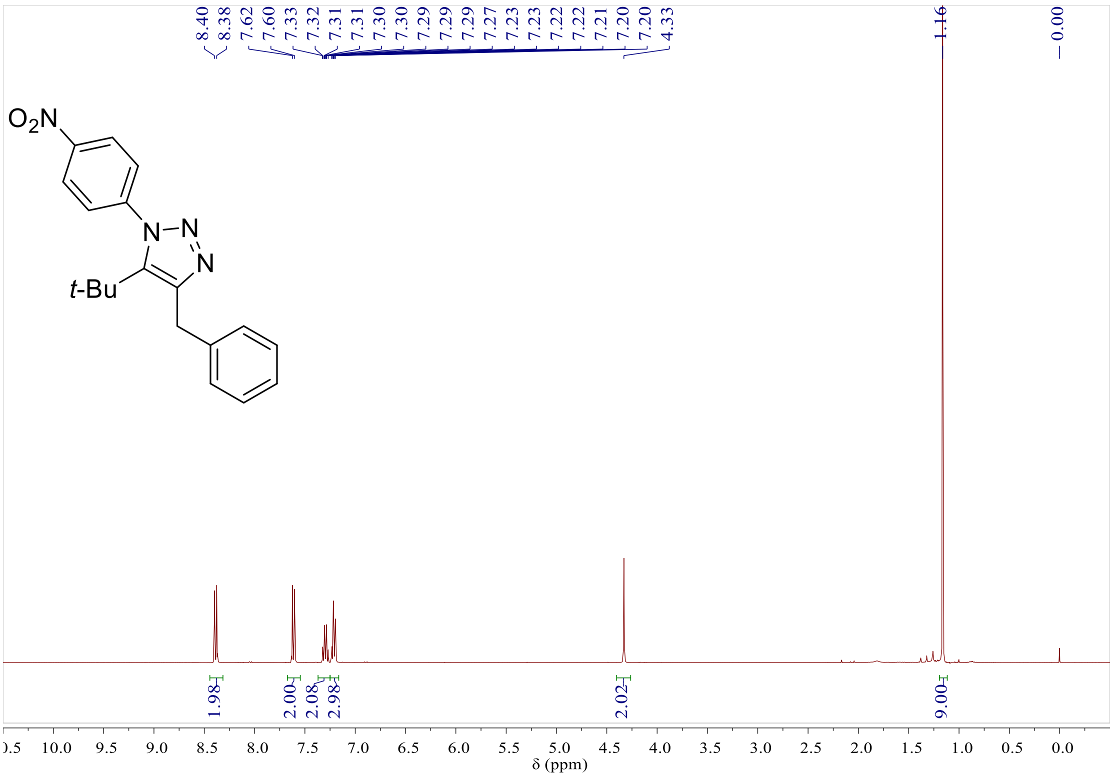

^{1}H NMR spectrum of **3ce** in $CDCl_3$ (400 MHz)

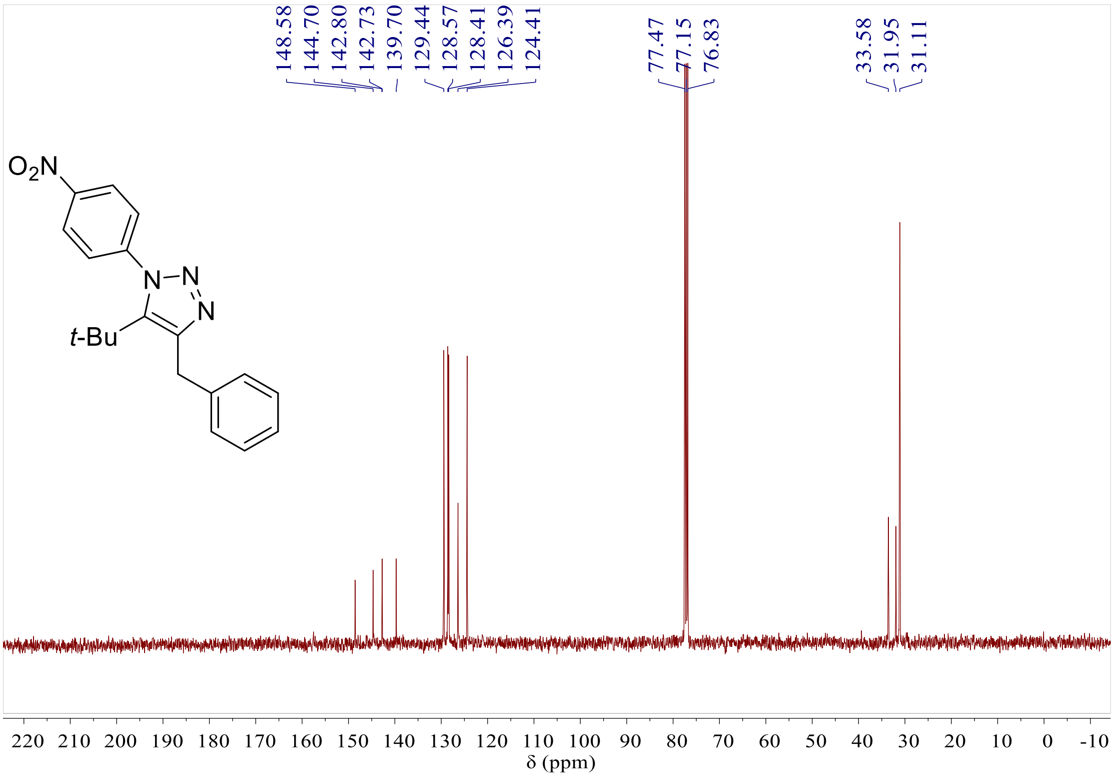

$^{13}C\{^{1}H\}$ NMR spectrum of **3ce** in $CDCl_3$ (100 MHz)

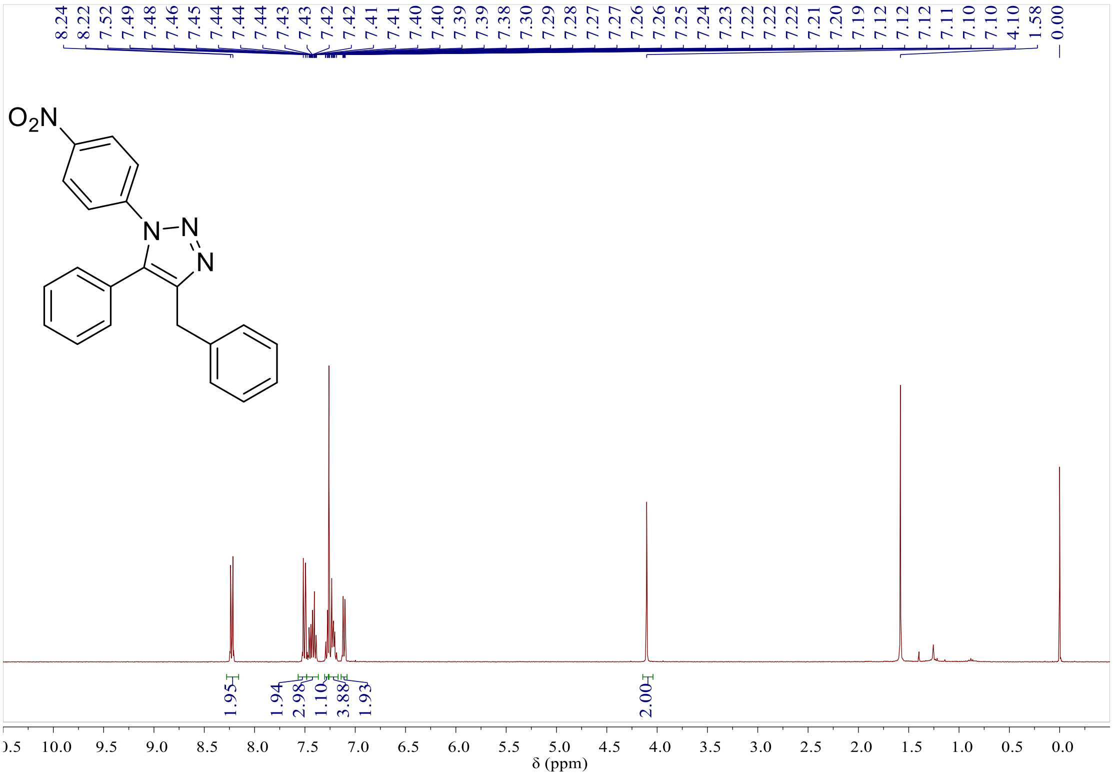

^{1}H NMR spectrum of **3de** in $CDCl_3$ (400 MHz)

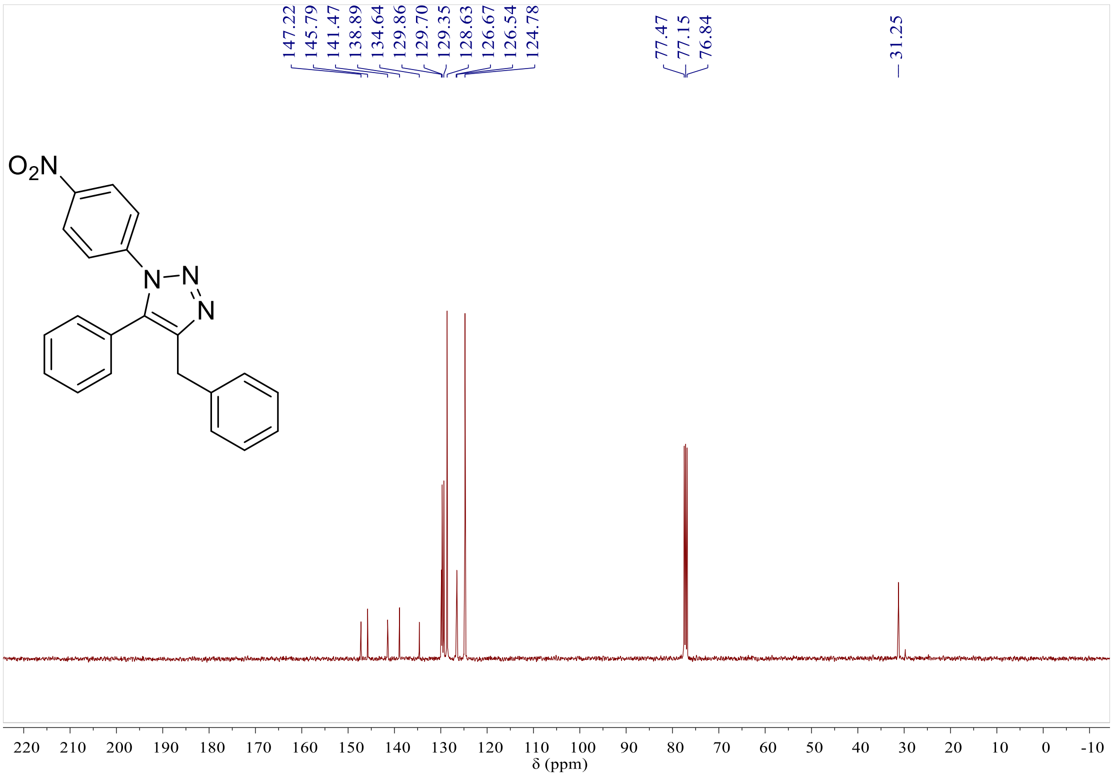

$^{13}C\{^{1}H\}$ NMR spectrum of **3de** in $CDCl_3$ (100 MHz)

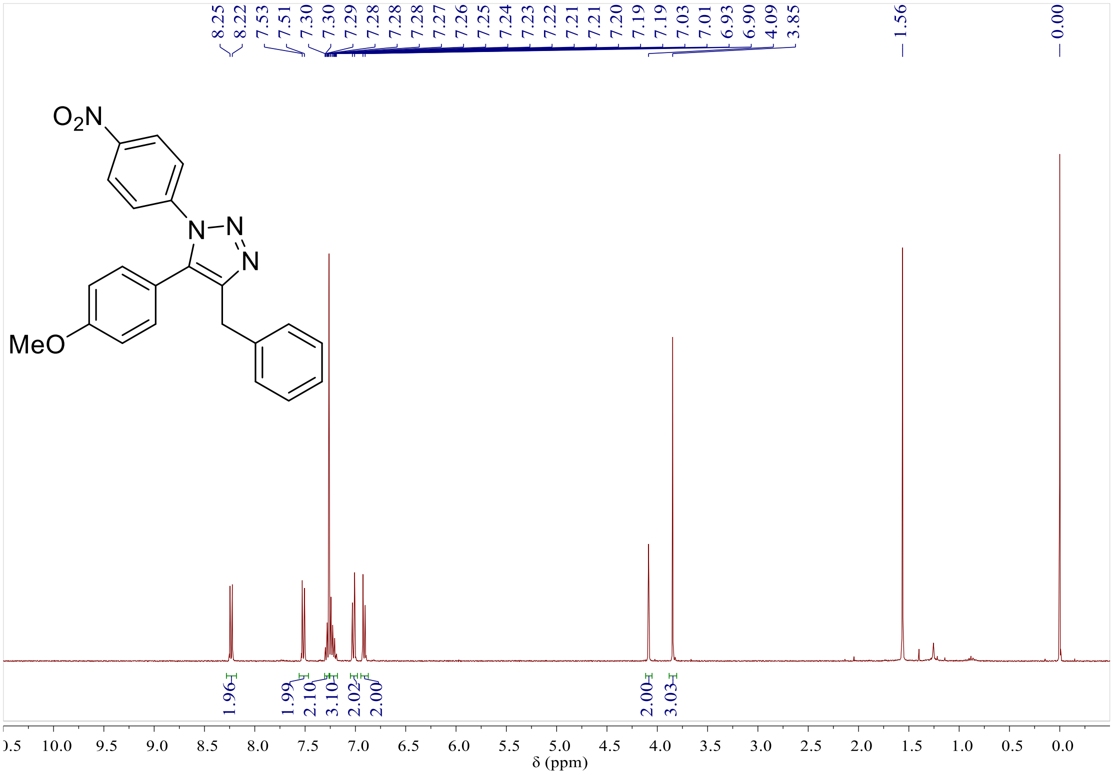

^{1}H NMR spectrum of **3ee** in $CDCl_3$ (400 MHz)

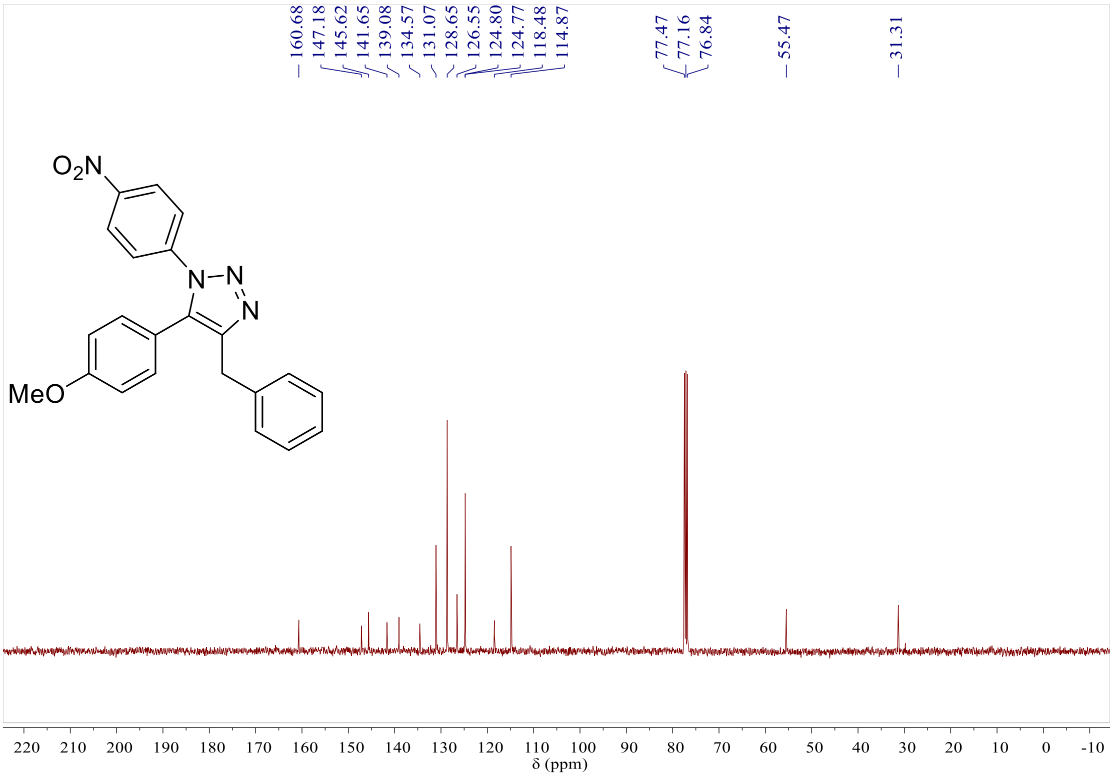

^{13}C{^{1}H} NMR spectrum of **3ee** in $CDCl_3$ (100 MHz)

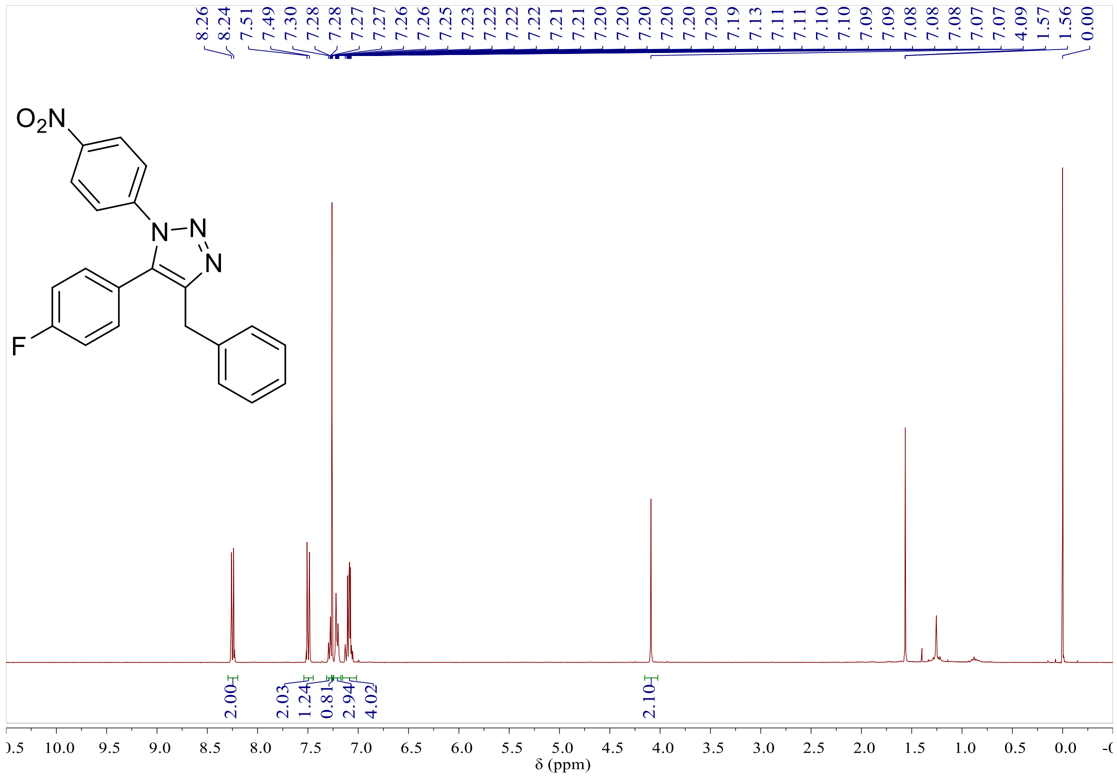

^{1}H NMR spectrum of **3fe** in $CDCl_3$ (400 MHz)

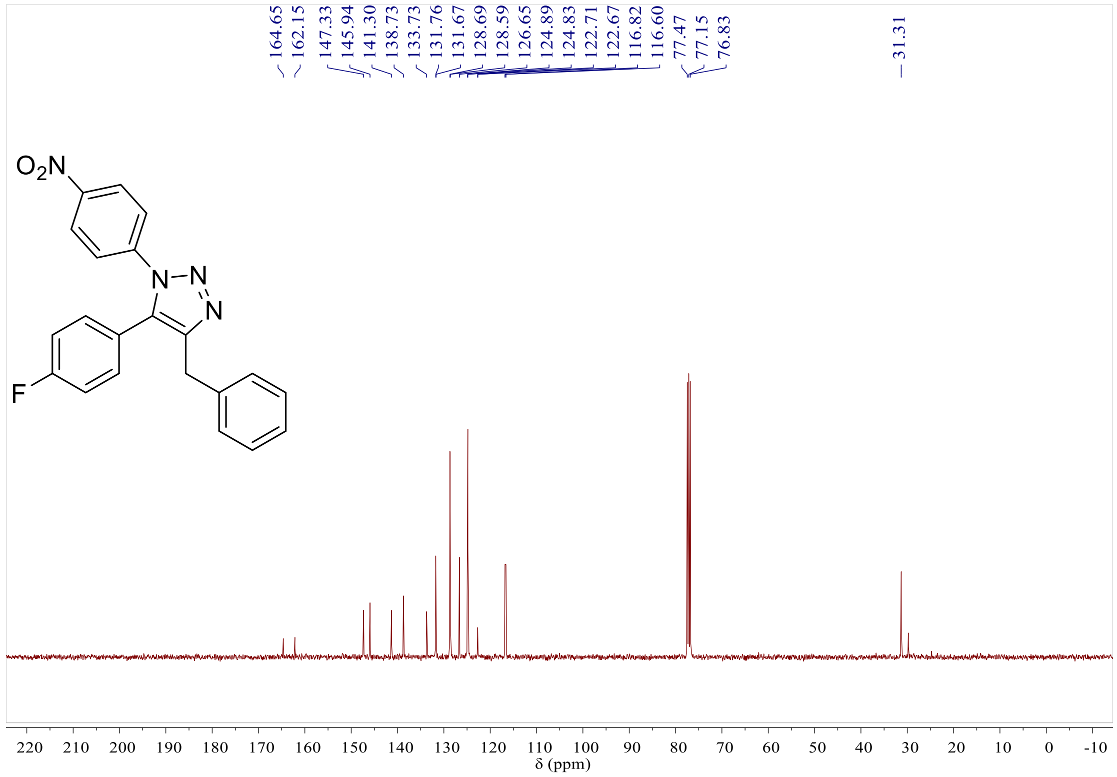

^{13}C{^{1}H} NMR spectrum of **3fe** in $CDCl_3$ (100 MHz)

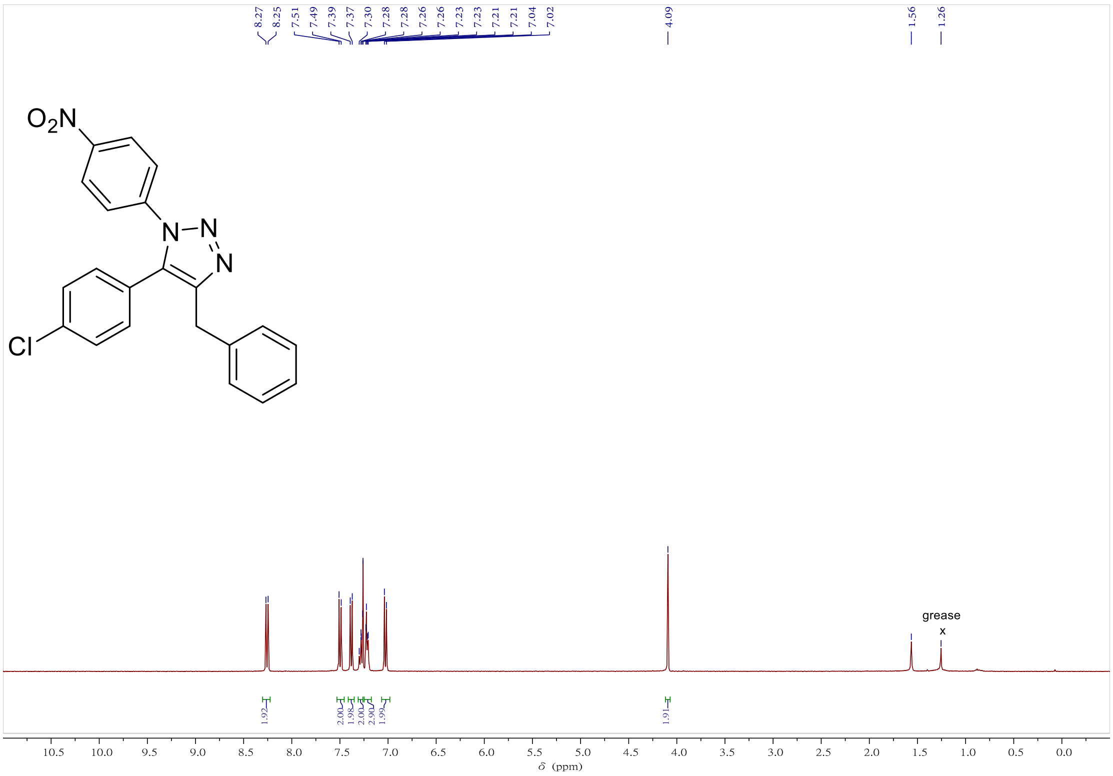

^{1}H NMR spectrum of **3ge** in $CDCl_3$ (400 MHz)

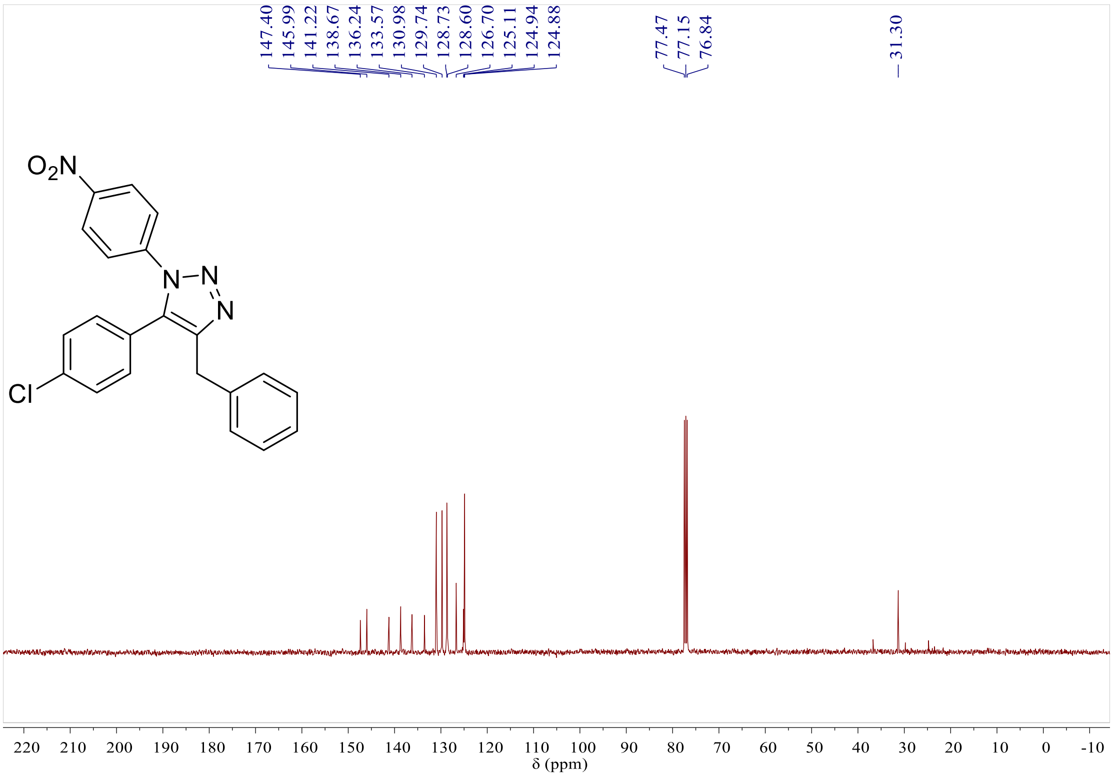

$^{13}C\{^1H\}$ NMR spectrum of **3ge** in $CDCl_3$ (100 MHz)

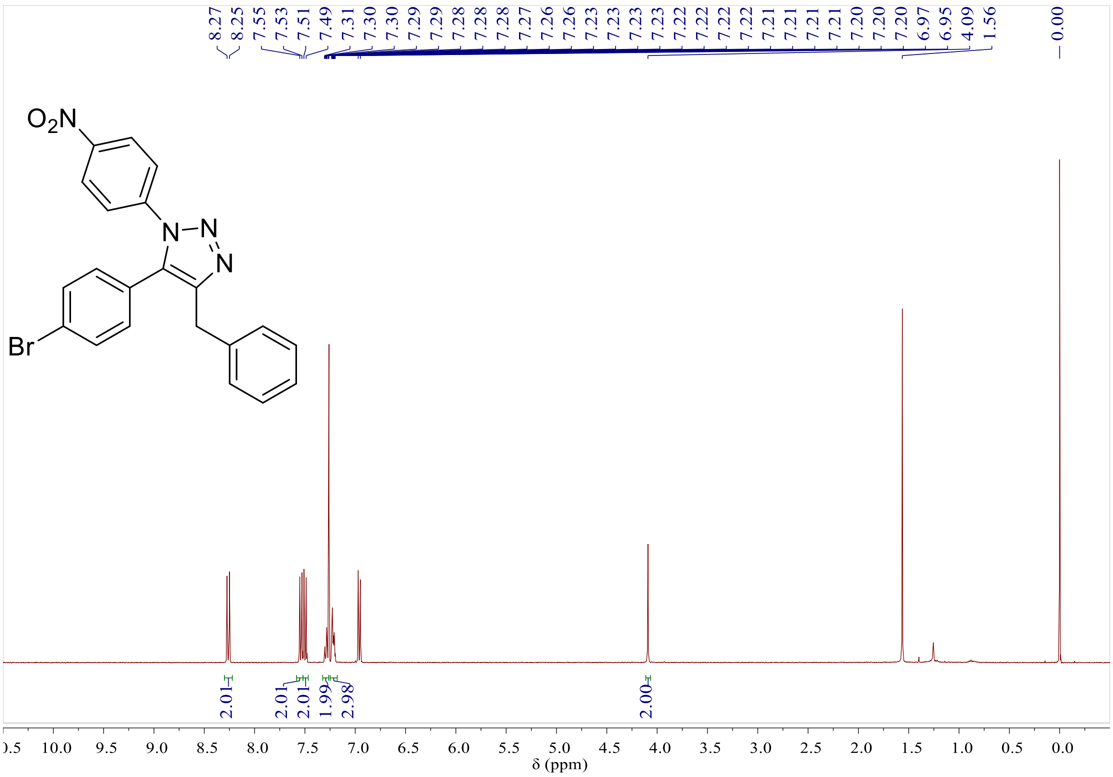

^{1}H NMR spectrum of **3he** in $CDCl_3$ (400 MHz)

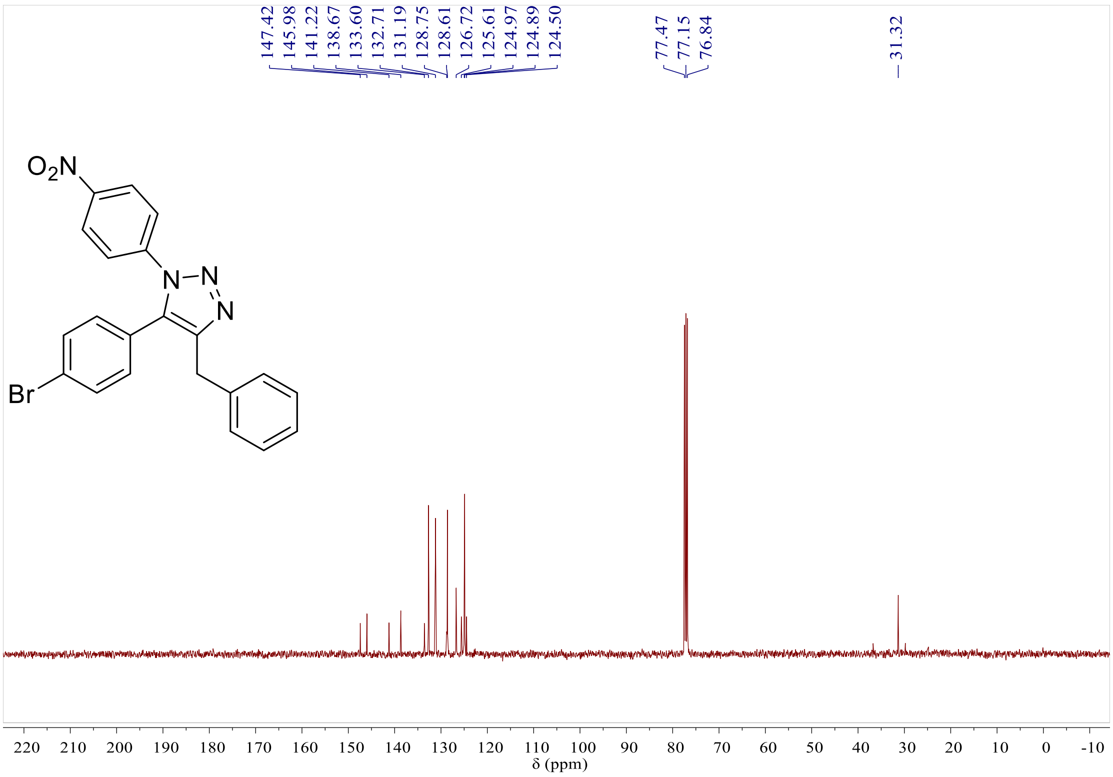

^{13}C{^{1}H} NMR spectrum of **3he** in $CDCl_3$ (100 MHz)

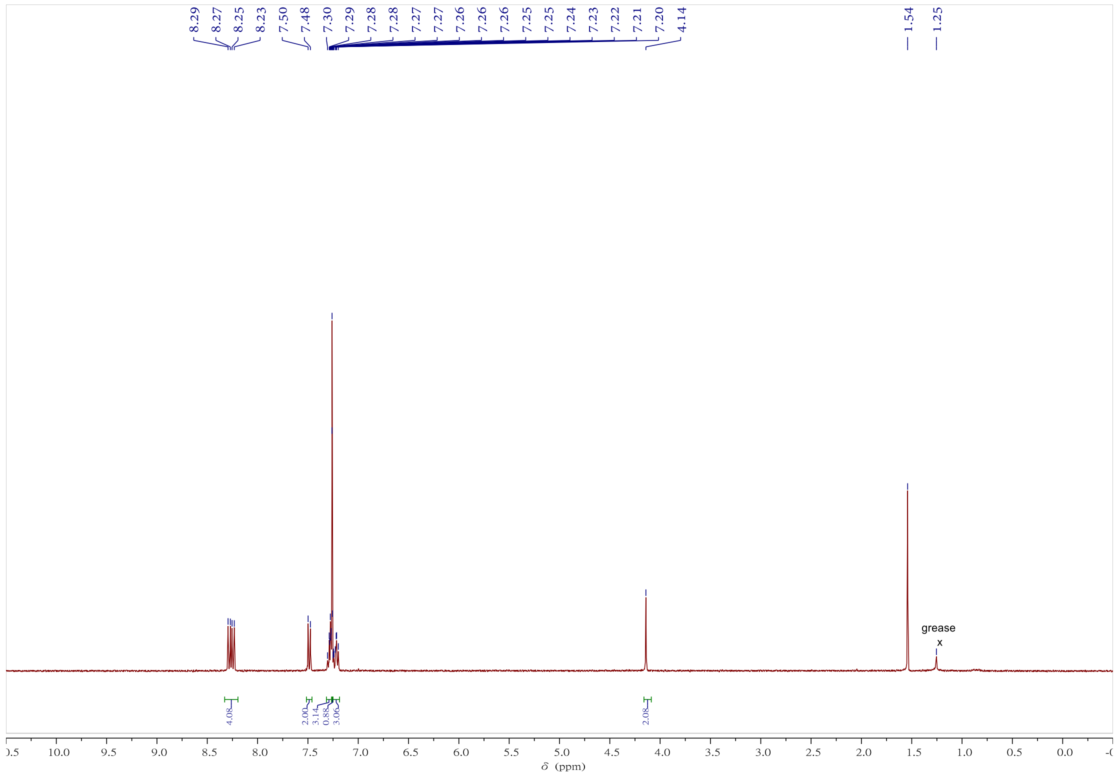

^{1}H NMR spectrum of **3ie** in $CDCl_3$ (400 MHz)

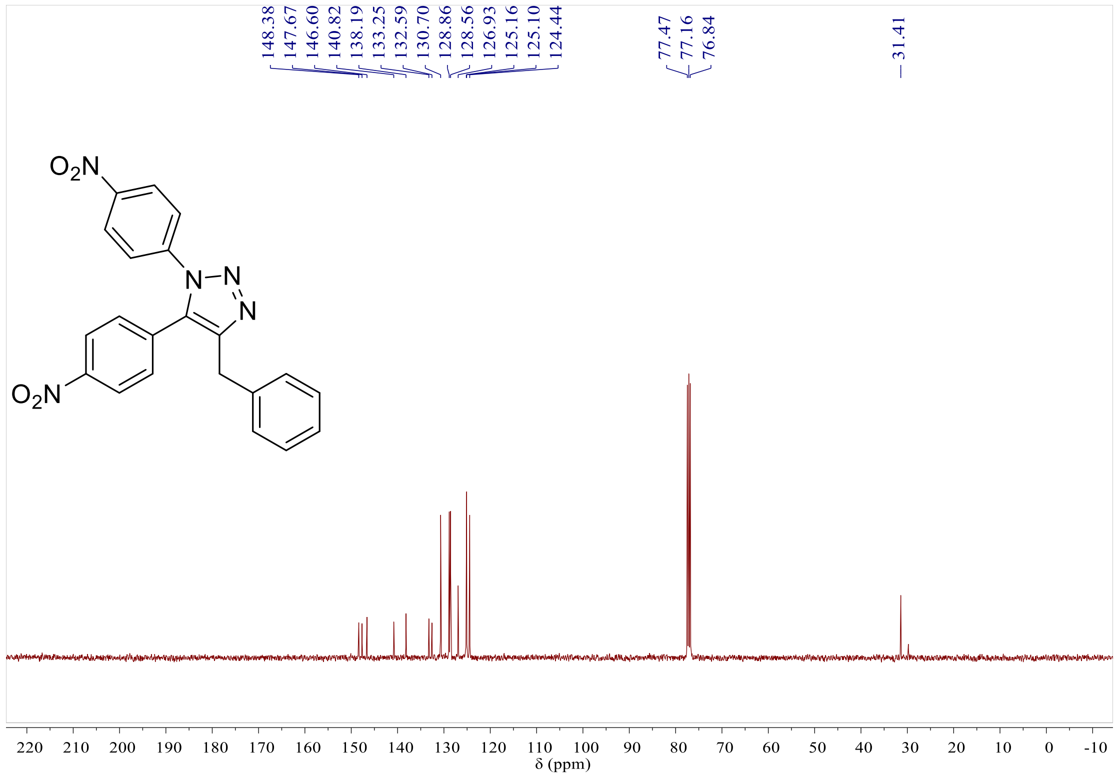

^{13}C{^{1}H} NMR spectrum of **3ie** in $CDCl_3$ (100 MHz)

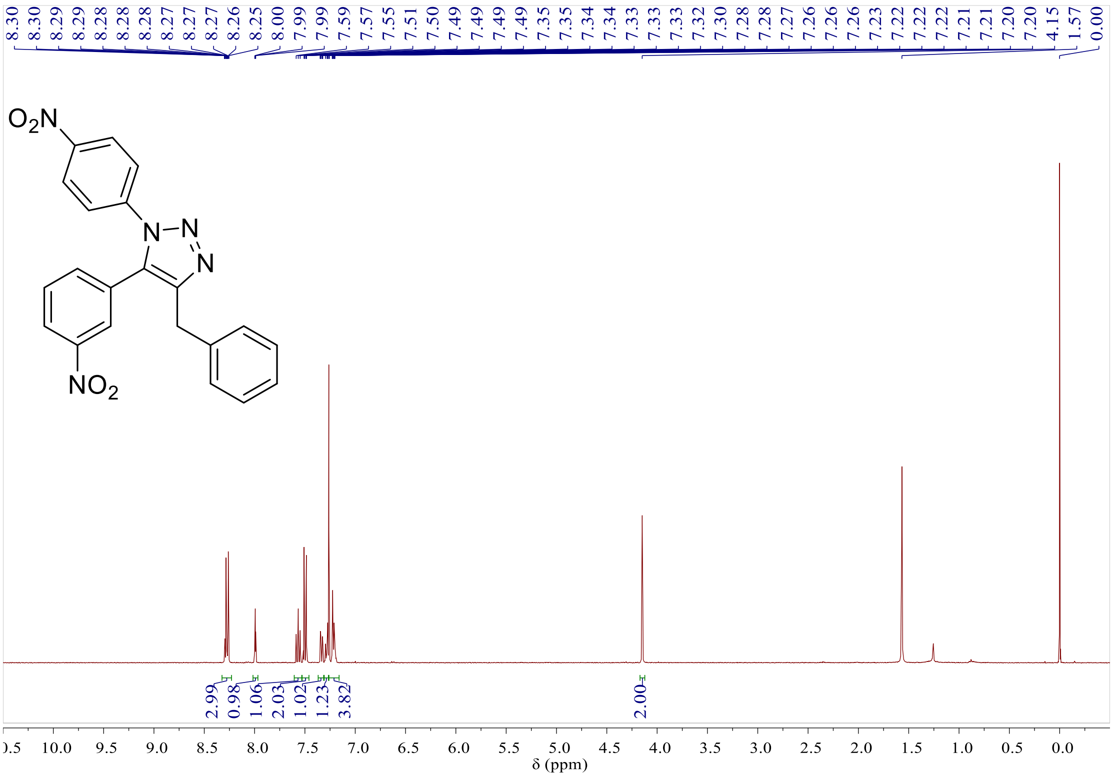

^{1}H NMR spectrum of **3je** in $CDCl_3$ (400 MHz)

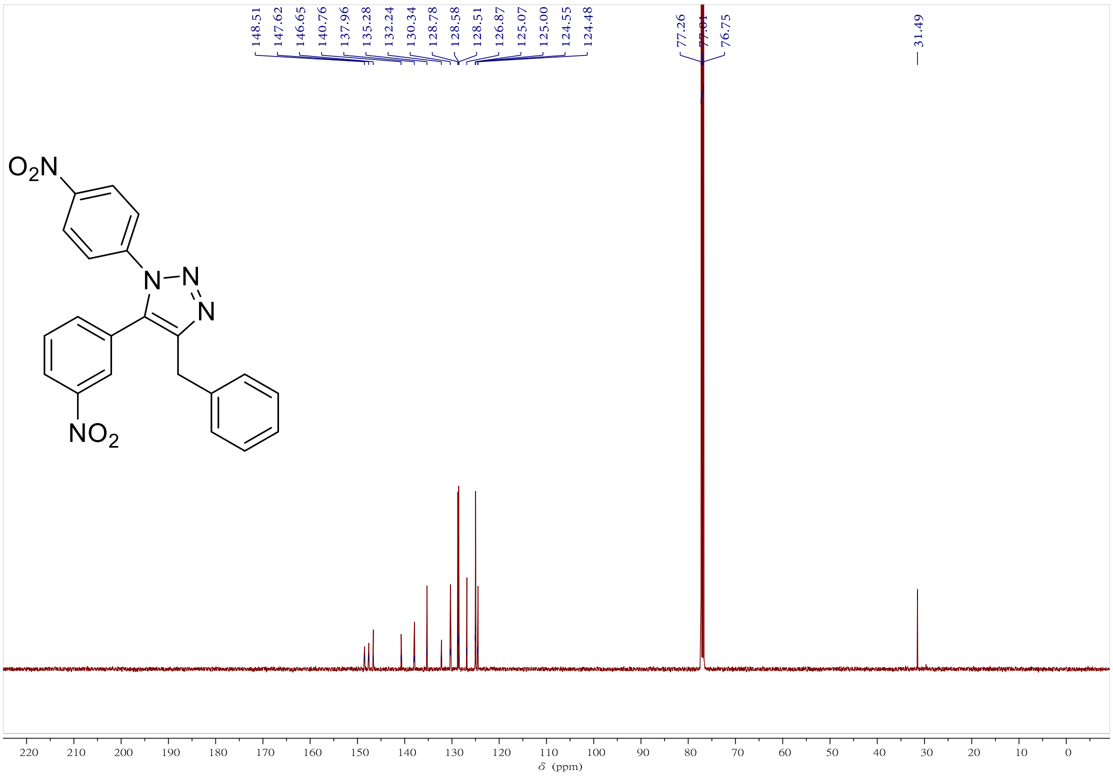

$^{13}C\{^{1}H\}$ NMR spectrum of **3je** in $CDCl_3$ (125 MHz)

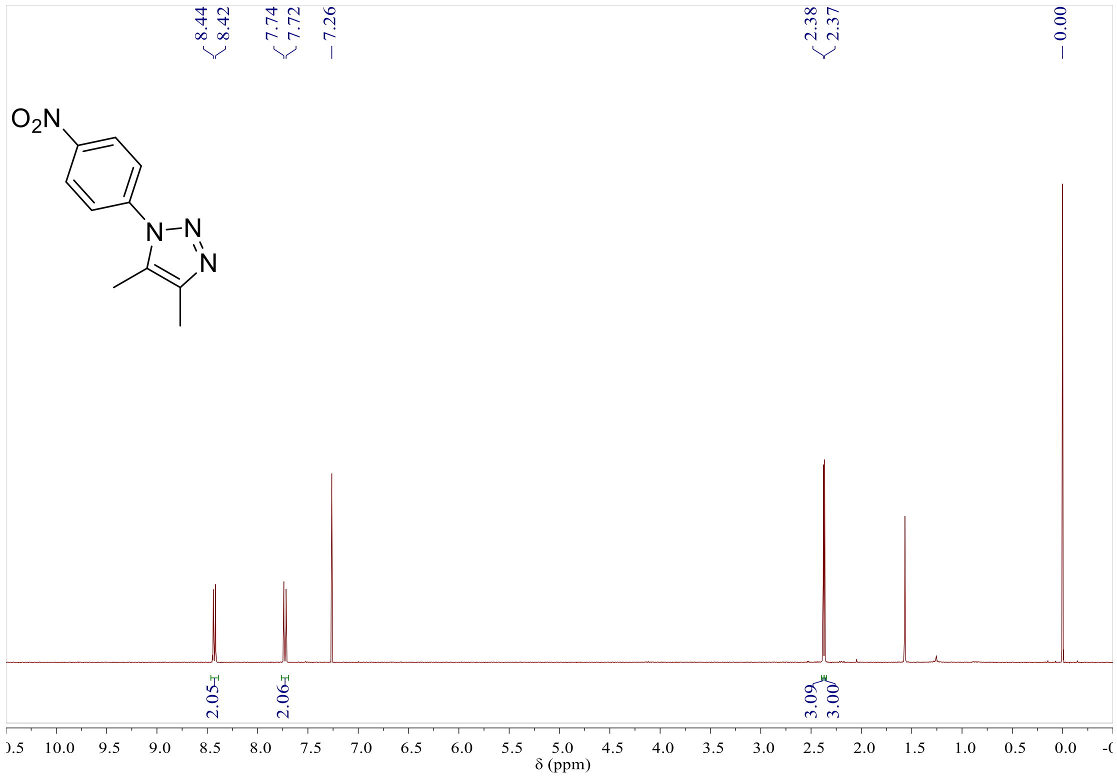

^{1}H NMR spectrum of **3ke** in $CDCl_3$ (400 MHz)

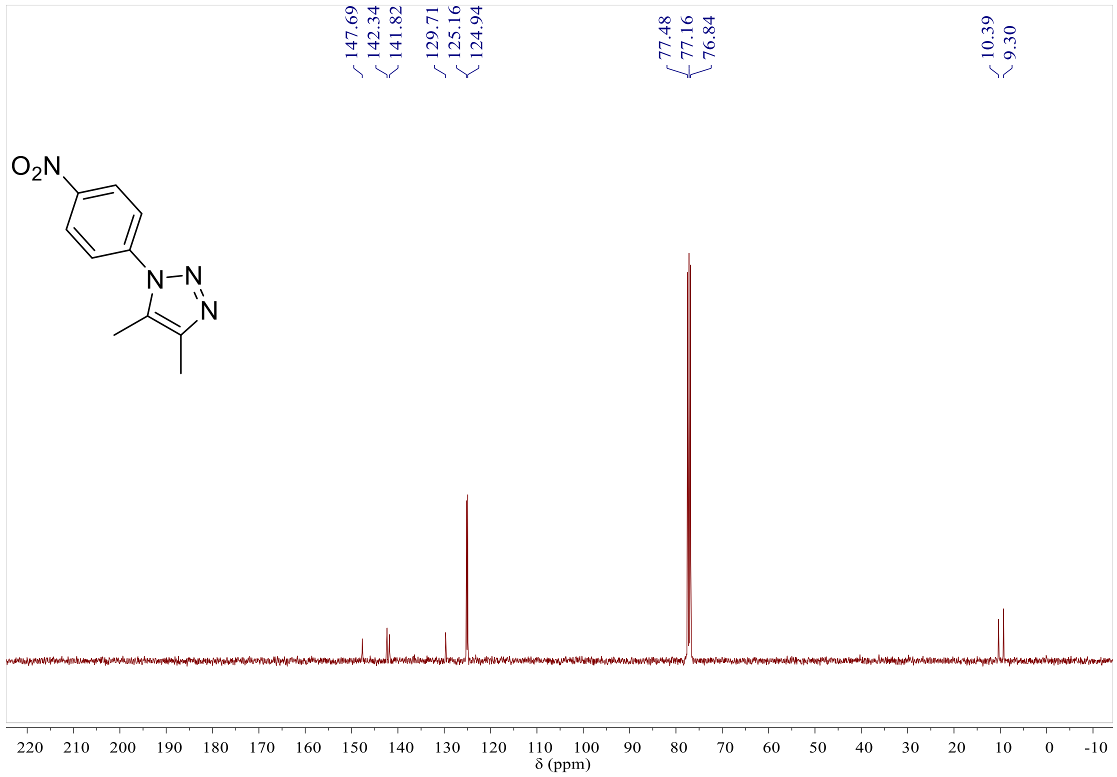

$^{13}C\{^{1}H\}$ NMR spectrum of **3ke** in $CDCl_3$ (100 MHz)

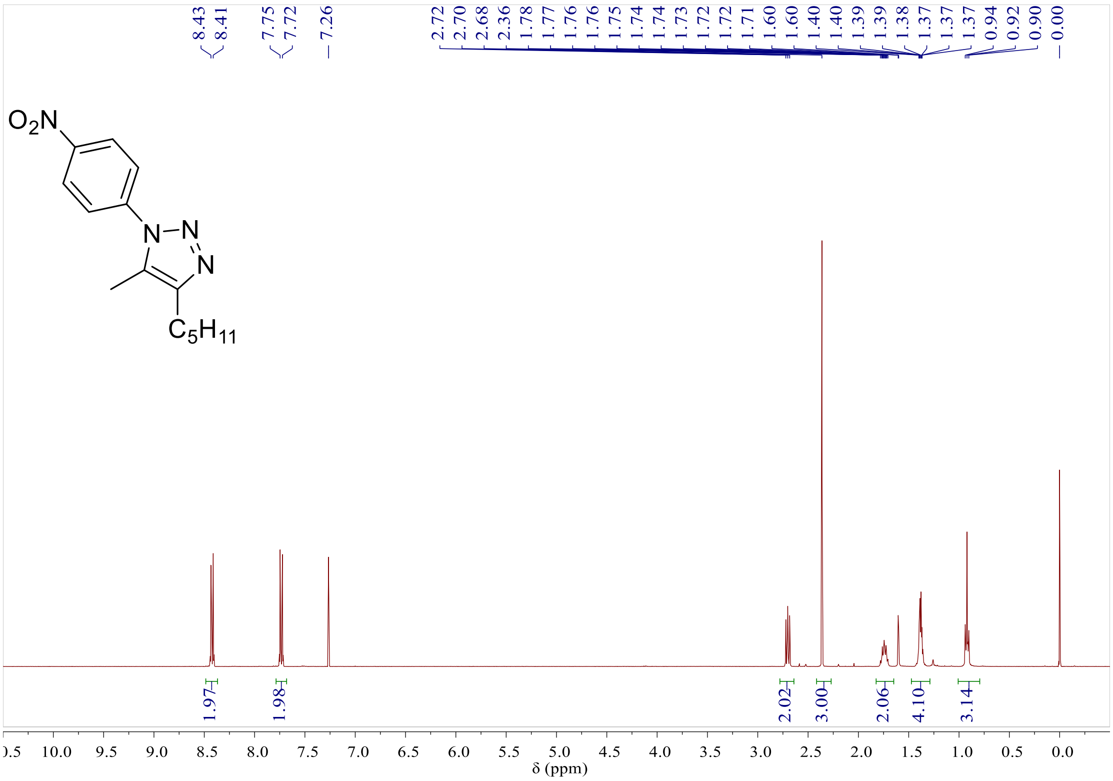

^{1}H NMR spectrum of **3le** in $CDCl_3$ (400 MHz)

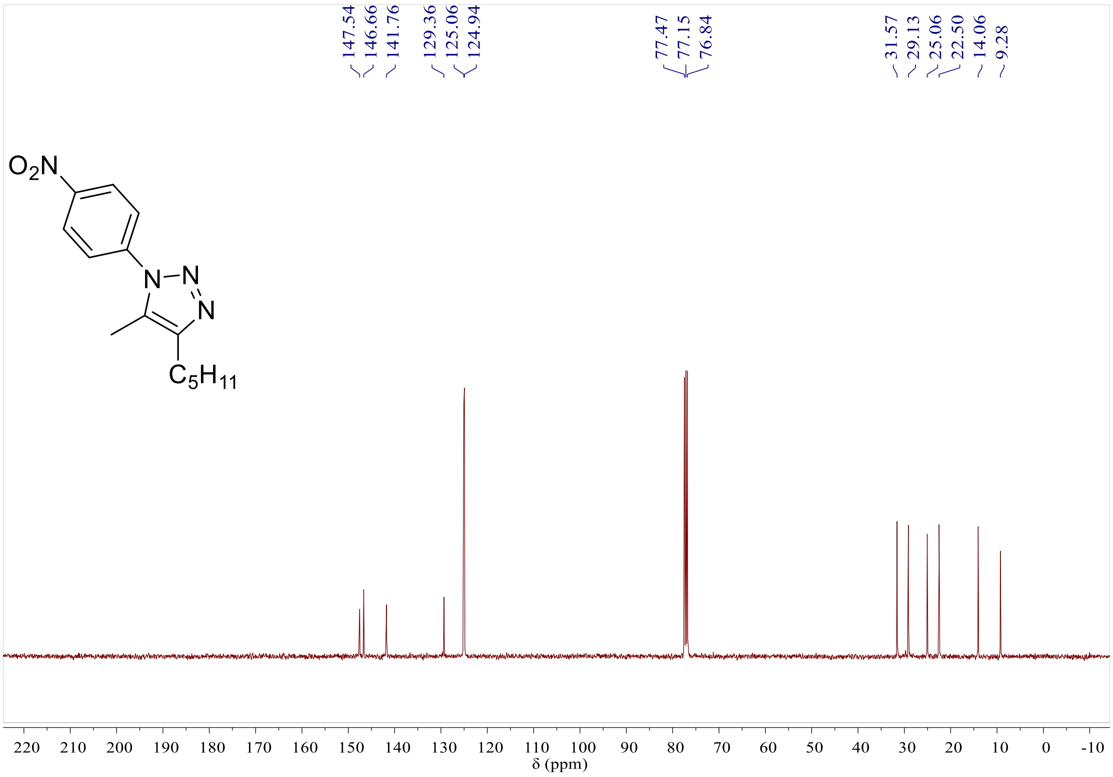

$^{13}C\{^1H\}$ NMR spectrum of **3le** in $CDCl_3$ (100 MHz)

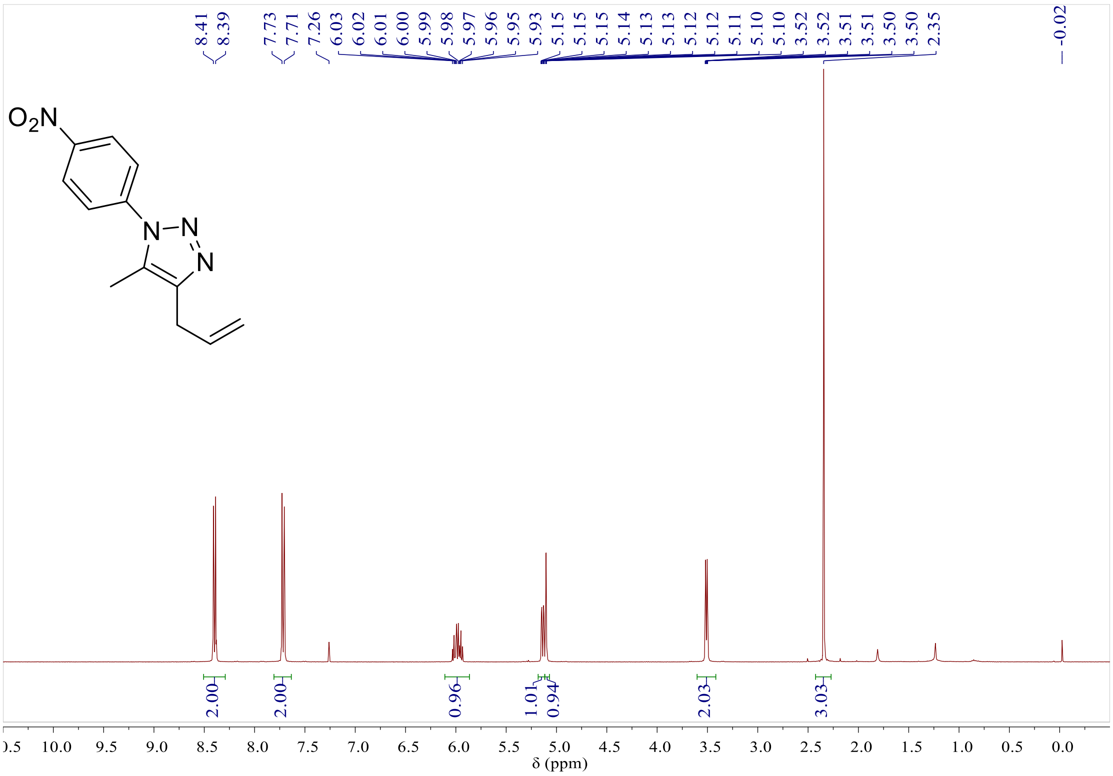

^1H NMR spectrum of **3me** in $CDCl_3$ (400 MHz)

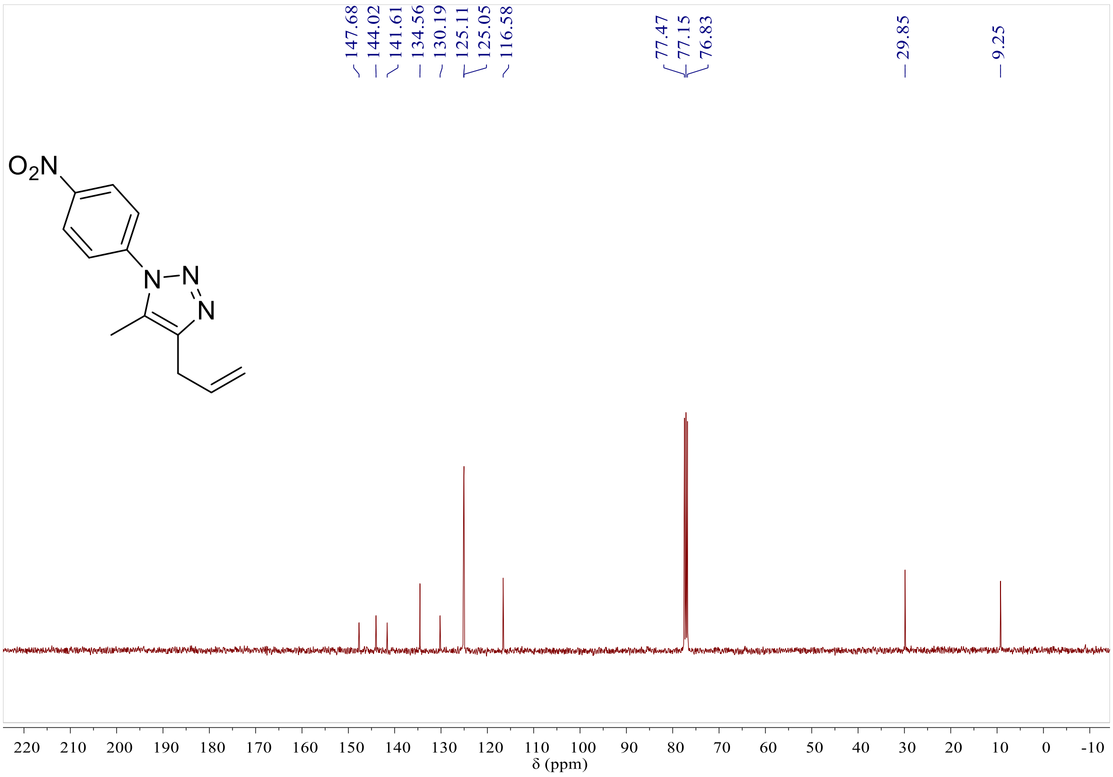

$^{13}C\{^1H\}$ NMR spectrum of **3me** in $CDCl_3$ (100 MHz)

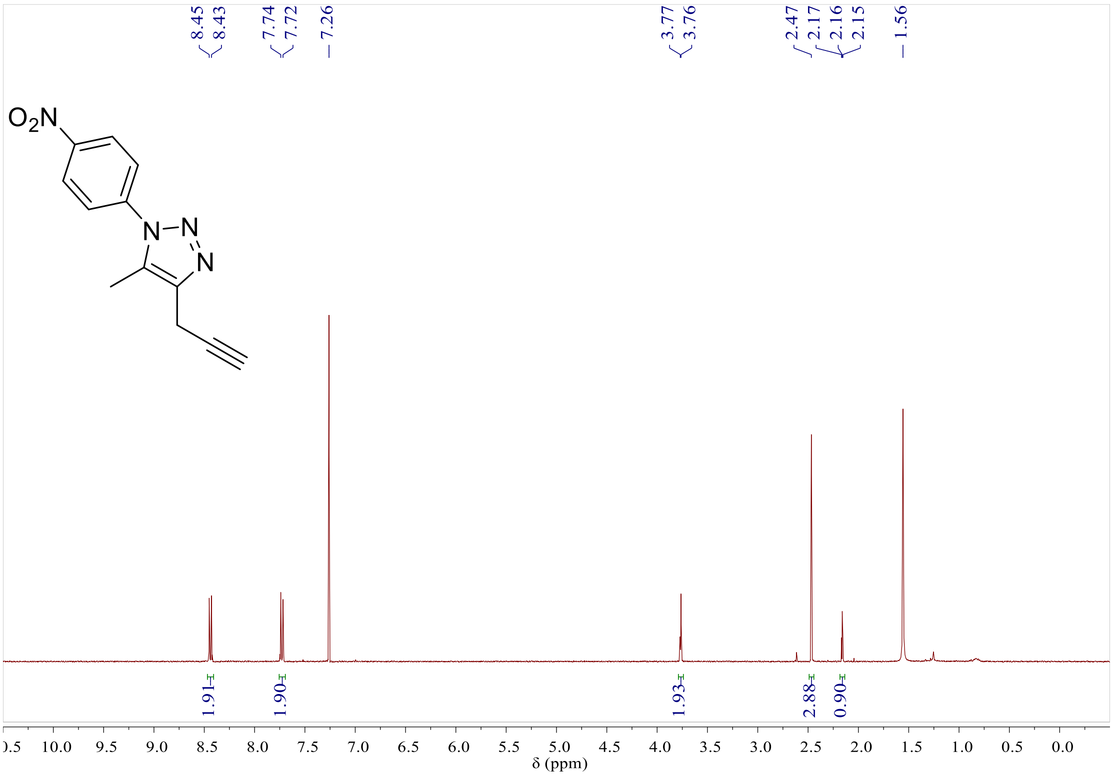

^{1}H NMR spectrum of **3ne** in $CDCl_3$ (400 MHz)

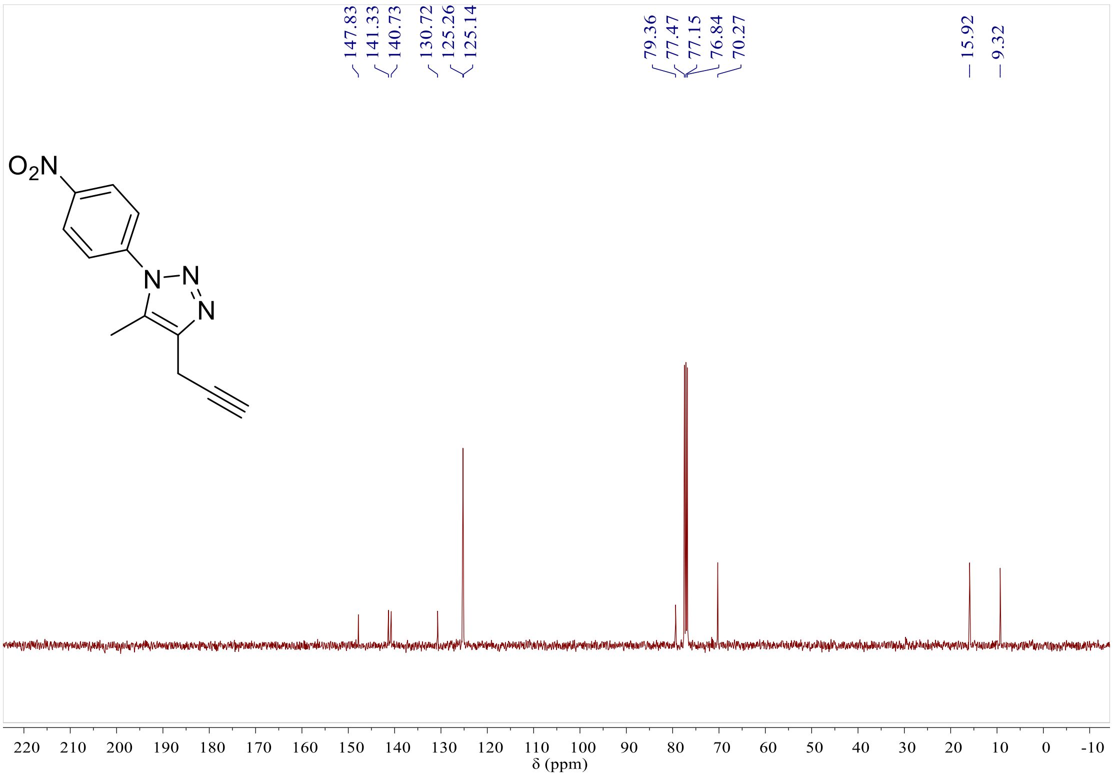

$^{13}C\{^1H\}$ NMR spectrum of **3ne** in $CDCl_3$ (100 MHz)

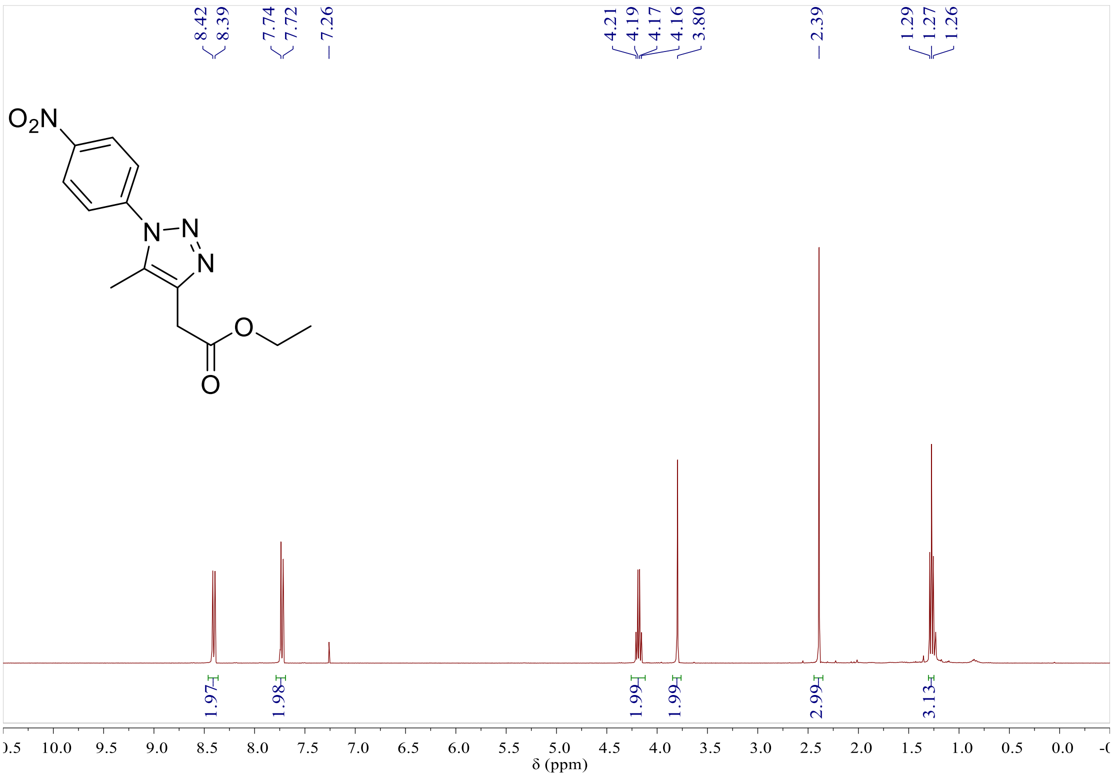

^{1}H NMR spectrum of **3oe** in $CDCl_3$ (400 MHz)

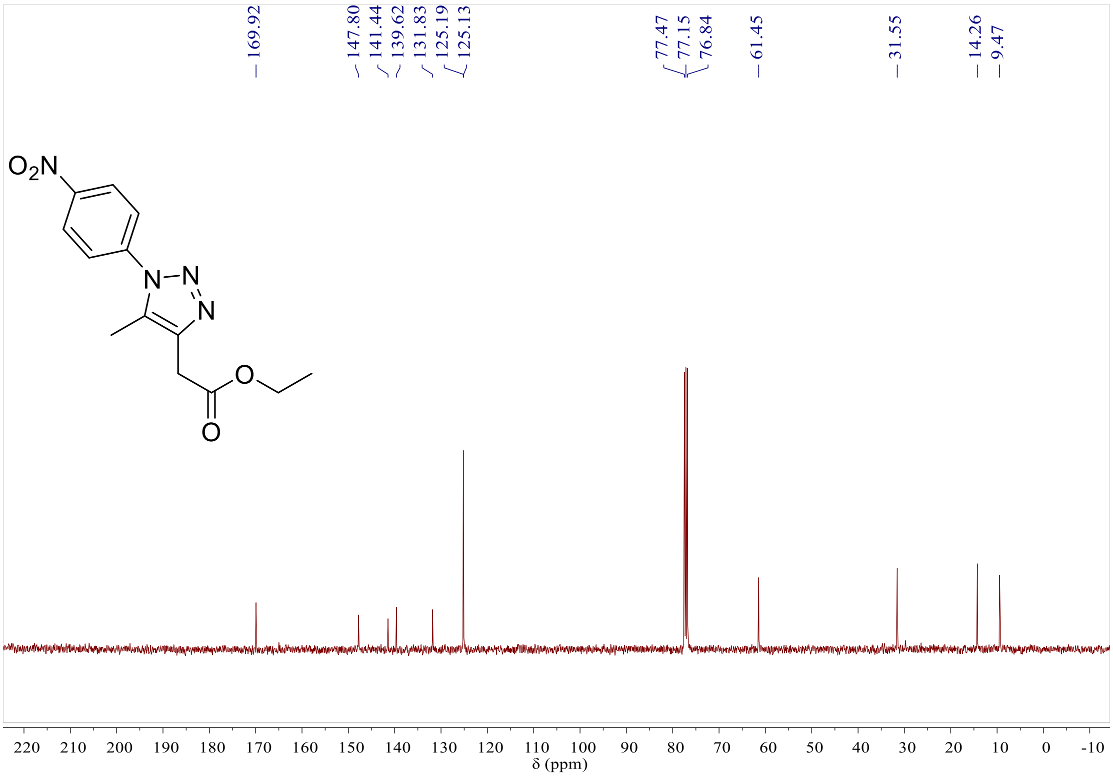

$^{13}C\{^{1}H\}$ NMR spectrum of **3oe** in $CDCl_3$ (100 MHz)

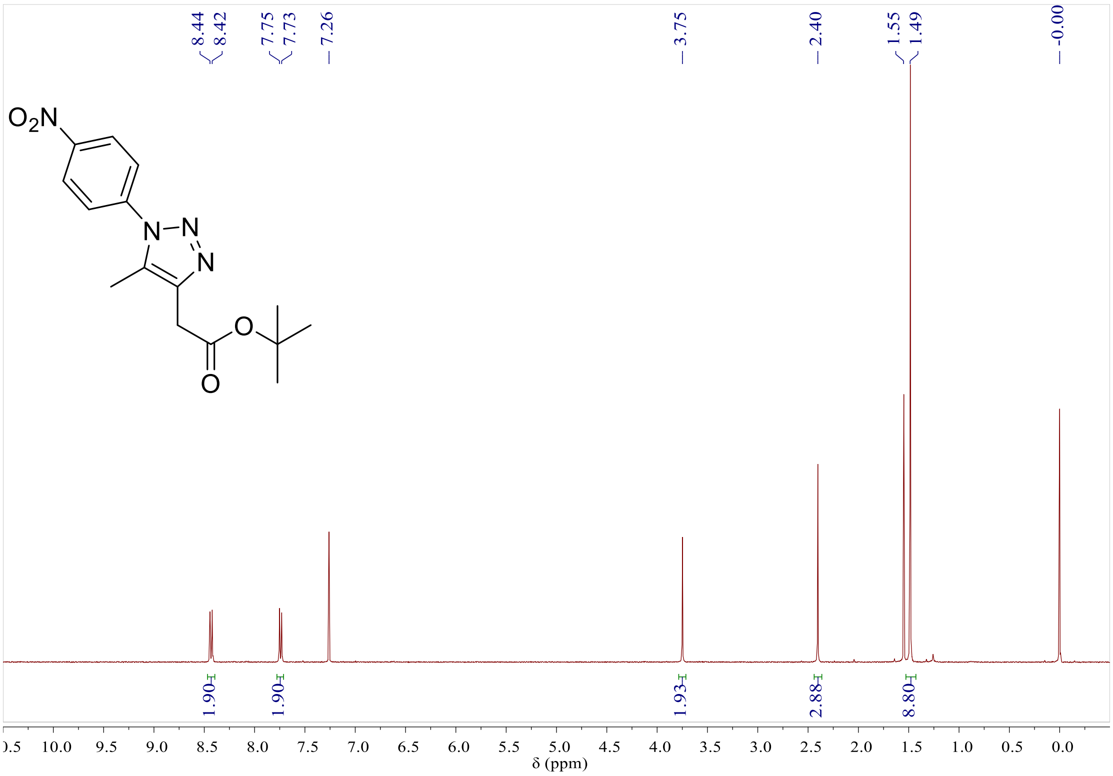

^{1}H NMR spectrum of **3pe** in $CDCl_3$ (400 MHz)

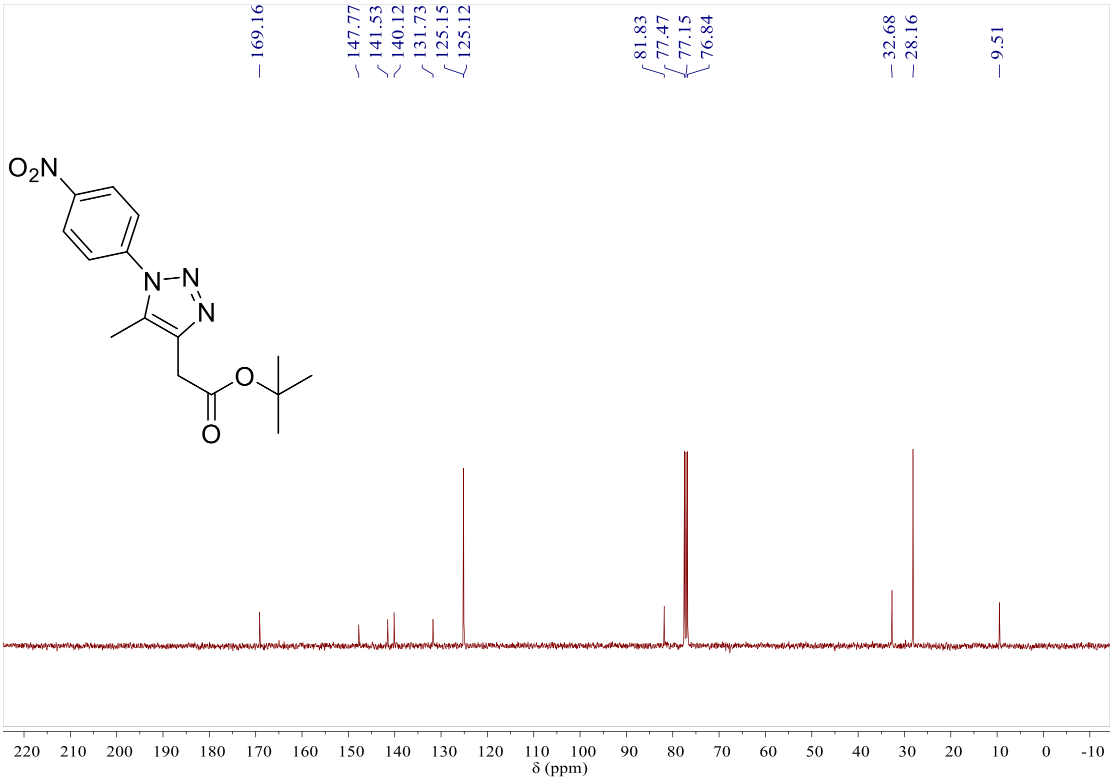

^{13}C{^{1}H} NMR spectrum of **3pe** in $CDCl_3$ (100 MHz)

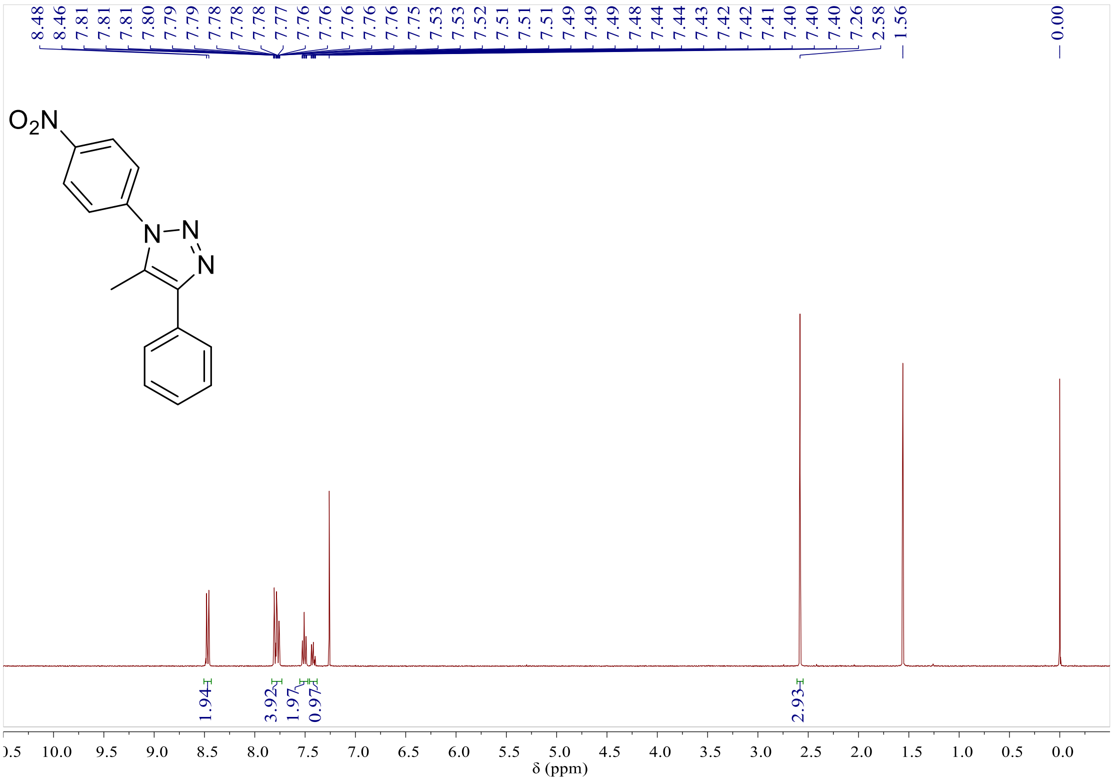

^{1}H NMR spectrum of **3qe** in $CDCl_3$ (400 MHz)

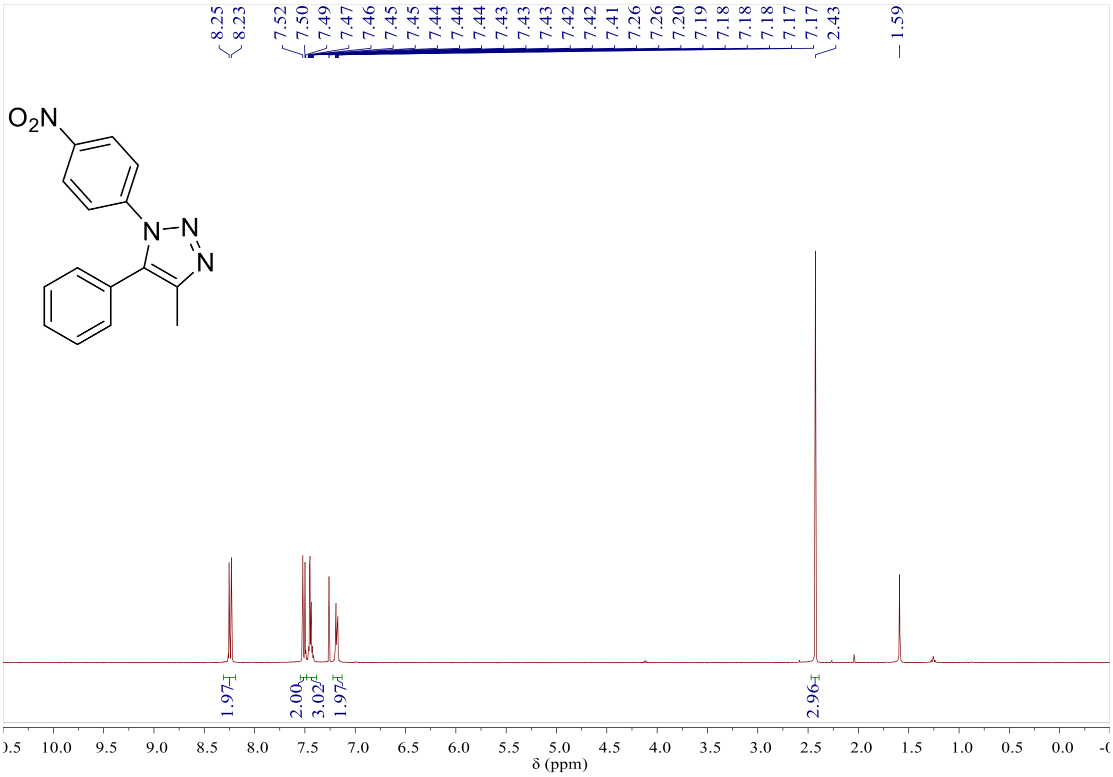

^{1}H NMR spectrum of **3re** in $CDCl_3$ (400 MHz)

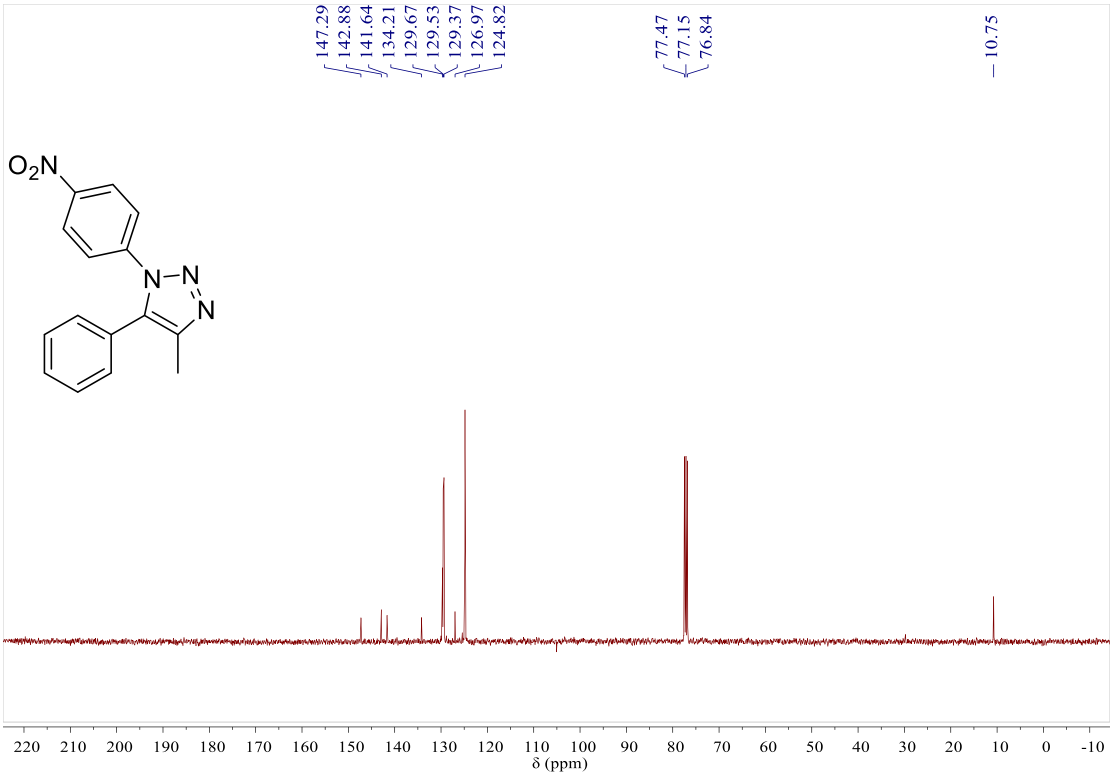

$^{13}C\{^{1}H\}$ NMR spectrum of **3re** in $CDCl_3$ (100 MHz)

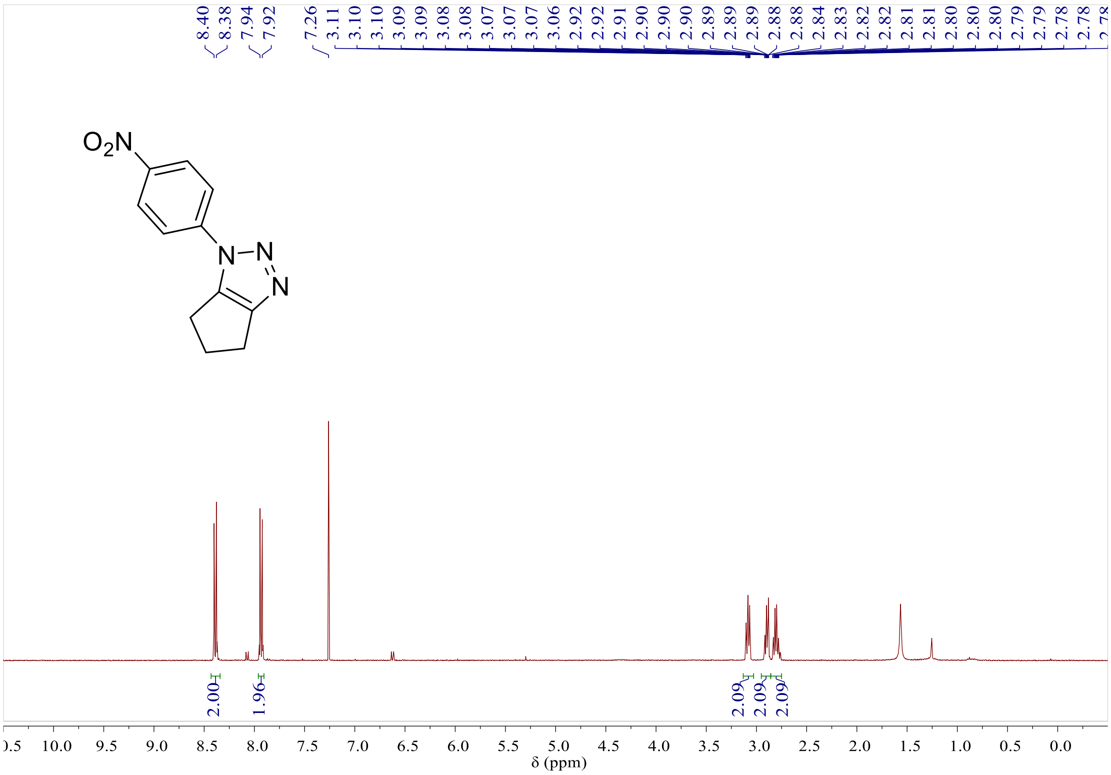

^{1}H NMR spectrum of **3se** in $CDCl_3$ (400 MHz)

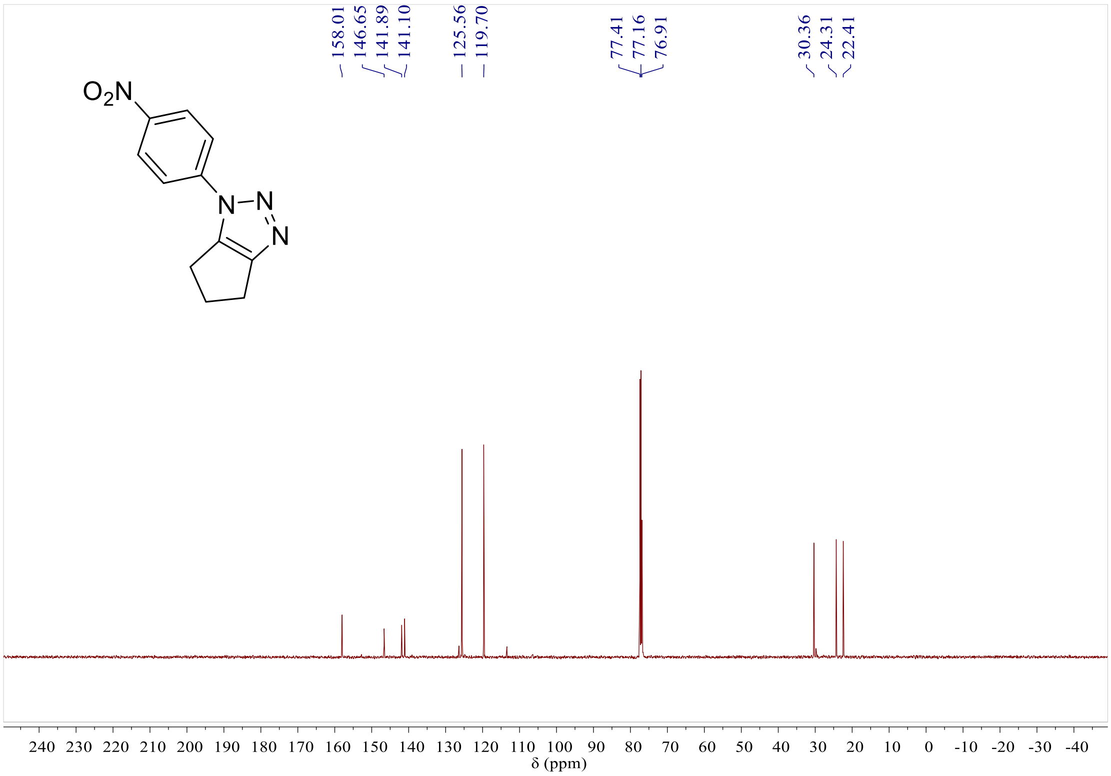

$^{13}C\{^{1}H\}$ NMR spectrum of **3se** in $CDCl_3$ (125 MHz)

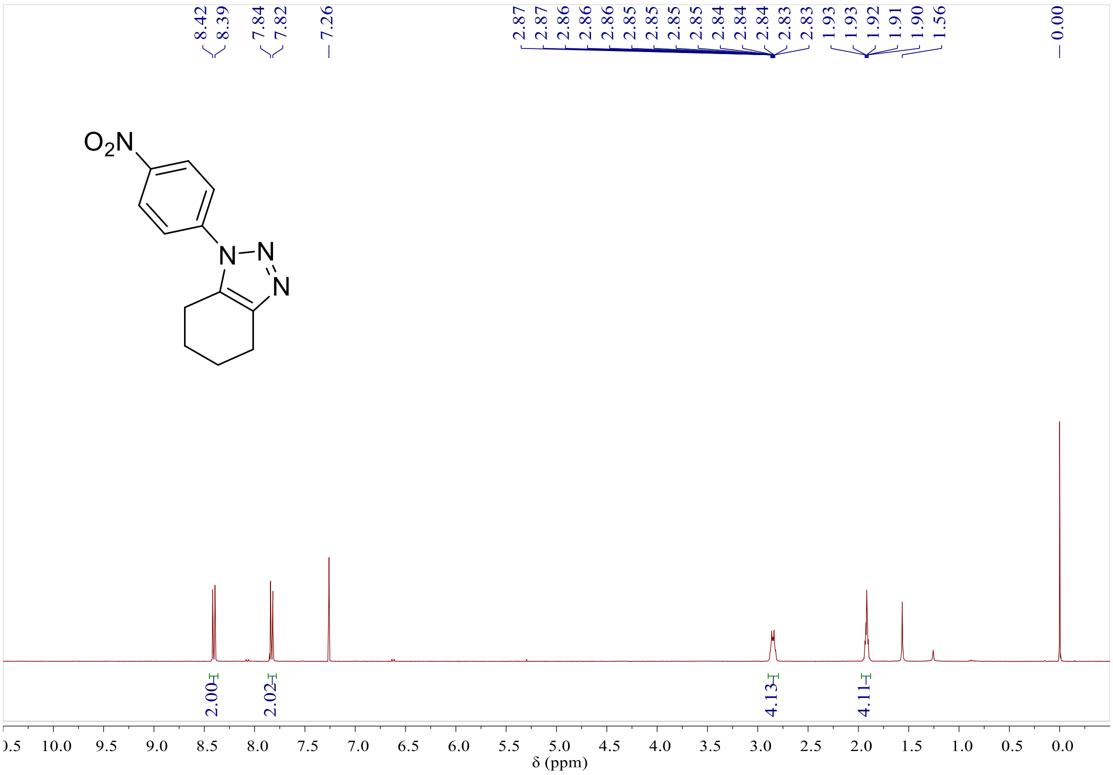

^{1}H NMR spectrum of **3te** in $CDCl_3$ (400 MHz)

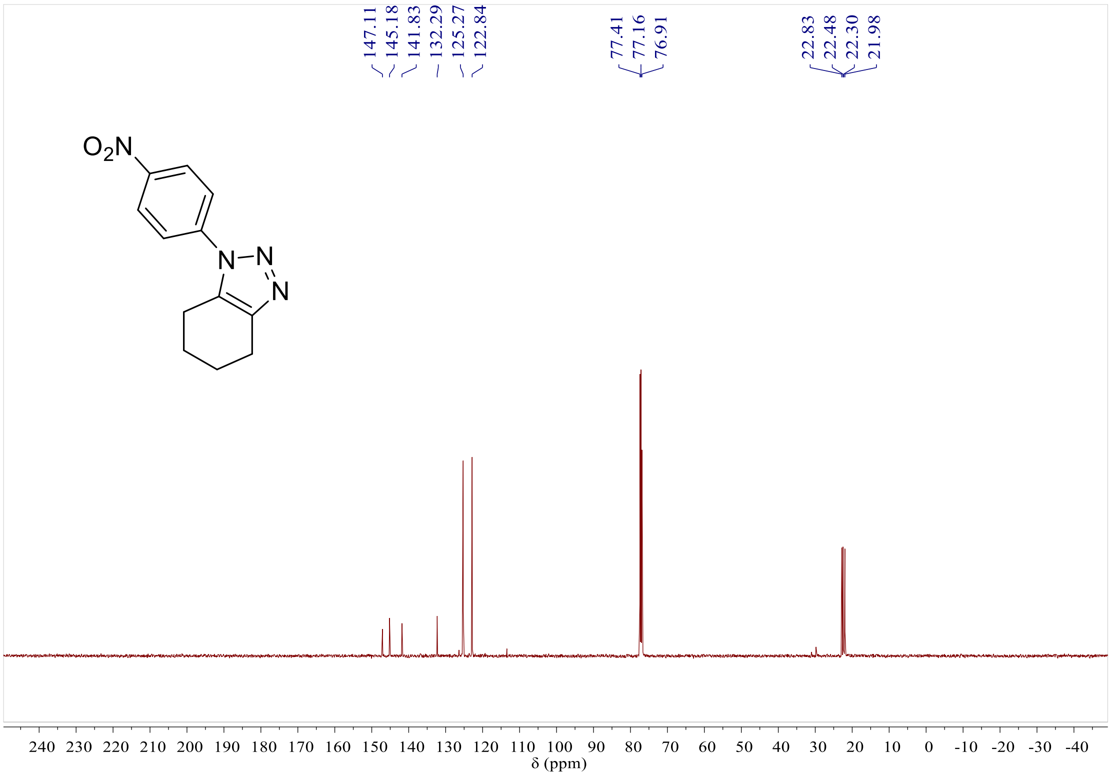

$^{13}C\{^{1}H\}$ NMR spectrum of **3te** in $CDCl_3$ (125 MHz)

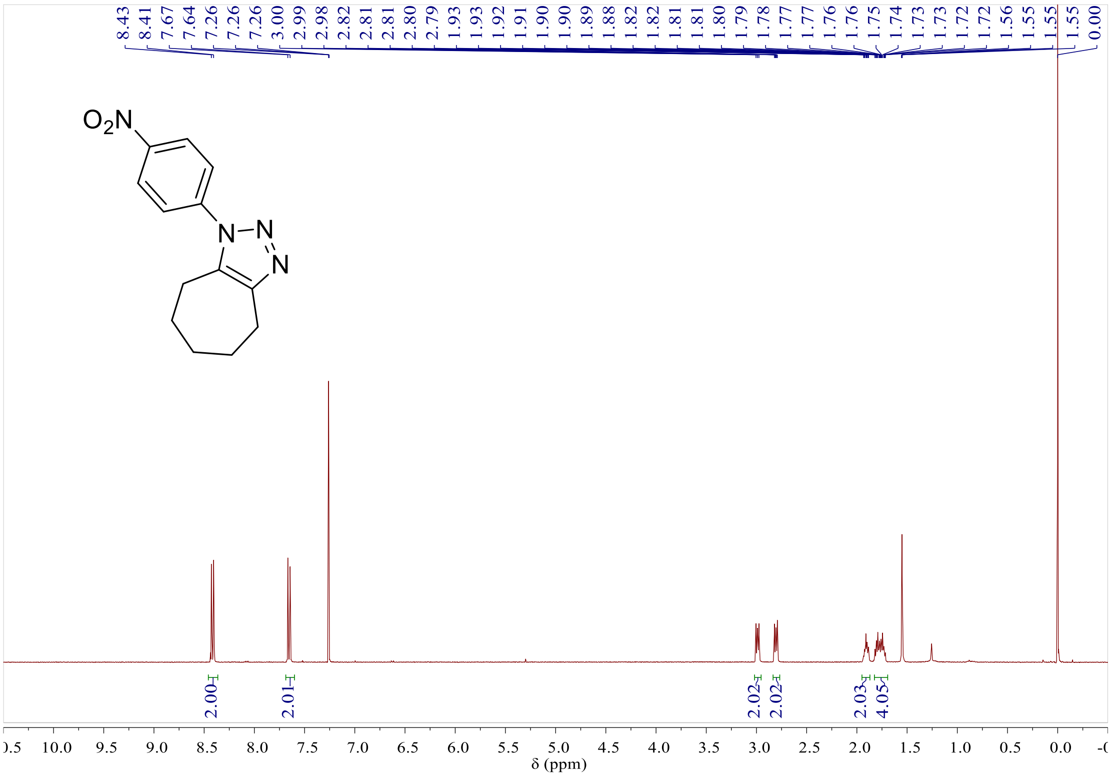

^{1}H NMR spectrum of **3ue** in $CDCl_3$ (400 MHz)

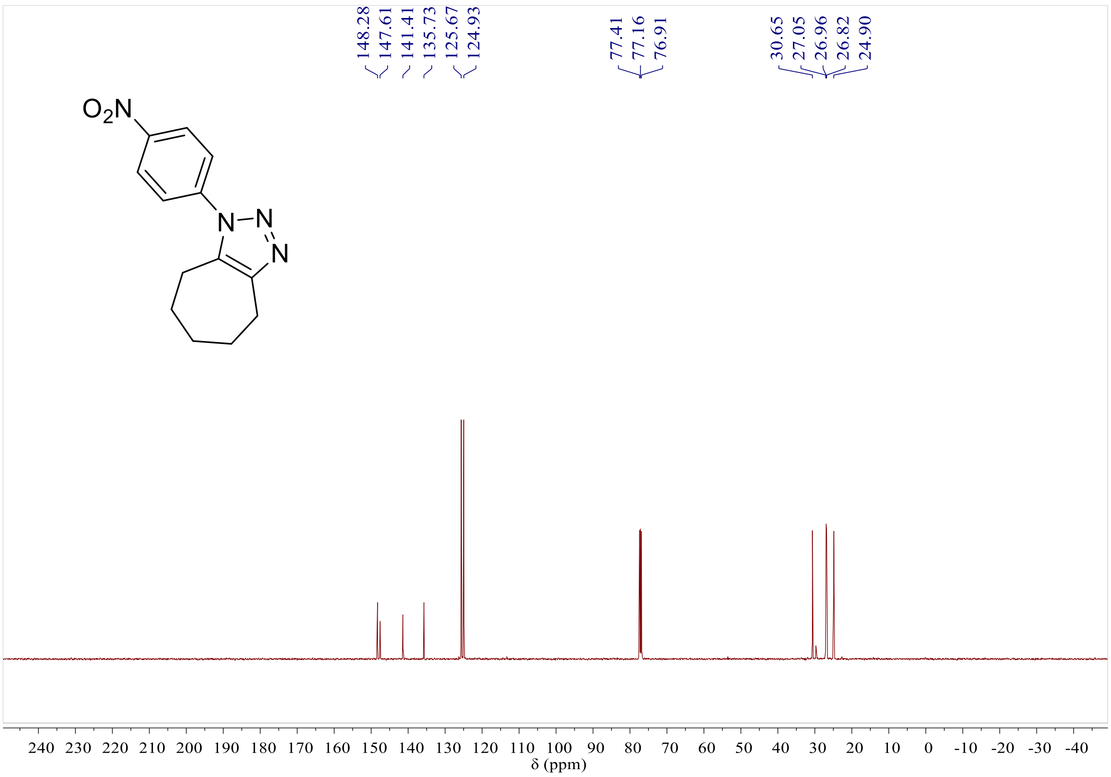

$^{13}C\{^{1}H\}$ NMR spectrum of **3ue** in $CDCl_3$ (125 MHz)

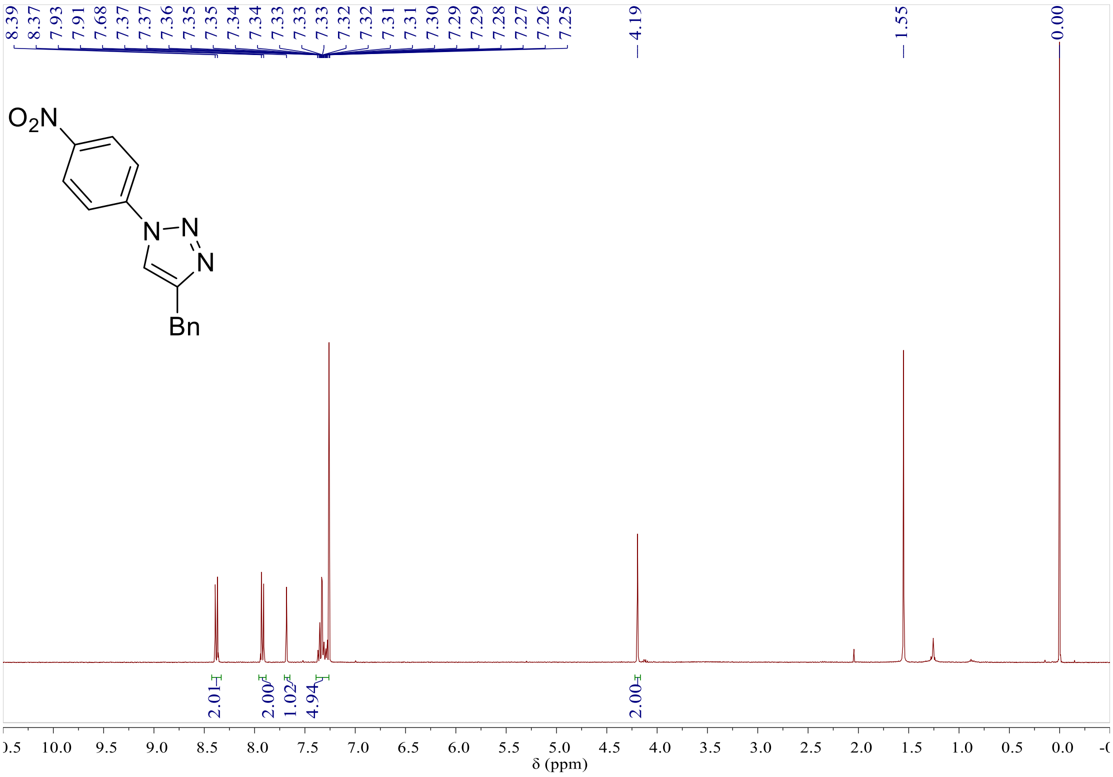

^{1}H NMR spectrum of **3ve** in $CDCl_3$ (400 MHz)

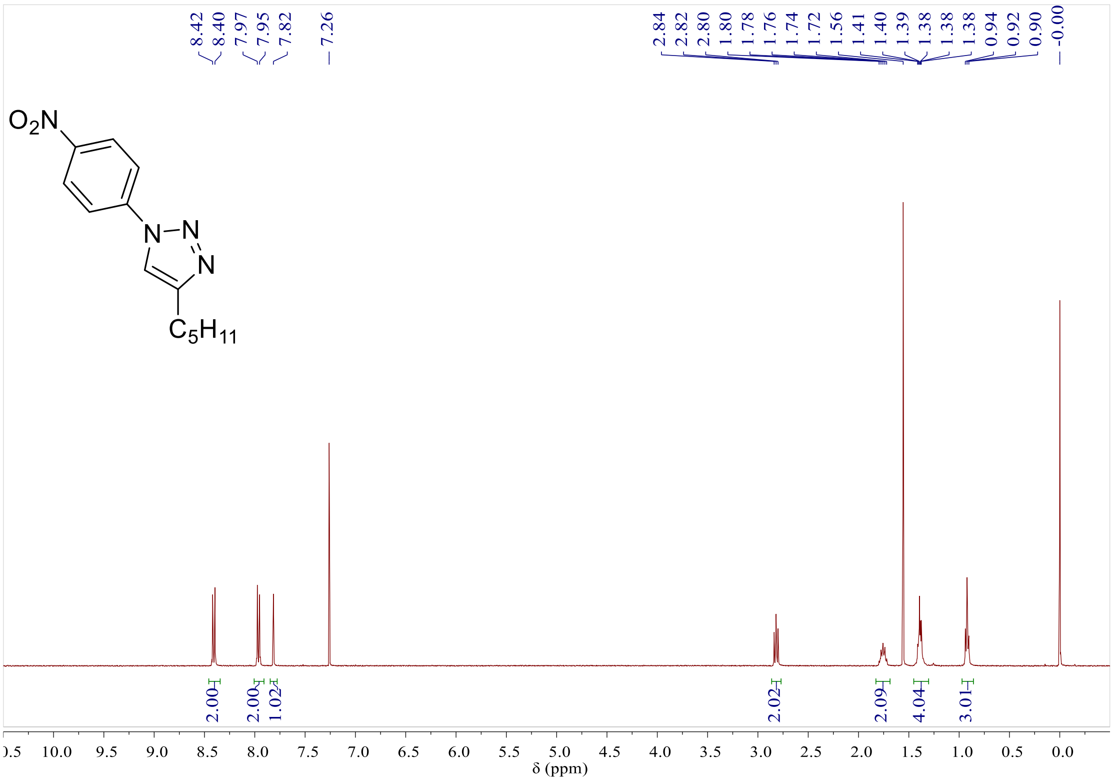

^{1}H NMR spectrum of **3we** in $CDCl_3$ (400 MHz)

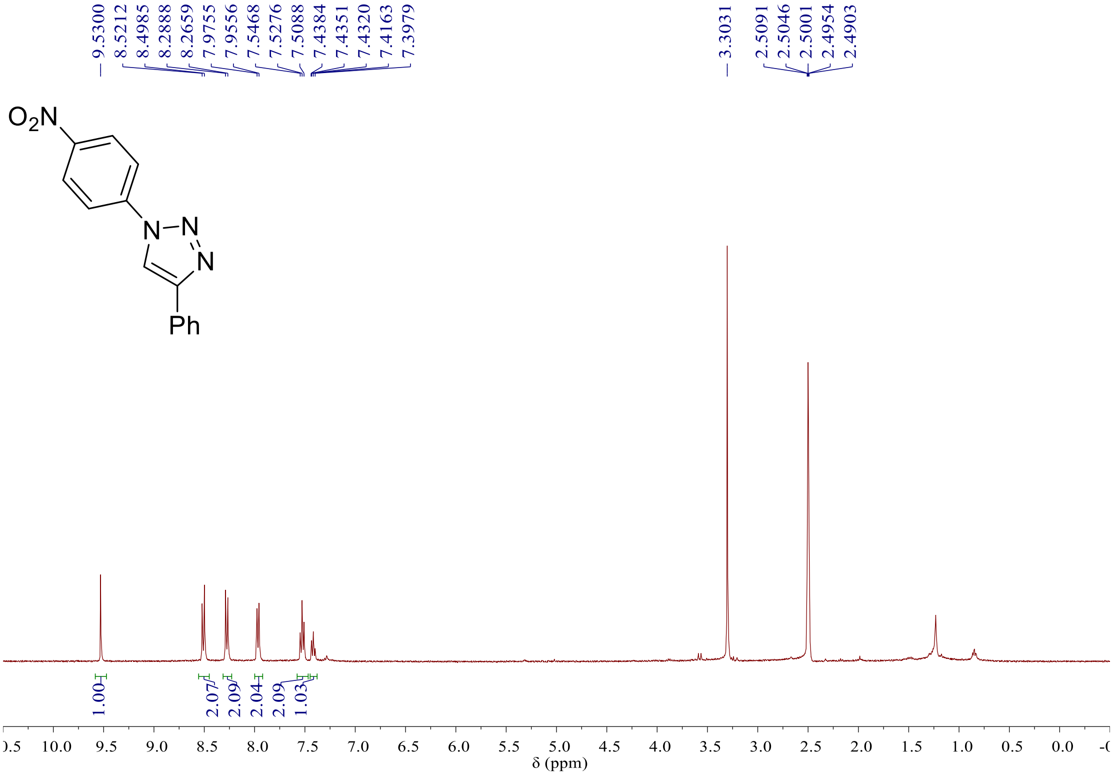

^{1}H NMR spectrum of **3xe** in DMSO (400 MHz)

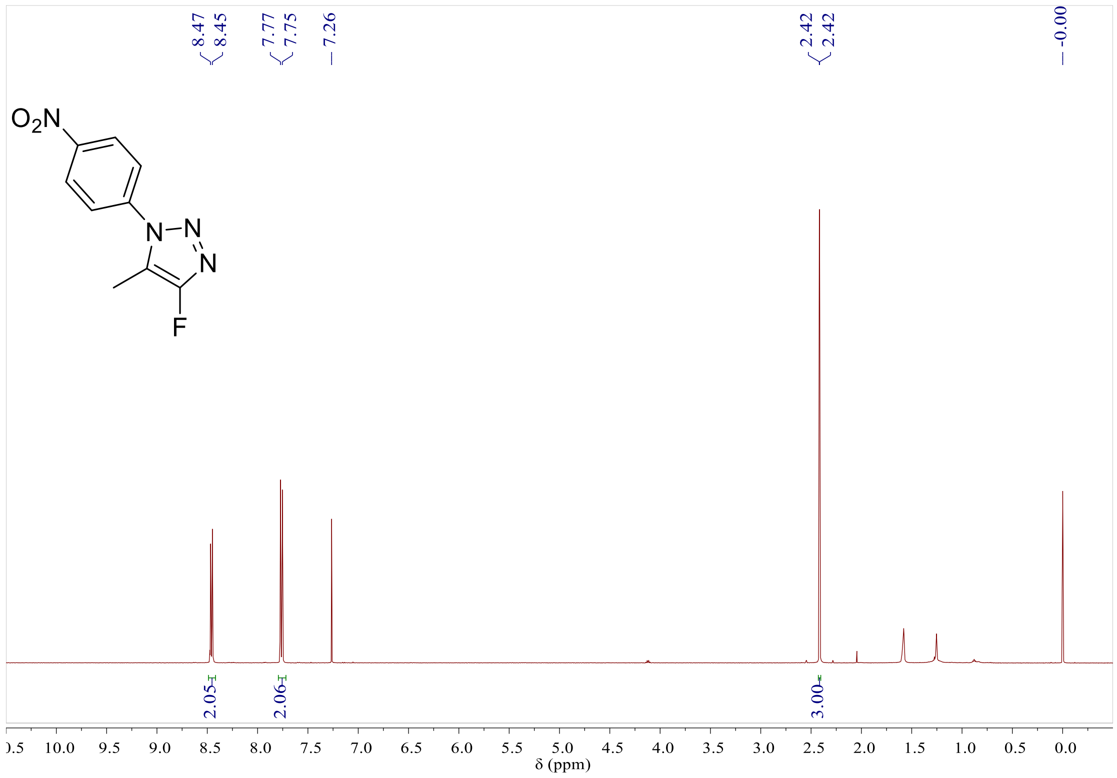

^{1}H NMR spectrum of **3ye** in $CDCl_3$ (500 MHz)

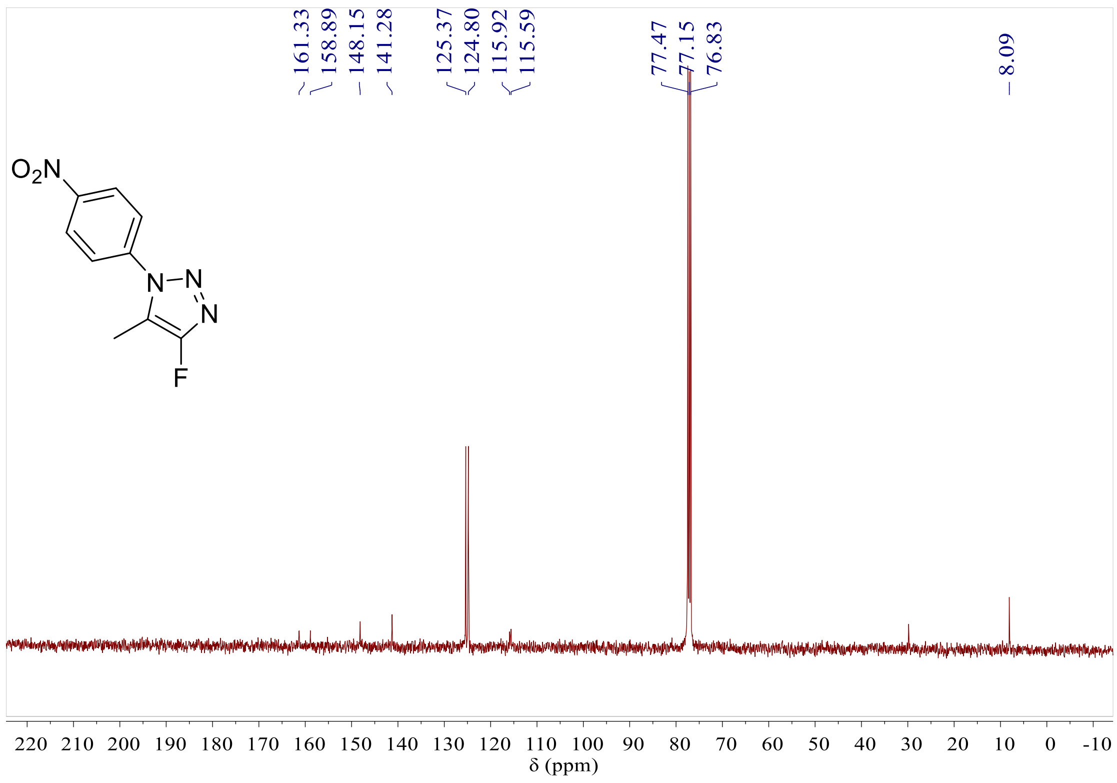

^{13}C{^{1}H} NMR spectrum of **3ye** in $CDCl_3$ (100 MHz)

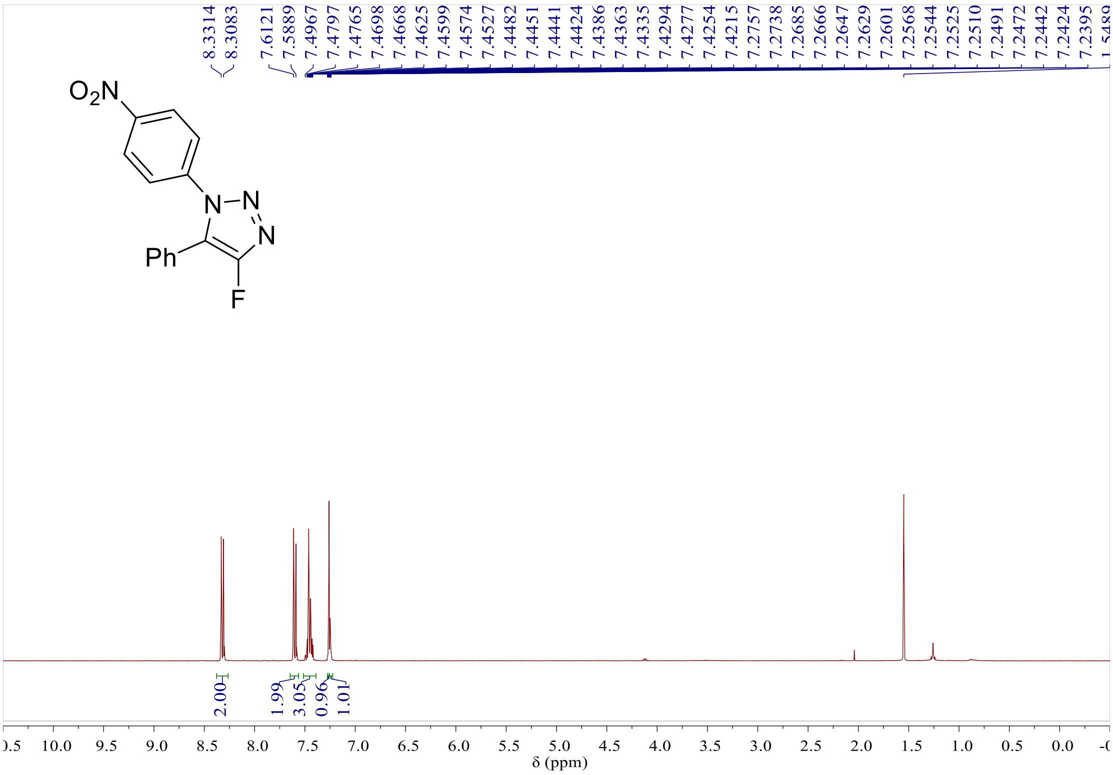

^{1}H NMR spectrum of **3ze** in $CDCl_3$ (400 MHz)

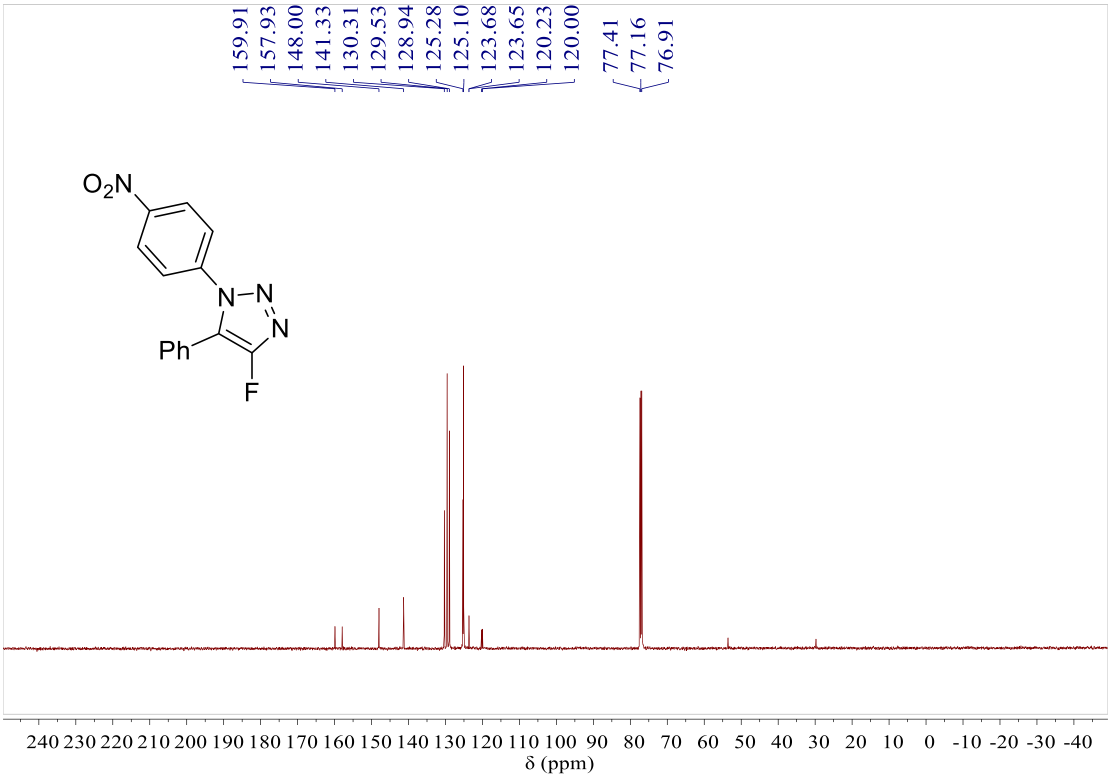

$^{13}C\{^{1}H\}$ NMR spectrum of **3ze** in $CDCl_3$ (125 MHz)

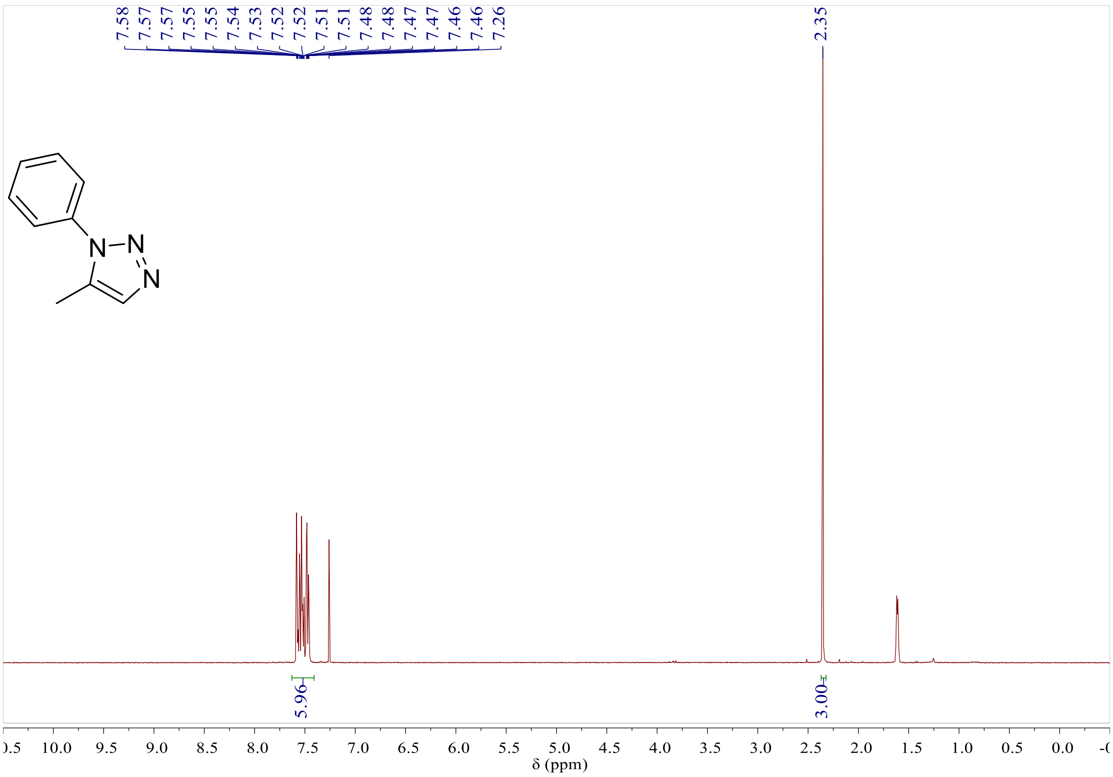

^{1}H NMR spectrum of **5aa** in $CDCl_3$ (400 MHz)

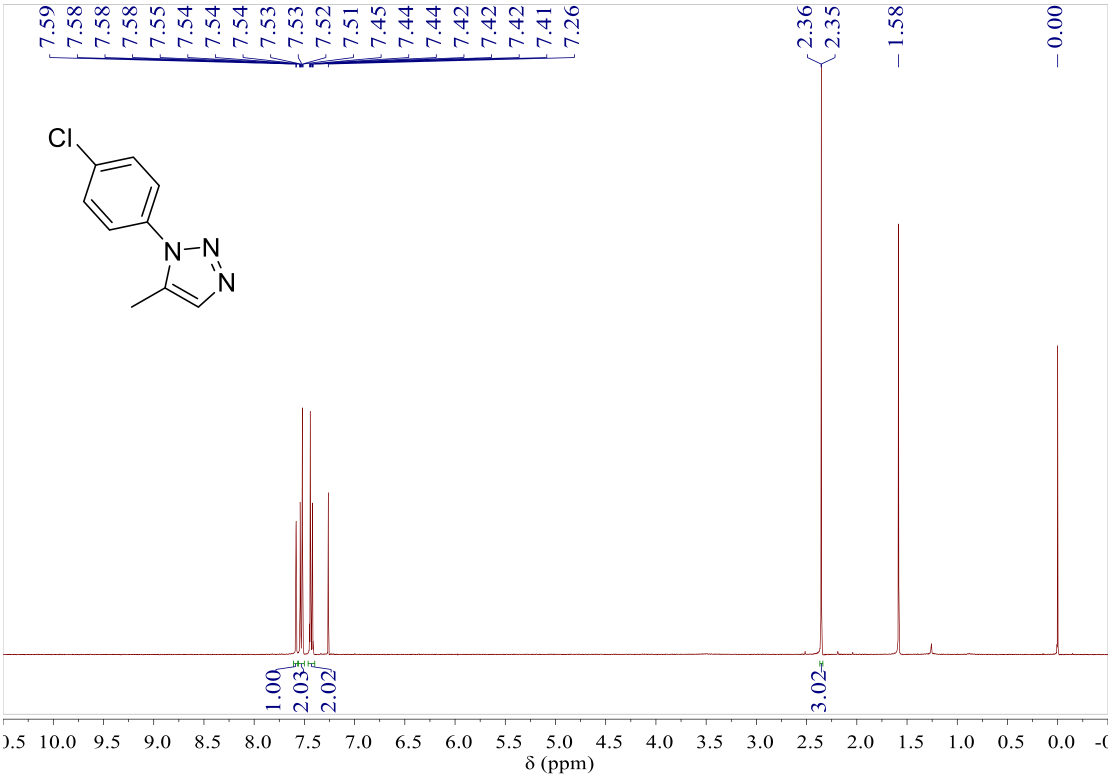

^{1}H NMR spectrum of **5ab** in $CDCl_3$ (400 MHz)

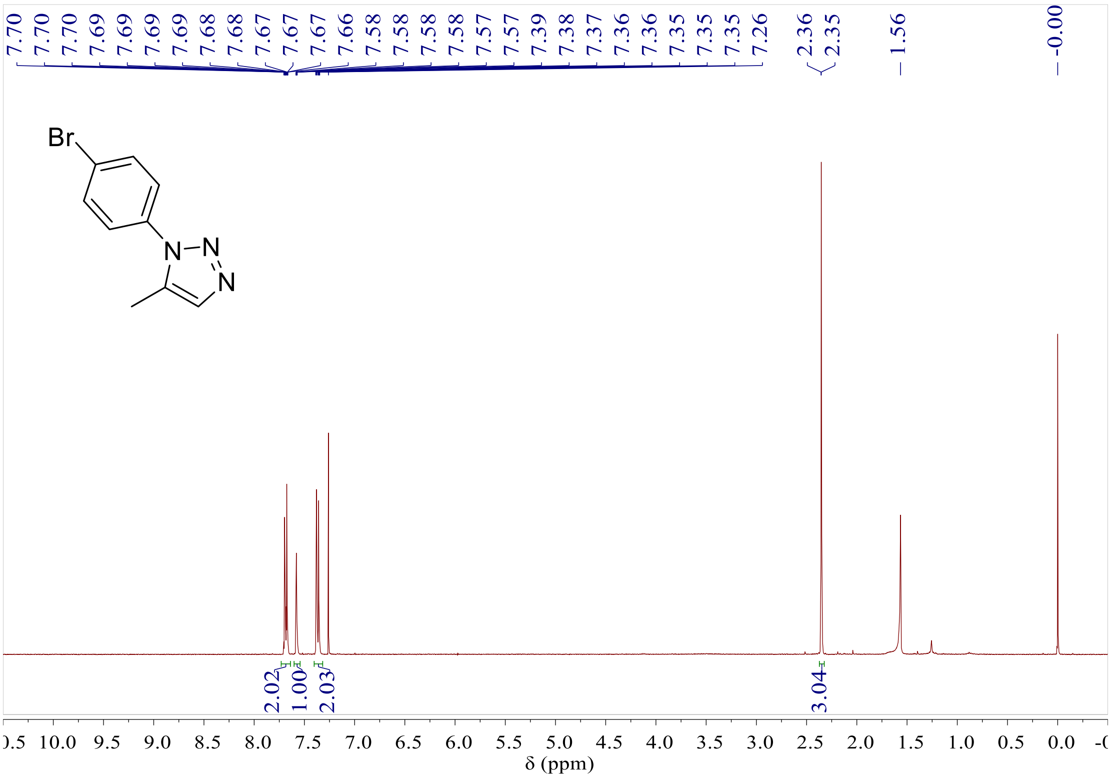

^{1}H NMR spectrum of **5ac** in $CDCl_3$ (400 MHz)

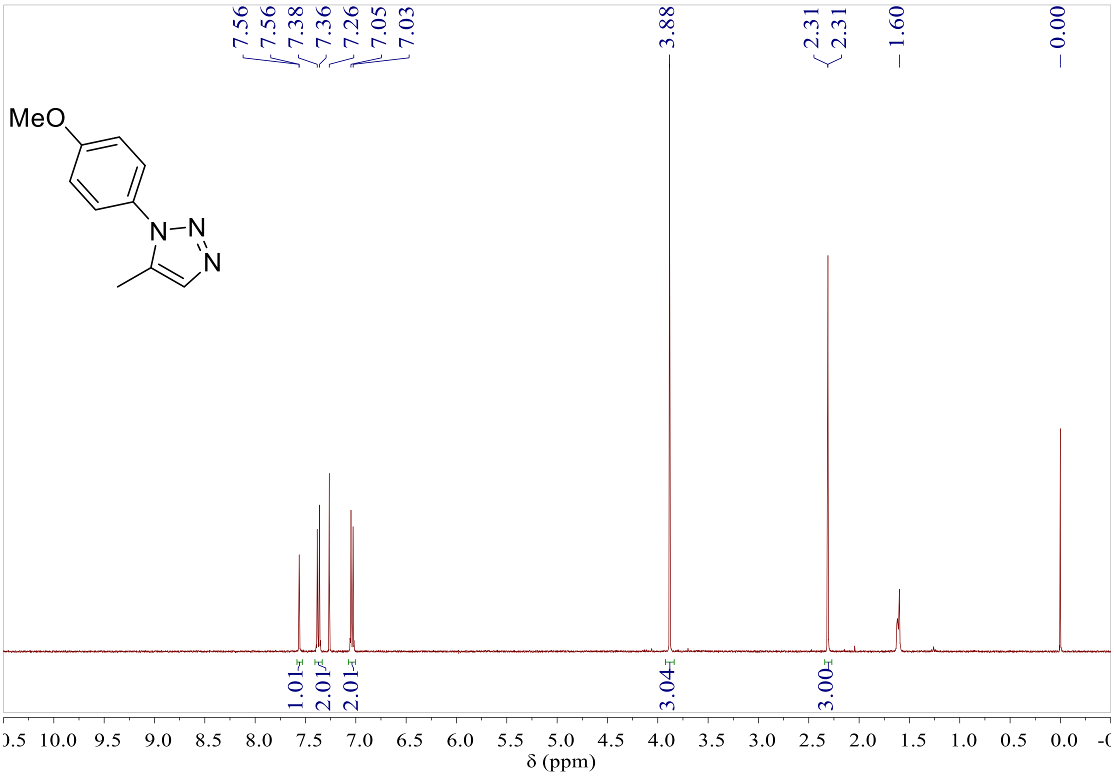

^{1}H NMR spectrum of **5ad** in $CDCl_3$ (400 MHz)

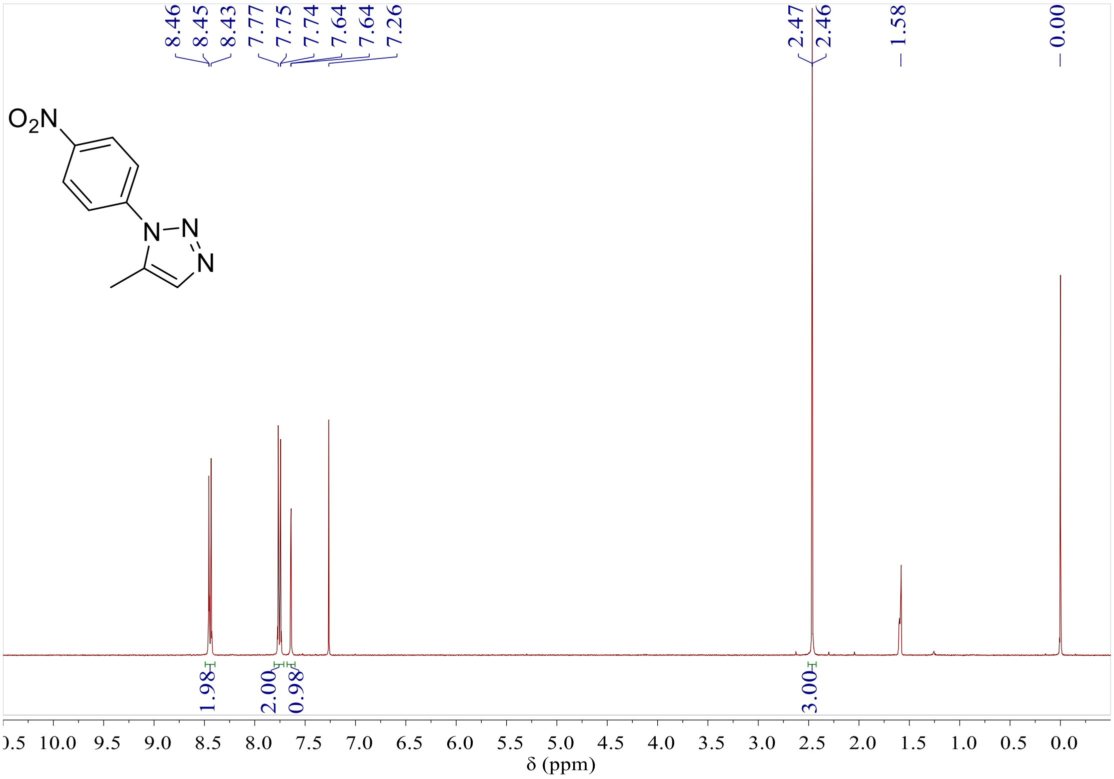

^{1}H NMR spectrum of **5ae** in $CDCl_3$ (400 MHz)

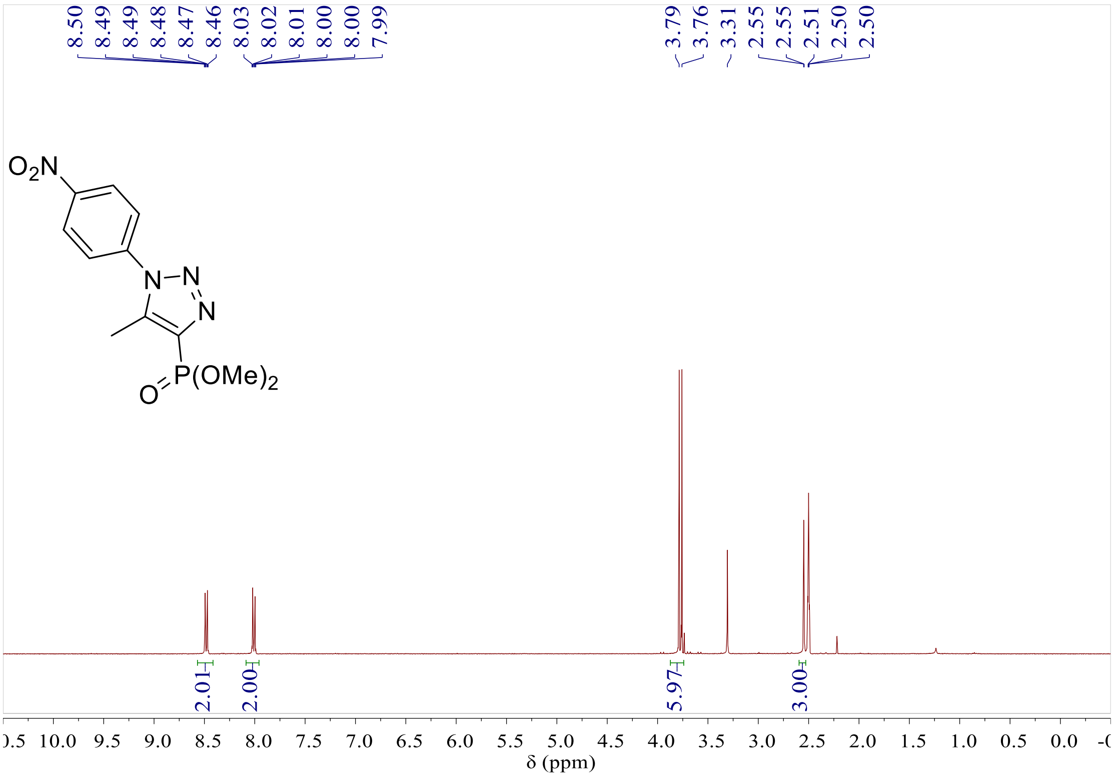

^{1}H NMR spectrum of **5ae'** in DMSO (400 MHz)

8 Crystal data and structure refinement

Crystal data and structure refinement for compound 6

Table 1.

Identification code	21oct66_1_0m_a_sq	
Empirical formula	$C_{144}H_0Cs_{12.49}O_{48}P_{12}$	
Formula weight	4529.41	
Temperature	100(2) K	
Wavelength	0.71073 Å	
Crystal system	Triclinic	
Space group	P-1	
Unit cell dimensions	*a* = 13.549(3) Å	*α* = 77.114(4)°.
	b = 16.081(3) Å	*β* = 74.841(4)°.
	c = 23.272(5) Å	*γ* = 68.005(4)°.
Volume	4493.4(15) $Å^3$	
Z	1	
Density (calculated)	1.674 Mg/m^3	
Absorption coefficient	2.678 mm^{-1}	
F(000)	2115	
Crystal size	0.186 × 0.069 × 0.030 mm^3	
θ range for data collection	2.442 to 28.973°.	
Index ranges	$-18 \leq h \leq 18$, $-21 \leq k \leq 21$, $-31 \leq l \leq 31$	
Reflections collected	106274	
Independent reflections	23126 (R_{int} = 0.0517)	
Completeness to *θ* = 25.242°	99.7 %	
Absorption correction	Numerical	
Max. and min. transmission	0.7457 and 0.5341	
Refinement method	Full-matrix least-squares on F^2	
Data / restraints / parameters	23126 / 6 / 1027	
Goodness-of-fit on F^2	1.092	
Final R indices [I>2*σ*(I)]	R_1 = 0.0695, wR_2 = 0.1642	
R indices (all data)	R_1 = 0.0780, wR_2 = 0.1699	
Extinction coefficient	n/a	
Largest diff. peak and hole	3.777 and -2.390 $e \cdot Å^{-3}$	

SC-XRD-Determined Structure of Cesium Enolate 6

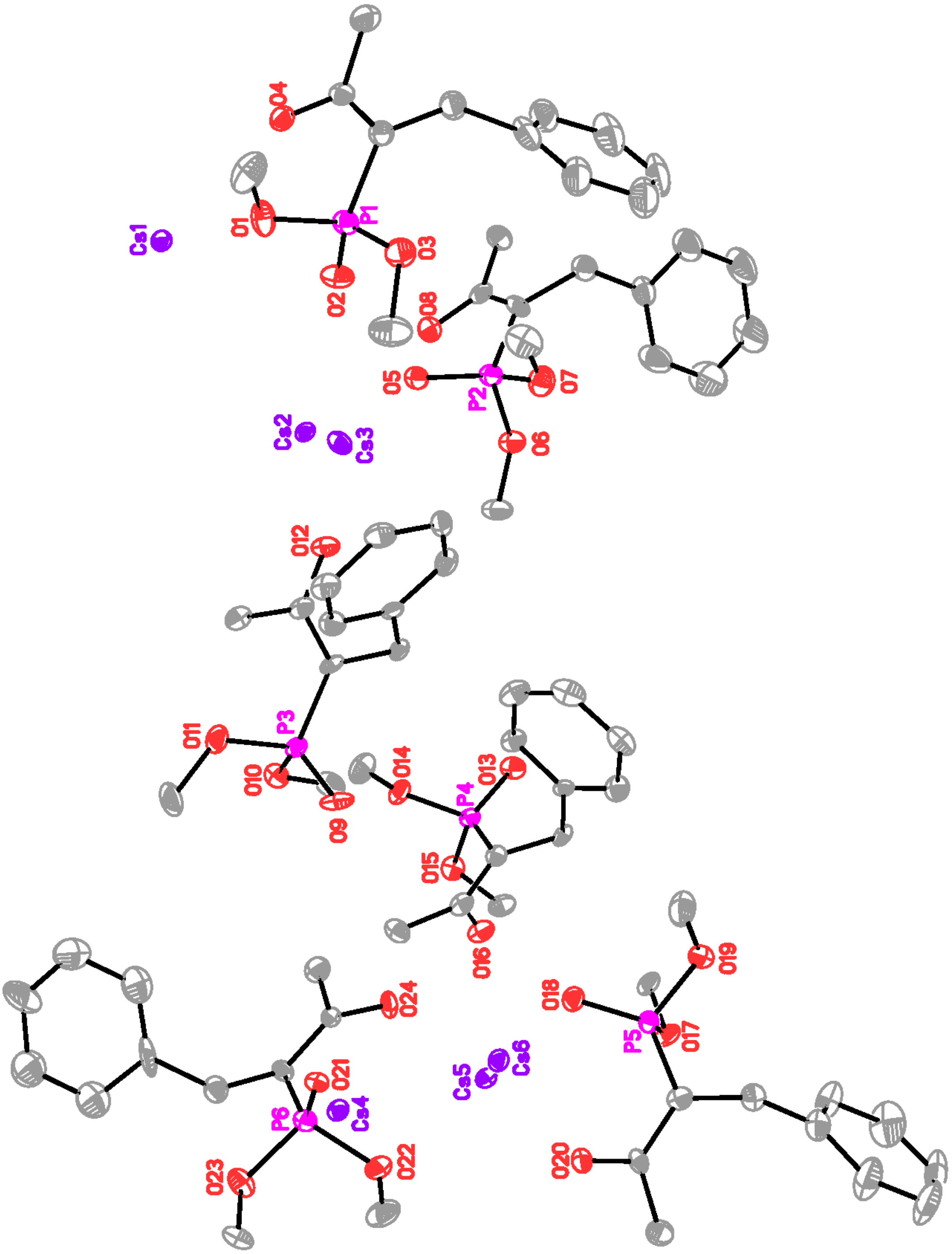

Figure S4. SC-XRD-Determined structure of cesium enolate **6** with hydrogen atoms not shown. Thermal ellipsoids are shown at a 50% probability level.

Crystal data and structure refinement for Compound 7.

Table 1.

Identification code	21NOV73_0m_a	
Empirical formula	$C_{20}H_{40}Cs_4O_{16}P_4$	
Formula weight	1192.04	
Temperature	100(2) K	
Wavelength	0.71073 Å	
Crystal system	Monoclinic	
Space group	$P2_1$	
Unit cell dimensions	*a* = 11.6555(13) Å	α = 90°.
	b = 14.3893(15) Å	β = 107.318(3)°.
	c = 11.9656(13) Å	γ = 90°.
Volume	1915.8(4) $Å^3$	
Z	2	
Density (calculated)	2.066 Mg/m^3	
Absorption coefficient	4.002 mm^{-1}	
F(000)	1136	
Crystal size	0.394 × 0.076 × 0.060 mm^3	
θ range for data collection	2.276 to 28.298°.	
Index ranges	$-15 \leq h \leq 15$, $-19 \leq k \leq 19$, $-15 \leq l \leq 15$	
Reflections collected	30740	
Independent reflections	9508 (R_{int} = 0.0298)	
Completeness to θ = 25.242°	99.9 %	
Absorption correction	Numerical	
Max. and min. transmission	0.7457 and 0.5916	
Refinement method	Full-matrix least-squares on F^2	
Data / restraints / parameters	9508 / 1 / 418	
Goodness-of-fit on F^2	1.030	
Final *R* indices [I>2σ(I)]	R_1 = 0.0153, wR_2 = 0.0346	
R indices (all data)	R_1 = 0.0158, wR_2 = 0.0348	
Absolute structure parameter	0.013(5)	
Extinction coefficient	n/a	
Largest diff. peak and hole	0.443 and -0.281 $e \cdot Å^{-3}$	

SC-XRD-Determined Structure of Cesium Enolate 7

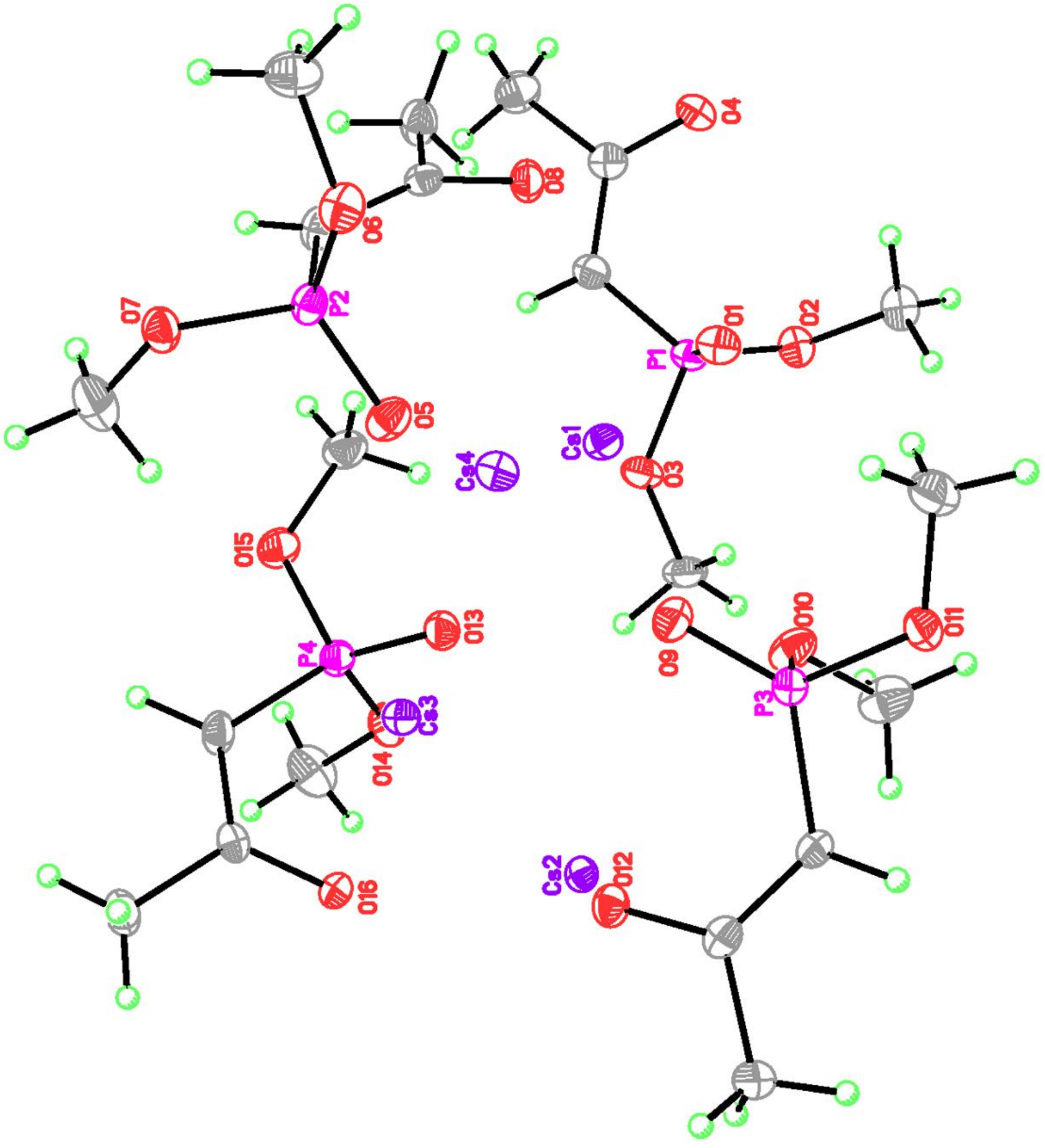

Figure S5. SC-XRD-Determined structure of cesium enolate **7**. Thermal ellipsoids are shown at a 50% probability level.
